# Radical‐Polar Crossover Bicyclization Enables a Modular Synthesis of Saturated Bicyclic Amines

**DOI:** 10.1002/advs.202501310

**Published:** 2025-04-25

**Authors:** Dewei Feng, Xiao Geng, Lingling Zuo, Zhifang Li, Lei Wang

**Affiliations:** ^1^ Advanced Research Institute and School of Pharmaceutical Sciences Taizhou University Jiaojiang Zhejiang 318000 P. R. China; ^2^ Key Laboratory of Organosilicon Chemistry and Material Technology of Ministry of Education Hangzhou Normal University Hangzhou Zhejiang 311121 P. R. China

**Keywords:** bicyclization, cyclopropylamine, photochemical, radical‐polar crossover, saturated bicyclic amine

## Abstract

The rapid assembly of diverse cyclic amines from simple precursors is now considered as an ideal platform with respect to efficiency and sustainability. To date, numerous synthetic methods have been successfully developed however, most of them are limited to a narrow subset of cyclic amines, with variations in ring size often requiring different substrates and distinct synthetic strategies. Furthermore, the “escape‐from‐Flatland” concept has led chemists to focus on the synthesis of C(sp^3^)‐rich small molecules for potential drug candidates. Herein, the successful realization of a radical‐polar crossover bicyclization reaction is reported from easily available cyclopropylamines and substituted alkenes through photoredox catalysis. This approach introduces an innovative methodology for the *de novo* synthesis of a diverse collection of 4/5‐, 5/5‐, 6/5‐,7/5‐, and 5/6‐fused saturated bicyclic amines in a systematic and modular manner with excellent diastereoselectivity. This work highlights the efficiency and utility of the photoinduced radical–polar crossover bicyclization, the applicability of which is showcased by excellent functional group tolerance, wide substrate scopes, and simple derivatization reactions.

## Graphical abstract

Herein, we report the successful realization of a radical‐polar crossover bicyclization reaction from easily available cyclopropylamines and substituted alkenes through photoredox catalysis. This approach introduces an innovative methodology for the de novo synthesis of a diverse collection of 4/5‐, 5/5‐, 6/5‐,7/5‐ and 5/6‐fused saturated bicyclic amines in a systematic and modular manner with excellent diastereoselectivity.

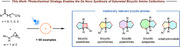

## 1 Introduction

**Figure 1:**
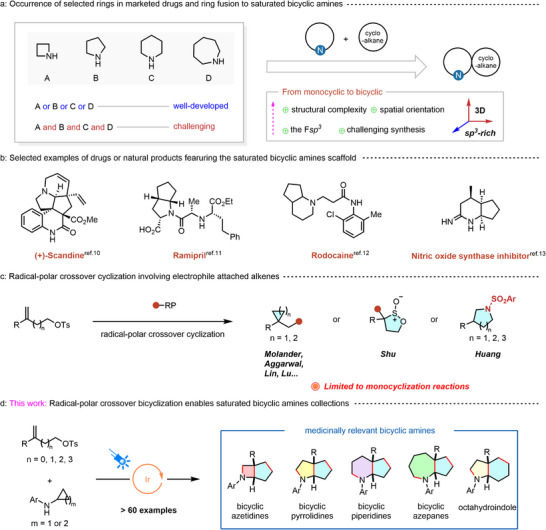
The reported method for the construction of saturated bicyclic amines and this method. a) Occurrence of selected rings in marketed drugs and ring fusion to saturated bicyclic amines. b) Selected examples of drugs or natural products containing a similar scaffold. c) Radical‐polar crossover cyclization involving electrophile attached alkenes. d) This works: photochemical strategy enables the *de novo* synthesis of 4/5‐, 5/5‐, 6/5‐, 7/5, and 5/6‐fused saturated bicyclic amines.

Cyclic amines — particularly azetidines, piperidines, pyrrolidines, and azepanes — have emerged as cornerstone compounds in the pharmaceutical and agrochemical industries, with their popularity reflected in the latest database of U.S. FDA‐approved small molecule drugs.^[^ ^1^, ^2^ ^]^ Hence, advances in the synthetic chemistry of cyclic amines are crucial for facilitating the development of innovative pharmaceuticals. Despite the fact that many efficient synthetic methods have been successfully developed over the past few decades, most of them are restricted to a narrow subset of cyclic amines.^[^ ^3^, ^4^ ^]^ Variations in ring size (particularly 4‐, 5‐, 6‐, and 7‐membered ring collections) often require different substrates and distinct synthetic strategies, which constitute a rate‐limiting factor in drug discovery (**Figure** **1**a, left).

Additionally, in modern drug discovery, a trend has emerged to increase the fraction of *sp^3^* (F*sp^3^*)‐hybridized carbons of potential drug candidates, as these often improve the biological activity of lead molecules while markedly reducing the rates of compound attrition.^[^ ^5^, ^6^, ^7^ ^]^ Looking ahead, strategies toward the introduction of cycloalkanes not only increase the F*sp^3^*, but also augment the molecular complexity, they engender are important means of offering greater opportunities for fine‐tuning of substituents while still providing a rigid, metabolically robust low molecular weight framework (Figure 1a, right).^[^ ^8^, ^9^ ^]^

Cyclopropane‐fused cyclic amines, a particularly relevant class of derivatives, possessing unique structural and physicochemical properties, are widely found in an array of natural products and pharmaceuticals (Figure 1b).^[^ ^10^, ^11^, ^12^, ^13^ ^]^ Traditional synthetic strategies for such bicyclic systems include hydrogenation,^[^ ^14^, ^15^, ^16^ ^]^ rearrangement,^[^ ^17^, ^18^ ^]^ cyclization,^[^ ^19^, ^20^, ^21^, ^22^, ^23^, ^24^ ^]^ and cycloaddition.^[^ ^25^, ^26^ ^]^ While being extensively studied, special substrates possessing at least one already‐formed ring are required, resulting in low synthetic efficiency and elaborate synthetic efforts. In insight reports, visible‐light‐mediated intramolecular radical cyclization (*aza* Paternm–Bgchi reaction) offers an ideal method toward saturated bicyclic azetidines.^[^ ^27^, ^28^, ^29^, ^30^ ^]^ This approach facilitates the simultaneous synthesis of bicyclic frameworks, obviating the need for already‐formed mono/bicyclic substrates that are characteristic of conventional methods. Nonetheless, its applicability can only be applied to [2 + 2] cyclization that lacks the opportunity for further modulating the ring size of cyclic amines[Supplementary-material advs12144-supitem-0001].

Inspired by the state‐of‐the‐art in radical–polar crossover (RPC),^[^ ^31^, ^32^, ^33^, ^34^, ^35^, ^36^, ^37^, ^38^, ^39^, ^40^ ^]^ especially, Molander,^[^ ^41^ ^]^ Aggarwal,^[^ ^42^, ^43^ ^]^ Shu,^[^ ^44^, ^45^ ^]^ Huang,^[^ ^46^ ^]^ Lin,^[^ ^47^ ^]^ and Lu^[^ ^48^ ^]^ developed a library of attractive approaches to synthesize saturated carbocyclic and heterocyclic compounds from electrophile attached alkenes under mild conditions. However, these advancements are limited to monocyclization reactions, and the bicyclization variants — that are obviously more challenging — remain rare in contemporary synthetic methodology. We are interested in exploring wherther the bicyclization reaction between electrophile attached alkenes and *N*‐aryl cyclopropylamines would happen, as this would be much more synthetically useful for constructing a library of bicyclic amines with different ring sizes. Nevertheless, some challenges need to be addressed: (1) cyclopropylamines predominantly undergo 1,3‐difunctionalization reactions, whereas trifunctionalization is rare and poses a great challenge;^[^ ^49^, ^50^, ^51^, ^52^, ^53^, ^54^, ^55^, ^56^ ^]^ (2) cyclization reactions often require correct spacing between two reactive sites, a general approach to cyclic amines with different ring sizes, particularly those encompassing small to medium size rings, is challenging.

Herein, we present our distinctive discovery in the photoinduced bicyclization of cyclopropylamines with readily available substituted alkenes (Figure 1d). The radical–polar crossover bicyclization (RPCBC) offers a straightforward approach to the *de novo* synthesis of diverse saturated bicyclic amines. In particular, pharmaceutically relevant 4/5‐, 5/5‐, 6/5‐, 7/5‐, and 5/6‐fused saturated bicyclic amines could be directly elaborated in a predictable and modular manner from a common set of reagents under uniform reaction conditions with excellent diastereoselectivity— a task that is otherwise challenging using established synthetic methods.

## 2 Reaction Optimization

The evaluation of reaction conditions commenced with cyclopropylamines (**1a**), substituted alkene (**2a**) as the model substrates (**Table** **1**; see the ESI for details). Pleasingly, under blue LEDs irradiation for 24 h, the desired saturated bicyclic amine **3** could be obtained in 81% isolated yield with excellent diastereoselectivity (dr>20:1) in the presence of Ir(dtbbpy)(ppy)_2_PF_6_ (PC1, 1 mol%), K_2_HPO_4_ (50 mol%), in a solution of PhCl (0.1 M) (entry 1, Table 1). The solvent of choice was found to be significant for achieving the desired reactivity and selectivity: benzene halides, namely PhCl and PhF, as solvents afforded **3** in good yields, whereas toluene, DMF, and DMA led to comparatively low yields (entries 1−5). We also tested other photocatalysts such as *fac*‐Ir((ppy)_3_, Ru(dbz)_3_(ppy)_2_ and 4CzIPN (entries 6–8), varying degrees of diminished yields were obtained. Moreover, decreasing or increasing the amount of base resulted in reduced yields to different extents (entry 9). When other alkenes (**2a’**, **2a’’**, and **2a’’’**) were used as substitutes for **2a** under the standard conditions, the desired product **3** was obtained in 53%, 68%, and 66% isolated yields, respectively (entry 10). This reaction is sensitive to the reaction atmosphere, as the reaction conducted under air afforded no product **3** (entry 11). Furthermore, control experiments confirmed that both light and photocatalyst were necessary (entries 12 and 13).

**Table 1:** Optimization of the reaction conditions^a^ ^)^.

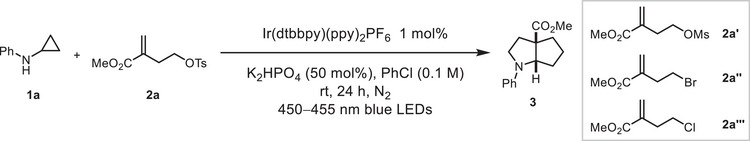		
Entry	Variation from “standard conditions”	Yield of 3 (%)^b^ ^)^
1	None	81
2	PhF instead of PhCl	80
3	Toluene instead of PhCl	63
4	DMF instead of PhCl	48
5	DMA instead of PhCl	66
6	*fac*‐Ir(ppy)_3_	69
7	Ru(dbz)_3_(ppy)_2_	18
8	4CzIPN	51
9	Without K_2_HPO_4_/100 mol% K_2_HPO_4_/200 mol% K_2_HPO_4_	71/79/67
10	**2a’**/**2a’’**/**2a’’’** instead of **2a**	53/68/66
11	Under air	
12	No light	0
13	No PC	0

^a)^ Reaction conditions: **1a** (0.2 mmol), **2a** (0.1 mmol), Ir(dtbbpy)(ppy)_2_PF_6_ (1 mol%), K_2_HPO_4_ (50 mol%), PhCl (1 mL, 0.1 M), rt, 24 h, N_2_, blue LEDs (450−455 nm);

^b)^ Isolated yield.

## 3 Substrate Scope

Under the established optimal reaction conditions, the reaction scope for saturated bicyclic pyrrolidines was examined. As shown in **Table** **2**, cyclopropylamines with various substituents on the aryl ring, including electron‐donating (Me, *n*‐Bu, OSiMe_2_ *^t^* Bu, OMe, OEt, di‐Me) and halogen (F, Cl, I), at either *para*, *meta*, or *ortho* position, also proceeded with moderate to good efficiency (**4**–**16**). The incorporation of the medicinally relevant trifluoromethoxy and trifluoromethyl groups provided the desired products **17** and **18** in 67% and 81% yields. 4‐Ph‐substituted cyclopropylamines underwent the reaction smoothly delivering product **19** in a yield of 72%. Additionally, the naphthyl‐substituted cyclopropylamines exhibited compatibility, resulting in the formation of the corresponding products with moderate to low yields (**20**, 55%; **21**, 35%). Gratifyingly, cyclopropylamine with “privileged skeleton” benzofuran group underwent the reaction smoothly and afforded the product **22** in good yield. As expected, a wide range of substituted alkenes **2** bearing different functional groups (**1b**–**1r**) participated in the reaction smoothly to afford the saturated bicyclic pyrrolidines smoothly in generally moderate to good yields. Diverse carboxylic esters substituted alkenes (**1b**–**1f**) proceeded well to give the corresponding saturated bicyclic pyrrolidines (**23**–**27**) in 64–83% yields. For the reaction of substituted aryl alkenes with different substituents on the benzene rings (**1g**–**1r**), even with potentially reactive functional groups such as cyano (**1o**), acyl (**1p**), carbonyl (**1q**) and nitro (**1r**) were suitable in the transformation (**28**–**39**), further demonstrating the diversity of substrates and pending for further modification. To expand the capacity of this approach, we considered the possibility of prolonging or shortening the alkyl chain of electrophile attached alkenes for the synthesis of bicyclic amine with different ring sizes. To our delight, this method demonstrated remarkable generality for bicyclic piperidines (**40**–**44**) under the above optimal conditions, and bicyclic azepanes (**45** and **46**) were also yielded in moderate yield by fine‐tuning reaction conditions.^[^ ^57^ ^]^ In fact, smaller heterocycles—azetidines have garnered significant recognition in the field of drug discovery for imparting favorable pharmacokinetic properties over more common 5‐, 6‐ and 7‐membered cyclic amines,^[^ ^58^, ^59^, ^60^ ^]^ yet conventional methods that are effective for synthesizing cyclic amines of other sizes often prove inadequate when it comes to accessing azetidines.^[^ ^61^, ^62^, ^63^ ^]^ To our satisfaction, by shortening the alkyl chain in substituted alkenes, electrophile attached alkene **2x** reacted smoothly with a variety of substituents on *N*‐aryl cyclopropylamines under optimal conditions, thus effectively yielding the desired bicyclic azetidines (**47**–**57**). *N*‐Phenyl cyclobutanamine was demonstrated to be a viable substrate, undergoing smooth conversion to afford the desired octahydroindole product **58** in 29% isolated yield. While this yield is comparatively modest, this result nevertheless establishes that the reaction system can accommodate the slightly larger cyclobutyl ring framework. Remarkably, the target products were obtained with excellent diastereoselectivity (dr > 20:1). Unfortunately, unsubstituted alkenes, alkyl‐substituted alkenes, internal alkenes, *N*‐phenyl cyclopentanamine, *N*‐phenyl cyclopropylether, *N*‐phenyl cyclopropylsulfide, *N*‐boc cyclopropylaniline, *N*‐benzoyl cyclopropylaniline, and N‐tosyl cyclopropylaniline were found to be incompatible with the transformation (Table 2, bottom).

**Table 2:** Substrate scope for 4/5‐, 5/5‐, 6/5‐, 7/5‐ and 5/6‐fused saturated bicyclic amines^a^ ^),^ ^b^ ^),^ ^c^ ^)^.

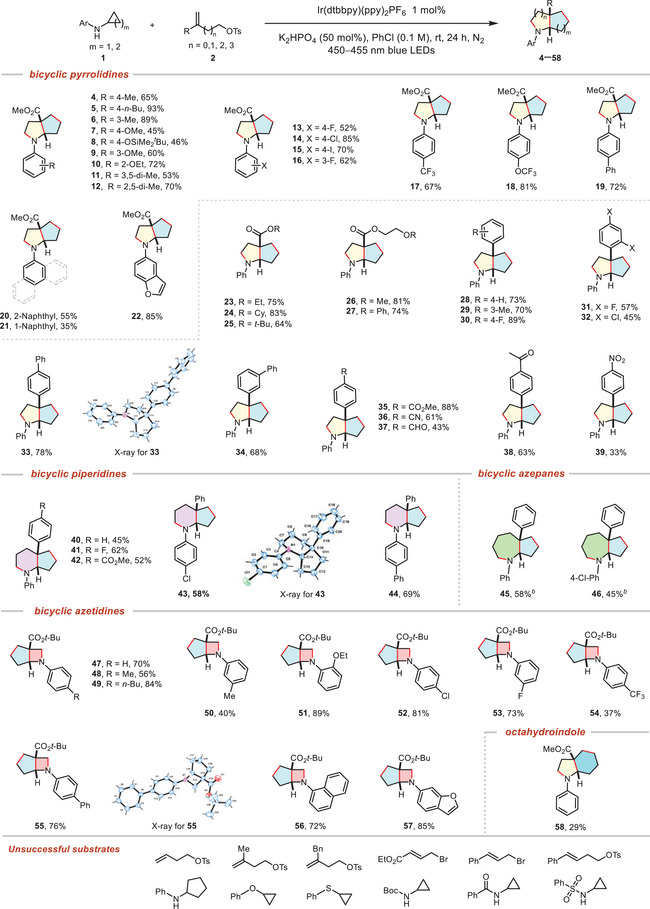

^a)^ General conditions: **1** (0.4 mmol), **2** (0.2 mmol), Ir(dtbbpy)(ppy)_2_PF_6_ (1 mol%), K_2_HPO_4_ (50 mol%), PhCl (2 mL, 0.1 M), rt, 24 h, N_2_, blue LEDs (450−455 nm);

^b)^ K_2_CO_3_ (200 mol%) as base, DMF as solvent;

^c)^ Isolated yields of products.

**Figure 2:**
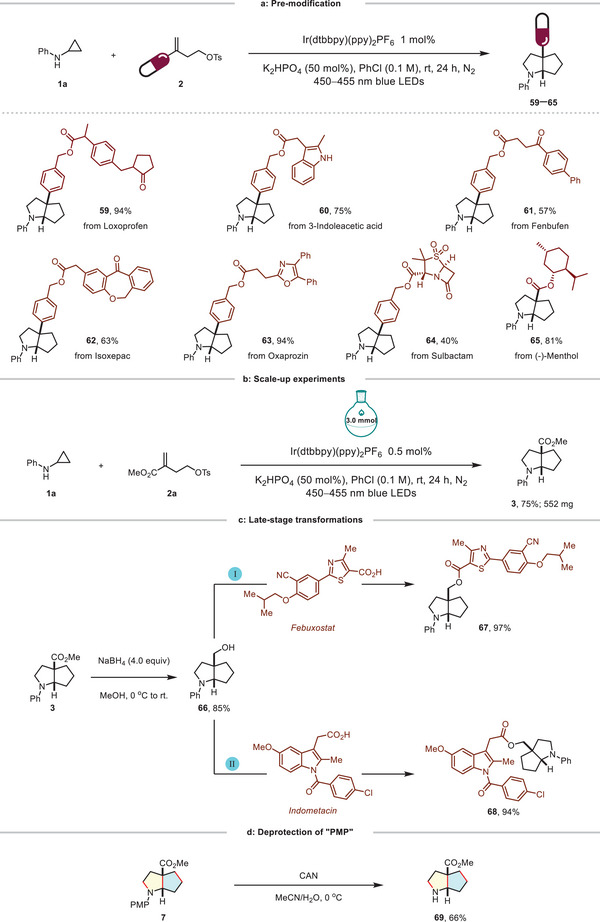
a) Pre‐modification, conditions: **1** (0.4 mmol), **2** (0.2 mmol), Ir(dtbbpy)(ppy)_2_PF_6_ (1 mol%), K_2_HPO_4_ (50 mol%), PhCl (2 mL, 0.1 M), rt, 24 h, N_2_, blue LEDs (450−455 nm), isolated yields of products. b) Gram‐scale experiments. c) Late‐stage transformations, conditions for esterification: **66** (0.2 mmol), *Febuxostat* or *Indometacin* (1.5 equiv), DMAP (0.2 equiv), EDC (2.0 equiv) in dry DCM (0.5 M), rt, 12 h. d) Deprotection of “PMP”, conditions: **7** (0.075 mmol), CAN (3.0 equiv), 0 °C, 0.5 h.

## 4 Synthetic Applications

Motivated by a wide range of successfully demonstrated substrates, we endeavored to further explore the applicability of this bicyclization reaction. Biological molecules such as Loxoprofen, 3‐Indoleacetic acid, Fenbufen, Isoxepac, and Sulbactam, derived styrenes and (‐)‐Menthol derived conjugate olefin were proved to be suitable substrates, yielding the desired product **59**−**65** in moderate to good yields (Figure 2a). The reaction was successfully scaled up to 3.0 mmol for the synthesis of product **3**, with the isolated yield maintained at a moderate level (Figure 2b). With the reduction of **3** by NaBH_4_, cycloalkyl alcohol **66** was produced in 85% yield (Figure 2c, left). The facile and effective incorporation of biological molecules, such as Febuxostat and Indometacin into product **66** rendered the beneficial impact of this synthetic methodology on pharmaceutical study (Figure 2c, right). Additionally, successful removal of the PMP group was achieved, providing the unprotected amine **69** in 66% yield (Figure 2d).

## 5 Mechanistic Studies

**Figure 3:**
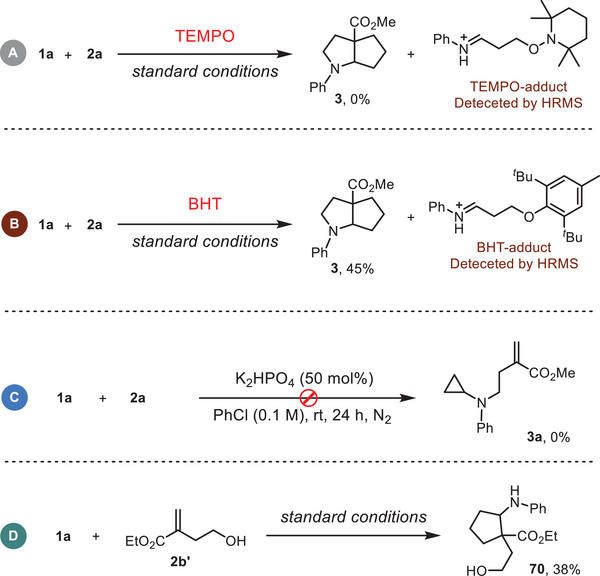
Controlled experiments.

To clarify the mechanism underlying this transformation, several control experiments were conducted (**Figure** **3**). Radical trapping experiments using (2,2,6,6‐tetramethylpiperidin 1‐yl)oxyl (TEMPO) or butylated hydroxytoluene (BHT) inhibited the production of **3** and the corresponding TEMPO/BHT‐adducts were detected by HRMS, suggesting a radical process in this transformation (Figure 3A,B, see the ESI for details). To further demonstrate the reaction sequence of this bicyclization reaction, two control experiments were conducted. Conducting the reaction in the absence of light and PC did not yield the nucleophilic substitution product **3a** (Figure 3C). Nevertheless, **1a** underwent [3 + 2] cyclization with ethyl 4‐hydroxy‐2‐methylenebutanoate (**2b’**) to produce **70** (Figure 3D), indicating that the radical cyclization might be the initial step of this transformation.

**Figure 4:**
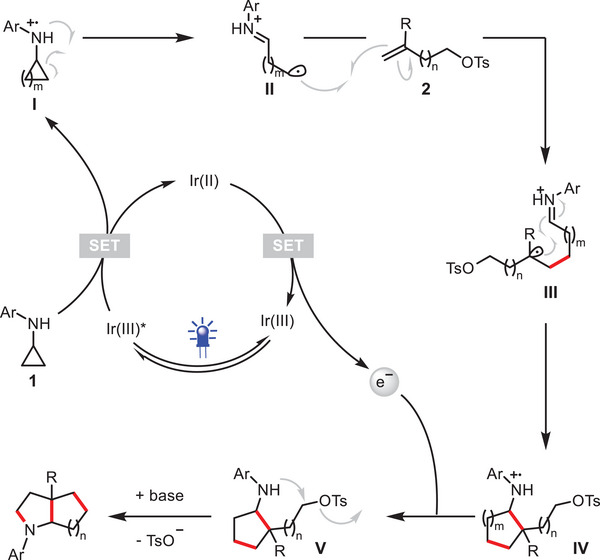
Proposed mechanism.

Based on the above mechanistic studies and previous works,^[^ ^41^, ^42^, ^43^, ^44^, ^45^, ^46^, ^47^, ^48^, ^64^ ^]^ a plausible mechanism is proposed in **Figure** **4**. First, light irradiation of Ir(dtbbpy)(ppy)_2_PF_6_ (Ir^III^) to the excited state catalyst Ir(dtbbpy)(ppy)_2_PF_6_* (Ir^III^*). Single‐electron transfer (SET) with the *N*‐aryl cyclopropylamine **1** results in reduction of the photocatalyst (Ir^II^), oxidation of **1** to radical cationic intermediate **I**, which will be transformed to alkyl radical specie **II** through a ring‐opening process. The subsequent reaction of this reactive intermediate with the substituted alkene **2** leads to the formation of a relatively stabilized distonic radical cation **III** with the radical center preferentially located on a tertiary carbon position. The facile radical cyclization provides radical cation **IV**.^[^ ^49^, ^50^, ^51^, ^52^, ^53^, ^54^, ^55^, ^56^ ^]^ A second SET between intermediates **IV** and the photocatalyst (Ir^II^), finishes the photoredox catalytic cycle and brings about intermediates **V**, which subsequently undergoes a 4, 5, 6, or 7‐*exo*‐*tet* cyclization to afford the expected saturated bicyclic amine products.

## 6 Conclusion

In summary, we have successfully developed a photocatalyzed radical‐polar crossover bicyclization for the *de novo* synthesis of a diverse collection of 4/5‐, 5/5‐, 6/5‐, 7/5‐, and 5/6‐fused saturated bicyclic amines in a systematic and modular manner under a common set of reaction conditions. The synthetic potential and practical applicability of this approach were highlighted by the flexible variations in ring sizes, good functional group tolerance, broad substrate compatibility, and excellent diastereoselectivity. This reaction pioneered the trifunctionalization of *N*‐aryl cyclopropylamines, adeptly employing them as dual alkylation and amination reagents, thereby establishing a groundbreaking synthetic pathway for the preparation of diverse saturated cyclic amine products. Given the high efficiency of this bicyclization reaction and the significant demand for saturated bicyclic amines in drug discovery, we anticipate that this method will have potential application value in the fields of synthetic and medicinal chemistry.

## 7 Experimental Section

In the glove box, a 10 mL Schlenk tube equipped with a magnetic stir bar was charged with *N*‐aryl cyclopropylamine **1** (0.4 mmol), substituted alkene **2** (0.2 mmol), Ir(dtbbpy)(ppy)_2_PF_6_ (0.91 mg, 0.001 mmol, 0.5 mol%), K_2_HPO_4_ (17.4 mg, 0.1 mmol, 50 mol%) and PhCl (2.0 mL). The reaction mixture was stirred under 2×3 W blue LEDs (λ = 450−455 nm) at room temperature with stirring for 24 h. After completion of the reaction, the solvent was removed under reduced pressure, and the residue was purified with silica gel chromatography (eluent: petroleum ether/ethyl acetate = 100:1, V/V) to give bicyclic azetidines, bicyclic pyrrolidines and bicyclic piperidines.

In the glove box, a 10 mL Schlenk tube equipped with a magnetic stir bar was charged with *N*‐aryl cyclopropylamine **1** (0.4 mmol), substituted alkene **2** (0.2 mmol), Ir(dtbbpy)(ppy)_2_PF_6_ (0.91 mg, 0.001 mmol, 0.5 mol%), K_2_CO_3_ (55.3 mg, 0.4 mmol, 200 mol%) and DMF (2.0 mL). The reaction mixture was stirred under 2×3 W blue LEDs (λ = 450−455 nm) at room temperature with stirring for 24 h. After completion of the reaction, the solution was extracted with ethyl acetate (3 × 15 mL) and the combined extracts were dried with anhydrous Na_2_SO_4_. The solvent was removed under reduced pressure, and the residue was purified with silica gel chromatography (eluent: petroleum ether/ethyl acetate = 100:1, V/V) to give bicyclic azepanes.

## Conflict of Interest

The authors declare no conflict of interest.

## Author Contributions

D.W.F. and X.G. performed the experiments. X.G. contributed to the designing and writing of the manuscript. L.L.Z. and L.W. checked up the data. Z.F.L. and L.W. reviewed and modified the manuscript. All authors approved the final version of the manuscript.

## Supporting information

Supporting Information

## Data Availability

The data that support the findings of this study are available in the supplementary material of this article.
