# Supplementary material for: Radical‐Polar Crossover Bicyclization Enables a Modular Synthesis of Saturated Bicyclic Amines
Source: Adv Sci (Weinh). 2025 Apr 25;12(25):2501310. doi: 10.1002/advs.202501310 (PMC12224996; doi:10.1002/advs.202501310)
Supplement: Supplementary file 1 — Supporting Information [file ADVS-12-2501310-s001.pdf]

ADVANCED SCIENCE
Open Access

Supporting Information

for *Adv. Sci.*, DOI 10.1002/advs.202501310

Radical-Polar Crossover Bicyclization Enables a Modular Synthesis of Saturated Bicyclic Amines

Dewei Feng, Xiao Geng, Lingling Zuo, Zhifang Li and Lei Wang**

Supporting Information

Radical-Polar Crossover Bicyclization Enables a Modular Synthesis of Saturated Bicyclic Amines

Dewei Feng,[1,2] Xiao Geng,[1,]* Lingling Zuo,[1] Zhifang Li,[2] and Lei Wang[1,2]*

1. Advanced Research Institute and School of Pharmaceutical Sciences, Taizhou University, Jiaojiang 318000, Zhejiang, P. R. China
2. Key Laboratory of Organosilicon Chemistry and Material Technology of Ministry of Education, Hangzhou Normal University, Hangzhou 311121, Zhejiang, P. R. China

Table of Contents

1. General information 2
2. Experimental procedure 3
3. Optimization reaction conditions 12
4. Characterization data of products 13
5. Gram level reaction and derivatization experiments 50
6. Mechanistic studies 54
7. X-ray structure and data for 33, 43, 55 57
8. References 62
9. NMR spectra of the products 63

1. General information

Unless otherwise noted, all commercially available compounds were used as provided without further purification. All reactions were monitored by thin-layer chromatography (TLC) on silica gel plates using UV light as visualizing agent. Compounds were visualized by irradiation with UV light or potassium permanganate staining. Flash column chromatography was performed using 200–300 or 300–400 mesh silica gel.

All ^{1}H NMR, ^{13}C NMR and ^{19}F NMR spectra were recorded on Bruker Avance III HD 400 spectrometer. Chemical shifts (δ) were reported in parts per million (ppm) relative to residual solvent peaks rounded to the nearest 0.01 for proton and 0.1 for carbon (ref: $CDCl_3$ [^{1}H: 0.00 (TMS), ^{13}C: 77.00; DMSO-d_6 [^{1}H: 2.50, ^{13}C: 39.50]. Coupling constants (J) were reported in Hz to the nearest 0.1 Hz. Peak multiplicity was indicated as follows s (singlet), d (doublet), t (triplet), q (quartet), m (multiplet). Attribution of peaks was done using the multiplicities and integrals of the peaks. High resolution mass spectroscopy data of the product were collected on the accurate masses were measured by the Q-Exactive mass spectrometer (Thermo Scientific, Sunnyvale, CA, USA) with heated electrospray ionization (HESI-II). Melting points were measured using a Stuart SMP30. Crystallographic data of product **33, 43,** and **55** were collected on Bruker SMART APEX II (Mo target, voltage 50 KV, current 30 mA). Melting points were measured using a Stuart SMP30.

2. Experimental procedure

2.1 General procedure for the synthesis of starting materials 1

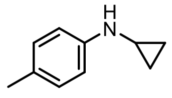

1a

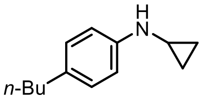

1b

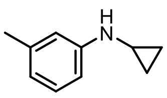

1c

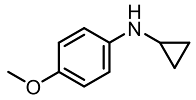

1d

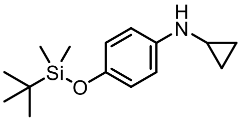

1e

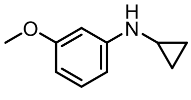

1f

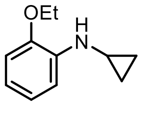

1g

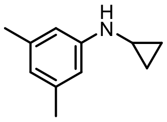

1h

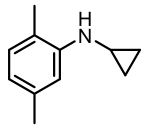

1i

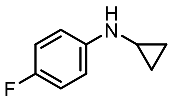

1j

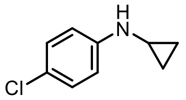

1k

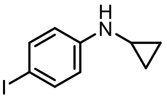

1l

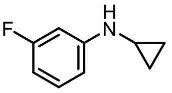

1m

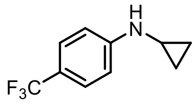

1n

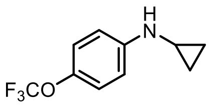

1o

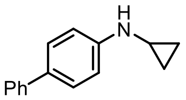

1p

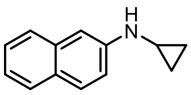

1q

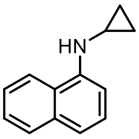

1r

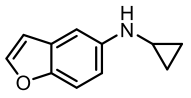

1s

Note: Substrates **1a–1s** were prepared according to the reported procedure.[1]

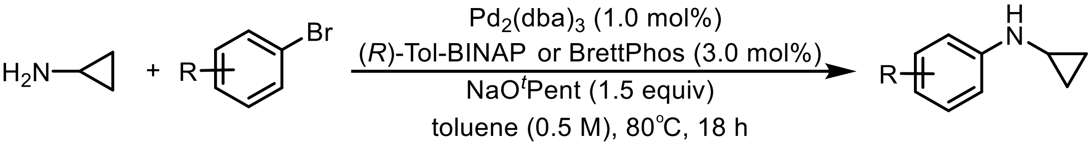

Following modified procedure,[1] an oven-dried microwave vial was charged with $Pd_2(dba)_3$ (1.0 mol%) and BrettPhos (3.0 mol%) or (*R*)-Tol-BINAP (3.0 mol%). The vial was sealed, evacuated, and back-filled with nitrogen (3 times). Then, toluene (0.5 M), cyclopropylamine (1.6 equiv.), aromatic bromide (1.0 equiv.) and NaO^{t}Pent (25% solution in toluene, 1.5 equiv.) were added via syringe to the vial, and it was heated at 80 °C overnight. The reaction mixture was then cooled to room temperature, diluted with Et_2O, and filtered through a short pad of silica. The filtrate was evaporated under reduced pressure, and the crude residue was purified by column chromatography (silica gel, petroleum ether/ethyl acetate = 100:1) to afford the desired product **1a–1s,** respectively. All recorded spectroscopic data matched those previously reported in the literature.

2.2 General procedure for the synthesis of starting materials 2

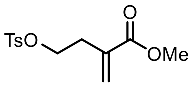

2a

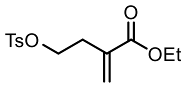

2b

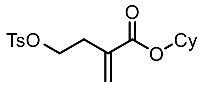

2c

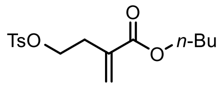

2d

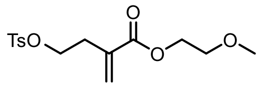

2e

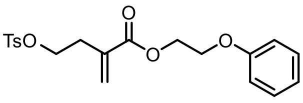

2f

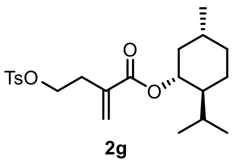

2g

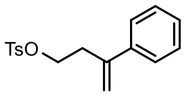

2h

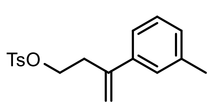

2i

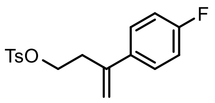

2j

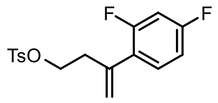

2k

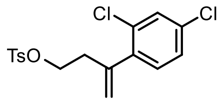

2l

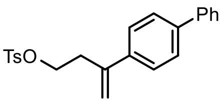

2m

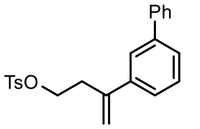

2n

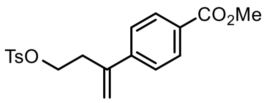

2o

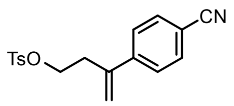

2p

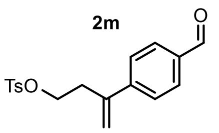

2q

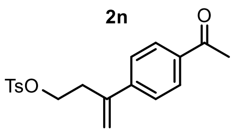

2r

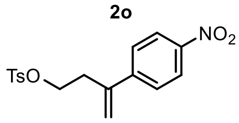

2s

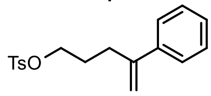

2t

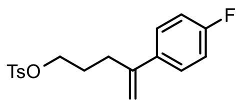

2u

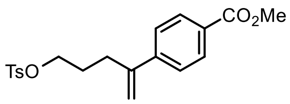

2v

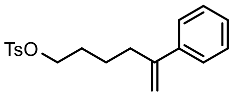

2w

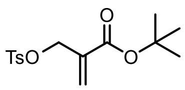

2x

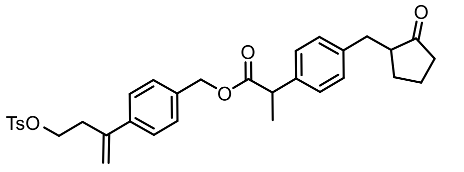

2y

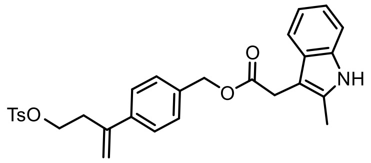

2z

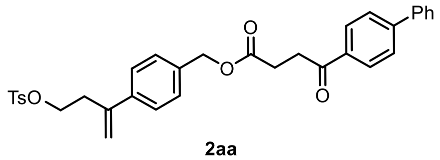

2aa

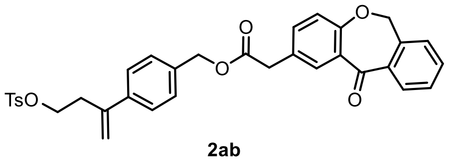

2ab

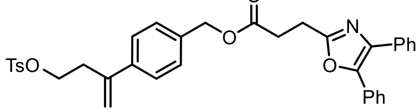

2ac

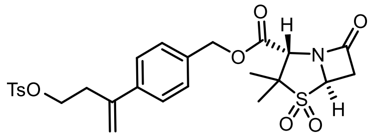

2ad

Note: Substrates **2** were prepared according to the reported procedure[2], and **2a–2s**, **2y–2ad** were known products.

General procedure A for the synthesis of 2a–2g

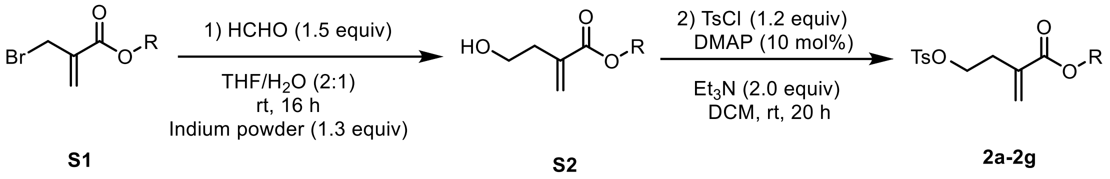

To a flask equipped with a magnetic stir bar was added **S1** (1.0 equiv.), THF and H_2O (2:1, 0.3 M) were then added with vigorous stirring, followed by formaldehyde (1.5 equiv.) and indium powder (1.3 equiv.). The reaction mixture was stirred vigorously for 16 h, then partitioned between EtOAc (30 mL) and H_2O (50 mL). The phases were separated and the aqueous phase was extracted into EtOAc (2 × 30 mL). The combined organic phases were washed with brine (50 mL), dried over $MgSO_4$, filtered, and concentrated in vacuo. The residue was purified by flash column chromatography gave **S2**.

Add **S2** (1.0 equiv.) to a flask equipped with a magnetic stir bar, followed by the addition of DCM (0.5 M), DMAP (0.1 equiv.) and *p*-toluenesulfonyl chloride (1.2 equiv.), then add triethylamine (2.0 equiv.) dropwise at 0 °C. After 20 h, aqueous 1 M HCl (20 mL) was added and the phases were separated. The aqueous phase was extracted into DCM (3 × 20 mL) and the combined organic phases dried ($MgSO_4$), filtered, and concentrated in vacuo. The crude residue was purified by column chromatography (silica gel, petroleum ether/ethyl acetate = 10:1−5:1) to afford the product **2a–2g**, respectively. All recorded spectroscopic data matched those previously reported in the literature.[2]

General procedure B for the synthesis of 2h–2w

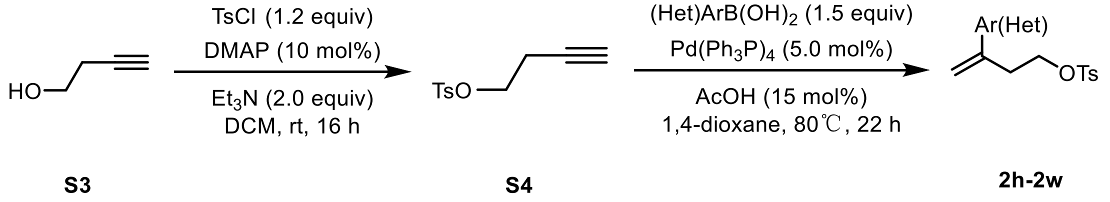

S4 was prepared following the general procedure **B** with **S3** (15.0 mmol). The crude product was purified by flash column chromatography (20% EtOAc /hexane) to

afford **S4** (3.04 g, 13.5 mmol, 90%) as a pale yellow oil. Under an N_2 atmosphere **S4** (1.12 g, 5.0 mmol, 1.0 equiv.), $Pd(PPh_3)_4$ (289 mg, 0.25 mmol, 5.0 mol%) and phenylboronic acid (914 mg, 7.5 mmol, 1.5 equiv.) were placed in a thick-walled glass vessel. 1,4-dioxane (20 mL) and AcOH (45 mg, 0.75 mmol, 15 mol%) were added and the solution was stirred at rt for 15 min, then at 80 °C for 22 h. The reaction was cooled to rt and the 1,4-dioxane was removed in vacuo. The crude residue was purified by column chromatography (silica gel, petroleum ether/ethyl acetate = 20:1~5:1) to afford the product **2h–2w**, respectively. All recorded spectroscopic data matched those previously reported in the literature.[2]

4-Phenylpent-4-en-1-yl 4-methylbenzenesulfonate (2t)

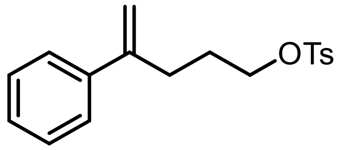

2t was prepared following the general procedure **B** with phenylboronic acid. The crude residue was purified by column chromatography (silica gel, petroleum ether/ ethyl acetate = 5:1) to afford product **2t** (1.32 g, 84% over two steps) as yellow oil.

^{1}H NMR (400 MHz, $CDCl_3$) δ 7.71 (d, J = 8.4 Hz, 2H), 7.27–7.23 (m, 5H), 7.23–7.15 (m, 2H), 5.18 (s, 1H), 4.92 (s, 1H), 3.97 (t, J = 6.4 Hz, 2H), 2.47 (t, J = 7.6 Hz, 2H), 2.36 (s, 3H), 1.75–1.64 (m, 2H).

^{13}C NMR (100 MHz, $CDCl_3$) δ 146.5, 144.8, 140.3, 133.0, 129.8, 128.4, 127.8, 127.5, 126.0, 113.3, 69.9, 30.9, 27.2, 21.6.

HRMS (ESI) *m/z*: Calcd for $C_{18}H_{21}O_3S^+$ $[M + H]^+$: 317.1206; found: 317.1204.

4-(4-Fluorophenyl)pent-4-en-1-yl 4-methylbenzenesulfonate (2u)

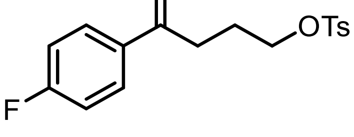

2u was prepared following the general procedure **B** with 4-fluorobenzeneboronic acid. The crude residue was purified by column chromatography (silica gel, petroleum

ether/ethyl acetate = 5:1) to afford product **2u** (917 mg, 60% over two steps) as colorless oil.

^{1}H NMR (400 MHz, $CDCl_3$) δ 7.76 (d, J = 8.4 Hz, 2H), 7.32 (d, J = 8.0 Hz, 2H), 7.28–7.24 (m, 2H), 6.97 (t, J = 8.8 Hz, 2H), 5.18 (s, 1H), 4.96 (s, 1H), 4.02 (t, J = 6.4 Hz, 2H), 2.50 (t, J = 7.6 Hz, 2H), 2.44 (s, 3H), 1.80–1.70 (m, 2H).

^{13}C NMR (100 MHz, $CDCl_3$) δ 162.5 (d, J = 246.5 Hz), 145.7, 144.9, 136.6 (d, J = 3.0 Hz), 133.2, 130.0, 128.0, 127.8 (d, J = 7.9 Hz), 115.3 (d, J = 21.3 Hz), 113.4, 69.9, 31.3, 27.3, 21.8.

^{19}F NMR (376 MHz, $CDCl_3$) δ −114.90 (s).

HRMS (ESI) ***m/z***: Calcd for $C_{18}H_{20}FO_3S^+$ [M + H]$^+$: 355.1112; found: 355.1110.

Methyl 4-(5-(tosyloxy)pent-1-en-2-yl)benzoate (2v):

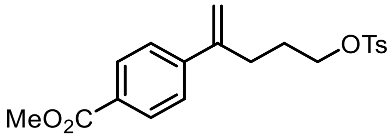

2v was prepared following the general procedure **B** with 4-methoxycarbonylphenylboronic acid. The crude residue was purified by column chromatography (silica gel, petroleum ether/ethyl acetate = 5:1) to afford product **2v** (1.38 g, 74% over two steps) as colorless oil.

^{1}H NMR (400 MHz, $CDCl_3$) δ 7.95 (d, J = 8.0 Hz, 2H), 7.76 (d, J = 7.6 Hz, 2H), 7.36 (d, J = 8.0 Hz, 2H), 7.31 (d, J = 7.6 Hz, 2H), 5.33 (s, 1H), 5.08 (s, 1H), 4.03 (t, J = 5.6 Hz, 2H), 3.89 (s, 3H), 2.55 (t, J = 6.8 Hz, 2H), 2.42 (s, 3H), 1.81–1.70 (m, 2H).

^{13}C NMR (100 MHz, $CDCl_3$) δ 166.7, 145.7, 144.9, 144.8, 133.0, 129.8, 129.7, 129.1, 127.8, 126.0, 115.1, 69.7, 52.0, 30.7, 27.1, 21.6.

HRMS (ESI) ***m/z***: Calcd for $C_{20}H_{23}O_5S^+$ [M + H]$^+$: 375.1261; found: 375.1258.

5-Phenylhex-5-en-1-yl 4-methylbenzenesulfonate (2w)

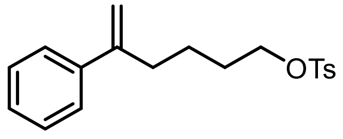

2w was prepared following the general procedure **B** with 4-methoxycarbonylphenylboronic acid. The crude residue was purified by column chromatography (silica gel, petroleum ether/ethyl acetate = 5:1) to afford product **2w** (1.46 g, 88% over two steps) as colorless oil.

^{1}H NMR (400 MHz, $CDCl_3$) δ 7.75 (d, J = 8.4 Hz, 2H), 7.34–7.23 (m, 7H), 5.25–4.95 (m, 2H), 3.99 (t, J = 6.4 Hz, 2H), 2.41 (s, 5H), 1.69–1.61 (m, 2H), 1.48–1.38 (m, 2H).

^{13}C NMR (100 MHz, $CDCl_3$) δ 147.72, 144.77, 140.91, 133.13, 129.88, 128.37, 127.90, 127.49, 126.10, 112.78, 77.48, 77.16, 76.84, 70.44, 34.51, 28.33, 23.88, 21.66.

HRMS (ESI) m/z: Calcd for $C_{19}H_{23}O_3S^+$ [M + H]$^+$: 331.1362; found: 331.1360.

General procedure C for the synthesis of 2y–2ad

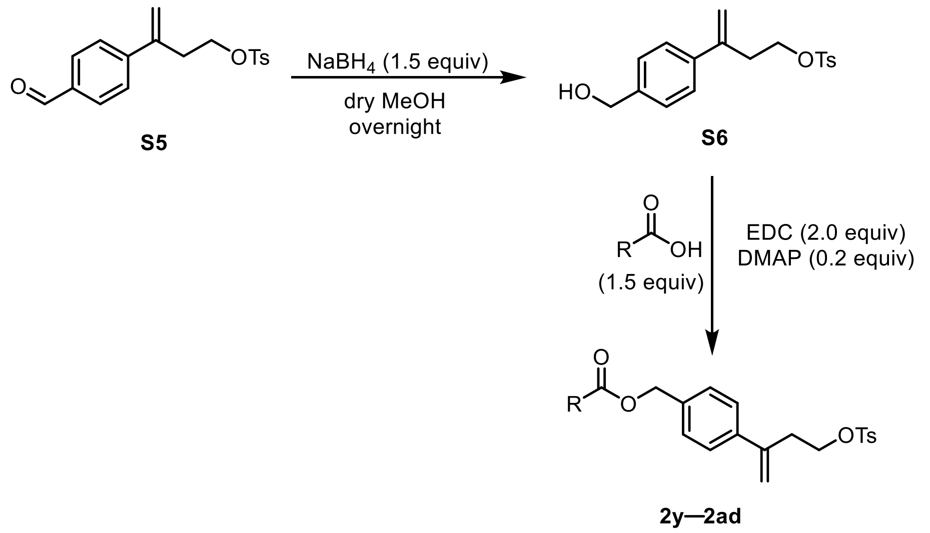

To a flask equipped with a magnetic stir bar was added **S5** (1.28 g, 3.87 mmol, 1.0 equiv), dry MeOH (20 mL) were then added with vigorous stirring, followed by $NaBH_4$ (219 mg, 5.8 mmol, 1.5 equiv.). The reaction mixture was stirred vigorously for overnight, then partitioned between EtOAc (25 mL) and H_2O (30 mL). The phases were separated and the aqueous phase was extracted into EtOAc (2 × 25 mL). The combined organic phases were washed with brine (30 mL), dried ($MgSO_4$), filtered, and concentrated in vacuo. The crude residue was purified by column chromatography (silica gel, petroleum ether/ethyl acetate = 2:1) to afford the product **S6** (1.16g, 3.49 mmol, 90%) as yellow liquid.

Added **S6** (1.5 equiv.) to a solution of acid (1.5 equiv.), DMAP (0.2 equiv.) and EDC (2.0 equiv.) in dry DCM (0.5 M) at 0 °C. Warm the reaction mixture to rt and stir for 17 h. The crude residue was purified by column chromatography (silica gel, petroleum ether/ethyl acetate = 10:1~5:1) to afford the product **2y–2ad**. All recorded spectroscopic data matched those previously reported in the literature.[2]

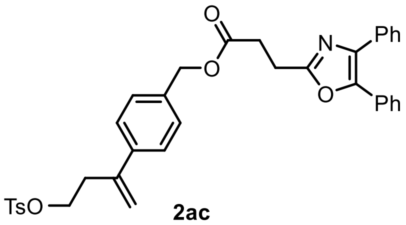

2ac

^{1}H NMR (400 MHz, $CDCl_3$) δ 7.72 (d, J = 8.4 Hz, 2H), 7.64–7.60 (m, 2H), 7.57–7.53 (m, 2H), 7.36–7.30 (m, 6H), 7.29–7.24 (m, 4H), 7.20–7.17 (m, 2H), 5.31 (s, 1H), 5.15 (s, 2H), 5.07 (s, 1H), 4.08 (t, J = 7.2 Hz, 2H), 3.21 (t, J = 7.2 Hz, 2H), 2.98 (t, J = 7.2 Hz, 2H), 2.81 (t, J = 7.2 Hz, 2H), 2.42 (s, 3H).

^{13}C NMR (100 MHz, $CDCl_3$) δ 171.9, 161.7, 145.5, 144.8, 142.3, 139.7, 135.4, 135.2, 133.1, 132.5, 129.9, 129.0, 128.7, 128.63, 128.57, 128.3, 128.2, 128.0, 127.9, 126.6, 126.2, 115.7, 68.6, 66.2, 34.7, 31.2, 23.6, 21.7.

HRMS (ESI) ***m/z***: Calcd for $C_{36}H_{34}O_6S^+$ $[M + H]^+$: 608.2101; found: 608.2100.

General procedure D for the synthesis of 2x[3]

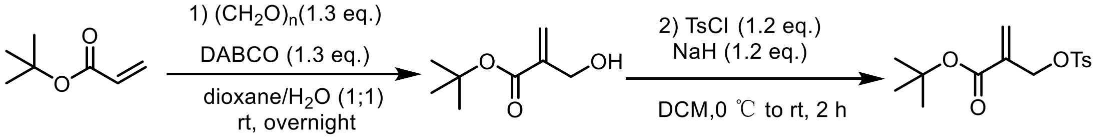

S7 **S8** **2x**

To a flask equipped with a magnetic stir bar was added $(CH_2O)n$ (390 mg, 13.0 mmol, 1.3 equiv.), DABCO (1.59 g, 13.0 mmol, 1.3 equiv.) and dioxane/H_2O (50 mL/50 mL) were then added with vigorous stirring, followed by *tert*-butyl acrylate (1.28 g, 10.0 mmol, 1.0 equiv.). The reaction mixture was stirred vigorously for overnight, then partitioned between EtOAc (60 mL) and H_2O (40 mL). The phases were separated and the aqueous phase was extracted into EtOAc (2 × 60 mL). The combined

organic phases were washed with brine (50 mL), dried ($MgSO_4$), filtered, and concentrated in vacuo. The crude residue was purified by column chromatography (silica gel, petroleum ether/ethyl acetate = 5:1) to afford product **S8** (1.39 g, 8.7 mmol, 87%). Then to a stirred solution of 1.39 g (8.8 mmol) of ester **S8** and 2.06 g (10.6 mmol) of *p*-toluenesulfonyl chloride in 100 mL of CH_2Cl_2 under N_2 at 0 °C was added 422 mg (10.6 mmol) of NaH (60% dispersion in mineral oil). After the mixture had been allowed to warm to room temperature for 2 h, 75 mL of water was added. The resulting mixture was transferred to a separatory funnel, and the layers were separated. The aqueous layer was extracted with CH_2Cl_2 (2 × 150 mL). The combined organic layers were washed with water (1 × 75 mL) and brine (1 × 75 mL), dried over $MgSO_4$, and filtered. The solvent was removed in vacuo, and the crude residue was purified by column chromatography (silica gel, petroleum ether/ethyl acetate = 20:1) to afford product **2x** (1.78 g, 65%).

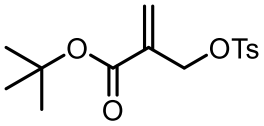

2x

^{1}H NMR (400 MHz, $CDCl_3$) δ 7.80 (d, J = 8.4 Hz, 2H), 7.35 (d, J = 8.0 Hz, 2H), 6.27 (d, J = 0.4 Hz, 1H), 5.84 (d, J = 0.8 Hz, 1H), 4.71 (s, 2H), 2.45 (s, 3H), 1.46 (s, 9H).

^{13}C NMR (100 MHz, $CDCl_3$) δ 163.7, 145.0, 135.1, 133.1, 130.0, 128.0, 127.7, 81.9, 68.0, 28.0, 21.7.

HRMS (ESI) *m/z*: Calcd for $C_{15}H_{21}O_5S^+$ $[M + H]^+$: 313.1104; found: 313.1103.

2.3 General procedure for the synthesis of product (3 as example)

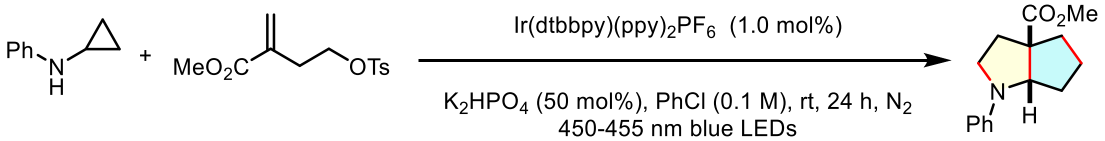

1a **2a** **3**

In the glove box, a 10 mL Schlenk tube equipped with a magnetic stir bar was charged with *N*-phenyl cyclopropylamine **1a** (53.2 mg, 0.4 mmol), **2a** (56.8 mg, 0.2 mmol), Ir(dtbbpy)(ppy)$_2$PF$_6$ (1.8 mg, 0.002 mmol, 1 mol%), K_2HPO_4 (17.4 mg, 0.1 mmol, 50 mol%) and PhCl (2.0 mL). The reaction mixture was stirred under 2×3 W blue LEDs (λ = 450−455 nm) at room temperature with stirring for 24 h. After completion of the reaction, the solvent was removed under reduced pressure, and the residue was purified with silica gel chromatography (petroleum ether/ethyl acetate = 100:1, V/V) to give the product **3**.

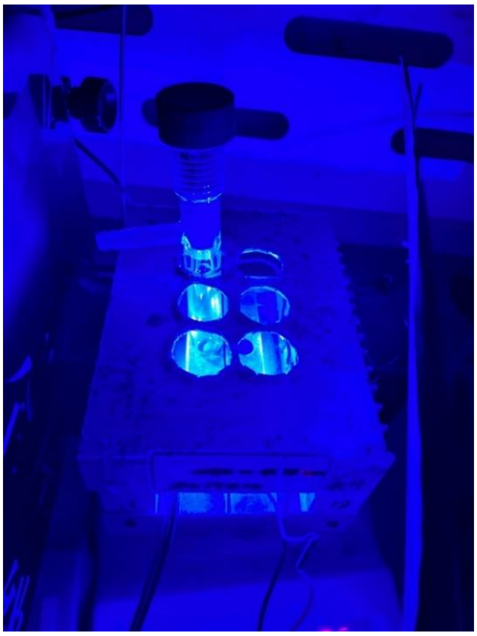	Manufacturer: GeAo Chemical Company<br>Model: 2×3 W, blue LEDs<br>Broadband source: λ = 450–455 nm<br>Material of the irradiation vessel:<br>Borosilicate reaction tube<br>Distance from the light source to the<br>irradiation vessel: 3.0 cm<br>No any filters

Figure S1. Photoreactor used in this research (2×3 W blue LEDs)

3. Optimization reaction conditions

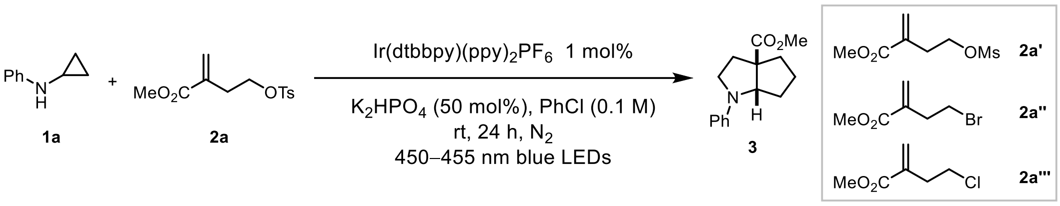

Entry	Solvent	Molar Ratio of **1a**:**2a**	PC	Additive	Yield of **3** (%)[b]
1	DMA	1:2	PC1	–	40
2	DMA	1:2	PC1	K_2HPO_4	57
3	DMA	1:2	PC1	NaH_2PO_4	50
4	DMA	1:2	PC1	$NaHCO_3$	51
5	DMA	1:2	PC1	CsF	46
6	DMA	1:2	PC1	Cs_2CO_3	32
7	DMA	1:2	PC1	DABCO	46
8	DMA	1:2	PC1	Et_3N	39
9	DMA	1:1	PC1	K_2HPO_4	53
10	DMA	1.5:1	PC1	K_2HPO_4	61
11	DMA	2:1	PC1	K_2HPO_4	66
12	DMA	3:1	PC1	K_2HPO_4	36
13	DMF	2:1	PC1	K_2HPO_4	48
**14**	**PhCl**	**2:1**	**PC1**	**K_2HPO_4**	**81**
15	PhF	2:1	PC1	K_2HPO_4	80
16	$PhCF_3$	2:1	PC1	K_2HPO_4	61
17	ethyl benzene	2:1	PC1	K_2HPO_4	69
18	toluene	2:1	PC1	K_2HPO_4	63
19	trimethylbenzene	2:1	PC1	K_2HPO_4	70
20	$MeNO_2$	2:1	PC1	K_2HPO_4	10%
21	DCM	2:1	PC1	K_2HPO_4	28%
22	DCE	2:1	PC1	K_2HPO_4	25%
23	DMSO	2:1	PC1	K_2HPO_4	34%
24	PhCl	2:1	PC2	K_2HPO_4	69
25	PhCl	2:1	PC3	K_2HPO_4	18
26	PhCl	2:1	PC4	K_2HPO_4	51
27	PhCl	2:1	PC1	–	71
28[c]	PhCl	2:1	PC1	K_2HPO_4	79
29[d]	PhCl	2:1	PC1	K_2HPO_4	67

30[e]	PhCl	2:1	PC1	K_2HPO_4	0
31[f]	PhCl	2:1	PC1	K_2HPO_4	0
32[g]	PhCl	2:1	PC1	K_2HPO_4	0
33[h]	PhCl	2:1	PC1	K_2HPO_4	53
34[i]	PhCl	2:1	PC1	K_2HPO_4	68
35[j]	PhCl	2:1	PC1	K_2HPO_4	66

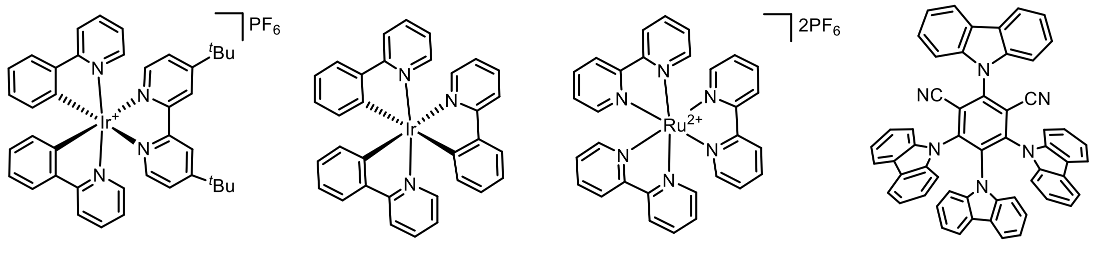

PC1 [Ir(ppy)$_2$(dtbbpy)]PF$_6$ PC2 *fac*-Ir(ppy)$_3$ PC3 [Ru(bpy)$_3$][2PF$_6$] PC4 4CzIPN

[a]Reaction conditions:**1a** (x mmol), **2a** (y mmol), PC (1.0 mol%), DMA (1.0 mL, 0.1M), rt, 24 h, N_2, 450−455nm. [b]Isolated yield. [c]100 mol% K_2HPO_4. [d]200 mol% K_2HPO_4. [e]No light. [f]No PC. [g]Under air. [h]**2a**' instead of **2a**. [i]**2a**'' instead of **2a**. [j]**2a**''' instead of **2a**.

4. Characterization data of products

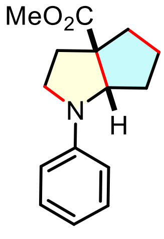

3

Methyl 1-phenylhexahydrocyclopenta[*b*]pyrrole-3a(1*H*)-carboxylate

39.6 mg, 81 % yield. Yellow oil (Flash column chromatography eluent, petroleum ether/ethyl acetate = 100/1, V/V).

^{1}H NMR (400 MHz, $CDCl_3$) δ 7.22 (t, J = 7.6 Hz, 1H), 6.71 (dd, J = 16.4, 8.4 Hz, 1H), 6.61 (d, J = 8.0 Hz, 1H), 4.13 (dd, J = 6.8, 2.0 Hz, 1H), 3.62 (s, 3H), 3.47–3.42 (m, 1H), 3.27–3.15 (m, 1H), 2.48–2.40 (m, 1H), 2.10–1.99 (m, 1H), 1.94–1.82 (m, 2H), 1.79–1.62 (m, 4H).

^{13}C NMR (100 MHz, $CDCl_3$) δ 177.2, 147.5, 129.2, 116.5, 113.1, 68.7, 60.0, 52.4, 48.8, 37.0, 34.5, 33.4, 25.1.

HRMS (ESI) *m/z*: Calcd for $C_{15}H_{20}NO_2^+$ $[M + H]^+$: 246.1489; found: 246.1488.

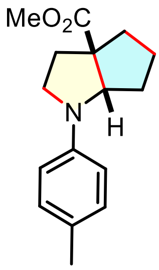

4

Methyl 1-(*p*-tolyl)hexahydrocyclopenta[*b*]pyrrole-3a(1*H*)-carboxylate

33.6 mg, 65 % yield. Yellow oil (Flash column chromatography eluent, petroleum ether/ethyl acetate = 100/1, V/V).

^{1}H NMR (400 MHz, $CDCl_3$) δ 7.06 (d, J = 8.4 Hz, 2H), 6.56 (d, J = 8.4 Hz, 2H), 4.21–4.14 (m, 1H), 3.69 (s, 3H), 3.56–3.47 (m, 1H), 3.30–3.21 (m, 1H), 2.58–2.47 (m, 1H), 2.27 (s, 3H), 2.16–2.08 (m, 1H), 1.99–1.89 (m, 2H), 1.86–1.70 (m, 4H).

^{13}C NMR (100 MHz, $CDCl_3$) δ 177.3, 145.6, 129.7, 125.7, 113.2, 69.0, 60.0, 52.3, 49.1, 37.1, 34.6, 33.5, 25.1, 20.4.

HRMS (ESI) m/z: Calcd for $C_{16}H_{22}NO_2^+$ $[M + H]^+$: 260.1645; found: 260.1642.

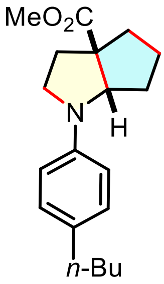

5

Methyl 1-(4-butylphenyl)hexahydrocyclopenta[*b*]pyrrole-3a(1*H*)-carboxylate

56 mg, 93 % yield. Yellow oil (Flash column chromatography eluent, petroleum ether/ethyl acetate = 100/1, V/V).

¹H NMR (400 MHz, $CDCl_3$) δ 7.08 (d, J = 8.4 Hz, 2H), 6.58 (d, J = 8.4 Hz, 2H), 4.21–4.17 (m, 1H), 3.70 (s, 3H), 3.56–3.51 (m, 1H), 3.30–3.24 (m, 1H), 2.56–2.51 (m, 3H), 2.17–2.08 (m, 1H), 2.00–1.91 (m, 2H), 1.90–1.73 (m, 4H), 1.61–1.56 (m, 2H), 1.40–1.34 (m, 2H), 0.94 (t, J = 7.2 Hz, 3H).

¹³C NMR (100 MHz, $CDCl_3$) δ 177.2, 145.7, 130.9, 129.1, 113.0, 68.9, 60.0, 52.3, 49.0, 37.1, 34.7, 34.5, 34.2, 33.5, 25.1, 22.5, 14.1.

HRMS (ESI) *m/z*: Calcd for $C_{19}H_{28}NO_2^+$ $[M + H]^+$: 302.2115; found: 302.2112.

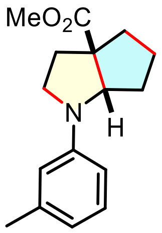

6

Methyl 1-(*m*-tolyl)hexahydrocyclopenta[*b*]pyrrole-3a(1*H*)-carboxylate

46.1 mg, 89 % yield. Colorless oil (Flash column chromatography eluent, petroleum ether/ethyl acetate = 100/1, V/V).

¹H NMR (400 MHz, $CDCl_3$) δ 7.17–7.07 (m, 1H), 6.55 (d, J = 7.6 Hz, 1H), 6.49 – 6.40 (m, 2H), 4.27 – 4.16 (m, 1H), 3.70 (s, 3H), 3.57–3.49 (m, 1H), 3.34–3.25 (m, 1H), 2.57–2.46 (m, 1H), 2.33 (s, 3H), 2.18–2.08 (m, 1H), 2.00–1.90 (m, 2H), 1.89–1.70 (m, 4H).

¹³C NMR (100 MHz, $CDCl_3$) δ 177.2, 147.5, 138.9, 129.1, 117.5, 113.8, 110.3, 68.7, 60.0, 52.3, 48.8, 37.0, 34.5, 33.5, 25.1, 22.0.

HRMS (ESI) *m/z*: Calcd for $C_{16}H_{22}NO_2^+$ $[M + H]^+$: 260.1645; found: 260.1642.

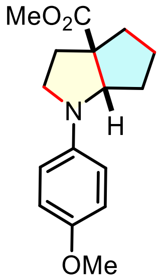

7

Methyl 1-(4-methoxyphenyl)hexahydrocyclopenta[*b*]pyrrole-3a(1*H*)-carboxylate

24.8 mg, 45 % yield. Yellow oil (Flash column chromatography eluent, petroleum ether/ethyl acetate = 50/1, V/V).

^{1}H NMR (400 MHz, $CDCl_3$) δ 6.86–6.83 (m, 2H), 6.61–6.57 (m, 2H), 4.13–4.09 (m, 1H), 3.76 (s, 3H), 3.69 (s, 3H), 3.53–3.44 (m, 1H), 3.23–3.14 (m, 1H), 2.56–2.46 (m, 1H), 2.14–2.04 (m, 1H), 1.98–1.86 (m, 2H), 1.83–1.70 (m, 4H).

^{13}C NMR (100 MHz, $CDCl_3$) δ 177.3, 151.6, 142.6, 115.0, 114.1, 69.4, 60.1, 56.0, 52.3, 49.5, 37.2, 34.8, 33.5, 25.1.

HRMS (ESI) ***m/z***: Calcd for $C_{16}H_{22}NO_3^+$ $[M + H]^+$: 276.1594; found: 276.1591.

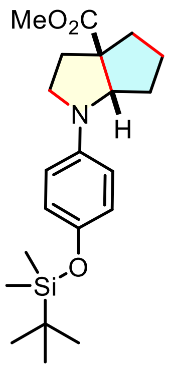

8

Methyl 1-(4-((*tert*-butyldimethylsilyl)oxy)phenyl)hexahydrocyclopenta[*b*]pyrrole -3a(1*H*)-carboxylate

34.6 mg, 46 % yield. Yellow oil (Flash column chromatography eluent, petroleum ether/ethyl acetate = 100/1, V/V).

^{1}H NMR (400 MHz, $CDCl_3$) δ 6.74 (d, J = 8.8 Hz, 2H), 6.50 (d, J = 8.8 Hz, 2H), 4.11 (d, J = 5.6 Hz, 1H), 3.69 (s, 3H), 3.51–3.44 (m, 1H), 3.24–3.13 (m, 1H), 2.56–2.46 (m, 1H), 2.14–2.04 (m, 1H), 1.95–1.86 (m, 2H), 1.84–1.71 (m, 4H), 0.97 (s, 9H), 0.15 (s, 6H).

^{13}C NMR (100 MHz, $CDCl_3$) δ 177.4, 146.9, 142.7, 120.6, 114.0, 69.3, 60.1, 52.3, 49.4, 37.3, 34.7, 33.5, 25.9, 25.1, 18.3, –4.32.

HRMS (ESI) ***m/z***: Calcd for $C_{21}H_{34}NO_3Si^+$ $[M + H]^+$: 376.2302; found: 376.2306.

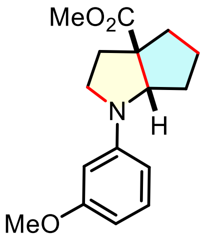

9

Methyl 1-(3-methoxyphenyl)hexahydrocyclopenta[*b*]pyrrole-3a(1*H*)-carboxylate

33 mg, 67 % yield. Yellow oil (Flash column chromatography eluent, petroleum ether/ethyl acetate = 50/1, V/V).

^{1}H NMR (400 MHz, $CDCl_3$) δ 7.16–7.09 (m, 1H), 6.32–6.22 (m, 2H), 6.18–6.14 (m, 1H), 4.21–4.19 (m, 1H), 3.80 (s, 3H), 3.69 (s, 3H), 3.54–3.47 (m, 1H), 3.34–3.25 (m, 1H), 2.55–2.47 (m, 1H), 2.17–2.06 (m, 1H), 2.00–1.91 (m, 2H), 1.88–1.70 (m, 4H).

^{13}C NMR (100 MHz, $CDCl_3$) δ 177.1, 160.7, 148.8, 129.9, 106.3, 101.4, 99.5, 68.7, 60.0, 55.2, 52.4, 48.9, 37.0, 34.4, 33.4, 25.1.

HRMS (ESI) m/z: Calcd for $C_{16}H_{22}NO_3^+$ $[M + H]^+$: 276.1594; found: 276.1592.

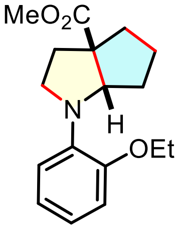

10

Methyl 1-(*m*-tolyl)hexahydrocyclopenta[*b*]pyrrole-3a(1*H*)-carboxylate

41.6 mg, 72 % yield. Yellow oil (Flash column chromatography eluent, petroleum ether/ethyl acetate = 100/1, V/V).

^{1}H NMR (400 MHz, $CDCl_3$) δ 6.89–6.82 (m, 3H), 6.76 (d, J = 7.2 Hz, 1H), 4.83–4.78 (m, 1H), 4.10–4.00 (m, 2H), 3.73 (s, 3H), 3.57–3.50 (m, 1H), 3.21–3.15 (m, 1H), 2.51–2.42 (m, 1H), 2.27–2.19 (m, 1H), 1.94–1.88 (m, 1H), 1.71–1.61 (m, 3H), 1.56–1.47 (m, 2H), 1.45 (t, J = 7.2 Hz, 3H).

^{13}C NMR (100 MHz, $CDCl_3$) δ 177.7, 150.0, 137.7, 121.2, 120.2, 117.5, 113.0, 69.0, 64.0, 59.0, 52.2, 49.6, 38.1, 35.1, 32.0, 25.9, 15.1.

HRMS (ESI) ***m/z***: Calcd for $C_{17}H_{24}NO_3^+$ $[M + H]^+$: 290.1751; found: 290.1747.

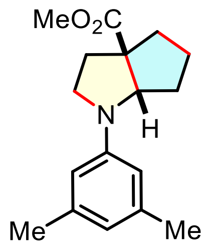

11

Methyl-(3,5-dimethylphenyl)hexahydrocyclopenta[*b*]pyrrole-3a(1*H*)-carboxylate

29 mg, 53 % yield. Yellow oil (Flash column chromatography eluent, petroleum ether/ethyl acetate = 100/1, V/V).

^{1}H NMR (400 MHz, $CDCl_3$) δ 6.40 (s, 1H), 6.27 (s, 2H), 4.25–4.13 (m, 1H), 3.69 (s, 3H), 3.56–3.47 (m, 1H), 3.34–3.22 (m, 1H), 2.56–2.46 (m, 1H), 2.29 (s, 6H), 2.17–2.07 (m, 1H), 2.01–1.91 (m, 2H), 1.89–1.68 (m, 4H).

^{13}C NMR (100 MHz, $CDCl_3$) δ 177.2, 147.6, 138.8, 118.5, 111.0, 68.7, 59.9, 52.3, 48.9, 37.0, 34.4, 33.6, 25.1, 21.9.

HRMS (ESI) ***m/z***: Calcd for $C_{17}H_{24}NO_2^+$ $[M + H]^+$: 274.1802; found: 274.1799.

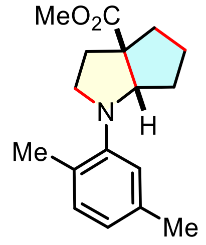

12

Methyl-1-(2,5-dimethylphenyl)hexahydrocyclopenta[*b*]pyrrole-3a(1*H*)-carboxylate

38.2 mg, 70 % yield. Yellow oil (Flash column chromatography eluent, petroleum ether/ethyl acetate = 100/1, V/V).

^{1}H NMR (400 MHz, $CDCl_3$) δ 7.02 (d, J = 7.6 Hz, 1H), 6.81 (s, 1H), 6.74 (d, J = 7.6 Hz, 1H), 4.37–4.32 (m, 1H), 3.76 (s, 3H), 3.44–3.35 (m, 1H), 2.99–2.90 (m, 1H), 2.50–

2.42 (m, 1H), 2.30 (s, 3H), 2.24 (s, 3H), 2.21–2.15 (m, 1H), 1.91–1.84 (m, 1H), 1.78–1.71 (m, 1H), 1.71–1.62 (m, 2H), 1.57–1.46 (m, 2H).

^{13}C NMR (100 MHz, $CDCl_3$) δ 178.0, 146.9, 135.8, 131.2, 128.6, 122.7, 120.0, 69.5, 59.2, 52.3, 51.7, 38.3, 36.1, 31.8, 25.7, 21.4, 19.3.

HRMS (ESI) m/z: Calcd for $C_{17}H_{24}NO_2^+$ $[M + H]^+$: 274.1802; found: 274.1799.

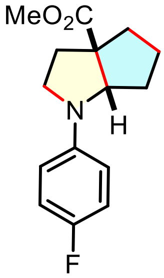

13

Methyl 1-(4-fluorophenyl)hexahydrocyclopenta[*b*]pyrrole-3a(1*H*)-carboxylate

26.6 mg, 52 % yield. Yellow oil (Flash column chromatography eluent, petroleum ether/ethyl acetate = 100/1, V/V).

^{1}H NMR (400 MHz, $CDCl_3$) δ 6.99–6.88 (m, 2H), 6.61–6.48 (m, 2H), 4.19–4.07 (m, 1H), 3.70 (s, 3H), 3.53–3.41 (m, 1H), 3.29–3.17 (m, 1H), 2.58–2.46 (m, 1H), 2.16–2.03 (m, 1H), 2.01–1.88 (m, 2H), 1.87–1.70 (m, 4H).

^{13}C NMR (100 MHz, $CDCl_3$) δ 177.1, 156.7, 154.3, 144.3, 115.7, 115.5, 113.72, 113.65, 69.1, 60.1, 52.4, 49.3, 37.1, 34.6, 33.3, 25.1.

^{19}F NMR (376 MHz, $CDCl_3$) δ –129.58.

HRMS (ESI) *m/z*: Calcd for $C_{15}H_{19}FNO_2^+$ $[M + H]^+$: 264.1394; found: 264.1391.

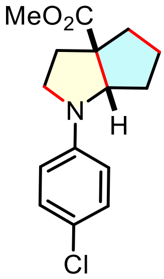

14

Methyl 1-(4-chlorophenyl)hexahydrocyclopenta[*b*]pyrrole-3a(1*H*)-carboxylate

48 mg, 85 % yield. Yellow oil (Flash column chromatography eluent, petroleum ether/ethyl acetate = 100/1, V/V).

^{1}H NMR (400 MHz, $CDCl_3$) δ 7.18–7.13 (m, 2H), 6.54–6.49 (m, 2H), 4.19–4.15 (m, 1H), 3.70 (s, 3H), 3.48–3.38 (m, 1H), 3.26 (q, J = 8.0 Hz, 1H), 2.55–2.46 (m, 1H), 2.16–2.07 (m, 1H), 2.00–1.91 (m, 2H), 1.83–1.69 (m, 4H).

^{13}C NMR (100 MHz, $CDCl_3$) δ 177.0, 146.0, 128.9, 121.3, 114.1, 68.7, 60.1, 52.4, 48.9, 36.9, 34.5, 33.1, 25.1.

HRMS (ESI) *m/z*: Calcd for $C_{15}H_{19}ClNO_2^+$ $[M + H]^+$: 280.1099; found: 280.1096.

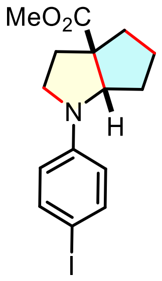

15

Methyl 1-(4-iodophenyl)hexahydrocyclopenta[*b*]pyrrole-3a(1*H*)-carboxylate

52 mg, 70 % yield. Yellow oil (Flash column chromatography eluent, petroleum ether/ethyl acetate = 100/1, V/V).

^{1}H NMR (400 MHz, $CDCl_3$) δ 7.49–7.38 (m, 2H), 6.45–6.29 (m, 2H), 4.23–4.12 (m, 1H), 3.69 (s, 3H), 3.50–3.40 (m, 1H), 3.31–3.20 (m, 1H), 2.57–2.45 (m, 1H), 2.18–2.06 (m, 1H), 2.01–1.88 (m, 2H), 1.82–1.68 (m, 4H).

^{13}C NMR (100 MHz, $CDCl_3$) δ 176.9, 146.8, 137.7, 115.3, 77.4, 68.5, 60.1, 52.4, 48.7, 36.9, 34.4, 33.1, 25.1.

HRMS (ESI) *m/z*: Calcd for $C_{15}H_{19}INO_2^+$ $[M + H]^+$: 372.0455; found: 372.0450.

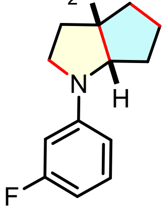

16

Methyl 1-(3-fluorophenyl)hexahydrocyclopenta[*b*]pyrrole-3a(1*H*)-carboxylate

32.8 mg, 62 % yield. Yellow oil (Flash column chromatography eluent, petroleum ether/ethyl acetate = 100/1, V/V).

^{1}H NMR (400 MHz, $CDCl_3$) δ 7.19–7.09 (m, 1H), 6.42–6.33 (m, 2H), 6.31–6.25 (m, 1H), 4.23–4.16 (m, 1H), 3.70 (s, 3H), 3.52–3.45 (m, 1H), 3.33–3.26 (m, 1H), 2.55–2.45 (m, 1H), 2.18–2.05 (m, 1H), 2.01–1.89 (m, 2H), 1.87–1.68 (m, 4H).

^{13}C NMR (100 MHz, $CDCl_3$) δ 176.9, 164.1 (d, J = 242.0 Hz), 149.0 (d, J = 10.8 Hz), 130.2 (d, J = 10.3 Hz), 108.7 (d, J = 2.2 Hz), 102.9 (d, J = 21.6 Hz), 110.0 (d, J = 25.7 Hz), 68.6, 60.1, 52.4, 48.8, 36.9, 34.4, 33.2, 25.1.

^{19}F NMR (376 MHz, $CDCl_3$) δ –112.73 (s).

HRMS (ESI) *m/z*: Calcd for $C_{15}H_{19}FNO_2^+$ [M + H] $^+$: 264.1394; found: 264.1391.

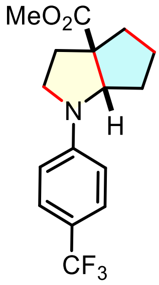

17

Methyl 1-(4-(trifluoromethyl)phenyl)hexahydrocyclopenta[*b*]pyrrole-3a(1*H*)-carboxylate

42 mg, 67 % yield. Yellow oil (Flash column chromatography eluent, petroleum ether/ethyl acetate = 100/1, V/V).

^{1}H NMR (400 MHz, $CDCl_3$) δ 7.44 (d, J = 8.8 Hz, 2H), 6.59 (d, J = 8.8 Hz, 2H), δ 4.29–4.26 (m, 1H), 3.71 (s, 3H), 3.56–3.49 (m, 1H), 3.41–3.33 (m, 1H), 2.57–2.49 (m, 1H), 2.18–2.10 (m, 1H), 2.05–1.96 (m, 2H), 1.85–1.68 (m, 4H).

^{13}C NMR (100 MHz, $CDCl_3$) δ 176.8, 149.2, 125.3 (q, J = 270.0 Hz), 126.4 (q, J = 3.8 Hz), 117.8 (q, J = 32.7 Hz), 112.2, 68.3, 60.1, 52.5, 48.6, 36.8, 34.3, 33.0, 25.1.

^{19}F NMR (376 MHz, $CDCl_3$) δ –60.79 (s).

HRMS (ESI) *m/z*: Calcd for $C_{16}H_{19}F_3NO_2^+$ [M + H]$^+$: 314.1362; found: 314.1357.

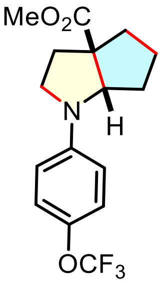

18

Methyl 1-(4-(trifluoromethoxy)phenyl)hexahydrocyclopenta[*b*]pyrrole-3a(1*H*)-carboxylate

53.4 mg, 81 % yield. Yellow oil (Flash column chromatography eluent, petroleum ether/ethyl acetate = 100/1, V/V).

^{1}H NMR (400 MHz, $CDCl_3$) δ 7.08 (d, J = 8.8 Hz, 2H), 6.54 (d, J = 9.2 Hz, 2H), 4.21 – 4.17 (m, 1H), 3.70 (s, 3H), 3.54–3.47 (m, 1H), 3.33–3.24 (m, 1H), 2.55–2.47 (m, 1H), 2.17–2.07 (m, 1H), 2.02–1.92 (m, 2H), 1.86–1.70 (m, 4H).

^{13}C NMR (100 MHz, $CDCl_3$) δ 177.0, 146.2, 140 (q, J = 3.8, 1.9 Hz), 122.3, 113.2, 68.8, 60.2, 52.4, 49.0, 36.9, 34.5, 33.2, 25.1.

^{19}F NMR (376 MHz, $CDCl_3$) δ –58.46 (s).

HRMS (ESI) *m/z*: Calcd for $C_{16}H_{19}F_3NO_3^+$ $[M + H]^+$: 330.1312; found: 330.1308.

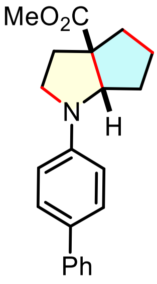

19

Methyl 1-([1,1'-biphenyl]-4-yl)hexahydrocyclopenta[*b*]pyrrole-3a(1*H*)-carboxylate

46.2 mg, 72 % yield. Yellow oil (Flash column chromatography eluent, petroleum ether/ethyl acetate = 100/1, V/V).

^{1}H NMR (400 MHz, $CDCl_3$) δ 7.57–7.52 (m, 2H), 7.51–7.46 (m, 2H), 7.38 (t, J = 7.6 Hz, 2H), 7.27–7.22 (m, 1H), 6.71–6.64 (m, 2H), 4.28–4.22 (m, 1H), 3.69 (s, 3H), 3.59–

3.52 (m, 1H), 3.37–3.30 (m, 1H), 2.56–2.48 (m, 1H), 2.17–2.08 (m, 1H), 2.02–1.93 (m, 2H), 1.91–1.71 (m, 4H).

^{13}C NMR (100 MHz, $CDCl_3$) δ 177.1, 146.8, 141.4, 129.3, 128.8, 127.8, 126.4, 126.1, 113.3, 68.7, 60.1, 52.4, 48.8, 37.0, 34.5, 33.4, 25.1.

HRMS (ESI) *m/z*: Calcd for $C_{21}H_{24}NO_2^+$ [M + H] $^+$: 322.1802; found: 322.1801.

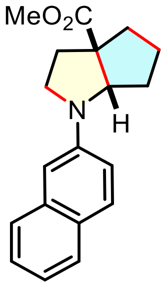

20

Methyl 1-(naphthalen-2-yl)hexahydrocyclopenta[*b*]pyrrole-3a(1*H*)-carboxylate

32.4 mg, 55 % yield. Yellow oil (Flash column chromatography eluent, petroleum ether/ethyl acetate = 100/1, V/V).

^{1}H NMR (400 MHz, $CDCl_3$) δ 7.75–7.68 (m, 2H), 7.66 (d, J = 8.4 Hz, 1H), 7.38 (t, J = 7.2 Hz, 1H), 7.21 (t, J = 7.6 Hz, 1H), 7.11–7.00 (m, 1H), 6.83 (d, J = 2.0 Hz, 1H), 4.46–4.36 (m, 1H), 3.73 (s, 3H), 3.68–3.61 (m, 1H), 3.49–3.40 (m, 1H), 2.64–2.53 (m, 1H), 2.23–2.14 (m, 1H), 2.10–2.00 (m, 2H), 1.94–1.73 (m, 4H).

^{13}C NMR (100 MHz, $CDCl_3$) δ 177.1, 145.2, 135.1, 128.8, 127.6, 126.8, 126.3, 126.1, 121.9, 116.6, 106.7, 68.7, 60.0, 52.4, 48.9, 37.1, 34.6, 33.5, 25.2.

HRMS (ESI) *m/z*: Calcd for $C_{19}H_{22}NO_2^+$ [M + H]$^+$: 296.1645; found: 296.1643.

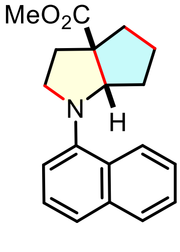

21

Methyl 1-(naphthalen-1-yl)hexahydrocyclopenta[*b*]pyrrole-3a(1*H*)-carboxylate

20.8 mg, 35 % yield. Yellow oil (Flash column chromatography eluent, petroleum ether/ethyl acetate = 200/1, V/V).

^{1}H NMR (400 MHz, $CDCl_3$) δ 8.23–8.19 (m, 1H), 7.83–7.80 (m, 1H), 7.53 (d, J = 8.4 Hz, 1H), 7.48–7.45 (m, 2H), 7.39 (t, J = 8.0 Hz, 1H), 7.14 (d, J = 7.2 Hz, 1H), 4.53–4.49 (m, 1H), 3.79 (s, 3H), 3.69–3.63 (m, 1H), 3.04–2.97 (m, 1H), 2.62–2.55 (m, 1H), 2.24–2.16 (m, 1H), 2.04–1.97 (m, 1H), 1.88–1.80 (m, 2H), 1.71–1.65 (m, 1H), 1.55–1.49 (m, 2H).

^{13}C NMR (100 MHz, $CDCl_3$) δ 178.0, 145.6, 134.9, 130.0, 128.3, 125.9, 125.8, 125.2, 124.3, 123.1, 115.7, 70.6, 59.2, 53.7, 52.4, 38.5, 36.7, 31.5, 25.7.

HRMS (ESI) *m/z*: Calcd for $C_{19}H_{22}NO_2^+$ $[M + H]^+$: 296.1645; found: 296.1642.

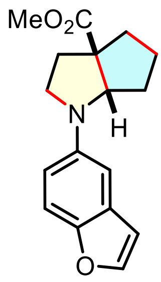

22

Methyl 1-(benzofuran-5-yl)hexahydrocyclopenta[*b*]pyrrole-3a(1*H*)-carboxylate

48.5 mg, 85 % yield. Yellow oil (Flash column chromatography eluent, petroleum ether/ethyl acetate = 100/1, V/V).

^{1}H NMR (400 MHz, $CDCl_3$) δ 7.54 (d, J = 2.0 Hz, 1H), 7.37 (d, J = 8.8 Hz, 1H), 6.76 (d, J = 2.4 Hz, 1H), 6.71–6.64 (m, 2H), 4.25–4.20 (m, 1H), 3.70 (s, 3H), 3.60–3.53 (m, 1H), 3.33–3.23 (m, 1H), 2.59–2.50 (m, 1H), 2.17–2.09 (m, 1H), 2.02–1.93 (m, 2H), 1.88–1.72 (m, 4H).

^{13}C NMR (100 MHz, $CDCl_3$) δ 177.3, 148.5, 145.3, 144.3, 128.3, 111.7, 111.5, 106.5, 103.7, 69.5, 60.0, 52.3, 49.7, 37.2, 34.7, 33.5, 25.1.

HRMS (ESI) *m/z*: Calcd for $C_{17}H_{20}NO_3^+$ $[M + H]^+$: 286.1438; found: 286.1435.

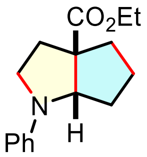

23

Ethyl 1-phenylhexahydrocyclopenta[*b*]pyrrole-3a(1*H*)-carboxylate

38.9 mg, 75 % yield. Yellow oil (Flash column chromatography eluent, petroleum ether/ethyl acetate = 100/1, V/V).

^{1}H NMR (400 MHz, $CDCl_3$) δ 7.27 – 7.21 (m, 2H), 6.70 (t, *J* = 7.2 Hz, 1H), 6.61 (d, *J* = 8.0 Hz, 2H), 4.27–4.18 (m, 1H), 4.14 (q, *J* = 7.2 Hz, 2H), 3.57–3.45 (m, 1H), 3.33–3.21 (m, 1H), 2.57–2.44 (m, 1H), 2.18–2.06 (m, 1H), 2.01–1.90 (m, 2H), 1.85–1.64 (m, 4H), 1.24 (t, *J* = 7.2 Hz, 3H).

^{13}C NMR δ 176.7, 147.5, 129.2, 116.4, 113.1, 68.6, 61.0, 60.1, 48.8, 37.1, 34.5, 33.4, 25.2, 14.3.

HRMS (ESI) *m/z*: Calcd for $C_{16}H_{22}NO_2^+$ [M + H]$^+$: 260.1645; found: 260.1643.

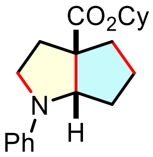

24

Cyclohexyl 1-phenylhexahydrocyclopenta[*b*]pyrrole-3a(1*H*)-carboxylate

52 mg, 83% yield. Yellow oil (Flash column chromatography eluent, petroleum ether/ethyl acetate = 100/1, V/V).

^{1}H NMR (400 MHz, $CDCl_3$) δ 7.22 (t, *J* = 8.0 Hz, 2H), 6.70 (t, *J* = 7.2 Hz, 1H), 6.61 (d, *J* = 8.0 Hz, 2H), 4.82–4.73 (m, 1H), 4.22–4.16 (m, 1H), 3.54–3.48 (m, 1H), 3.33–3.26 (m, 1H), 2.54–2.45 (m, 1H), 2.16–2.08 (m, 1H), 1.99–1.91 (m, 2H), 1.85–1.75 (m, 5H), 1.72–1.58 (m, 4H), 1.47–1.30 (m, 5H).

^{13}C NMR (100 MHz, $CDCl_3$) δ 176.1, 147.5, 129.2, 116.3, 113.0, 72.7, 68.6, 60.2, 48.8, 37.1, 34.5, 33.4, 31.45, 31.40, 25.5, 25.2, 23.6, 23.5.

HRMS (ESI) *m/z*: Calcd for $C_{20}H_{28}NO_2^+$ [M + H] $^+$: 314.2115; found: 314.2110.

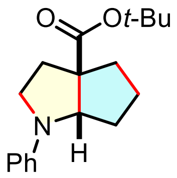

25

***tert*-Butyl 1-phenylhexahydrocyclopenta[*b*]pyrrole-3a(1*H*)-carboxylate**

37 mg, 64% yield. Yellow oil (Flash column chromatography eluent, petroleum ether/ethyl acetate = 100/1, V/V).

^{1}H NMR (400 MHz, $CDCl_3$) δ 7.26–7.20 (m, 2H), 6.70 (t, J = 7.2 Hz, 1H), 6.61 (d, J = 8.0 Hz, 2H), 4.23–4.17 (m, 1H), 4.08 (t, J = 6.8 Hz, 2H), 3.54–3.47 (m, 1H), 3.32–3.23 (m, 1H), 2.55–2.46 (m, 1H), 2.16–2.07 (m, 1H), 2.00–1.91 (m, 2H), 1.86–1.69 (m, 4H), 1.61–1.56 (m, 2H), 1.39–1.32 (m, 2H), 0.91 (t, J = 7.6 Hz, 3H).

^{13}C NMR (100 MHz, $CDCl_3$) δ 176.7, 147.5, 129.2, 116.4, 113.0, 68.7, 64.9, 60.1, 48.8, 37.0, 34.4, 33.4, 30.8, 25.2, 19.3, 13.8.

HRMS (ESI) ***m/z***: Calcd for $C_{18}H_{26}NO_2^+$ [M + H]$^+$: 288.1958; found:288.1954.

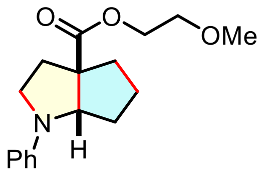

26

2-Methoxyethyl 1-phenylhexahydrocyclopenta[*b*]pyrrole-3a(1*H*)-carboxylate

46.9 mg, 81% yield. Yellow oil (Flash column chromatography eluent, petroleum ether/ethyl acetate = 100/1, V/V).

^{1}H NMR (400 MHz, $CDCl_3$) δ 7.22 (t, J = 8.4, 7.5 Hz, 2H), 6.70 (t, J = 7.2 Hz, 1H), 6.61 (d, J = 8.0 Hz, 2H), 4.28–4.17 (m, 3H), 3.60–3.54 (m, 2H), 3.54–3.48 (m, 1H), 3.33 (s, 3H), 3.31–3.26 (m, 1H), 2.58–2.47 (m, 1H), 2.19–2.09 (m, 1H), 2.02–1.91 (m, 2H), 1.87–1.69 (m, 4H).

^{13}C NMR (100 MHz, $CDCl_3$) δ 176.6, 147.4, 129.2, 116.4, 113.0, 70.5, 68.6, 63.9, 60.1, 59.0, 48.8, 36.9, 34.4, 33.3, 25.1.

HRMS (ESI) ***m/z***: Calcd for $C_{17}H_{24}NO_3^+$ [M + H]$^+$: 290.1751; found: 290.1747.

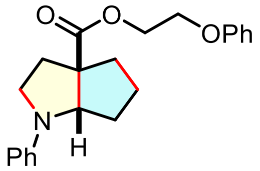

27

2-Phenoxyethyl 1-phenylhexahydrocyclopenta[*b*]pyrrole-3a(1*H*)-carboxylate

52 mg, 74% yield. Yellow oil (Flash column chromatography eluent, petroleum ether/ethyl acetate = 100/1, V/V).

^{1}H NMR (400 MHz, $CDCl_3$) δ 7.29–7.18 (m, 4H), 6.95 (t, J = 7.2 Hz, 1H), 6.86 (d, J = 8.0 Hz, 2H), 6.70 (t, J = 7.2 Hz, 1H), 6.59 (d, J = 8.0 Hz, 2H), 4.47–4.39 (m, 2H), 4.24–4.19 (m, 1H), 4.15 (t, J = 4.8 Hz, 2H), 3.53–3.45 (m, 1H), 3.33–3.25 (m, 1H), 2.56–2.46 (m, 1H), 2.17–2.08 (m, 1H), 1.98–1.88 (m, 2H), 1.83–1.66 (m, 4H).

^{13}C NMR (100 MHz, $CDCl_3$) δ 176.6, 158.6, 147.4, 129.6, 129.2, 121.3, 116.5, 114.8, 113.1, 68.7, 65.9, 63.3, 60.1, 48.8, 36.9, 34.4, 33.4, 25.1.

HRMS (ESI) ***m/z***: Calcd for $C_{22}H_{26}NO_3^+$ $[M + H]^+$: 352.1907; found: 352.1906.

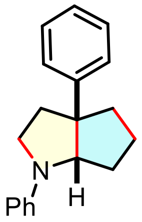

28

1,3a-Diphenyloctahydrocyclopenta[*b*]pyrrole

38.5 mg, 73 % yield. Colorless oil (Flash column chromatography eluent, petroleum ether/ethyl acetate = 100/1, V/V).

^{1}H NMR (400 MHz, $CDCl_3$) δ 7.30–7.23 (m, 4H), 7.22–7.13 (m, 3H), 6.71 (t, J = 7.2 Hz, 1H), 6.64 (d, J = 8.0 Hz, 2H), 4.20 (d, J = 7.6 Hz, 1H), 3.55–3.48 (m, 1H), 3.33–3.24 (m, 1H), 2.31–2.20 (m, 2H), 2.08–2.00 (m, 2H), 1.96–1.80 (m, 4H).

^{13}C NMR (100 MHz, $CDCl_3$) δ 149.1, 147.2, 129.3, 128.5, 126.1, 126.0, 115.9, 112.6, 69.9, 58.6, 47.6, 38.8, 38.6, 33.2, 24.2.

HRMS (ESI) ***m/z***: Calcd for $C_{19}H_{22}N^{+}$ $[M + H]^{+}$: 264.1747; found: 264.1745.

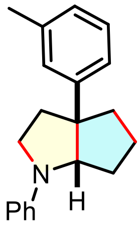

29

1-Phenyl-3a-(*m*-tolyl)octahydrocyclopenta[*b*]pyrrole

38.8 mg, 70% yield. Colorless oil (Flash column chromatography eluent, petroleum ether/ethyl acetate = 100/1, V/V).

^{1}H NMR (400 MHz, $CDCl_3$) δ 7.28–7.24 (m, 2H), 7.16 (t, J = 7.6 Hz, 1H), 7.06 (s, 1H), 7.02–6.97 (m, 2H), 6.71 (t, J = 7.2 Hz, 1H), 6.64 (d, J = 8.0 Hz, 2H), 4.22–4.18 (m, 1H), 3.54–3.48 (m, 1H), 3.35–3.26 (m, 1H), 2.32 (s, 3H), 2.27–2.15 (m, 2H), 2.09–1.99 (m, 2H), 1.93–1.76 (m, 4H).

^{13}C NMR (100 MHz, $CDCl_3$) δ 149.1, 147.2, 138.0, 129.3, 128.3, 126.83, 126.79, 123.2, 115.8, 112.6, 69.8, 58.5, 47.7, 38.9, 38.8, 33.2, 24.3, 21.8.

HRMS (ESI) ***m/z***: Calcd for $C_{20}H_{24}N^{+}$ $[M + H]^{+}$: 278.1903; found: 278.1902.

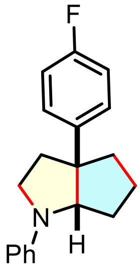

30

3a-(4-Fluorophenyl)-1-phenyloctahydrocyclopenta[*b*]pyrrole

50.1 mg, 89% yield. Colorless oil (Flash column chromatography eluent, petroleum ether/ethyl acetate = 100/1, V/V).

^{1}H NMR (400 MHz, $CDCl_3$) δ 7.29–7.24 (m, 2H), 7.18–7.13 (m, 2H), 6.95 (t, J = 12.0, 5.4 Hz, 2H), 6.72 (t, J = 7.2 Hz, 1H), 6.64 (d, J = 8.0 Hz, 2H), 4.15 (d, J = 8.8 Hz, 1H),

3.55–3.49 (m, 1H), 3.30–3.23 (m, 1H), 2.31–2.19 (m, 2H), 2.05–1.96 (m, 2H), 1.91–1.79 (m, 4H).

^{13}C NMR (100 MHz, $CDCl_3$) δ 162.4 (d, J = 244.2 Hz), 147.1, 144.8 (d, J = 3.2 Hz), 129.3, 127.6 (d, J = 7.5 Hz), 116.0, 115.2 (d, J = 20.9 Hz), 112.6, 70.0, 58.1, 47.5, 38.8, 38.7, 33.1, 24.1.

^{19}F NMR (376 MHz, $CDCl_3$) δ –116.77 ~ –119.14 (m).

HRMS (ESI) *m/z*: Calcd for $C_{19}H_{21}N^+$ $[M + H]^+$: 282.1653; found: 282.1652.

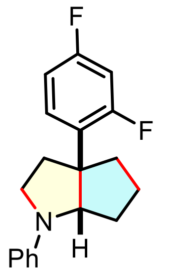

31

3a-(2,4-Difluorophenyl)-1-phenyloctahydrocyclopenta[*b*]pyrrole

34.1 mg, 57% yield. Yellow oil (Flash column chromatography eluent, petroleum ether/ethyl acetate = 100/1, V/V).

^{1}H NMR (400 MHz, $CDCl_3$) δ 7.29–7.22 (m, 2H), 7.08–7.00 (m, 1H), 6.82–6.76 (m, 1H), 6.75–6.67 (m, 2H), 6.62 (d, J = 8.0 Hz, 2H), 4.32–4.23 (m, 1H), 3.55–3.46 (m, 1H), 3.21–3.12 (m, 1H), 2.40–2.25 (m, 2H), 2.19–2.05 (m, 2H), 1.95–1.76 (m, 4H).

^{13}C NMR (100 MHz, $CDCl_3$) δ 161.6 (dd, J = 247.3, 12.4 Hz), 161.1 (dd, J = 249.7, 11.4 Hz), 147.1, 130.4 (dd, J = 13.2, 3.9 Hz), 129.3, 128.3 (dd, J = 9.2, 7.0 Hz), 116.0, 112.4, 110.5 (dd, J = 20.4, 3.5 Hz), 104.8 (dd, J = 27.3, 25.0 Hz), 68.6, 55.9 (d, J = 2.7 Hz), 47.6, 37.8 (d, J = 4.8 Hz), 36.8, 32.4, 24.3.

^{19}F NMR (376 MHz, $CDCl_3$) δ –106.04 ~ –106.16 (m), –113.57 ~ –113.69 (m).

HRMS (ESI) *m/z*: Calcd for $C_{19}H_{20}F_2N^+$ $[M + H]^+$: 300.1558; found: 300.1555.

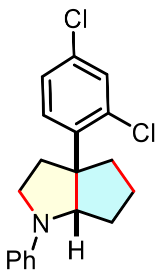

32

3a-(2,4-Dichlorophenyl)-1-phenyloctahydrocyclopenta[*b*]pyrrole

29.9 mg, 45% yield. Yellow oil (Flash column chromatography eluent, petroleum ether/ethyl acetate = 100/1, V/V).

^{1}H NMR (400 MHz, $CDCl_3$) δ 7.40 (d, J = 2.4 Hz, 1H), 7.28–7.24 (m, 2H), 7.08 (dd, J = 8.4, 2.4 Hz, 1H), 7.00 (d, J = 8.8 Hz, 1H), 6.70 (t, J = 7.2 Hz, 1H), 6.62 (d, J = 8.0 Hz, 2H), 4.41–4.36 (m, 1H), 3.49–3.43 (m, 1H), 3.13–3.06 (m, 1H), 2.57–2.48 (m, 1H), 2.45–2.33 (m, 2H), 2.27–2.18 (m, 1H), 2.05–1.98 (m, 1H), 1.95–1.86 (m, 1H), 1.77–1.68 (m, 2H).

^{13}C NMR (100 MHz, $CDCl_3$) δ 146.8, 142.6, 134.6, 132.7, 131.3, 129.4, 128.8, 126.8, 115.9, 112.1, 68.5, 58.2, 47.6, 37.1, 36.3, 31.9, 24.5.

HRMS (ESI) *m/z*: Calcd for $C_{19}H_{20}Cl_2N^+$ $[M + H]^+$: 332.0967; found: 332.0963.

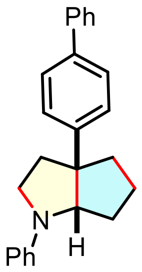

33

3a-([1,1'-Biphenyl]-4-yl)-1-phenyloctahydrocyclopenta[*b*]pyrrole

53 mg, 78% yield. White solid, melting point: 138.1–138.9 ºC (Flash column chromatography eluent, petroleum ether/ethyl acetate = 100/1, V/V).

^{1}H NMR (400 MHz, $CDCl_3$) δ 7.55 (d, J = 7.6 Hz, 2H), 7.50 (d, J = 8.0 Hz, 2H), 7.41 (t, J = 7.6 Hz, 2H), 7.35–7.30 (m, 1H), 7.27 (t, J = 7.2 Hz, 4H), 6.72 (t, J = 7.2 Hz, 1H),

6.66 (d, J = 8.0 Hz, 2H), 4.23 (d, J = 7.6 Hz, 1H), 3.62–3.48 (m, 1H), 3.36–3.25 (m, 1H), 2.35–2.21 (m, 2H), 2.14–1.95 (m, 3H), 1.91–1.76 (m, 3H).

^{13}C NMR (100 MHz, $CDCl_3$) δ 148.2, 147.2, 140.9, 139.0, 129.3, 128.9, 127.24, 127.15, 127.1, 126.5, 116.0, 112.6, 70.0, 58.4, 47.7, 38.8, 38.7, 33.2, 24.2.

HRMS (ESI) *m/z*: Calcd for $C_{25}H_{26}N^+$ $[M + H]^+$: 340.2060; found: 340.2056.

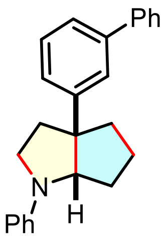

34

3a-([1,1'-Biphenyl]-3-yl)-1-phenyloctahydrocyclopenta[*b*]pyrrole

46.2 mg, 68% yield. Yellow oil (Flash column chromatography eluent, petroleum ether/ethyl acetate = 100/1, V/V).

^{1}H NMR (400 MHz, $CDCl_3$) δ 7.54 (d, J = 7.6 Hz, 2H), 7.47 (s, 1H), 7.44–7.38 (m, 3H), 7.35–7.30 (m, 2H), 7.28–7.22 (m, 2H), 7.16 (d, J = 7.6 Hz, 1H), 6.71 (t, J = 7.2 Hz, 1H), 6.65 (d, J = 8.0 Hz, 2H), 4.31–4.23 (m, 1H), 3.57–3.49 (m, 1H), 3.38–3.29 (m, 1H), 2.36–2.28 (m, 1H), 2.28–2.22 (m, 1H), 2.16–2.10 (m, 1H), 2.08–1.96 (m, 2H), 1.92–1.80 (m, 3H).

^{13}C NMR (100 MHz, $CDCl_3$) δ 149.5, 147.2, 141.6, 141.5, 129.3, 128.9, 128.8, 127.4, 125.1, 125.04, 125.00, 116.0, 112.7, 70.0, 58.7, 47.7, 38.9, 38.8, 33.2, 24.3.

HRMS (ESI) *m/z*: Calcd for $C_{25}H_{26}N^+$ $[M + H]^+$: 340.2060; found: 340.2056.

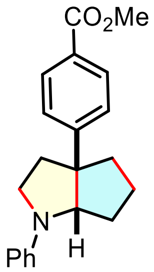

35

Methyl 4-(1-phenylhexahydrocyclopenta[*b*]pyrrol-3a(1*H*)-yl)benzoate

56.7 mg, 88% yield. White solid, melting point: 109.9–111.1 ºC (Flash column chromatography eluent, petroleum ether/ethyl acetate = 100/1, V/V).

^{1}H NMR (400 MHz, $CDCl_3$) δ 7.94 (d, J = 7.6 Hz, 2H), 7.27 (d, J = 6.4 Hz, 4H), 6.80–6.70 (m, 1H), 6.67 (t, J = 14.8 Hz, 2H), 4.29–4.13 (m, 1H), 3.89 (s, 3H), 3.61–3.45 (m, 1H), 3.36–3.17 (m, 1H), 2.36–2.18 (m, 2H), 2.09–1.97 (m, 2H), 1.95–1.65 (m, 4H).

^{13}C NMR (100 MHz, $CDCl_3$) δ 167.1, 154.3, 147.0, 129.8, 129.3, 128.0, 126.1, 116.2, 112.7, 69.7, 58.8, 52.1, 47.6, 38.7, 38.6, 33.1, 24.1.

HRMS (ESI) ***m/z***: Calcd for $C_{21}H_{24}NO_2^+$ [M + H]$^+$: 322.1802; found: 322.1800.

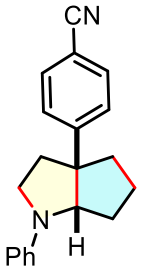

36

4-(1-Phenylhexahydrocyclopenta[*b*]pyrrol-3a(1*H*)-yl)benzonitrile

35.1 mg, 61% yield. White solid, melting point: 99.9–101.1 ºC (Flash column chromatography eluent, petroleum ether/ethyl acetate = 100/1, V/V).

^{1}H NMR (400 MHz, $CDCl_3$) δ 7.56 (d, J = 8.4 Hz, 2H), 7.32–7.23 (m, 4H), 6.74 (t, J = 7.2 Hz, 1H), 6.64 (d, J = 8.0 Hz, 2H), 4.18 (d, J = 7.6 Hz, 1H), 3.61–3.49 (m, 1H), 3.35–3.22 (m, 1H), 2.35–2.20 (m, 2H), 2.08–1.98 (m, 2H), 1.93–1.81 (m, 4H).

^{13}C NMR (100 MHz, $CDCl_3$) δ 154.5, 146.8, 132.3, 129.4, 126.9, 119.0, 116.4, 112.7, 110.0, 69.8, 58.9, 47.5, 38.6, 38.3, 33.0, 24.0.

HRMS (ESI) ***m/z***: Calcd for $C_{20}H_{21}N_2^+$ [M + H] $^+$: 289.1699; found: 289.1696.

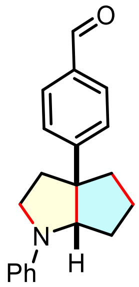

37

4-(1-Phenylhexahydrocyclopenta[*b*]pyrrol-3a(1*H*)-yl)benzaldehyde

25.1 mg, 43% yield. White solid, melting point: 101.2–104.5 ºC (Flash column chromatography eluent, petroleum ether/ethyl acetate = 100/1, V/V).

^{1}H NMR (400 MHz, $CDCl_3$) δ 9.96 (s, 1H), 7.79 (d, J = 8.4 Hz, 2H), 7.37 (d, J = 8.4 Hz, 2H), 7.27 (dd, J = 13.6, 5.2 Hz, 2H), 6.74 (t, J = 7.2 Hz, 1H), 6.65 (d, J = 8.0 Hz, 2H), 4.23 (d, J = 8.4 Hz, 1H), 3.61–3.52 (m, 1H), 3.37–3.27 (m, 1H), 2.39–2.24 (m, 2H), 2.10–2.01 (m, 2H), 1.97–1.81 (m, 4H).

^{13}C NMR (100 MHz, $CDCl_3$) δ 192.0, 156.2, 146.9, 134.6, 130.1, 129.4, 126.8, 116.3, 112.7, 69.8, 59.0, 47.6, 38.7, 38.5, 33.1, 24.1.

HRMS (ESI) *m/z*: Calcd for $C_{20}H_{22}NO^+$ $[M + H]^+$: 292.1696; found: 292.1693.

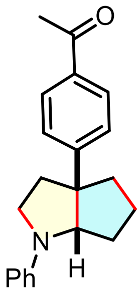

38

1-(4-(1-Phenylhexahydrocyclopenta[*b*]pyrrol-3a(1*H*)-yl)phenyl)ethan-1-one

38.4 mg, 63% yield. Yellow oil (Flash column chromatography eluent, petroleum ether/ethyl acetate = 100/1, V/V).

^{1}H NMR (400 MHz, $CDCl_3$) δ 7.96–7.70 (m, 2H), 7.33–7.09 (m, 4H), 6.82–6.69 (m, 1H), 6.71–6.56 (m, 2H), 4.31–4.12 (m, 1H), 3.63–3.47 (m, 1H), 3.34–3.18 (m, 1H), 2.56 (s, 3H), 2.35–2.19 (m, 2H), 2.10–1.98 (m, 2H), 1.97–1.75 (m, 4H).

^{13}C NMR (100 MHz, CDCl$_3$) δ 197.8, 154.6, 146.9, 135.1, 129.3, 128.6, 126.3, 116.2, 112.6, 69.7, 58.8, 47.6, 38.6, 38.5, 33.1, 26.7, 24.1.

HRMS (ESI) ***m/z***: Calcd for $C_{21}H_{24}NO^+$ [M + H]$^+$: 306.1852; found: 306.1850.

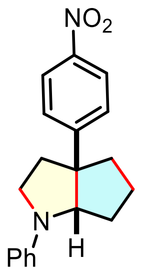

39

1-(4-(1-Phenylhexahydrocyclopenta[*b*]pyrrol-3a(1*H*)-yl)phenyl)ethan-1-one

20.3 mg, 33% yield. Yellow solid, melting point: 97.7–98.8 ºC (Flash column chromatography eluent, petroleum ether/ethyl acetate = 100/1, V/V).

^{1}H NMR (400 MHz, CDCl$_3$) δ 8.21–7.98 (m, 2H), 7.41–7.32 (m, 2H), 7.31–7.24 (m, 2H), 6.75 (t, J = 7.2 Hz, 1H), 6.65 (d, J = 8.0 Hz, 2H), 4.22 (d, J = 8.8 Hz, 1H), 3.62–3.51 (m, 1H), 3.35–3.25 (m, 1H), 2.41–2.23 (m, 2H), 2.10–1.99 (m, 2H), 1.98–1.82 (m, 4H).

^{13}C NMR (100 MHz, CDCl$_3$) δ 156.7, 146.8, 146.3, 129.4, 127.0, 123.8, 116.5, 112.7, 69.8, 58.9, 47.5, 38.7, 38.5, 33.0, 24.1.

HRMS (ESI) ***m/z***: Calcd for $C_{19}H_{21}N_2O_2^+$ [M + H]$^+$: 309.1598; found: 309.1593.

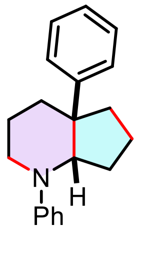

40

1,4a-Diphenyloctahydro-1*H*-cyclopenta[*b*]pyridine

25.0 mg, 45% yield. Yellow oil (Flash column chromatography eluent, petroleum ether/ethyl acetate = 100/1, V/V).

^{1}H NMR (400 MHz, $CDCl_3$) δ 7.42 (d, J = 7.6 Hz, 2H), 7.24–7.15 (m, 4H), 7.11–7.04 (m, 1H), 6.94 (d, J = 7.6 Hz, 2H), 6.71 (t, J = 7.2 Hz, 1H), 4.45 (d, J = 8.0 Hz, 1H), 3.41 (d, J = 8.4 Hz, 1H), 2.89–2.74 (m, 1H), 2.08–2.00 (m, 1H), 1.90–1.52 (m, 7H), 1.51–1.29 (m, 2H).

^{13}C NMR (100 MHz, $CDCl_3$) δ 150.7, 149.3, 129.4, 128.0, 127.4, 125.6, 117.8, 114.6, 61.7, 46.2, 41.6, 39.4, 32.8, 21.8, 21.6, 18.8.

HRMS (ESI) ***m/z***: Calcd for $C_{20}H_{24}N^+$ $[M + H]^+$: 278.1903; found: 278.1901.

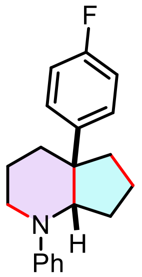

41

4a-(4-Fluorophenyl)-1-phenyloctahydro-1H-cyclopenta[*b*]pyridine

36.6 mg, 62% yield. Yellow oil (Flash column chromatography eluent, petroleum ether/ethyl acetate = 100/1, V/V).

^{1}H NMR (400 MHz, $CDCl_3$) δ 7.41–7.33 (m, 2H), 7.24–7.15 (m, 2H), 6.93 (d, J = 8.4 Hz, 2H), 6.87 (t, J = 8.4 Hz, 2H), 6.72 (t, J = 7.2 Hz, 1H), 4.40 (t, J = 8.8 Hz, 1H), 3.41 (d, J = 11.6 Hz, 1H), 2.88–2.76 (m, 1H), 2.06–1.98 (m, 1H), 1.80–1.67 (m, 4H), 1.58 (d, J = 13.2 Hz, 2H), 1.53–1.43 (m, 2H), 1.40–1.32 (m, 1H).

^{13}C NMR (100 MHz, $CDCl_3$) δ 160.9 (d, J = 243.6 Hz), 150.7, 144.9 (d, J = 3.1 Hz), 129.4, 128.9 (d, J = 7.5 Hz), 118.0, 114.7, 114.6 (d, J = 20.7 Hz), 62.0, 45.8, 41.6, 39.4, 32.8, 21.7, 21.4, 18.7.

^{19}F NMR (376 MHz, $CDCl_3$) δ –111.96 ~ –125.69 (m).

HRMS (ESI) ***m/z***: Calcd for $C_{20}H_{23}FN^+$ $[M + H]^+$: 296.1809; found: 296.1805.

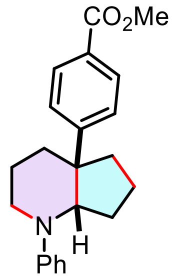

42

Methyl 4-(1-phenyloctahydro-4a*H*-cyclopenta[*b*]pyridin-4a-yl)benzoate

34.9 mg, 52% yield. White solid, melting point: 114.1–114.8 ºC (Flash column chromatography eluent, petroleum ether/ethyl acetate = 100/1, V/V).

^{1}H NMR (400 MHz, $CDCl_3$) δ 7.95 (d, J = 8.4 Hz, 2H), 7.58 (d, J = 8.4 Hz, 2H), 7.30 (t, J = 8.0 Hz, 2H), 7.03 (d, J = 8.4 Hz, 2H), 6.82 (t, J = 7.2 Hz, 1H), 4.54 (t, J = 8.8 Hz, 1H), 3.90 (s, 3H), 3.50 (d, J = 11.6 Hz, 1H), 2.98–2.84 (m, 1H), 2.21–2.09 (m, 1H), 1.94–1.66 (m, 7H), 1.58 (s, 1H), 1.46–1.34 (m, 1H).

^{13}C NMR (100 MHz, $CDCl_3$) δ 167.3, 154.8, 150.6, 129.4, 129.3, 127.5, 118.1, 114.8, 61.8, 52.1, 46.6, 41.6, 39.0, 32.7, 21.8, 21.4, 18.7.

HRMS (ESI) *m/z*: Calcd for $C_{22}H_{26}NO_2^+$ [M + H]$^+$: 336.1958; found: 336.1953.

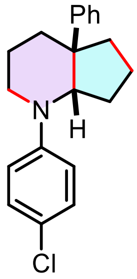

43

1-(4-Chlorophenyl)-4a-phenyloctahydro-1*H*-cyclopenta[*b*]pyridine

36.2 mg, 58% yield. Yellow oil (Flash column chromatography eluent, petroleum ether/ethyl acetate = 100/1, V/V).

^{1}H NMR (400 MHz, $CDCl_3$) δ 7.37 (d, J = 7.6 Hz, 2H), 7.20 (t, J = 7.6 Hz, 2H), 7.16–7.05 (m, 3H), 6.84 (d, J = 9.2 Hz, 2H), 4.38 (t, J = 8.8 Hz, 1H), 3.35 (d, J = 11.6 Hz,

1H), 2.86–2.73 (m, 1H), 2.09–1.97 (m, 1H), 1.82–1.55 (m, 7H), 1.47–1.43 (m, 1H), 1.42–1.32 (m, 1H).

^{13}C NMR (100 MHz, $CDCl_3$) δ 149.3, 149.0, 129.2, 128.1, 127.3, 125.7, 122.5, 115.8, 61.9, 46.3, 41.8, 39.3, 32.6, 21.7, 21.6, 18.7.

HRMS (ESI) *m/z*: Calcd for $C_{20}H_{23}ClN^+$ $[M + H]^+$: 312.1514; found: 312.1509.

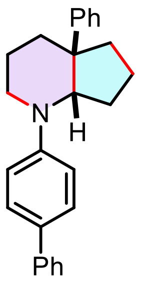

44

1-([1,1'-Biphenyl]-4-yl)-4a-phenyloctahydro-1*H*-cyclopenta[*b*]pyridine

48.8 mg, 69% yield. Yellow oil (Flash column chromatography eluent, petroleum ether/ethyl acetate = 100/1, V/V).

^{1}H NMR (400 MHz, $CDCl_3$) δ 7.65–7.57 (m, 4H), 7.53 (d, J = 7.2 Hz, 2H), 7.44 (t, J = 7.6 Hz, 2H), 7.32 (t, J = 7.6 Hz, 3H), 7.21 (t, J = 7.2 Hz, 1H), 7.12 (d, J = 8.8 Hz, 2H), 4.62 (t, J = 8.8 Hz, 1H), 3.61 (d, J = 11.2 Hz, 1H), 3.07–2.91 (m, 1H), 2.22–2.14 (m, 1H), 2.04–1.98 (m, 1H), 1.92–1.71 (m, 6H), 1.60–1.48 (m, 2H).

^{13}C NMR (100 MHz, $CDCl_3$) δ 149.9, 149.2, 141.2, 130.4, 128.8, 128.1, 128.0, 127.4, 126.5, 126.2, 125.7, 114.6, 61.5, 46.3, 41.7, 39.4, 32.8, 21.8, 21.7, 18.8.

HRMS (ESI) *m/z*: Calcd for $C_{26}H_{28}N^+$ $[M + H]^+$: 354.2216; found: 354.2212.

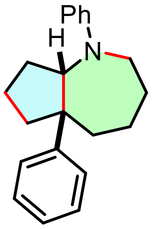

45

1,5a-Diphenyldecahydrocyclopenta[*b*]azepine

33.8 mg, 58% yield. Yellow oil (Flash column chromatography eluent, petroleum ether/ethyl acetate = 100/1, V/V).

^{1}H NMR (400 MHz, $CDCl_3$) *δ* 7.28–7.19 (m, 6H), 7.15–7.09 (m, 1H), 6.81 (d, *J* = 8.0 Hz, 2H), 6.68 (t, *J* = 7.2 Hz, 1H), 4.68 (t, *J* = 8.0 Hz, 1H), 3.58–3.48 (m, 1H), 3.44–3.35 (m, 1H), 2.16–2.03 (m, 3H), 1.99–1.84 (m, 4H), 1.83–1.77 (m, 1H), 1.74–1.61 (m, 2H), 1.54–1.45 (m, 1H), 1.45–1.36 (m, 1H).

^{13}C NMR (100 MHz, $CDCl_3$) *δ* 151.09, 151.07, 129.4, 128.2, 126.8, 125.5, 116.4, 113.4, 64.8, 53.8, 45.4, 40.1, 37.1, 28.0, 27.7, 22.1, 21.5.

HRMS (ESI) *m/z*: Calcd for $C_{21}H_{26}N^+$ [M + H]$^+$: 292.2060; found: 292.2057.

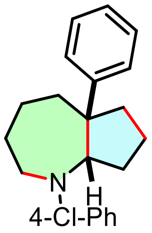

46

1-(4-Chlorophenyl)-5a-phenyldecahydrocyclopenta[*b*]azepine

29.3 mg, 45% yield. Yellow oil (Flash column chromatography eluent, petroleum ether/ethyl acetate = 100/1, V/V).

^{1}H NMR (400 MHz, $CDCl_3$) *δ* 7.23 (d, *J* = 4.0 Hz, 4H), 7.18–7.08 (m, 3H), 6.73–6.63 (m, 2H), 4.60 (t, *J* = 8.0 Hz, 1H), 3.59–3.44 (m, 1H), 3.40–3.29 (m, 1H), 2.15–2.03 (m, 3H), 1.99–1.65 (m, 7H), 1.45–1.35 (m, 1H).

^{13}C NMR (100 MHz, $CDCl_3$) δ 150.8, 149.6, 129.1, 128.2, 126.7, 125.6, 120.9, 114.6, 65.3, 53.8, 45.3, 40.3, 36.9, 27.8, 27.7, 22.1, 21.5.

HRMS (ESI) *m/z*: Calcd for $C_{21}H_{24}ClN^+$ [M + H]$^+$: 326.1670; found: 326.1668.

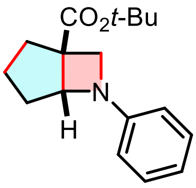

47

***tert*-Butyl 6-phenyl-6-azabicyclo[3.2.0]heptane-1-carboxylate**

38.3 mg, 70% yield. Yellow oil (Flash column chromatography eluent, petroleum ether/ethyl acetate = 200/1, V/V).

^{1}H NMR (400 MHz, $CDCl_3$) δ 7.12 (t, J = 7.6 Hz, 2H), 6.62 (t, J = 7.2 Hz, 1H), 6.35 (d, J = 8.0 Hz, 2H), 4.45 (d, J = 3.6 Hz, 1H), 4.03 (d, J = 7.2 Hz, 1H), 3.54 (d, J = 7.6 Hz, 1H), 2.01–1.91 (m, 3H), 1.84–1.75 (m, 1H), 1.64–1.41 (m, 2H), 1.37 (s, 9H).

^{13}C NMR (100 MHz, $CDCl_3$) δ 174.2, 149.5, 129.1, 116.8, 110.7, 80.9, 72.8, 57.1, 50.7, 34.9, 32.5, 28.1, 25.4.

HRMS (ESI) *m/z*: Calcd for $C_{17}H_{24}NO_2^+$ $[M + H]^+$: 274.1802; found: 274.1799.

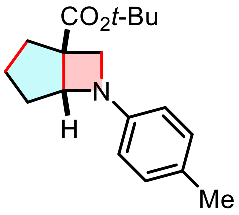

48

***tert*-Butyl 6-(*p*-tolyl)-6-azabicyclo[3.2.0]heptane-1-carboxylate**

32 mg, 56% yield. Yellow oil (Flash column chromatography eluent, petroleum ether/ethyl acetate = 200/1, V/V).

^{1}H NMR (400 MHz, $CDCl_3$) δ 7.01 (d, J = 8.0 Hz, 2H), 6.35 (d, J = 8.4 Hz, 2H), 4.48 (d, J = 4.0 Hz, 1H), 4.06 (d, J = 7.6 Hz, 1H), 3.60 (d, J = 7.6 Hz, 1H), 2.24 (s, 3H), 2.09–1.94 (m, 4H), 1.63–1.57 (m, 2H), 1.44 (s, 9H).

^{13}C NMR (100 MHz, $CDCl_3$) δ 174.4, 147.6, 129.7, 126.0, 110.9, 80.8, 73.1, 57.3, 50.7, 34.9, 32.6, 28.1, 25.4, 20.5.

HRMS (ESI) *m/z*: Calcd for $C_{18}H_{26}NO_2^+$ $[M + H]^+$: 288.1958; found:288.1952.

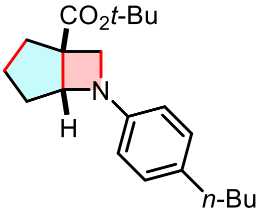

49

***tert*-Butyl 6-(4-butylphenyl)-6-azabicyclo[3.2.0]heptane-1-carboxylate**

55.0 mg, 84% yield. Yellow oil (Flash column chromatography eluent, petroleum ether/ethyl acetate = 100/1, V/V).

^{1}H NMR (400 MHz, $CDCl_3$) δ 7.04 (d, J = 8.4 Hz, 2H), 6.81 (d, J = 8.4 Hz, 2H), 6.10 (d, J = 1.2 Hz, 1H), 5.41 (d, J = 1.2 Hz, 1H), 4.19–4.13 (m, 2H), 2.60–2.56 (m, 1H), 2.55–2.50 (m, 2H), 1.61–1.56 (m, 2H), 1.53 (s, 9H), 1.38–1.33 (m, 2H), 0.93 (t, J = 7.2 Hz, 3H), 0.82–0.76 (m, 2H), 0.66–0.60 (m, 2H).

^{13}C NMR (100 MHz, $CDCl_3$) δ 166.0, 147.5, 139.2, 131.8, 128.8, 123.9, 113.5, 81.1, 53.8, 34.8, 34.1, 33.0, 28.3, 22.6, 14.1, 8.7.

HRMS (ESI) *m/z*: Calcd for $C_{21}H_{32}NO_2^+$ [M + H]$^+$: 330.2428; found: 330.2425.

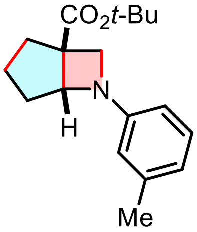

50

***tert*-Butyl 6-(*m*-tolyl)-6-azabicyclo[3.2.0]heptane-1-carboxylate**

22.9 mg, 40% yield. Yellow oil (Flash column chromatography eluent, petroleum ether/ethyl acetate = 100/1, V/V).

^{1}H NMR (400 MHz, $CDCl_3$) δ 7.13–7.06 (m, 1H), 6.53 (d, J = 7.6 Hz, 1H), 6.30–6.20 (m, 2H), 4.52 (d, J = 4.4 Hz, 1H), 4.09 (d, J = 7.6 Hz, 1H), 3.61 (d, J = 7.6 Hz, 1H), 2.29 (s, 3H), 2.11–1.95 (m, 4H), 1.91–1.82 (m, 1H), 1.62–1.57 (m, 1H), 1.44 (s, 9H).

^{13}C NMR (100 MHz, $CDCl_3$) δ 174.3, 149.6, 139.0, 129.0, 117.8, 111.4, 107.9, 80.8, 72.8, 57.1, 50.6, 34.9, 32.5, 28.1, 25.4, 21.8.

HRMS (ESI) *m/z*: Calcd for $C_{18}H_{26}NO_2^+$ [M+H] $^+$: 288.1958; found:288.1971.

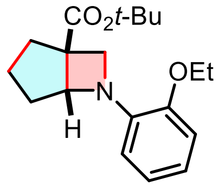

51

***tert*-Butyl 6-(*p*-tolyl)-6-azabicyclo[3.2.0]heptane-1-carboxylate**

56.4 mg, 89% yield. Yellow oil (Flash column chromatography eluent, petroleum ether/ethyl acetate = 200/1, V/V).

^{1}H NMR (400 MHz, $CDCl_3$) δ 6.87–6.81 (m, 1H), 6.76 (t, J = 6.8 Hz, 1H), 6.69 (t, J = 6.8 Hz, 1H), 6.43 (t, J = 7.2 Hz, 1H), 4.74–4.68 (m, 1H), 4.25–4.18 (m, 1H), 4.03–3.94 (m, 2H), 3.79–3.72 (m, 1H), 2.11–1.82 (m, 5H), 1.66–1.55 (m, 1H), 1.48–1.39 (m, 12H).

^{13}C NMR (100 MHz, $CDCl_3$) δ 174.7, 147.9, 138.9, 121.2, 118.1, 112.3, 112.1, 80.6, 74.0, 63.7, 58.6, 50.4, 35.3, 33.2, 28.1, 25.5, 15.0.

HRMS (ESI) *m/z*: Calcd for $C_{19}H_{28}NO_3^+$ $[M + H]^+$: 318.2064; found: 318.2062.

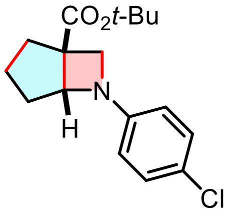

52

***tert*-Butyl 6-(4-butylphenyl)-6-azabicyclo[3.2.0]heptane-1-carboxylate**

55.0 mg, 84% yield. Yellow oil (Flash column chromatography eluent, petroleum ether/ethyl acetate = 100/1, V/V).

^{1}H NMR (400 MHz, $CDCl_3$) δ 7.04 (d, J = 8.4 Hz, 2H), 6.81 (d, J = 8.4 Hz, 2H), 6.10 (d, J = 1.2 Hz, 1H), 5.41 (d, J = 1.2 Hz, 1H), 4.19–4.13 (m, 2H), 2.60–2.56 (m, 1H), 2.55–2.50 (m, 2H), 1.61–1.56 (m, 2H), 1.53 (s, 9H), 1.38–1.33 (m, 2H), 0.93 (t, J = 7.2 Hz, 3H), 0.82–0.76 (m, 2H), 0.66–0.60 (m, 2H).

^{13}C NMR (100 MHz, $CDCl_3$) δ 166.0, 147.5, 139.2, 131.8, 128.8, 123.9, 113.5, 81.1, 53.8, 34.8, 34.1, 33.0, 28.3, 22.6, 14.1, 8.7.

HRMS (ESI) *m/z*: Calcd for $C_{21}H_{32}NO_2^+$ $[M + H]^+$: 330.2428; found: 330.2425.

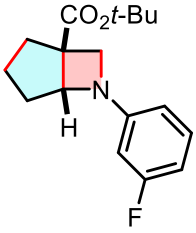

53

***tert*-Butyl 6-(3-fluorophenyl)-6-azabicyclo[3.2.0]heptane-1-carboxylate**

43 mg, 73% yield. White solid, melting point: 105.2–105.9 ºC (Flash column chromatography eluent, petroleum ether/ethyl acetate = 200/1, V/V).

^{1}H NMR (400 MHz, $CDCl_3$) δ 7.15–7.08 (m, 1H), 6.40–6.34 (m, 1H), 6.19–6.14 (m, 1H), 6.12–6.06 (m, 1H), 4.52 (d, J = 4.4 Hz, 1H), 4.10 (d, J = 7.6 Hz, 1H), 3.59 (d, J = 7.6 Hz, 1H), 2.10–1.99 (m, 4H), 1.92–1.85 (m, 1H), 1.62–1.57 (m, 1H), 1.45 (s, 9H).

^{13}C NMR (100 MHz, $CDCl_3$) δ 173.9, 164.2 (d, J = 243.2 Hz), 151.0 (d, J = 10.6 Hz), 130.3 (d, J = 10.2 Hz), 106.4 (d, J = 2.2 Hz), 103.3 (d, J = 21.7 Hz), 97.7 (d, J = 25.0 Hz), 81.1, 72.8, 57.1, 50.7, 34.6, 32.1, 28.1, 25.3.

^{19}F NMR (376 MHz, $CDCl_3$) δ –109.87 ~–117.67 (m).

HRMS (ESI) *m/z*: Calcd for $C_{17}H_{23}FNO_2^+$ $[M + H]^+$: 292.1707; found: 292.1705.

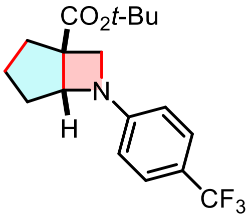

54

***tert*-Butyl 6-(4-(trifluoromethyl)phenyl)-6-azabicyclo[3.2.0]heptane-1-carboxylate**

25.3 mg, 37% yield. Yellow oil (Flash column chromatography eluent, petroleum ether/ethyl acetate = 200/1, V/V).

^{1}H NMR (400 MHz, $CDCl_3$) δ 7.47–7.37 (m, 2H), 6.45–6.35 (m, 2H), 4.59 (d, J = 3.6 Hz, 1H), 4.16 (d, J = 6.4 Hz, 1H), 3.67–3.60 (m, 1H), 2.12–1.98 (m, 4H), 1.94–1.87 (m, 1H), 1.62–1.59 (m, 1H), 1.46 (s, 9H).

^{13}C NMR (100 MHz, $CDCl_3$) δ 173.7, 151.1, 126.5 (q, J = 9.2 Hz),, 118.3 (q, J = 57.2 Hz), 109.9, 81.3, 77.5, 77.2, 76.8, 72.5, 56.9, 50.8, 34.6, 31.9, 28.1, 25.2.

^{19}F NMR (376 MHz, $CDCl_3$) δ –60.85 (s).

HRMS (ESI) *m/z*: Calcd for $C_{18}H_{23}F_3NO_2^+$ [M + H]$^+$: 342.1675; found: 342.1671.

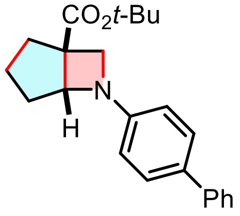

55

***tert*-Butyl 6-([1,1'-biphenyl]-4-yl)-6-azabicyclo[3.2.0]heptane-1-carboxylate**

53.1 mg, 76% yield. White solid, melting point: 112.2–112.9 ºC (Flash column chromatography eluent, petroleum ether/ethyl acetate = 100/1, V/V).

^{1}H NMR (400 MHz, $CDCl_3$) δ 7.52 (d, J = 8.0 Hz, 2H), 7.46 (d, J = 7.2 Hz, 2H), 7.38 (t, J = 7.2 Hz, 2H), 7.24 (t, J = 6.0 Hz, 1H), 6.49 (d, J = 7.2 Hz, 2H), 4.57 (d, J = 3.6 Hz, 1H), 4.15 (d, J = 7.6 Hz, 1H), 3.65 (d, J = 6.8 Hz, 1H), 2.14–1.98 (m, 4H), 1.93–1.85 (m, 1H), 1.60 (d, J = 5.2 Hz, 1H), 1.45 (d, J = 1.2 Hz, 9H).

^{13}C NMR (100 MHz, $CDCl_3$) δ 174.1, 148.8, 141.5, 129.8, 128.8, 127.9, 126.5, 126.1, 111.0, 81.0, 72.8, 57.2, 50.7, 34.8, 32.4, 28.1, 25.4.

HRMS (ESI) *m/z*: Calcd for $C_{23}H_{28}NO_2^+$ [M + H]$^+$: 350.2115; found: 350.2111.

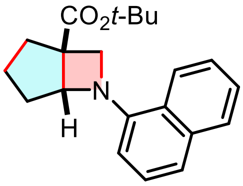

56

***tert*-Butyl 6-(naphthalen-1-yl)-6-azabicyclo[3.2.0]heptane-1-carboxylate**

47.0 mg, 72% yield. Yellow oil (Flash column chromatography eluent, petroleum ether/ethyl acetate = 100/1, V/V).

^{1}H NMR (400 MHz, $CDCl_3$) δ 7.96 (d, J = 8.4 Hz, 1H), 7.79 (d, J = 8.0 Hz, 1H), 7.44–7.29 (m, 4H), 6.56 (d, J = 7.2 Hz, 1H), 5.06 (d, J = 4.8 Hz, 1H), 4.42 (d, J = 8.0 Hz, 1H), 4.13 (d, J = 8.0 Hz, 1H), 2.07–1.93 (m, 5H), 1.72 – 1.65 (m, 1H), 1.50 (s, 9H).

^{13}C NMR (100 MHz, $CDCl_3$) δ 174.2, 145.4, 135.2, 128.6, 126.2, 125.6, 125.0, 124.0, 118.7, 107.3, 81.0, 74.2, 60.4, 50.5, 35.3, 32.3, 28.2, 25.9.

HRMS (ESI) *m/z*: Calcd for $C_{21}H_{26}NO_2^+$ $[M + H]^+$: 324.1958; found: 324.1955.

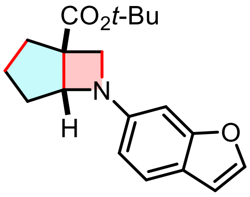

57

***tert*-Butyl 6-(benzofuran-6-yl)-6-azabicyclo[3.2.0]heptane-1-carboxylate**

53.2 mg, 85% yield. Yellow oil (Flash column chromatography eluent, petroleum ether/ethyl acetate = 100/1, V/V).

^{1}H NMR (400 MHz, $CDCl_3$) δ 7.53 (d, J = 1.6 Hz, 1H), 7.34 (d, J = 9.2 Hz, 1H), 7.05 (dd, J = 2.0, 0.4 Hz, 1H), 6.94–6.88 (m, 1H), 6.69–6.64 (m, 1H), 6.12 (d, J = 1.2 Hz, 1H), 5.45 (d, J = 1.6 Hz, 1H), 4.27–4.21 (m, 2H), 2.65–2.57 (m, 1H), 1.53 (s, 9H), 0.86–0.80 (m, 2H), 0.67–0.62 (m, 2H).

^{13}C NMR (100 MHz, $CDCl_3$) δ 166.0, 149.0, 146.1, 145.3, 139.3, 128.1, 124.0, 112.2, 111.2, 106.6, 104.6, 81.1, 54.7, 33.4, 28.3, 8.9.

HRMS (ESI) *m/z*: Calcd for $C_{19}H_{24}NO_3^+$ $[M + H]^+$: 314.1751; found: 314.1749.

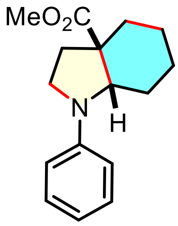

58

Methyl 1-phenyloctahydro-3a*H*-indole-3a-carboxylate

15.0 mg, 29 % yield. Yellow oil (Flash column chromatography eluent, petroleum ether/ethyl acetate = 100/1, V/V).

^{1}H NMR (400 MHz, $CDCl_3$) δ 7.19 (t, J = 8.0 Hz, 2H), 6.62 (t, J = 7.2 Hz, 1H), 6.56 (d, J = 8.0 Hz, 2H), 4.25–4.17 (m, 1H), 3.65 (s, 3H), 3.34–3.25 (m, 2H), 2.47–2.40 (m, 1H), 2.23–2.14 (m, 2H), 1.92–1.84 (m, 2H), 1.72–1.62 (m, 2H), 1.45–1.38 (m, 1H), 1.35–1.30 (m, 1H), 1.15–1.08 (m, 1H).

^{13}C NMR (100 MHz, $CDCl_3$) δ 177.2, 146.8, 129.3, 115.5, 112.2, 58.7, 53.1, 52.5, 45.2, 30.8, 28.5, 25.7, 23.1, 21.6.

HRMS (ESI) *m/z*: Calcd for $C_{16}H_{22}NO_2^+$ [M + H] $^+$: 260.1645; found: 260.1643.

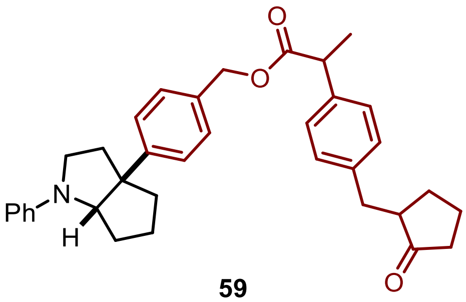

59

from Loxoprofen

4-(1-Phenylhexahydrocyclopenta[*b*]pyrrol-3a(1*H*)-yl)benzyl 2-(4-((2-oxocyclopentyl)methyl)phenyl)propanoate

98.1 mg, 94% yield. Yellow oil (Flash column chromatography eluent, petroleum ether/ethyl acetate = 5/1, V/V).

^{1}H NMR (400 MHz, $CDCl_3$) δ 7.26 (d, J = 7.6 Hz, 1H), 7.23 (d, J = 3.2 Hz, 1H), 7.20 (d, J = 8.0 Hz, 2H), 7.15 (s, 4H), 7.10 (d, J = 8.0 Hz, 2H), 6.71 (t, J = 7.2 Hz, 1H), 6.63 (d, J = 8.0 Hz, 2H), 5.10–4.99 (m, 2H), 4.17 (d, J = 7.6 Hz, 1H), 3.72 (q, J = 7.2 Hz, 1H), 3.54–3.48 (m, 1H), 3.30–3.23 (m, 1H), 3.13–3.08 (m, 1H), 2.53–2.46 (m, 1H), 2.36–2.26 (m, 3H), 2.23–2.18 (m, 1H), 2.11–1.99 (m, 4H), 1.93–1.79 (m, 5H), 1.75–1.64 (m, 2H), 1.48 (d, J = 7.2 Hz, 3H).

^{13}C NMR (100 MHz, $CDCl_3$) δ 220.2, 174.5, 148.9, 147.0, 138.9, 138.3, 133.7, 129.24, 129.17, 128.0, 127.7, 126.2, 116.0, 112.5, 69.8, 66.2, 58.4, 51.0, 47.5, 45.2, 38.7, 38.5, 38.3, 35.2, 33.1, 29.2, 24.1, 20.6, 18.6.

HRMS (ESI) ***m/z***: Calcd for $C_{35}H_{40}NO_3^+$ [M + H]$^+$: 522.3003; found: 522.2999.

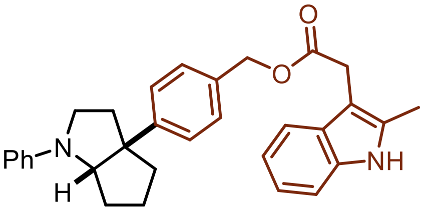

60

from 3-Indoleacetic acid

4-(1-Phenylhexahydrocyclopenta[*b*]pyrrol-3a(1*H*)-yl)benzyl 2-(2-methyl-1*H*-indol-3-yl)acetate

70 mg, 75% yield. Yellow oil (Flash column chromatography eluent, petroleum ether/ethyl acetate = 5/1, V/V).

^{1}H NMR (400 MHz, $CDCl_3$) δ 7.79 (s, 1H), 7.48 (d, J = 8.0 Hz, 1H), 7.27 (t, J = 8.0 Hz, 2H), 7.21–7.14 (m, 5H), 7.10–7.03 (m, 2H), 6.72 (t, J = 7.2 Hz, 1H), 6.64 (d, J = 8.0 Hz, 2H), 5.05 (s, 2H), 4.16 (d, J = 7.6 Hz, 1H), 3.70 (s, 2H), 3.54–3.47 (m, 1H), 3.30–3.22 (m, 1H), 2.31 (d, J = 4.8 Hz, 3H), 2.28–2.18 (m, 2H), 2.05–1.97 (m, 2H), 1.92–1.78 (m, 4H).

^{13}C NMR (100 MHz, $CDCl_3$) δ 172.0, 149.0, 147.1, 135.2, 133.7, 132.8, 129.3, 128.5, 128.3, 126.2, 121.3, 119.6, 118.2, 116.0, 112.6, 110.4, 104.5, 69.9, 66.3, 58.4, 47.6, 38.7, 38.6, 33.1, 30.5, 24.2, 11.8.

HRMS (ESI) ***m/z***: Calcd for $C_{31}H_{33}N_2O_2^+$ [M + H] $^+$: 465.2537; found: 465.2534.

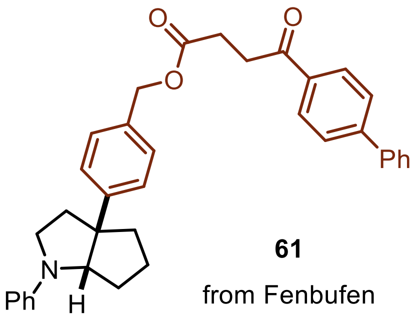

61

from Fenbufen

4-(1-Phenylhexahydrocyclopenta[*b*]pyrrol-3a(1*H*)-yl)benzyl 4-([1,1'-biphenyl]-4-yl)-4-oxobutanoate

60.4 mg, 57% yield. Yellow oil (Flash column chromatography eluent, petroleum ether/ethyl acetate = 5/1, V/V).

^{1}H NMR (400 MHz, $CDCl_3$) δ 8.03 (d, J = 8.0 Hz, 2H), 7.67 (d, J = 8.0 Hz, 2H), 7.61 (d, J = 7.6 Hz, 2H), 7.46 (t, J = 7.2 Hz, 2H), 7.41–7.33 (m, 1H), 7.28–7.17 (m, 6H), 6.71 (t, J = 7.2 Hz, 1H), 6.63 (d, J = 8.0 Hz, 2H), 5.11 (s, 2H), 4.18 (d, J = 7.2 Hz, 1H), 3.51 (t, J = 6.8 Hz, 1H), 3.34 (t, J = 6.4 Hz, 2H), 3.29–3.21 (m, 1H), 2.82 (t, J = 6.4 Hz, 2H), 2.31–2.16 (m, 2H), 2.05–1.96 (m, 2H), 1.92–1.76 (m, 4H).

^{13}C NMR (100 MHz, $CDCl_3$) δ 197.7, 172.9, 149.2, 147.1, 146.0, 139.9, 135.3, 133.6, 129.3, 129.1, 128.8, 128.5, 128.4, 127.4, 127.3, 126.3, 116.0, 112.6, 69.9, 66.4, 58.4, 47.6, 38.7, 38.6, 33.5, 33.1, 28.4, 24.1.

HRMS (ESI) *m/z*: Calcd for $C_{36}H_{36}NO_3^+$ $[M + H]^+$: 530.2690; found: 530.2690.

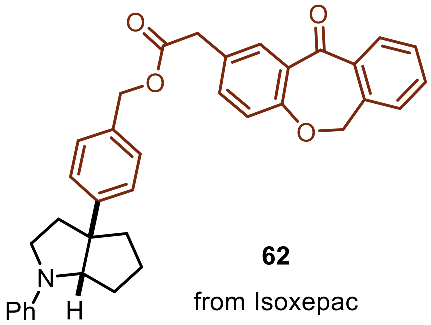

62

from Isoxepac

4-(1-Phenylhexahydrocyclopenta[*b*]pyrrol-3a(1*H*)-yl)benzyl 2-(11-oxo-6,11-dihydrodibenzo[*b*,*e*]oxepin-2-yl)acetate

69 mg, 63% yield. Yellow oil (Flash column chromatography eluent, petroleum ether/ethyl acetate = 10/1, V/V).

^{1}H NMR (400 MHz, $CDCl_3$) δ 8.11 (d, J = 2.4 Hz, 1H), 7.87 (d, J = 7.6 Hz, 1H), 7.53 (t, J = 7.6 Hz, 1H), 7.47–7.39 (m, 2H), 7.33 (d, J = 7.6 Hz, 1H), 7.27–7.22 (m, 4H), 7.19 (d, J = 8.4 Hz, 2H), 7.01 (d, J = 8.4 Hz, 1H), 6.71 (t, J = 7.2 Hz, 1H), 6.63 (d, J = 8.0 Hz, 2H), 5.15 (s, 2H), 5.09 (s, 2H), 4.18 (d, J = 8.0 Hz, 1H), 3.66 (s, 2H), 3.54–3.47 (m, 1H), 3.31–3.22 (m, 1H), 2.30–2.17 (m, 2H), 2.05–1.97 (m, 2H), 1.93–1.78 (m, 4H).

^{13}C NMR (100 MHz, $CDCl_3$) δ 190.9, 171.3, 160.6, 149.2, 147.1, 140.5, 136.5, 135.6, 133.4, 132.8, 132.6, 129.6, 129.34, 129.25, 128.5, 127.9, 127.8, 126.3, 125.2, 121.2, 116.0, 112.6, 73.7, 69.8, 66.6, 58.4, 47.6, 40.2, 38.65, 38.58, 33.1, 24.1.

HRMS (ESI) *m/z*: Calcd for $C_{36}H_{34}NO_4^+$ $[M + H]^+$: 544.2482; found: 544.2482.

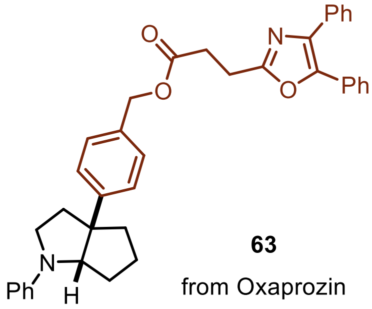

63

from Oxaprozin

4-(1-Phenylhexahydrocyclopenta[*b*]pyrrol-3a(1*H*)-yl)benzyl 3-(4,5-diphenyloxazol-2-yl)propanoate

107 mg, 94% yield. Yellow oil (Flash column chromatography eluent, petroleum ether/ethyl acetate = 5/1, V/V).

^{1}H NMR (400 MHz, $CDCl_3$) δ 7.63–7.60 (m, 2H), 7.55–7.52 (m, 2H), 7.34–7.28 (m, 6H), 7.26–7.21 (m, 4H), 7.14 (d, J = 8.4 Hz, 2H), 6.73–6.68 (m, 1H), 6.62 (d, J = 8.0 Hz, 2H), 5.11 (s, 2H), 4.15 (d, J = 8.4 Hz, 1H), 3.52–3.46 (m, 1H), 3.28–3.22 (m, 1H), 3.18 (t, J = 7.6 Hz, 2H), 2.94 (t, J = 7.6 Hz, 2H), 2.28–2.13 (m, 2H), 2.02–1.95 (m, 2H), 1.86–1.76 (m, 4H).

^{13}C NMR (100 MHz, $CDCl_3$) δ 171.9, 161.8, 149.1, 147.0, 145.5, 135.2, 133.4, 132.5, 129.3, 129.1, 128.7, 128.6, 128.5, 128.3, 128.1, 128.0, 126.5, 126.3, 116.0, 112.6, 69.8, 66.4, 58.4, 47.5, 38.64, 38.58, 33.1, 31.2, 24.1, 23.6.

HRMS (ESI) *m/z*: Calcd for $C_{38}H_{37}N_2O_3^+$ $[M + H]^+$: 569.2799; found: 569.2797.

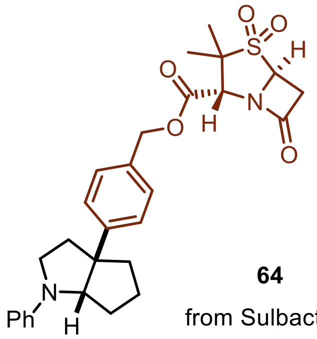

64

from Sulbactam

4-(1-Phenylhexahydrocyclopenta[*b*]pyrrol-3a(1*H*)-yl)benzyl (2*R*,5*S*)-3,3-dimethyl-7-oxo-4-thia-1-azabicyclo[3.2.0]heptane-2-carboxylate 4,4-dioxide

41 mg, 40% yield. Yellow oil (Flash column chromatography eluent, petroleum ether/ethyl acetate = 5/1, V/V).

^{1}H NMR (400 MHz, $CDCl_3$) δ 7.33–7.25 (m, 6H), 6.77 (t, J = 7.2 Hz, 1H), 6.71 (t, J = 14.8 Hz, 2H), 5.33–5.05 (m, 2H), 4.64 (d, J = 2.0 Hz, 1H), 4.44 (s, 1H), 4.23 (d, J = 7.6 Hz, 1H), 3.61–3.54 (m, 1H), 3.54–3.43 (m, 2H), 3.39–3.26 (m, 1H), 2.40–2.22 (m, 2H), 2.12–2.03 (m, 2H), 2.00–1.85 (m, 4H), 1.60 (s, 3H), 1.34 (s, 3H).

^{13}C NMR (100 MHz, $CDCl_3$) δ 170.8, 167.0, 150.1, 147.0, 132.0, 129.3, 129.0, 126.6, 116.1, 112.6, 69.9, 68.0, 63.3, 62.9, 61.2, 58.5, 47.6, 38.7, 38.6, 38.4, 33.1, 24.1, 20.3, 18.8.

HRMS (ESI) *m/z*: Calcd for $C_{28}H_{33}N_2O_5S^+$ [M + H]$^+$: 509.2105; found: 509.2100.

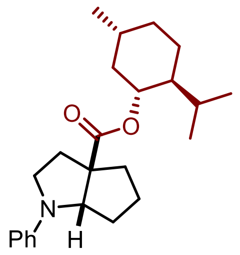

65

from (-)-Menthol

(1*R*,2*S*,5*R*)-2-*iso*-Propyl-5-methylcyclohexyl-1-phenylhexahydrocyclopenta[*b*]pyrrole-3a(1*H*)-carboxylate

60 mg, 81% yield. Yellow oil (Flash column chromatography eluent, petroleum ether/ethyl acetate = 100/1, V/V).

^{1}H NMR (400 MHz, $CDCl_3$) δ 7.26–7.20 (m, 2H), 6.73–6.67 (m, 1H), 6.64–6.55 (m, 2H), 4.71–4.58 (m, 1H), 4.25–4.14 (m, 1H), 3.56–3.46 (m, 1H), 3.34–3.23 (m, 1H), 2.51–2.42 (m, 1H), 2.15–2.06 (m, 1H), 2.01–1.91 (m, 3H), 1.85–1.64 (m, 7H), 1.51–1.35 (m, 2H), 1.08–1.00 (m, 1H), 0.91–0.86 (m, 5H), 0.81–0.63 (m, 5H).

^{13}C NMR (100 MHz, $CDCl_3$) δ 176.13, 176.10, 147.44, 147.39, 129.16, 129.15, 116.33, 116.31, 113.0, 112.9, 74.73, 74.70, 68.7, 68.2, 60.3, 60.2, 49.0, 48.7, 47.2, 47.1, 40.8, 40.7, 37.0, 36.8, 34.6, 34.5, 34.38, 34.36, 33.4, 33.2, 31.5, 26.4, 26.3, 25.2, 25.1, 23.4, 23.3, 22.2, 22.1, 21.0, 20.9, 16.2, 16.1.

HRMS (ESI) ***m/z***: Calcd for $C_{24}H_{36}NO_2^+$ [M + H]$^+$: 370.2741; found: 370.2751.

5. Gram level reaction and derivatization experiment

5.1 General procedure for the synthesis of 3 in 3.0 mmol scale

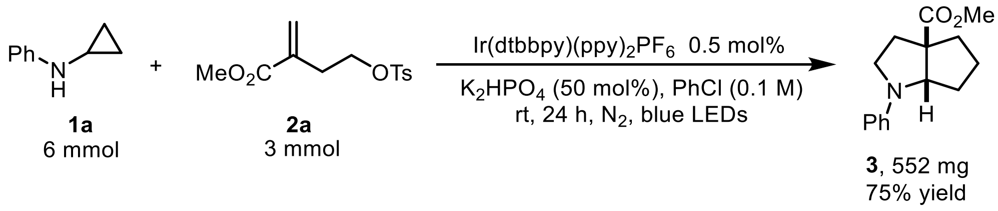

In the glove box, a 10 mL Schlenk tube equipped with a magnetic stir bar was charged with *N*-phenyl cyclopropylamine **1a** (798 mg, 6.00 mmol), **2a** (852 mg, 3.00 mmol), Ir(dtbbpy)(ppy)$_2$PF$_6$ (13.7 mg, 0.015 mmol, 0.5 mol%), K_2HPO_4 (261 mg, 1.5 mmol, 50 mol%) and PhCl (30.0 mL). Then irradiated by blue LEDs for 24 h. After completion of the reaction, the solvent was removed under reduced pressure, and the residue was purified with silica gel chromatography (petroleum ether/ethyl acetate = 100:1, V/V) to give the product **3** (552 mg, 75% yield).

5.2 General procedure for the reduction of 3

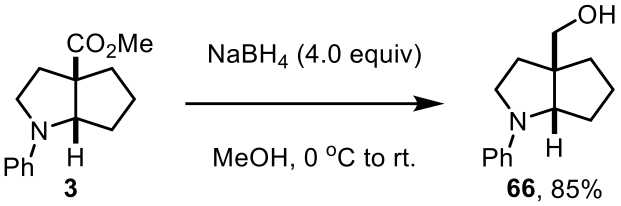

A round-bottom flask equipped with a stir bar was charged with **3** (245 mg, 1 mmol). Then, dry MeOH (20.0 ml) was added, followed by the addition of $NaBH_4$ (148 mg, 4.00 mmol, 4.00 equiv.) in an ice water bath. After the addition is complete, let the reaction proceed at room temperature for 24 h. After completion of the reaction, the solvent was removed under reduced pressure, and the residue was purified with silica gel chromatography (petroleum ether/ethyl acetate = 10:1, V/V) to give the product **66** (85% yield).

1-Phenylhexahydrocyclopenta[*b*]pyrrol-3a(1*H*)-yl)methanol (65)

^{1}H NMR (400 MHz, $CDCl_3$) δ 7.22 (t, J = 8.0 Hz, 2H), 6.68 (t, J = 7.2 Hz, 1H), 6.60 (d, J = 8.0 Hz, 2H), 3.66 (dd, J = 6.0, 2.8 Hz, 1H), 3.56 (q, J = 10.8 Hz, 2H), 3.47 (td, J = 8.8, 4.8 Hz, 1H), 3.24 (dd, J = 16.8, 8.0 Hz, 1H), 2.03 (ddd, J = 12.4, 7.2, 4.8 Hz, 1H), 1.88–1.74 (m, 4H), 1.72–1.65 (m, 2H), 1.64–1.54 (m, 2H).

^{13}C NMR (100 MHz, $CDCl_3$) δ 147.7, 129.2, 116.1, 113.0, 68.6, 66.8, 56.3, 48.4, 35.5, 33.6, 33.0, 24.8.

HRMS (ESI) *m/z*: Calcd for $C_{14}H_{20}NO^+$ $[M + H]^+$: 218.1539; found: 218.1538.

5.3 General procedure for the derivatizations of 66

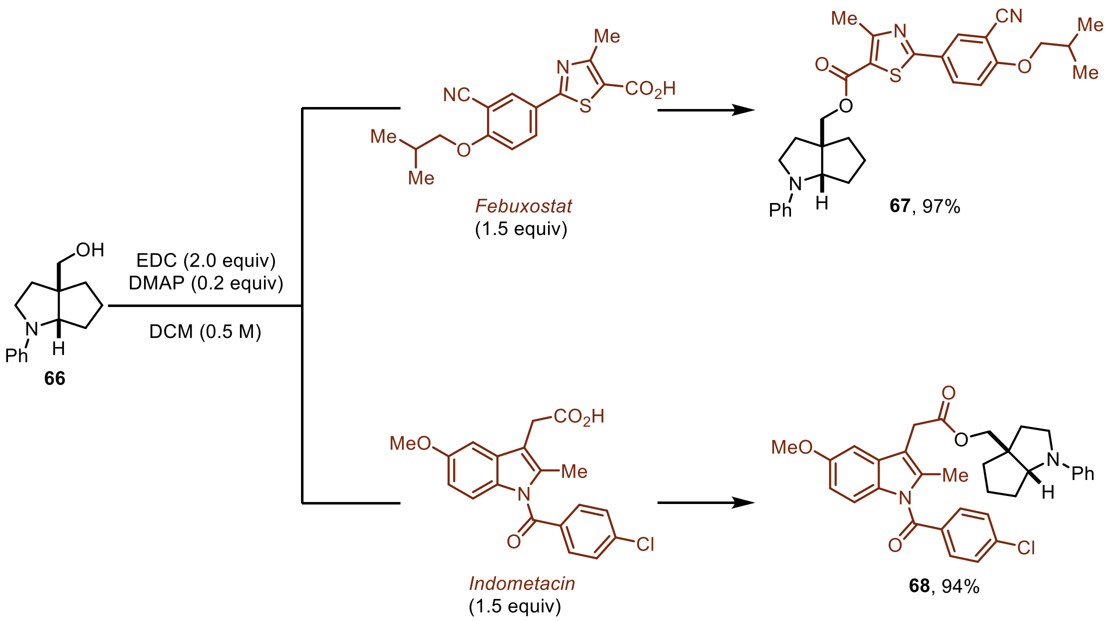

Added **66** (43.5 mg, 0.2 mmol) to a solution of *Febuxostat* or *Indometacin* (1.5 equiv.), DMAP (0.2 equiv.) and EDC (2.0 equiv.) in dry DCM (0.5 M) at 0 °C. Warm the reaction mixture to rt and stir for 12 h. After completion of the reaction, the solvent was removed under reduced pressure, and the residue was purified with silica gel chromatography (petroleum ether/ethyl acetate = 5:1, V/V) to give the product **67** or **68**, respectively.

(1-Phenylhexahydrocyclopenta[*b*]pyrrol-3a(1*H*)-yl)methyl 2-(3-cyano-4-isobutoxyphenyl)-4-methylthiazole-5-carboxylate (67)

100 mg, 97% yield. Colorless oil (Flash column chromatography eluent, petroleum ether/ethyl acetate = 5/1, V/V).

^{1}H NMR (400 MHz, $CDCl_3$) δ 8.09 (d, J = 2.4 Hz, 1H), 8.06–8.01 (m, 1H), 7.26 (t, J = 8.0 Hz, 2H), 6.99 (d, J = 8.8 Hz, 1H), 6.74 (t, J = 7.2 Hz, 1H), 6.64 (d, J = 8.0 Hz, 2H), 4.28 (q, J = 10.8 Hz, 2H), 3.90 (d, J = 6.4 Hz, 2H), 3.85–3.79 (m, 1H), 3.59–3.51 (m, 1H), 3.38–3.30 (m, 1H), 2.74 (s, 3H), 2.25–2.16 (m, 1H), 2.11–1.98 (m, 2H), 1.94–1.81 (m, 2H), 1.80–1.67 (m, 4H), 1.10 (d, J = 6.8 Hz, 6H).

^{13}C NMR (100 MHz, $CDCl_3$) δ 167.5, 162.6, 162.1, 161.5, 147.6, 132.7, 132.2, 129.2, 126.0, 121.5, 116.4, 115.5, 113.0, 112.7, 103.0, 75.8, 70.9, 67.4, 54.1, 48.6, 36.0, 34.4, 33.2, 28.3, 24.7, 19.2, 17.6.

HRMS (ESI) *m/z*: Calcd for $C_{30}H_{34}N_3O_3S^+$ [M + H]$^+$: 516.2315; found: 516.2322

(1-Phenylhexahydrocyclopenta[b]pyrrol-3a(1*H*)-yl)methyl 2-(1-(4-chlorobenzoyl)-5-methoxy-2-methyl-1*H*-indol-3-yl)acetate (68)

105 mg, 94% yield. Colorless oil (Flash column chromatography eluent, petroleum ether/ethyl acetate = 5/1, V/V).

^{1}H NMR (400 MHz, $CDCl_3$) δ 7.67–7.61 (m, 2H), 7.46 (d, J = 8.4 Hz, 2H), 7.19 (t, J = 8.0 Hz, 2H), 6.98–6.94 (m, 1H), 6.87 (d, J = 9.2 Hz, 1H), 6.74–6.59 (m, 1H), 6.49 (d, J = 8.0 Hz, 2H), 4.13–4.02 (m, 2H), 3.82 (s, 3H), 3.69–3.65 (m, 2H), 3.59 (d, J = 4.0 Hz, 1H), 3.47–3.39 (m, 1H), 3.21–3.11 (m, 1H), 2.34 (s, 3H), 1.93–1.79 (m, 2H), 1.73–1.52 (m, 5H), 1.30–1.25 (m, 1H).

^{13}C NMR (100 MHz, $CDCl_3$) δ 171.1, 168.3, 156.2, 147.4, 139.4, 136.0, 134.0, 131.3, 130.9, 130.7, 129.23, 129.17, 116.3, 115.1, 112.9, 112.7, 111.7, 101.4, 69.7, 66.9, 55.8, 54.0, 48.2, 35.8, 34.0, 32.9, 30.6, 24.6, 13.4.

HRMS (ESI) *m/z*: Calcd for $C_{33}H_{34}ClN_2O_4^+$ [M + H]$^+$: 557.2202; found: 557.2208.

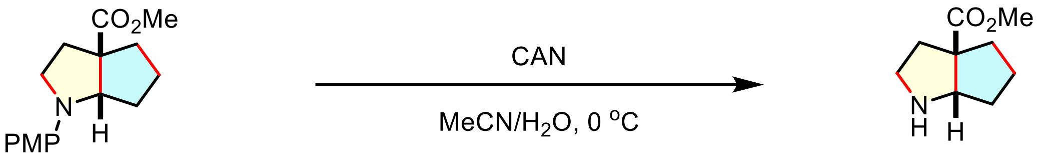

7 **69**, 66%

A round bottomed flask was charged with **7** (20.6 mg, 0.075 mmol, 1 equiv) in acetonitrile (0.75 mL), flushed with argon and cooled to 0 ºC. A separate round bottomed flask was charged with ceric ammonium nitrate (123 mg, 0.224 mmol, 3 equiv) in water (0.25 mL) and flushed with argon. The ceric ammonium nitrate solution was added dropwise to the reaction and let stir for 0.5 h. The reaction was quenched with 3 mL of water. The aqueous layer was extracted three times with Et_2O. The aqueous layer was basified with 1 M aqueous NaOH until the solution reached a pH of 9. The aqueous layer was then extracted three times with DCM. The combined organic layers were washed with brine, dried over Na_2SO_4, and concentrated under reduced pressure afforded the corresponding product.

Methyl hexahydrocyclopenta[*b*]pyrrole-3a(1*H*)-carboxylate (**69**)

8.3 mg, 66 % yield. Brown oil.

^{1}H NMR (400 MHz, $CDCl_3$) δ 3.94–3.82 (m, 1H), 3.69 (s, 3H), 3.06–2.88 (m, 2H), 2.70–2.40 (m, 2H), 2.37–2.27 (m, 1H), 2.22–2.14 (m, 1H), 1.91–1.83 (m, 1H), 1.71–1.58 (m, 4H).

^{13}C NMR (100 MHz, $CDCl_3$) δ 178.1, 69.7, 60.1, 52.3, 47.4, 39.5, 37.3, 34.0, 25.3.

HRMS (ESI) *m/z*: Calcd for $C_9H_{16}NO_2^+$ [M+H] $^+$: 170.1176; found: 170.1175.

1a +

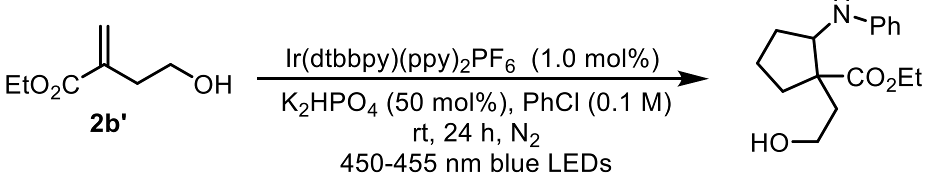

2b' **70**, 38%

In the glove box, a 10 mL Schlenk tube equipped with a magnetic stir bar was charged with *N*-phenyl cyclopropylamine (**1a**, 53.2 mg, 0.4 mmol), ethyl 4-hydroxy-2-methylenebutanoate (**2b'**, 28.8 mg, 0.2 mmol), $Ir(dtbbpy)(ppy)_2PF_6$ (1.8 mg, 0.002 mmol, 1 mol%), K_2HPO_4 (17.4 mg, 0.1 mmol, 50 mol%) and PhCl (2.0 mL). The

reaction mixture was stirred under 2×3 W blue LEDs (450−455 nm) at room temperature with stirring for 24 h. After completion of the reaction, the solvent was removed under reduced pressure, and the residue was purified with silica gel chromatography (petroleum ether/ethyl acetate = 10:1, V/V) to give the product **70**.

Ethyl 1-(2-hydroxyethyl)-2-(phenylamino)cyclopentane-1-carboxylate (70)

21.1 mg, 38 % yield. Yellow oil (Flash column chromatography eluent, petroleum ether/ethyl acetate = 10/1, V/V).

^{1}H NMR (400 MHz, $CDCl_3$) δ 7.15 (t, J = 8.0 Hz, 2H), 6.69 (t, J = 7.2 Hz, 1H), 6.65 (d, J = 8.0 Hz, 2H), 4.25 (t, J = 6.8 Hz, 1H), 4.19–4.06 (m, 2H), 3.67 (t, J = 6.8 Hz, 2H), 2.93 (s, 1H), 2.35–2.26 (m, 1H), 2.18–2.10 (m, 2H), 1.81–1.67 (m, 4H), 1.56–1.47 (m, 1H), 1.23 (t, J = 7.2 Hz, 3H).

^{13}C NMR (100 MHz, $CDCl_3$) δ 177.4, 147.5, 129.4, 117.9, 113.7, 61.3, 61.1, 60.1, 55.3, 34.8, 33.6, 32.5, 21.3, 14.2.

HRMS (ESI) *m/z*: Calcd for $C_{16}H_{23}NO_3^+$ [M+H] $^+$: 278.1751; found: 278.1748.

6. Mechanistic studies

6.1 Radical trapping experiments

To further investigate the mechanism of this photocatalytic radical reaction, a control experiment was conducted using radical scavenger TEMPO or BHT.

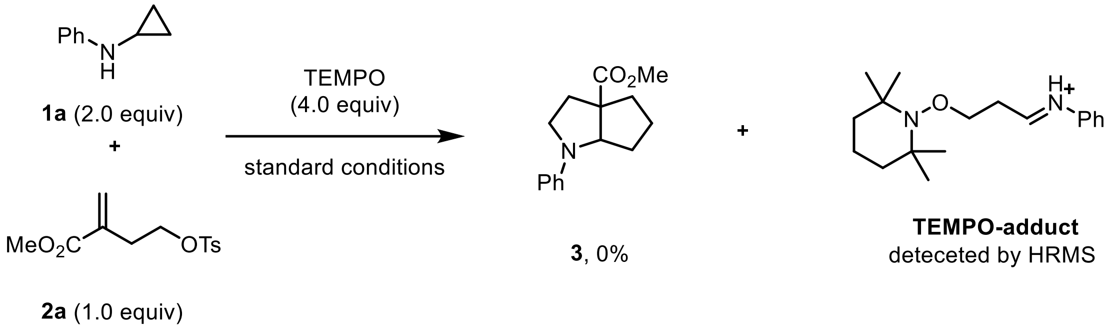

In the glove box, a 10 mL Schlenk tube equipped with a magnetic stir bar was charged with *N*-phenyl cyclopropylamine **1a** (53.2 mg, 0.4 mmol), **2a** (56.8 mg, 0.2 mmol), Ir(dtbbpy)(ppy)$_2$PF$_6$ (0.91 mg, 0.001 mmol, 0.5 mol%), K_2HPO_4 (17.4 mg, 0.1 mmol, 50 mol%) and PhCl (2.0 mL). Then TEMPO (126 mg, 0.8 mmol, 4.0 equiv.) was

added to the mixture, and irradiated by blue LEDs (450−455 nm) for 24 h. After completion of the reaction, HRMS analysis of this reaction crude mixture showed that the corresponding TEMPO-adduct was detected (Figure S2) and no product **3** was observed.

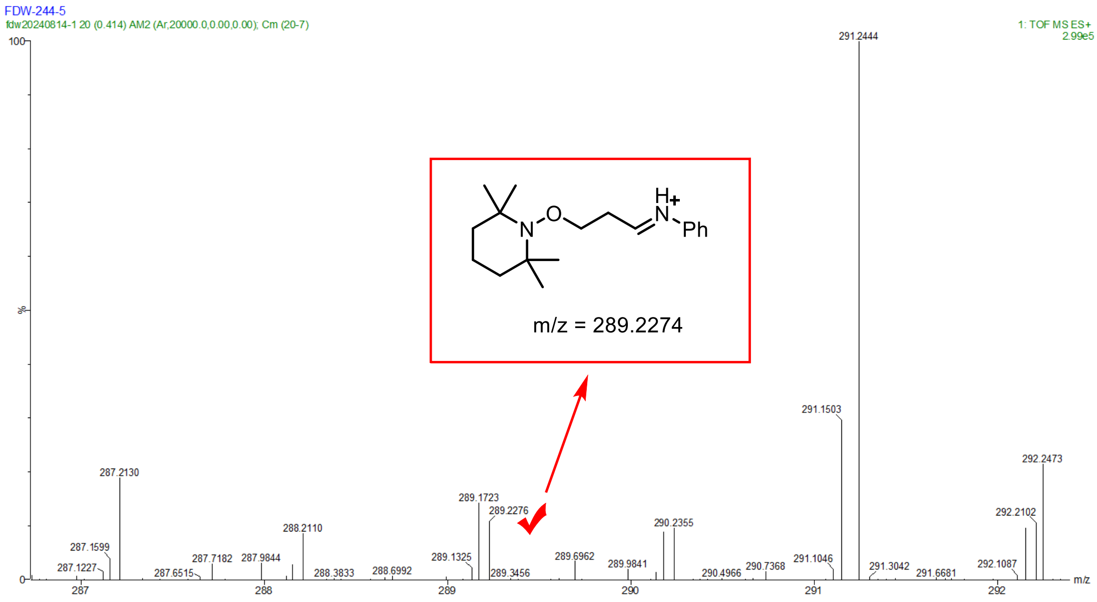

Figure S2. HRMS analysis of TEMPO-adduct

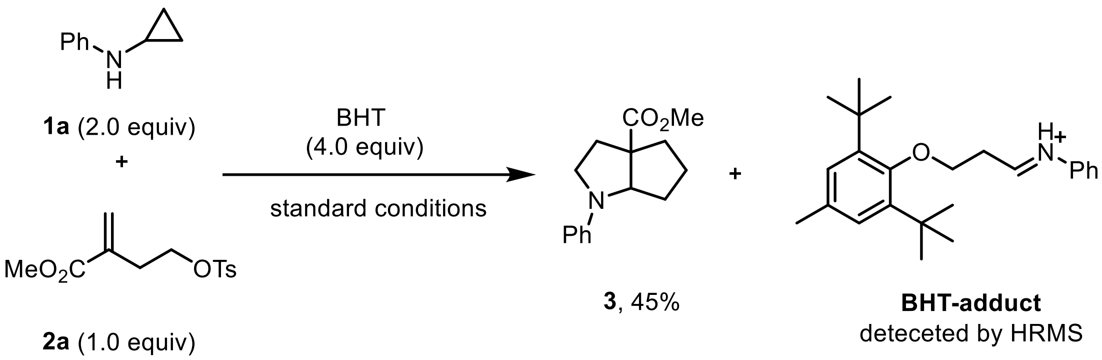

In the glove box, a 10 mL Schlenk tube equipped with a magnetic stir bar was charged with *N*-phenyl cyclopropylamine **1a** (53.2 mg, 0.4 mmol), **2a** (56.8 mg, 0.2 mmol), Ir(dtbbpy)(ppy)$_2$PF$_6$ (0.91 mg, 0.001 mmol, 0.5 mol%), K_2HPO_4 (17.4 mg, 0.1 mmol, 50 mol%) and PhCl (2.0 mL). Then BHT (176 mg, 0.8 mmol, 4.0 equiv) was added to the mixture, then irradiated by blue LEDs (λ = 450−455 nm) for 24 h. After completion of the reaction, from crude ^{1}H-NMR wither inter standard, the reaction was

inhibited and product **3** was formed in 45% yield. HRMS analysis of this reaction crude mixture showed that the corresponding BHT-adduct was observed (Figure S3).

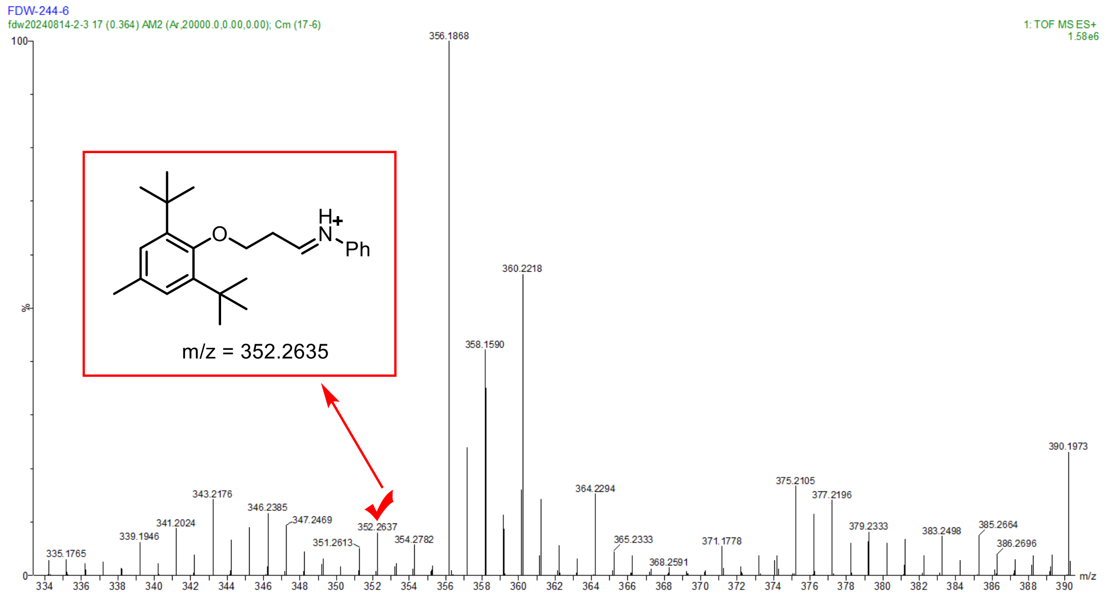

Figure S3. HRMS analysis of BHT-adduct

These results supported the speculation that the reaction proceeded *via* a radical pathway and confirmed the generation of amino radical cation.

6.2 Time profile of the transformation with the light ON/OFF over time.

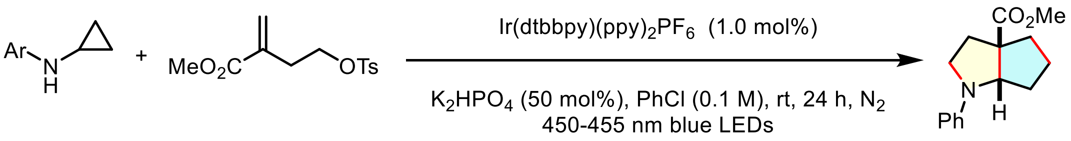

1a **2a** **3**

In the glove box, a 10 mL Schlenk tube equipped with a magnetic stir bar was charged with *N*-phenyl cyclopropylamine **1a** (133 mg, 1.0 mmol), **2a** (142 mg, 0.5 mmol), Ir(dtbbpy)(ppy)$_2$PF$_6$ (2.3 mg, 0.001 mmol, 0.5 mol%), K_2HPO_4 (43.5 mg, 0.25 mmol, 50 mol%) and PhCl (5.0 mL). The tube was sealed, irradiated with blue LEDs (450−455 nm). The mixture was stirred under blue light irradiation at ambient temperature for the 4 h, then 1.0 ml of the reaction mixture was extracted from the system, turn off the light, react for 4 h, then take out 1.0 mL from the remaining reaction solution and add it to the internal standard for nuclear magnetic analysis. Turn on the

light and continue the reaction for 4 hours, then take out 1.0ml from the remaining reaction solution and add it to the internal standard for nuclear magnetic analysis. Perform nuclear magnetic analysis every 4 h until the reaction time reaches 24 h. The yield of **3** determined by ^{1}H NMR using CH_2Br_2 as an internal standard.

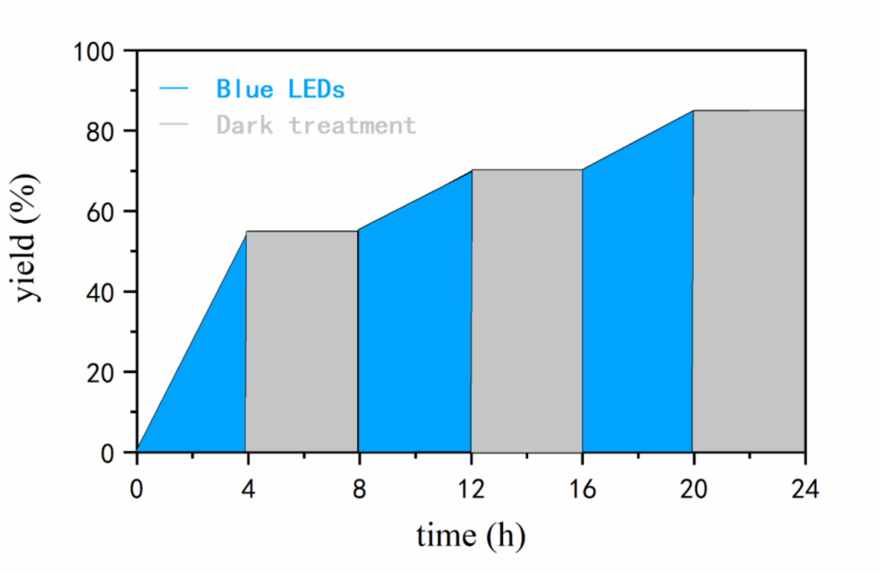

Figure S4. Time profile of the transformation with the light **ON/OFF** over time

7. X-ray structure and data for 33, 43, 55

7.1 Crystallographic data and molecular structure of **33** (CCDC: 2379567).

General procedure for crystal culture of **33**: To a test tube (15 mL) with added **33** (20 mg), dichloromethane (1.0 mL) was added slowly to make it dissolve completely. After it dissolved, a mixture of petroleum ether (2.0 mL) and EtOAc (3.0 mL) was added. Then, the test tube was sealed with a rubber stopper, and connected to air with a syringe needle. Finally, the tube was put in a dry and ventilated place to make the organic solvent to volatilize slowly. After a few days, the crystal of **33** was obtained. The X-ray crystal structure of **33** was shown in Figure **S5**.

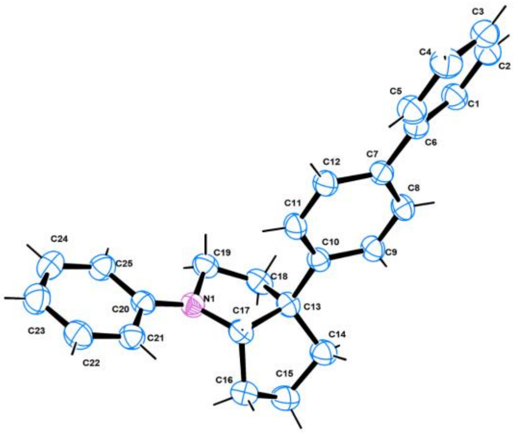

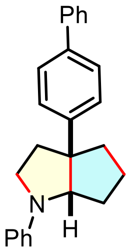

33

Figure S5. ORTEP diagram of **33** with thermal displacement parameters drawn at 30% probability

---

Bond precision: C-C = 0.0043 A Wavelength=0.71073

Cell:	a=11.132(5)	b=12.623(6)	c=14.347(6)
	alpha=90	beta=110.582(14)	gamma=90
Temperature:	300 K		

	Calculated	Reported
Volume	1887.4(15)	1887.1(15)
Space group	P 21/n	P 1 21/n 1
Hall group	-P 2yn	-P 2yn
Moiety formula	C25 H25 N	C25 H25 N
Sum formula	C25 H25 N	C25 H25 N
Mr	339.46	339.46
Dx,g cm-3	1.195	1.195
Z	4	4
Mu (mm-1)	0.068	0.068
F000	728.0	728.0
F000’	728.25	
h,k,lmax	13,15,17	13,15,17
Nref	3516	3502
Tmin,Tmax	0.984,0.988	0.622,0.746
Tmin’	0.984	

Correction method= # Reported T Limits: Tmin=0.622 Tmax=0.746
AbsCorr = ?

Data completeness= 0.996 Theta(max)= 25.500

R(reflections)= 0.0610(2089) wR2(reflections)= 0.1896(3502)

S = 1.032 Npar= 235

7.2 Crystallographic data and molecular structure of **43** (CCDC = 2379565).

General procedure for crystal culture of **43**: To a test tube (15 mL) with added **43** (20 mg), dichloromethane (1.0 mL) was added slowly to make it dissolve completely. After it dissolved, a mixture of petroleum ether (2.0 mL) and EtOAc (3.0 mL) was added. Then, the test tube was sealed with a rubber stopper, and connected to air with a syringe needle. Finally, the tube was put in a dry and ventilated place to make the organic solvent to volatilize slowly. After a few days, the crystal of **43** was obtained. The X-ray crystal structure of **43** was shown in Figure **S6**.

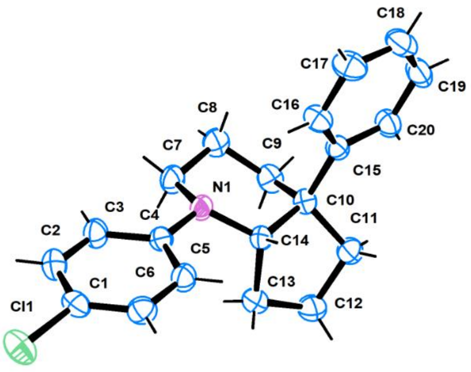

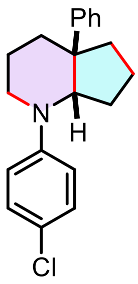

43

Figure S6. ORTEP diagram of **43** with thermal displacement parameters drawn at 30% probability

Bond precision:	C-C = 0.0043 A		Wavelength=0.71073
Cell:	a=11.132(5)	b=12.623(6)	c=14.347(6)
	alpha=90	beta=110.582(14)	gamma=90
Temperature:	300 K		

	Calculated	Reported
Volume	1887.4(15)	1887.1(15)
Space group	P 21/n	P 1 21/n 1
Hall group	-P 2yn	-P 2yn
Moiety formula	C25 H25 N	C25 H25 N
Sum formula	C25 H25 N	C25 H25 N
Mr	339.46	339.46
Dx,g cm-3	1.195	1.195
Z	4	4
Mu (mm-1)	0.068	0.068
F000	728.0	728.0
F000'	728.25	
h,k,lmax	13,15,17	13,15,17
Nref	3516	3502
Tmin,Tmax	0.984,0.988	0.622,0.746
Tmin'	0.984	

Correction method= # Reported T Limits: Tmin=0.622 Tmax=0.746
AbsCorr = ?

Data completeness= 0.996 Theta(max)= 25.500

R(reflections)= 0.0610(2089) wR2(reflections)= 0.1896(3502)

S = 1.032 Npar= 235

7.3 Crystallographic data and molecular structure of **55** (CCDC = 2379566).

General procedure for crystal culture of **55**: To a test tube (15 mL) with added **55** (20 mg), dichloromethane (1.0 mL) was added slowly to make it dissolve completely. After it dissolved, a mixture of petroleum ether (2.0 mL) and EtOAc (3.0 mL) was added. Then, the test tube was sealed with a rubber stopper, and connected to air with a syringe needle. Finally, the tube was put in a dry and ventilated place to make the organic solvent to volatilize slowly. After a few days, the crystal of **55** was obtained. The X-ray crystal structure of **55** was shown in Figure **S7**.

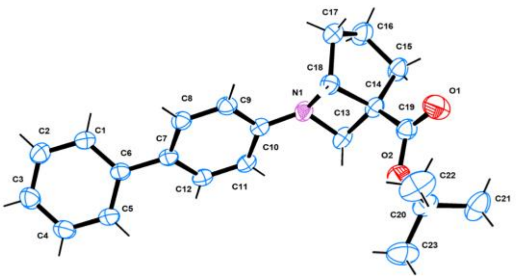

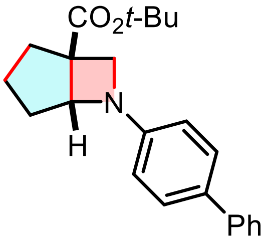

55

Figure S7. ORTEP diagram of **55** with thermal displacement parameters drawn at 30% probability

---

Bond precision: C-C = 0.0023 A Wavelength=1.54178

Cell:	a=6.3681(1)	b=23.3400(4)	c=13.4530(2)
	alpha=90	beta=97.905(1)	gamma=90
Temperature:	273 K		

	Calculated	Reported
Volume	1980.54(6)	1980.54(5)
Space group	P 21/c	P 1 21/c 1
Hall group	-P 2ybc	-P 2ybc
Moiety formula	C23 H27 N O2	C23 H27 N O2
Sum formula	C23 H27 N O2	C23 H27 N O2
Mr	349.46	349.45
Dx,g cm-3	1.172	1.172
Z	4	4
Mu (mm-1)	0.579	0.579
F000	752.0	752.0
F000'	754.08	
h,k,lmax	7,28,16	7,28,16
Nref	3630	3586
Tmin,Tmax	0.891,0.901	0.518,0.753
Tmin'	0.891	

Correction method= # Reported T Limits: Tmin=0.518 Tmax=0.753
AbsCorr = MULTI-SCAN

Data completeness= 0.988 Theta(max)= 68.290

R(reflections)= 0.0440(3201) wR2(reflections)= 0.1204(3586)

S = 1.031 Npar= 238

8. References

[1] (a) Maity, S., Zhu, M., Shinabery, R. S. & Zheng, N. Intermolecular [3+2] Cycloaddition of Cyclopropylamines with Olefins by Visible-Light Photocatalysis. *Angew. Chem. Int. Ed.* **51**, 222–226 (2011). (b) Muriel, B., Gagnebin, A. & Waser, J. Synthesis of Bicyclo[3.1.0]hexanes by (3 + 2) Annulation of Cyclopropenes with Aminocyclopropanes. *Chem. Sci.* **10**, 10716–10722 (2019). (c) Kuang, Y., Ning, Y., Zhu, J. & Wang, Y. Dirhodium(II)-Catalyzed (3 + 2) Cycloaddition of the *N*-Arylaminocyclopropane with Alkene Derivatives. *Org. Lett.* **20**, 2693–2697 (2018). (d) Cui, W. & Loeppky, R. N. The Synthesis of *N*-Arylcyclopropylamines via Palladium-catalyzed C–N Bond Formation. *Tetrahedron* **57**, 2953–2956 (2001). (e) Lv, S., Xu, W. F., Yang, T. Y., Lan, M. X., Xiao, R. X., Mou, X. Q., Chen, Y. Z. & Cui, B. D. Iron(II)-Catalyzed Radical [3 + 2] Cyclization of *N*-Aryl Cyclopropylamines for the Synthesis of Polyfunctionalized Cyclopentylamines. *Org. Lett.* **26**, 3151–3157 (2024).

[2] Li, H., Zhang, Y., Yang, X., Deng, Z., Zhu, Z., Zhou, P., Ouyang, X., Yuan, Y., Chen, X., Yang, L., Liu, M. & Shu, C. Synthesis of Multifluoromethylated γ-Sultines by a Photoinduced Radical Addition-Polar Cyclization. *Angew. Chem. Int. Ed.* **62**, e202300159 (2023).

[3] Pasini, D., Klopp, J. M. & Fréchet, J. M. J. Design, Synthesis, and Characterization of Carbon-Rich Cyclopolymers for 193 Nm Microlithography. *Chem. Mater.* **13**, 4136–4146 (2001).

9. NMR spectra of the products

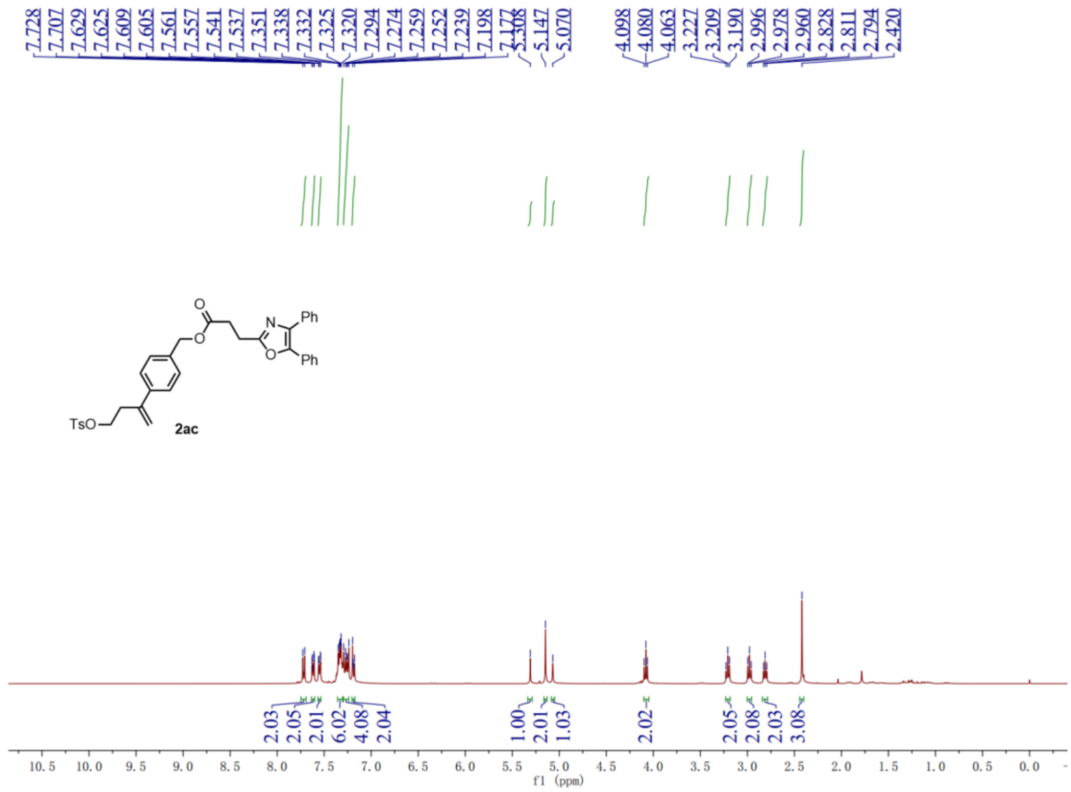

^{1}H NMR Spectrum of Compound **2ac** (400 MHz, $CDCl_3$)

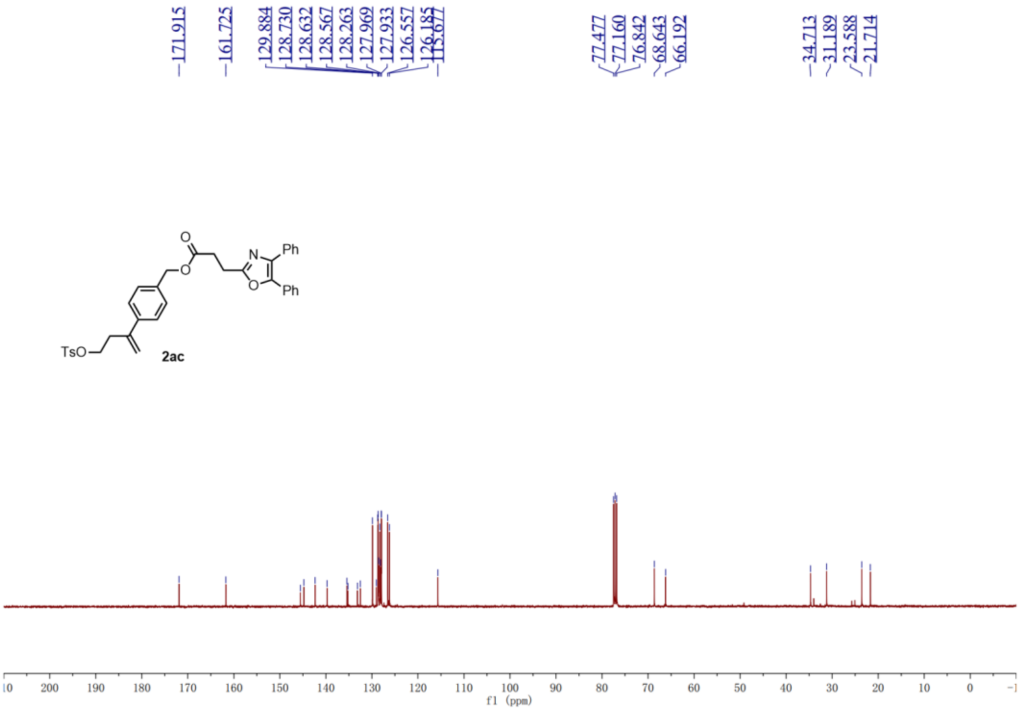

^{13}C{^{1}H} NMR Spectrum of Compound **2ac** (100 MHz, $CDCl_3$)

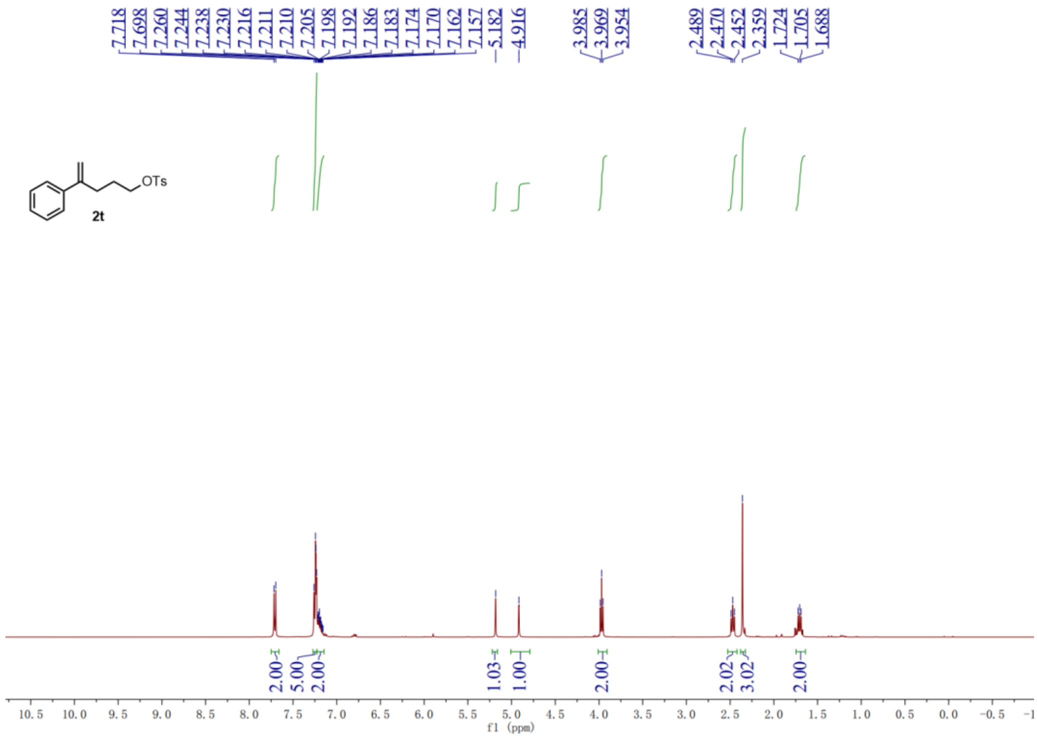

^{1}H NMR Spectrum of Compound **2t** (400 MHz, $CDCl_3$)

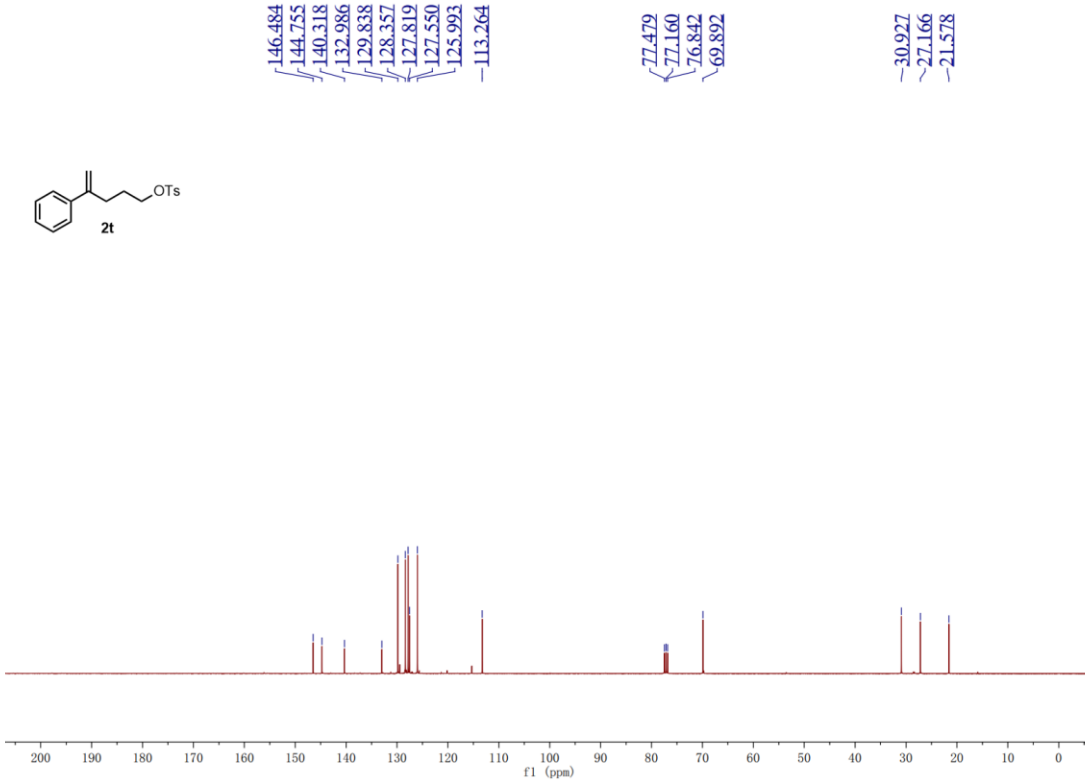

^{13}C{^{1}H} NMR Spectrum of Compound **2t** (100 MHz, $CDCl_3$)

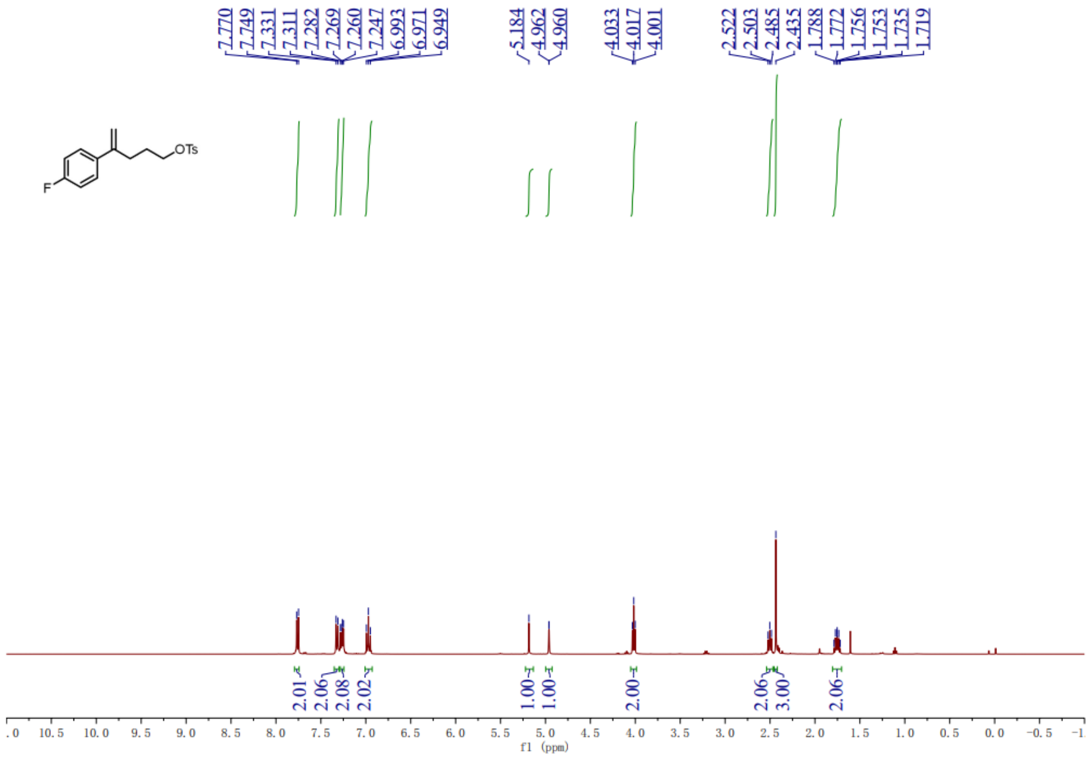

^{1}H NMR Spectrum of Compound **2u** (400 MHz, $CDCl_3$)

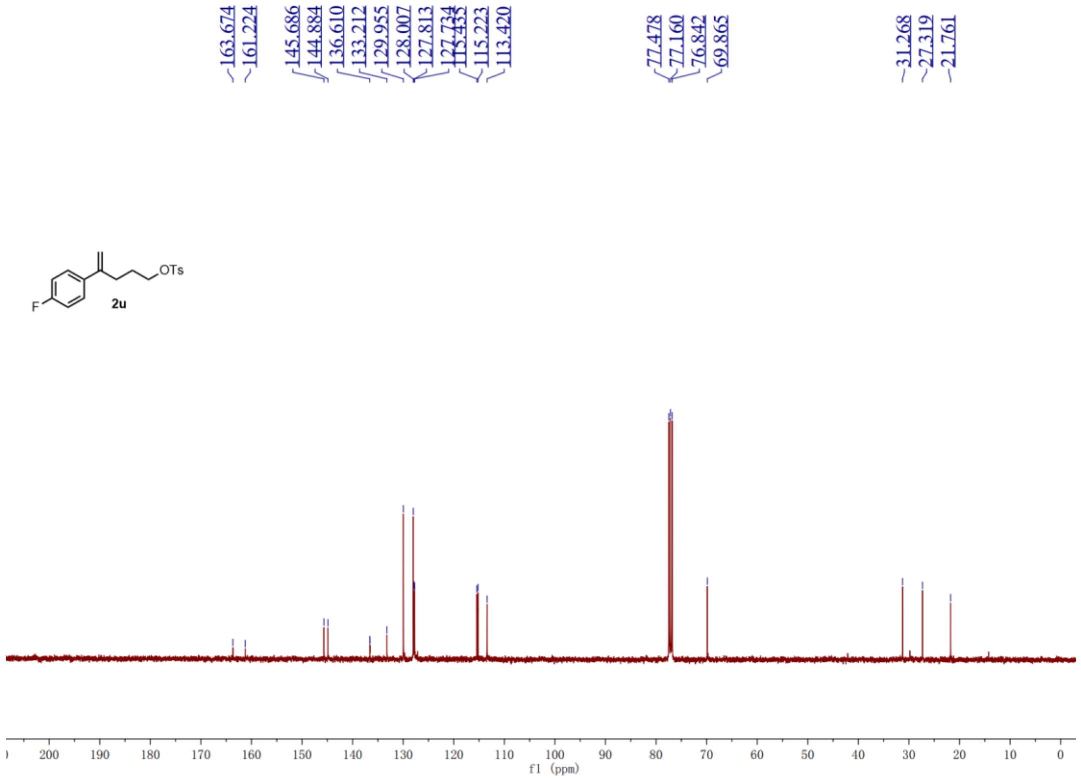

$^{13}C\{^{1}H\}$ NMR Spectrum of Compound **2u** (100 MHz, $CDCl_3$)

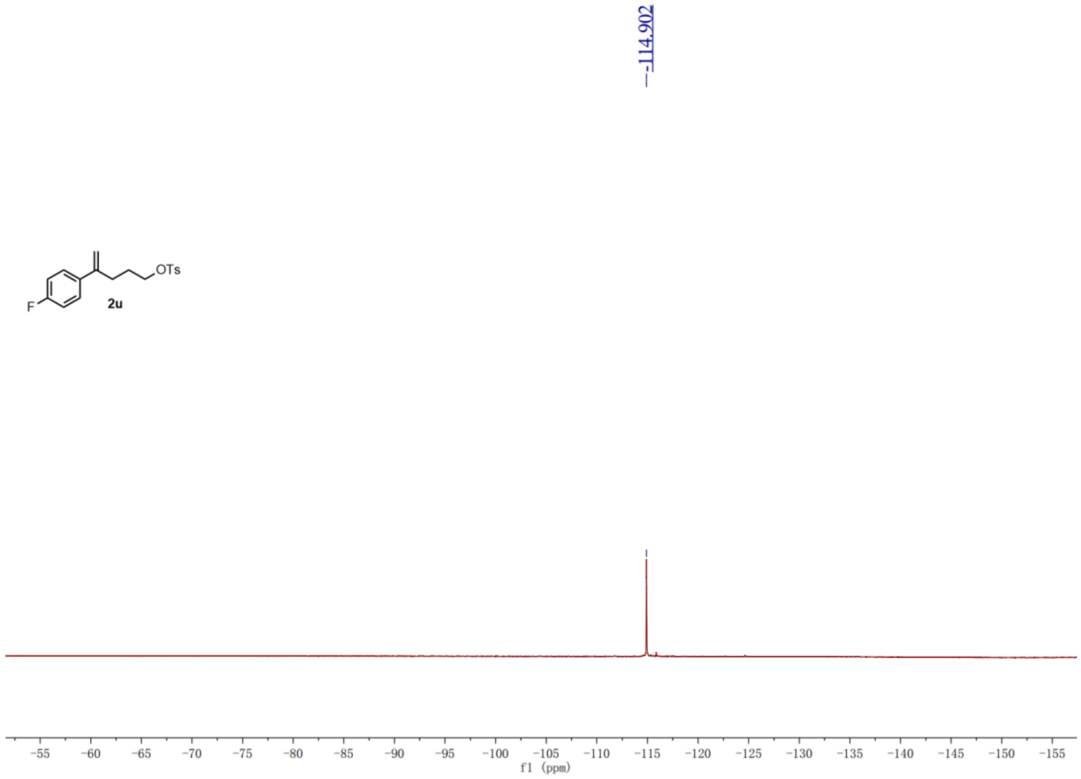

^{19}F{^{1}H} NMR Spectrum of Compound **2u** (376 MHz, $CDCl_3$)

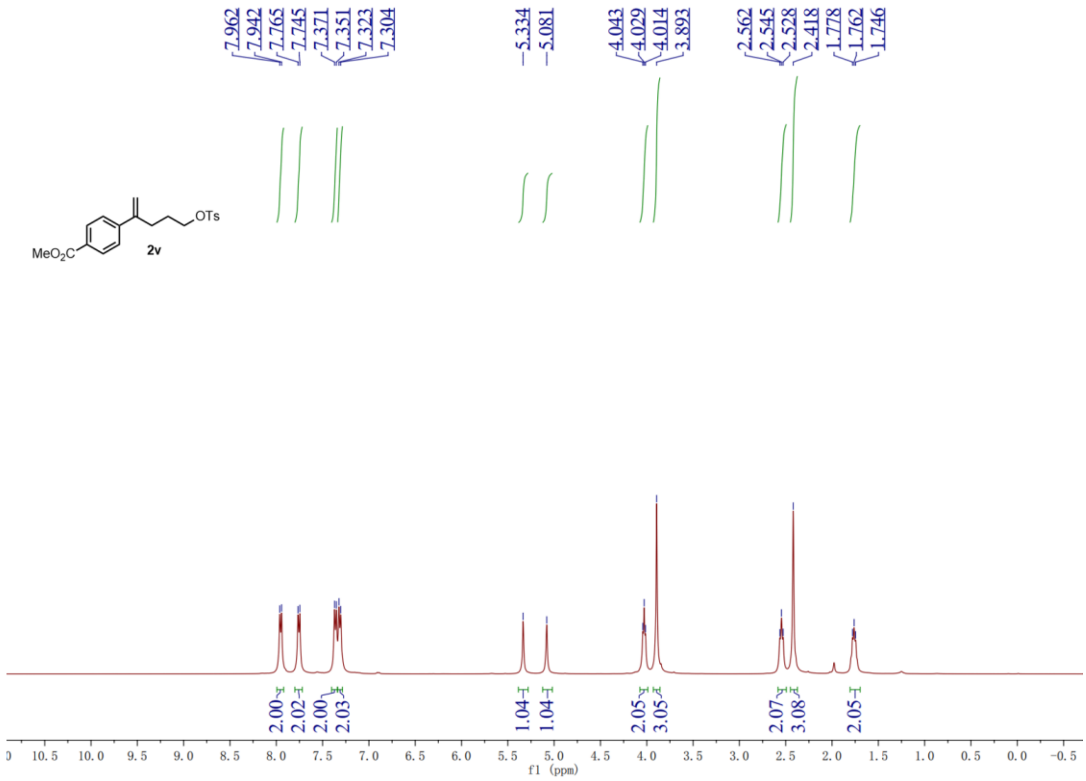

^{1}H NMR Spectrum of Compound **2v** (400 MHz, $CDCl_3$)

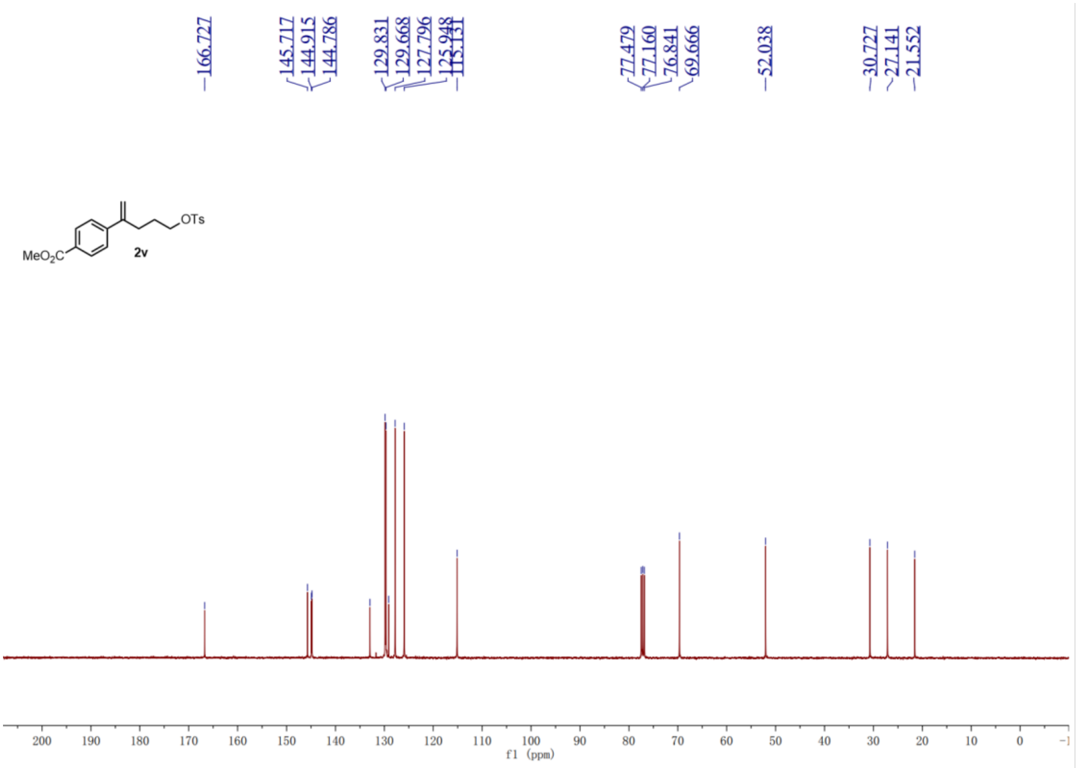

$^{13}C\{^1H\}$ NMR Spectrum of Compound **2v** (100 MHz, $CDCl_3$)

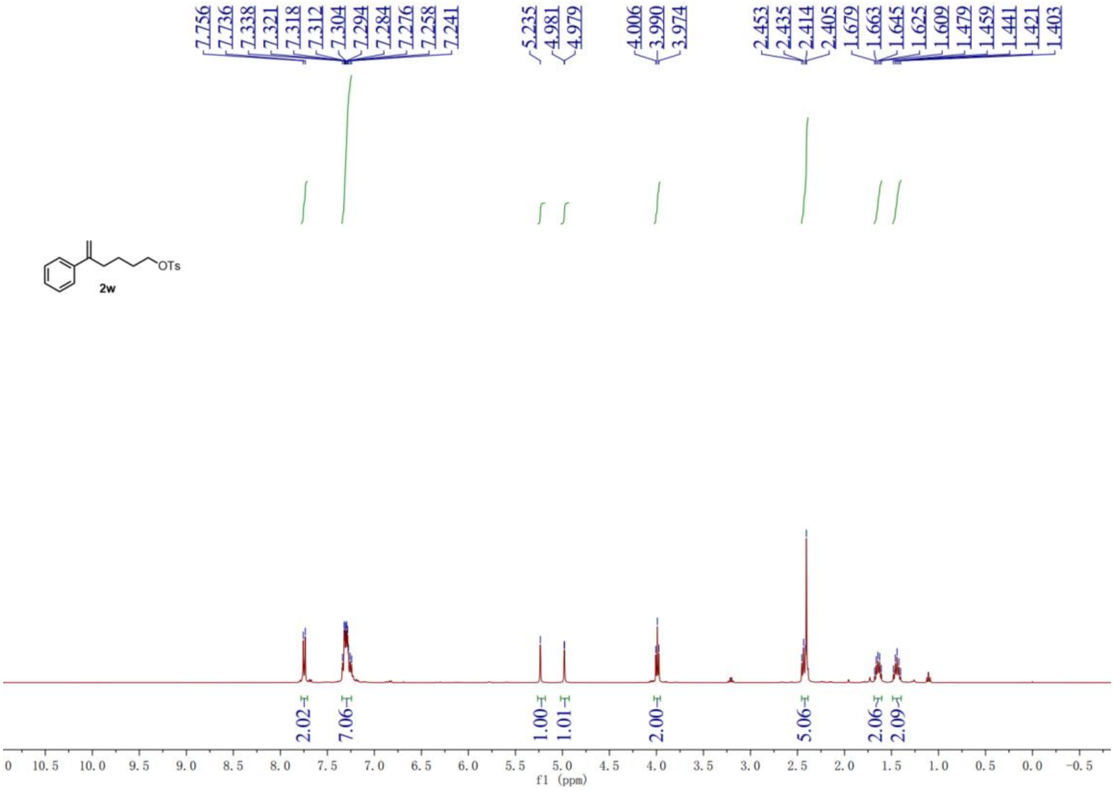

^1H NMR Spectrum of Compound **2w** (400 MHz, $CDCl_3$)

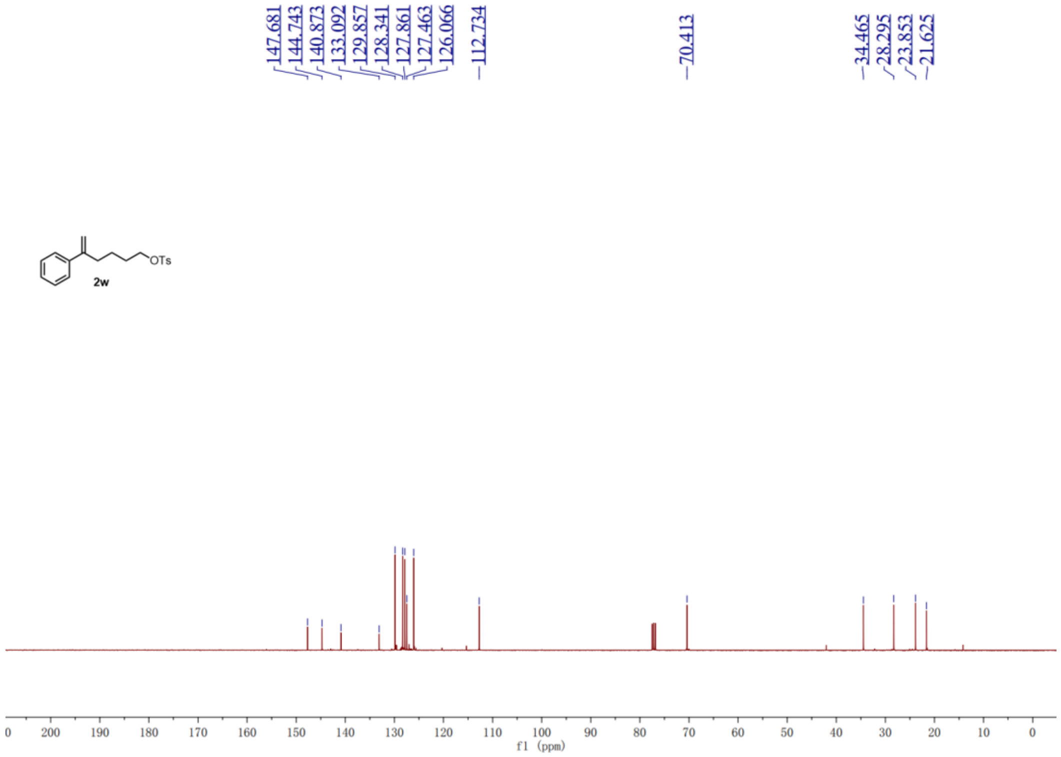

$^{13}C\{^{1}H\}$ NMR Spectrum of Compound **2w** (100 MHz, $CDCl_3$)

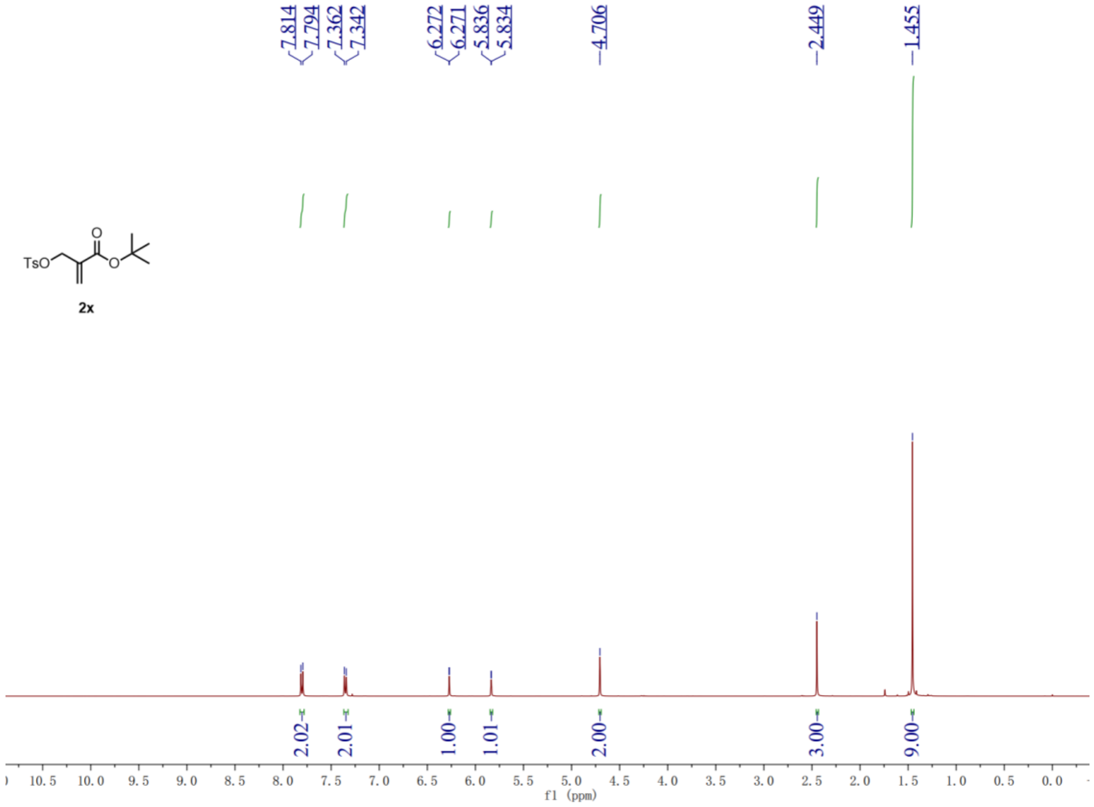

^{1}H NMR Spectrum of Compound **2x** (400 MHz, $CDCl_3$)

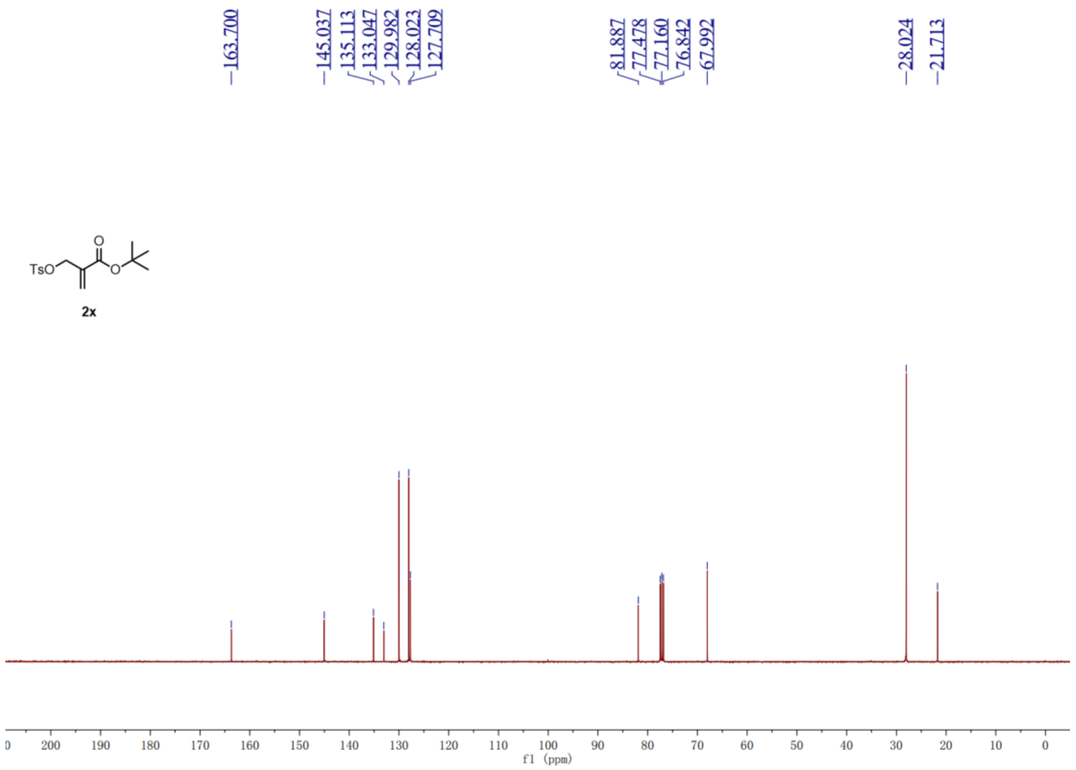

$^{13}C\{^{1}H\}$ NMR Spectrum of Compound **2x** (100 MHz, $CDCl_3$)

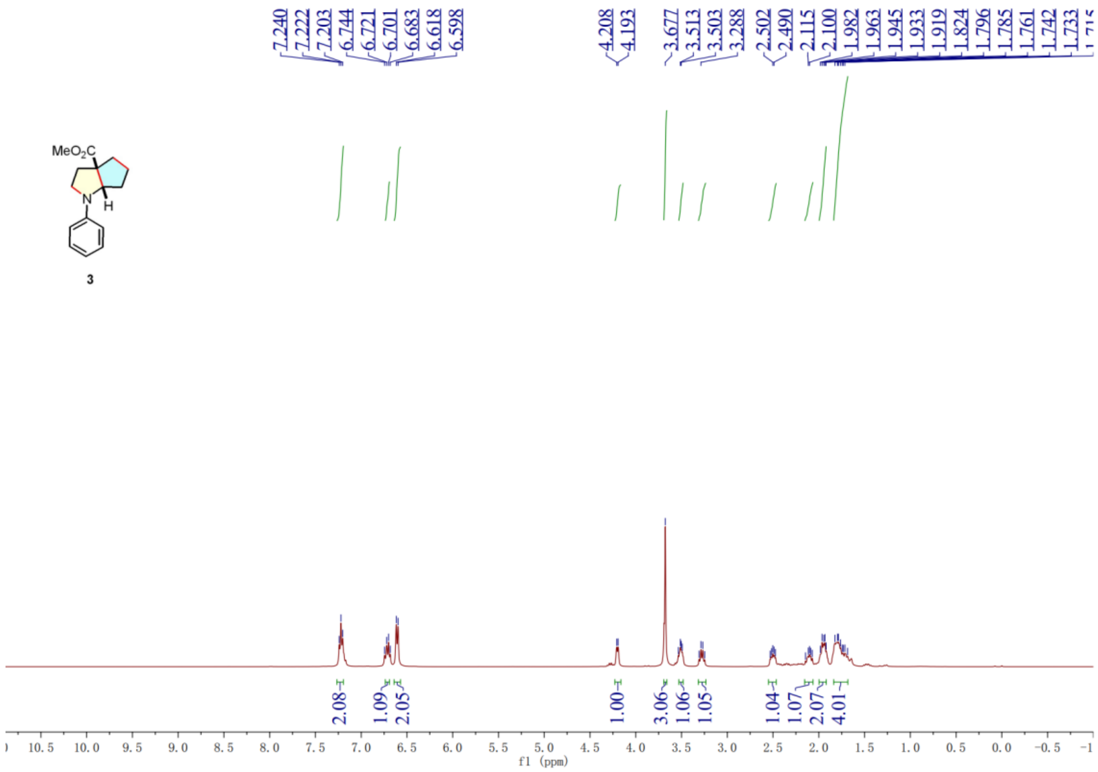

^{1}H NMR Spectrum of Compound **3** (400 MHz, $CDCl_3$)

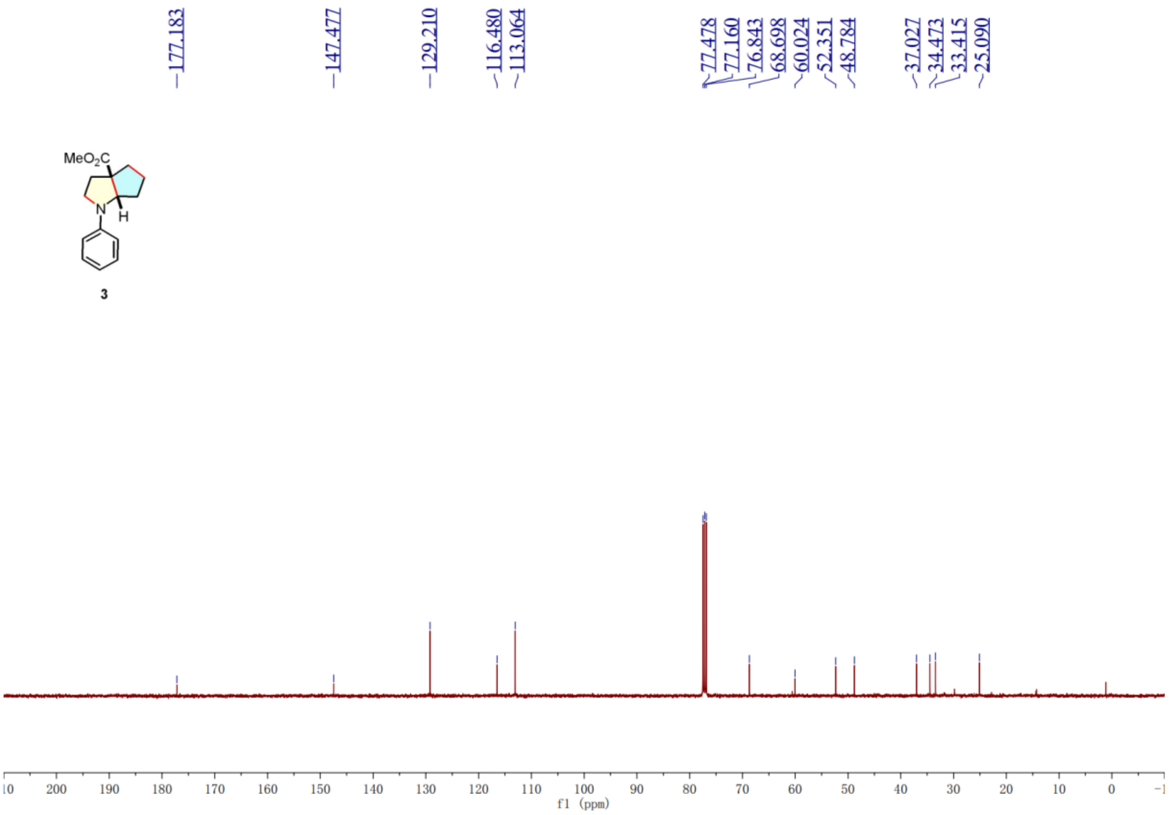

$^{13}C\{^{1}H\}$ NMR Spectrum of Compound **3** (100 MHz, $CDCl_3$)

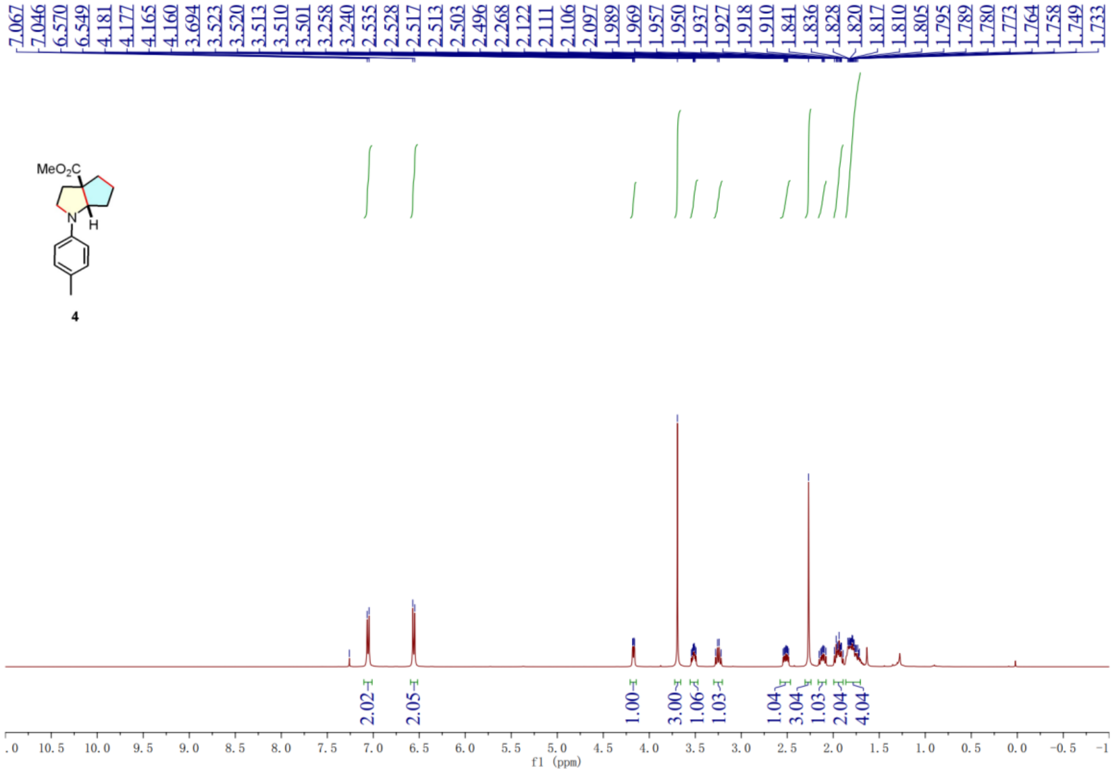

^{1}H NMR Spectrum of Compound **4** (400 MHz, $CDCl_3$)

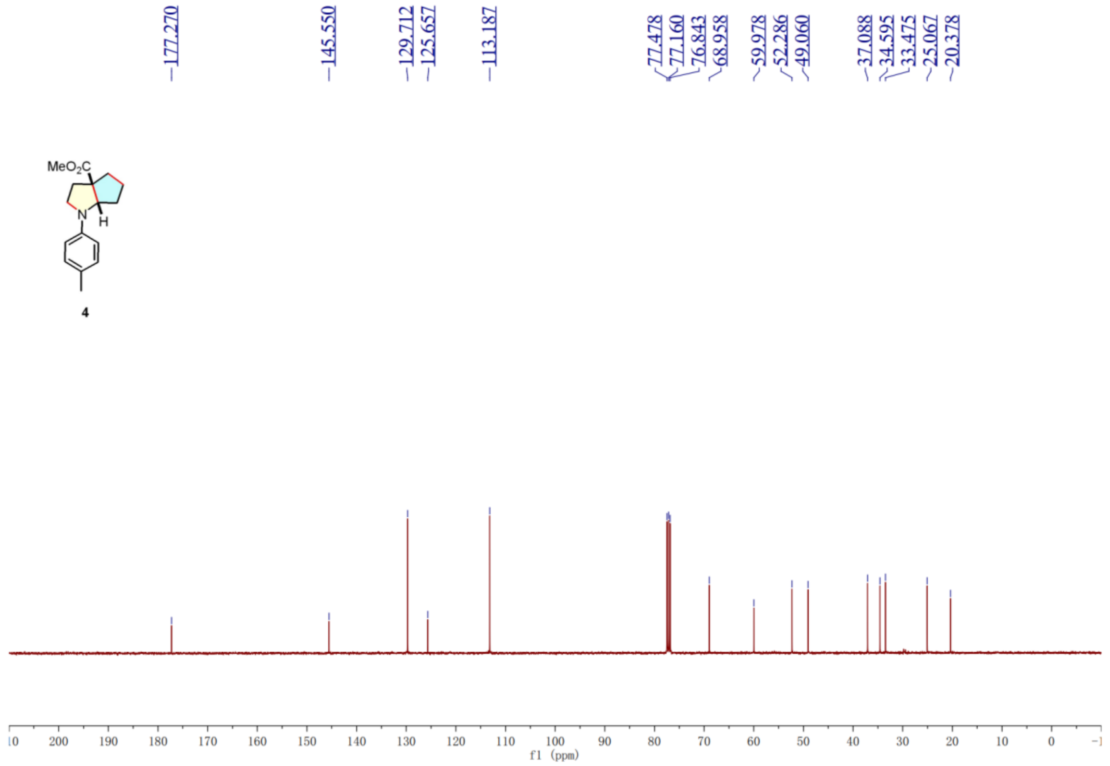

$^{13}C\{^{1}H\}$ NMR Spectrum of Compound **4** (100 MHz, $CDCl_3$)

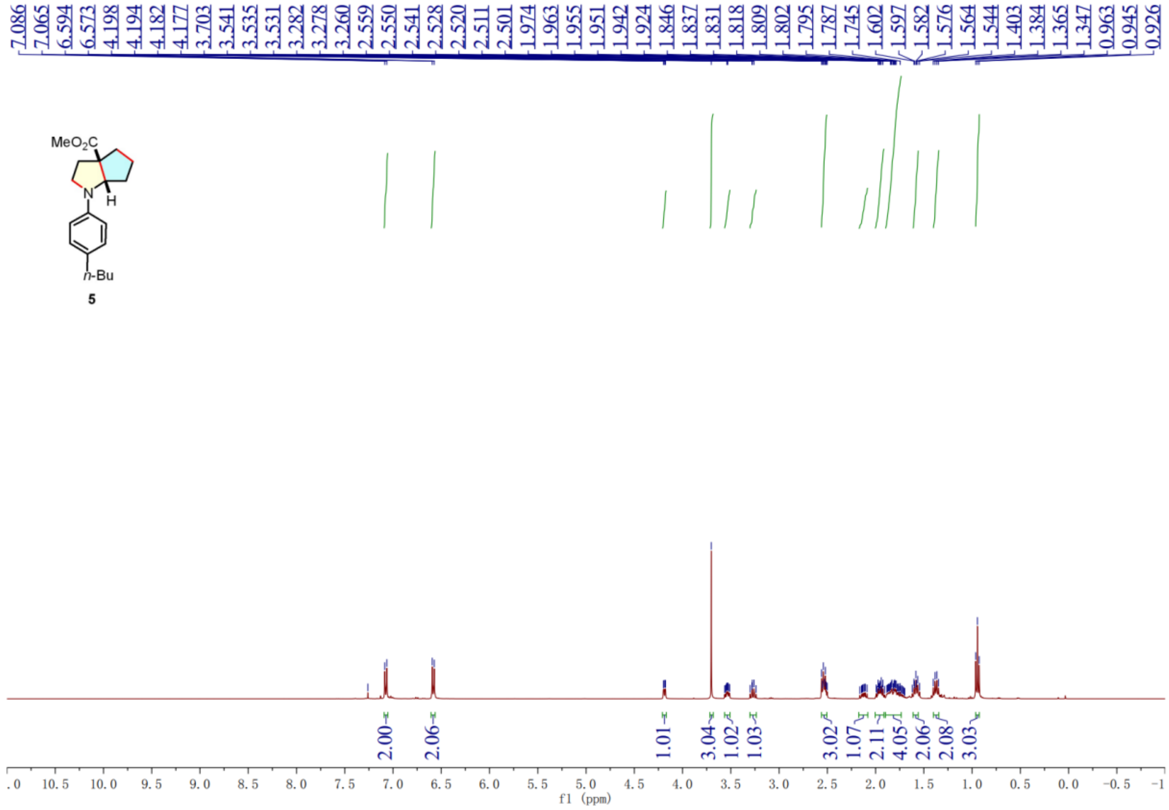

^{1}H NMR Spectrum of Compound **5** (400 MHz, $CDCl_3$)

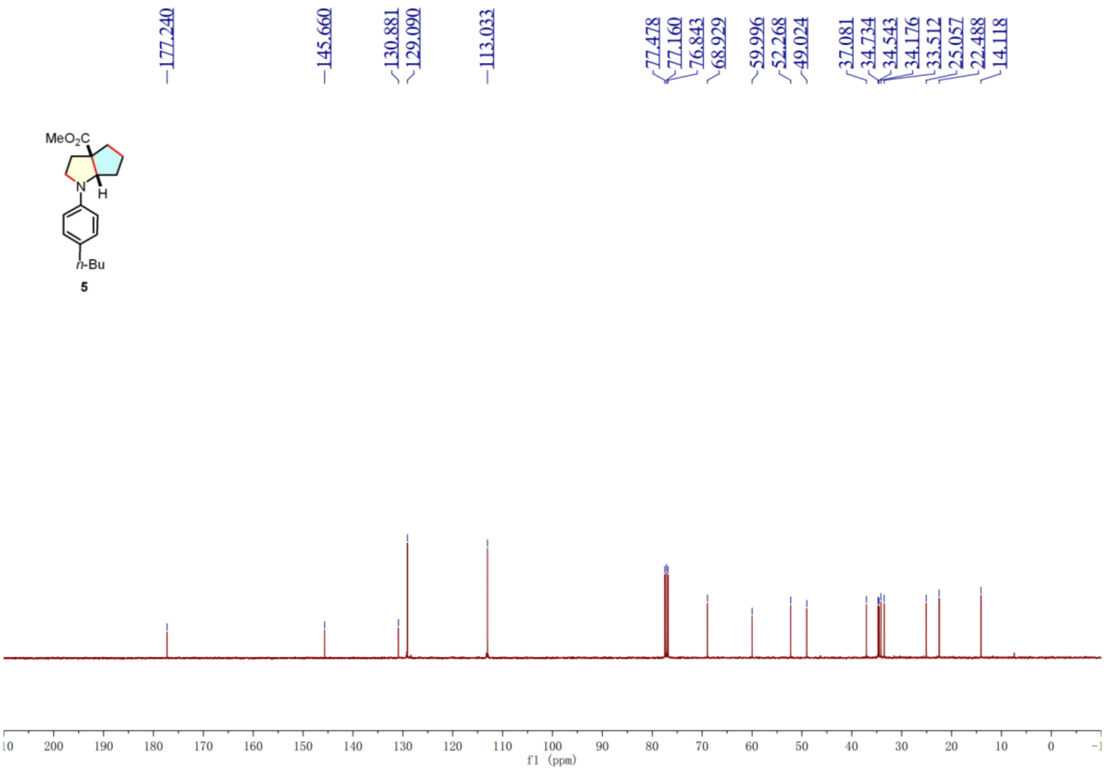

$^{13}C\{^{1}H\}$ NMR Spectrum of Compound **5** (100 MHz, $CDCl_3$)

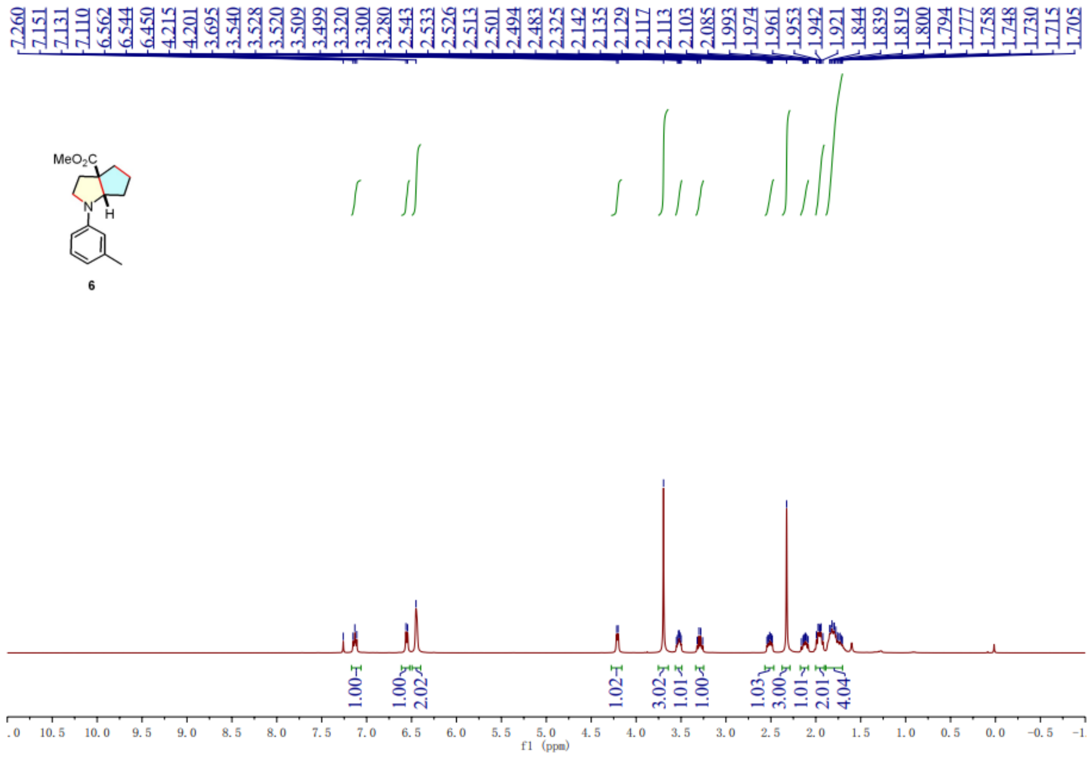

^{1}H NMR Spectrum of Compound **6** (400 MHz, $CDCl_3$)

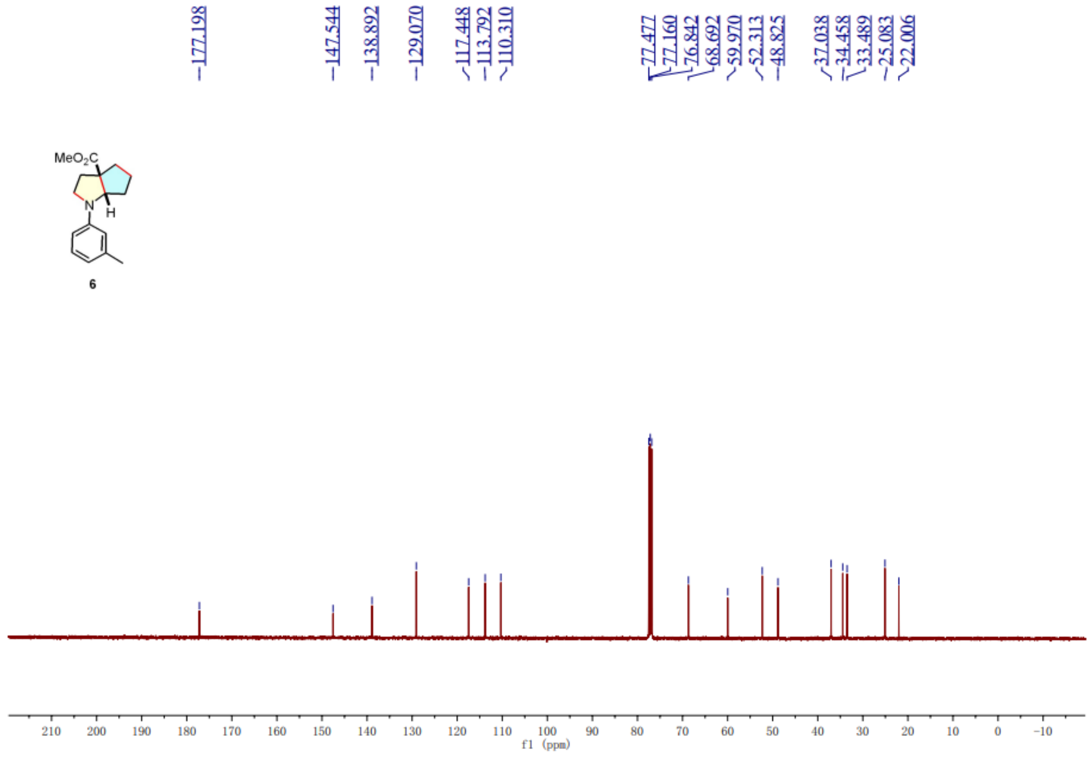

$^{13}C\{^1H\}$ NMR Spectrum of Compound **6** (100 MHz, $CDCl_3$)

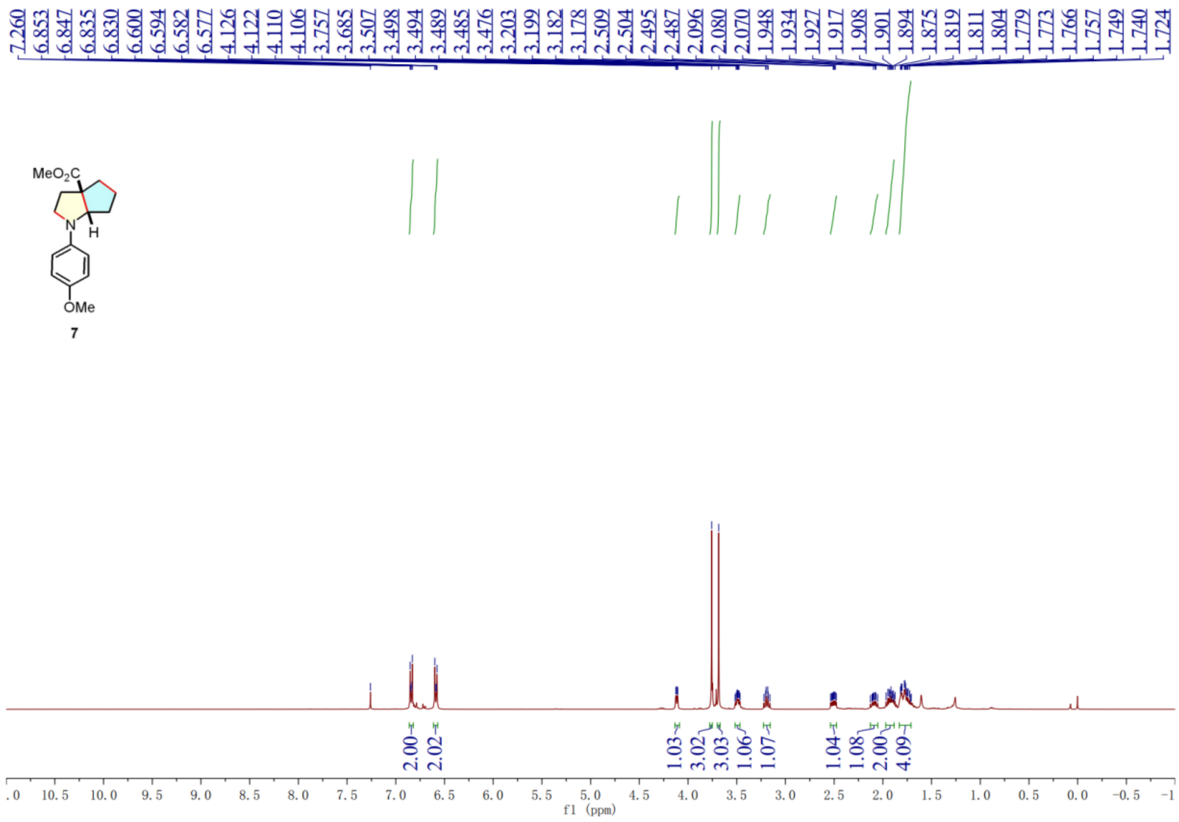

^1H NMR Spectrum of Compound **7** (400 MHz, $CDCl_3$)

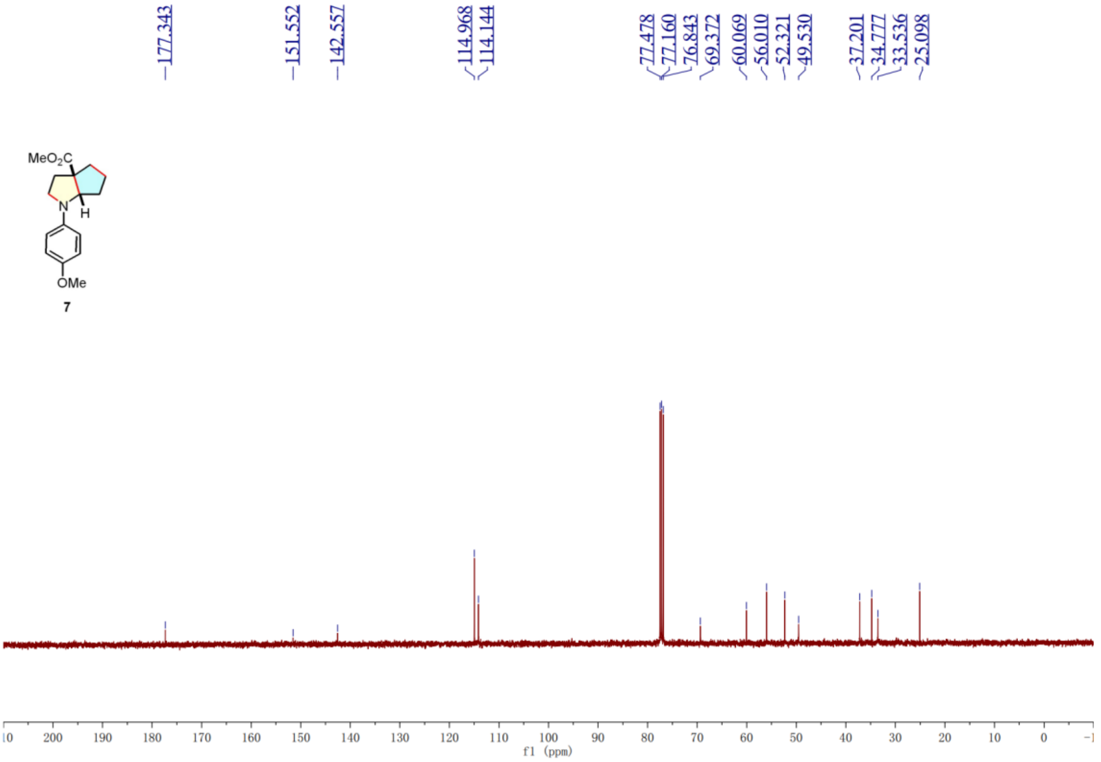

$^{13}C\{^{1}H\}$ NMR Spectrum of Compound **7** (100 MHz, $CDCl_3$)

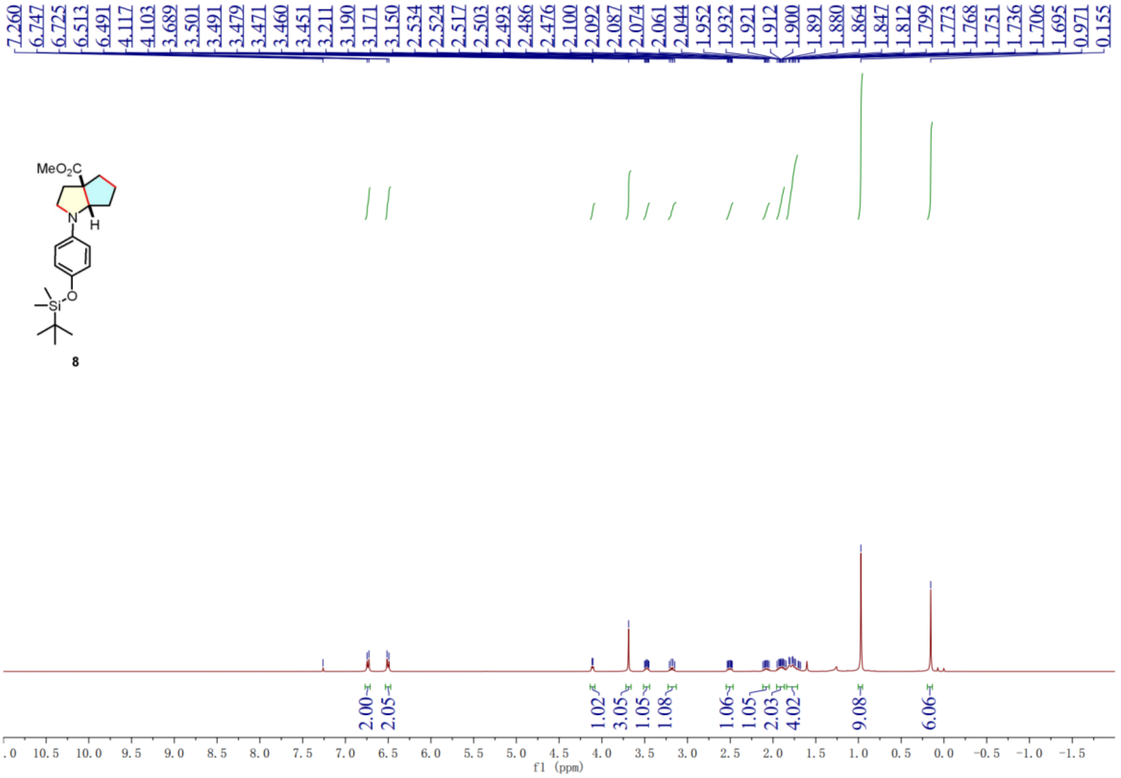

^{1}H NMR Spectrum of Compound **8** (400 MHz, $CDCl_3$)

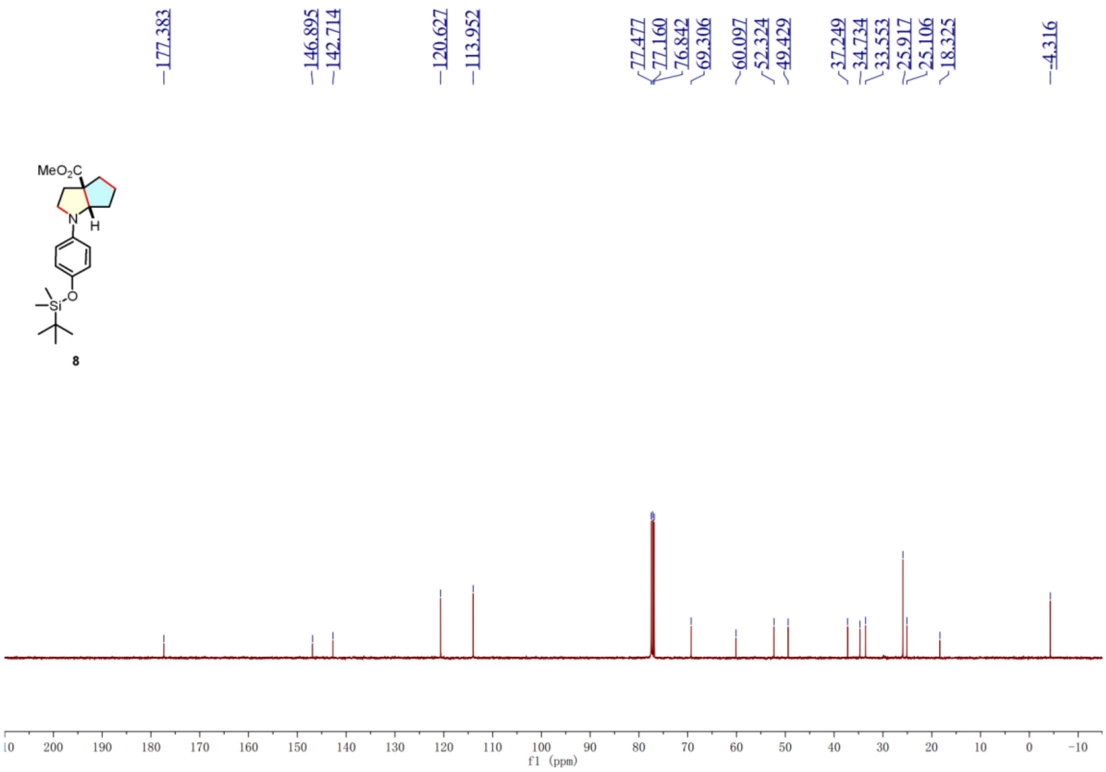

$^{13}C\{^{1}H\}$ NMR Spectrum of Compound **8** (100 MHz, $CDCl_3$)

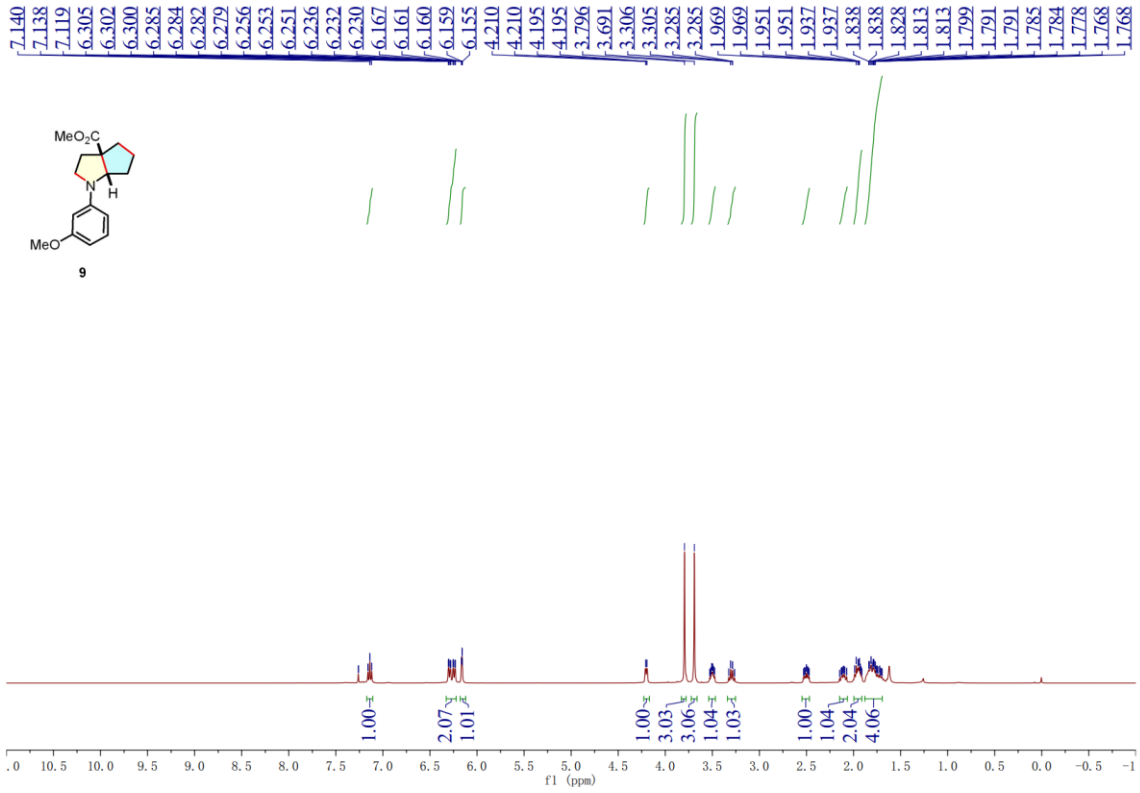

^{1}H NMR Spectrum of Compound **9** (400 MHz, $CDCl_3$)

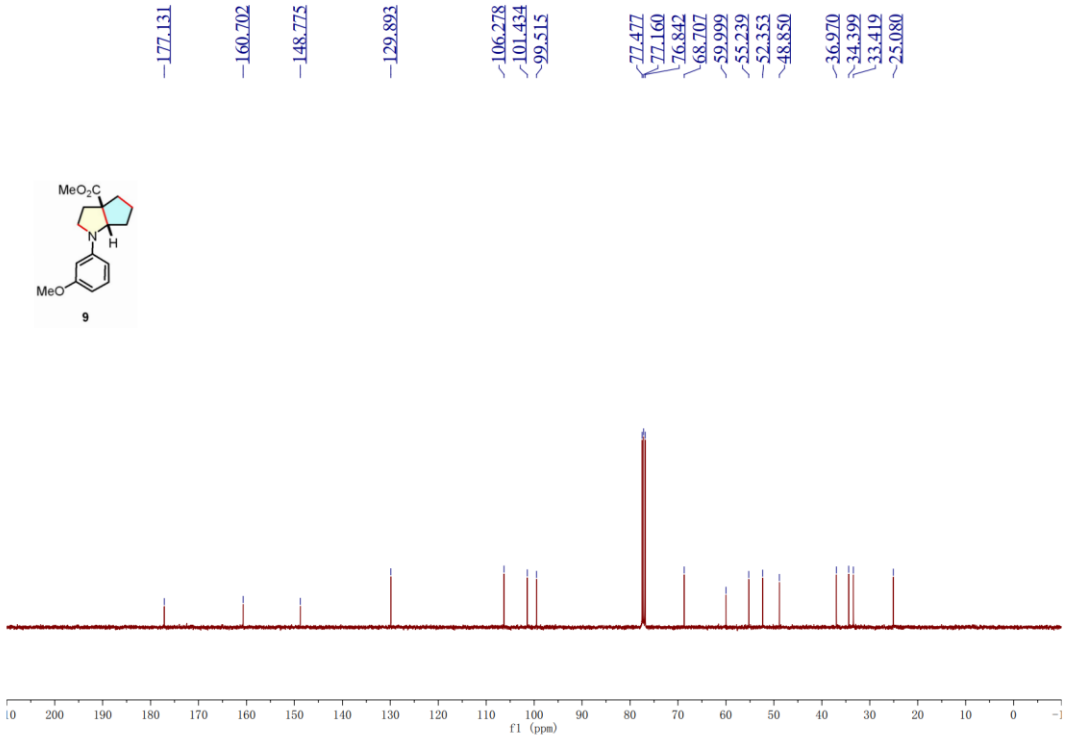

$^{13}C\{^{1}H\}$ NMR Spectrum of Compound **9** (100 MHz, $CDCl_3$)

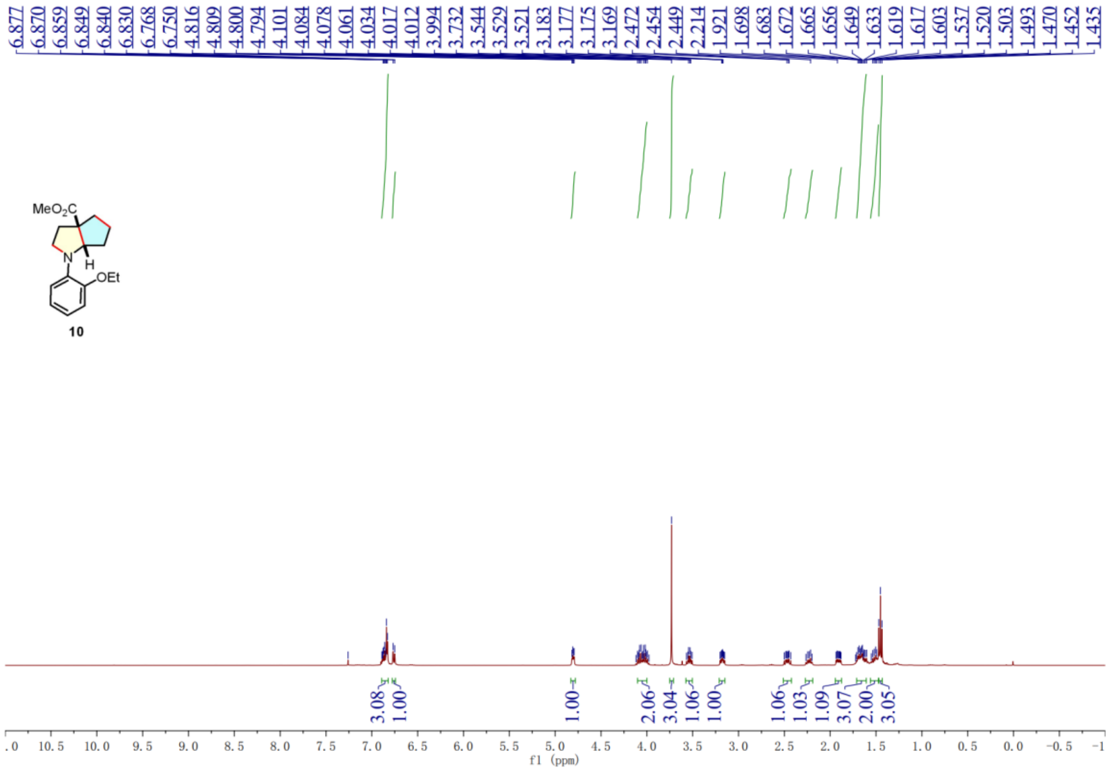

^{1}H NMR Spectrum of Compound **10** (400 MHz, $CDCl_3$)

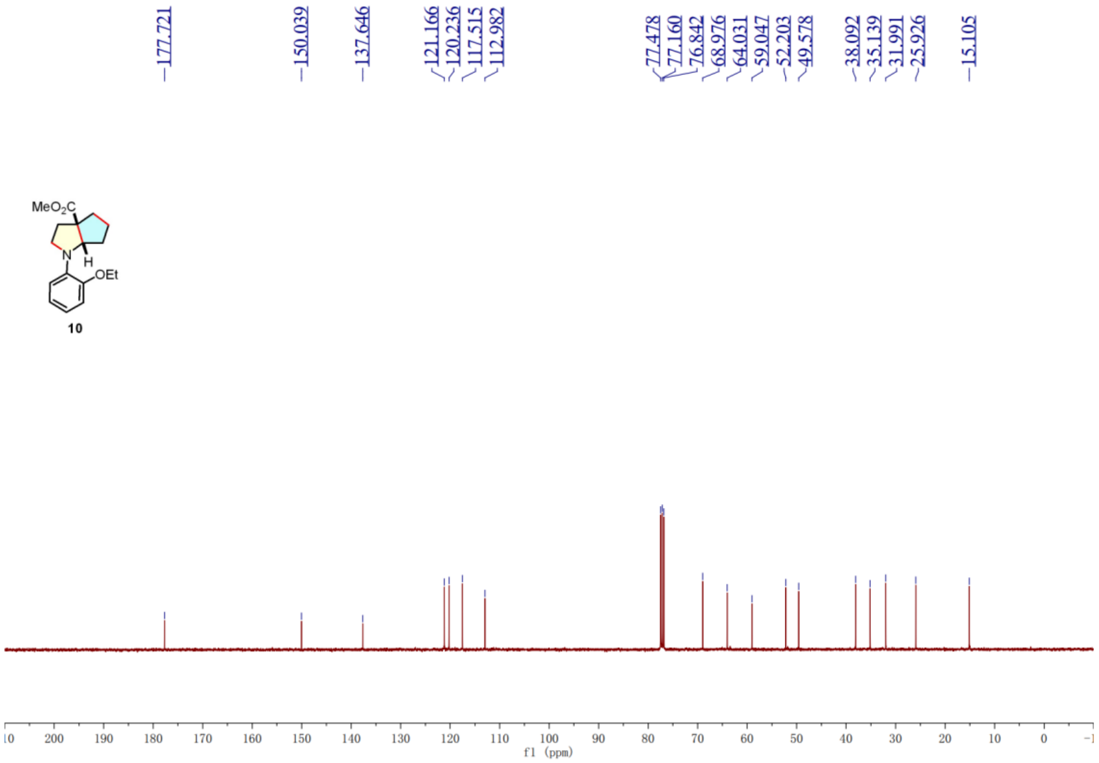

$^{13}C\{^{1}H\}$ NMR Spectrum of Compound **10** (100 MHz, $CDCl_3$)

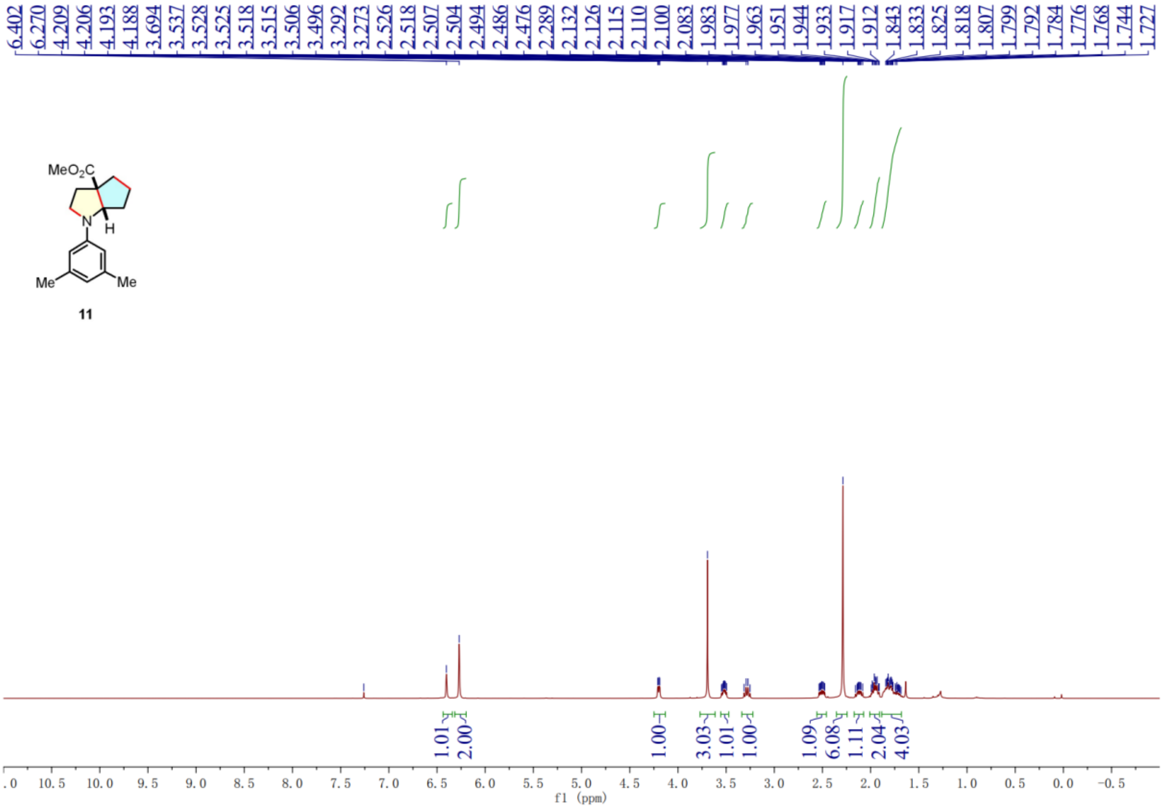

^{1}H NMR Spectrum of Compound **11** (400 MHz, $CDCl_3$)

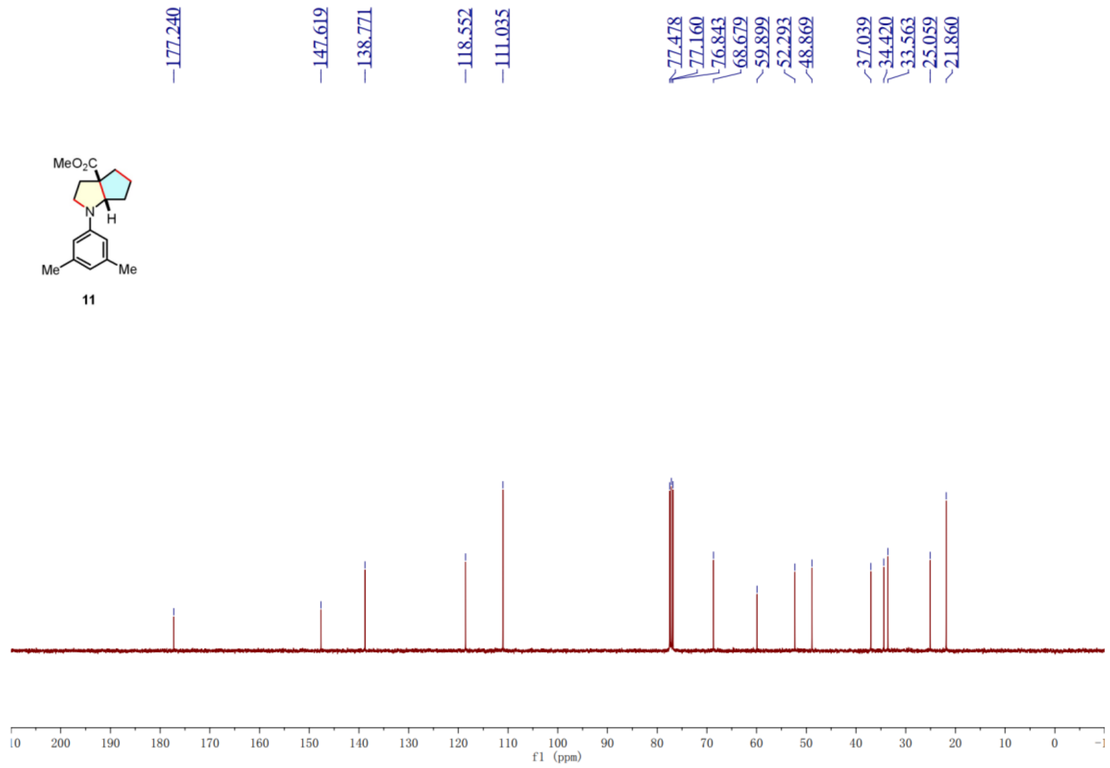

$^{13}C\{^{1}H\}$ NMR Spectrum of Compound **11** (100 MHz, $CDCl_3$)

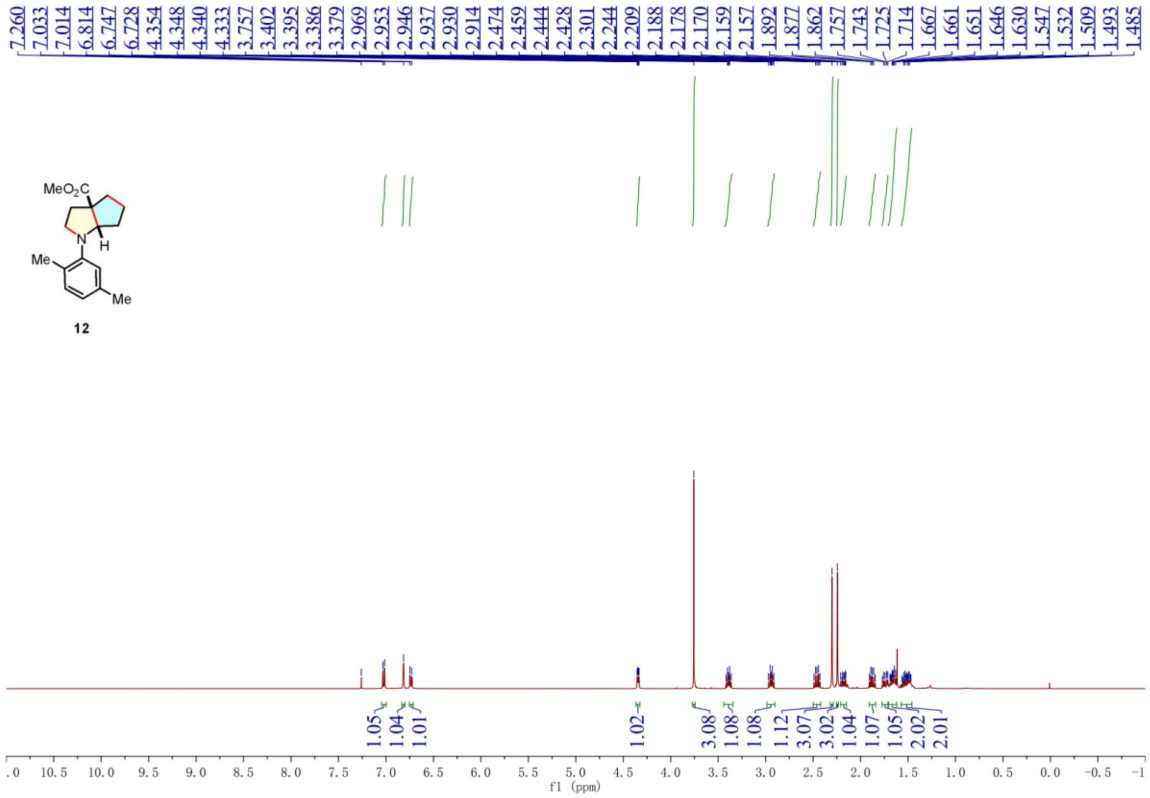

^{1}H NMR Spectrum of Compound **12** (400 MHz, $CDCl_3$)

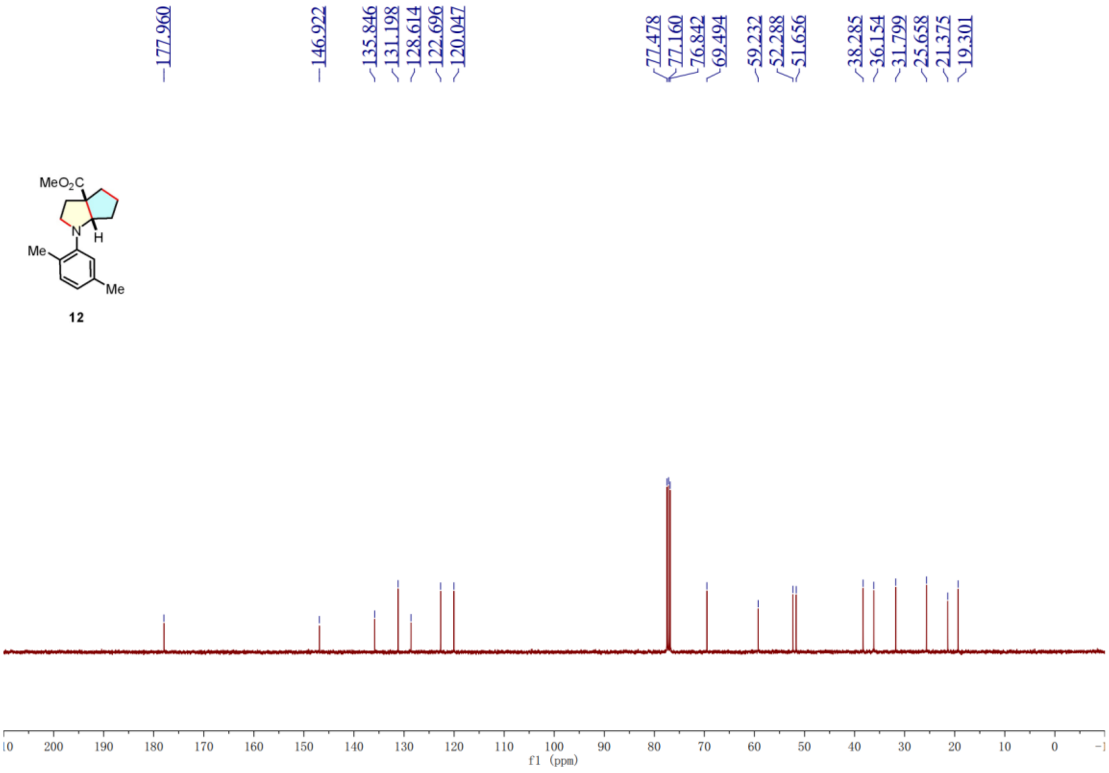

$^{13}C\{^{1}H\}$ NMR Spectrum of Compound **12** (100 MHz, $CDCl_3$)

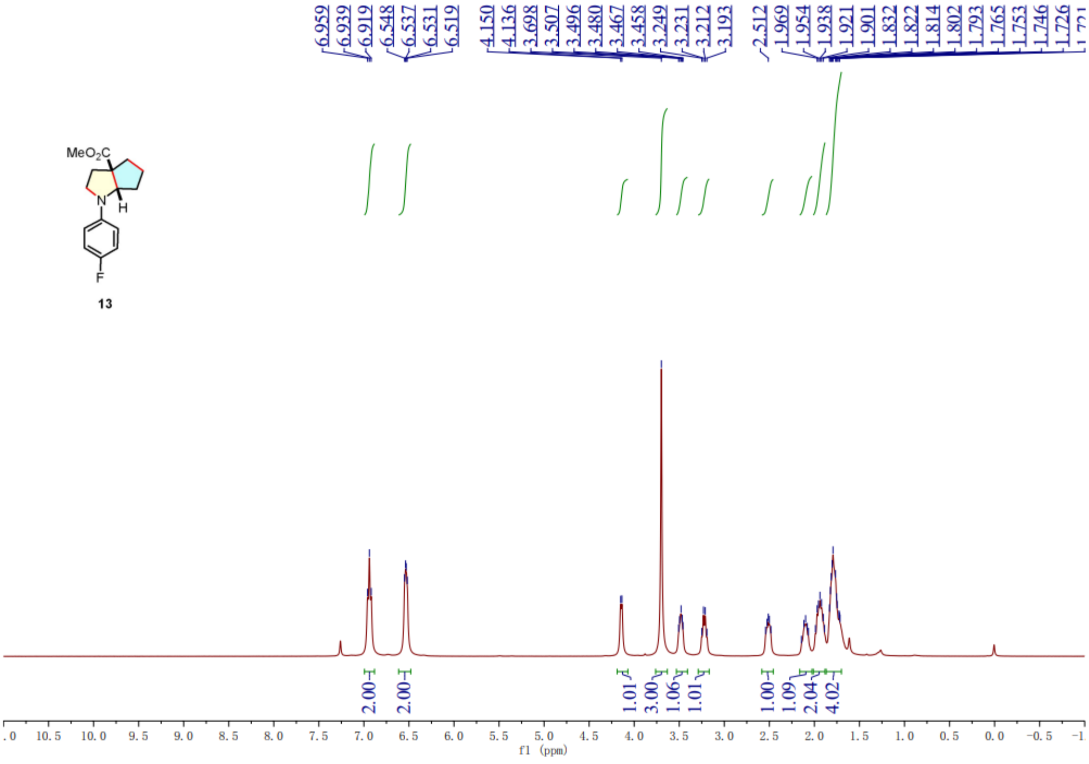

^{1}H NMR Spectrum of Compound **13** (400 MHz, $CDCl_3$)

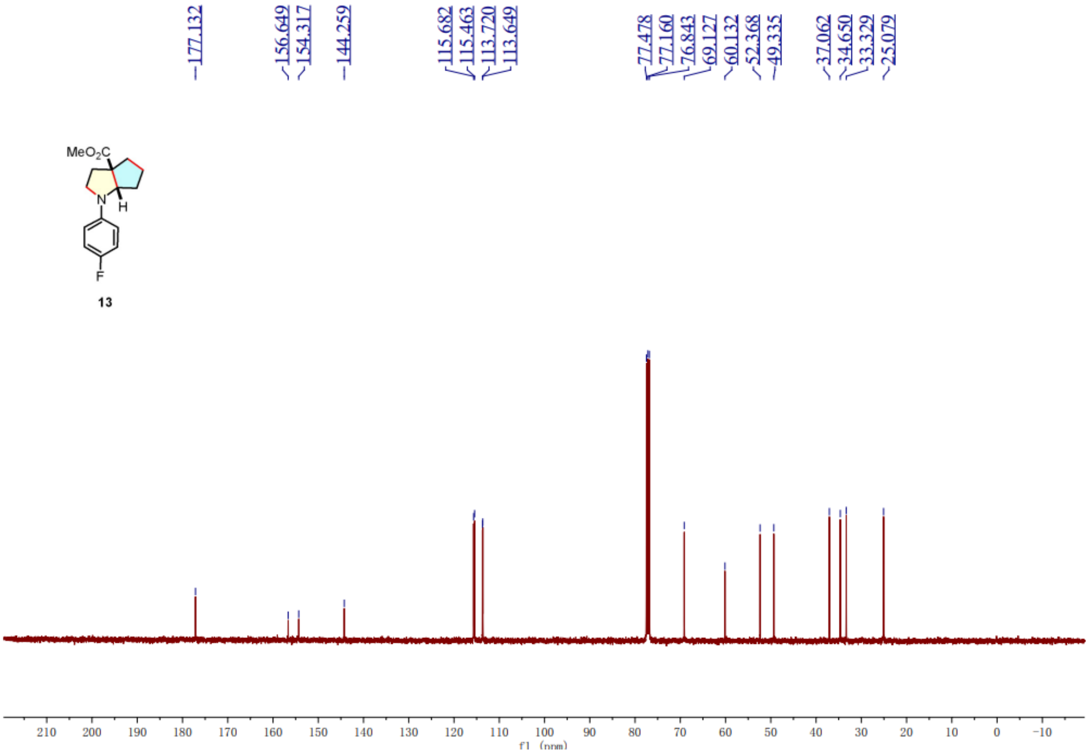

$^{13}C\{^{1}H\}$ NMR Spectrum of Compound **13** (100 MHz, $CDCl_3$)

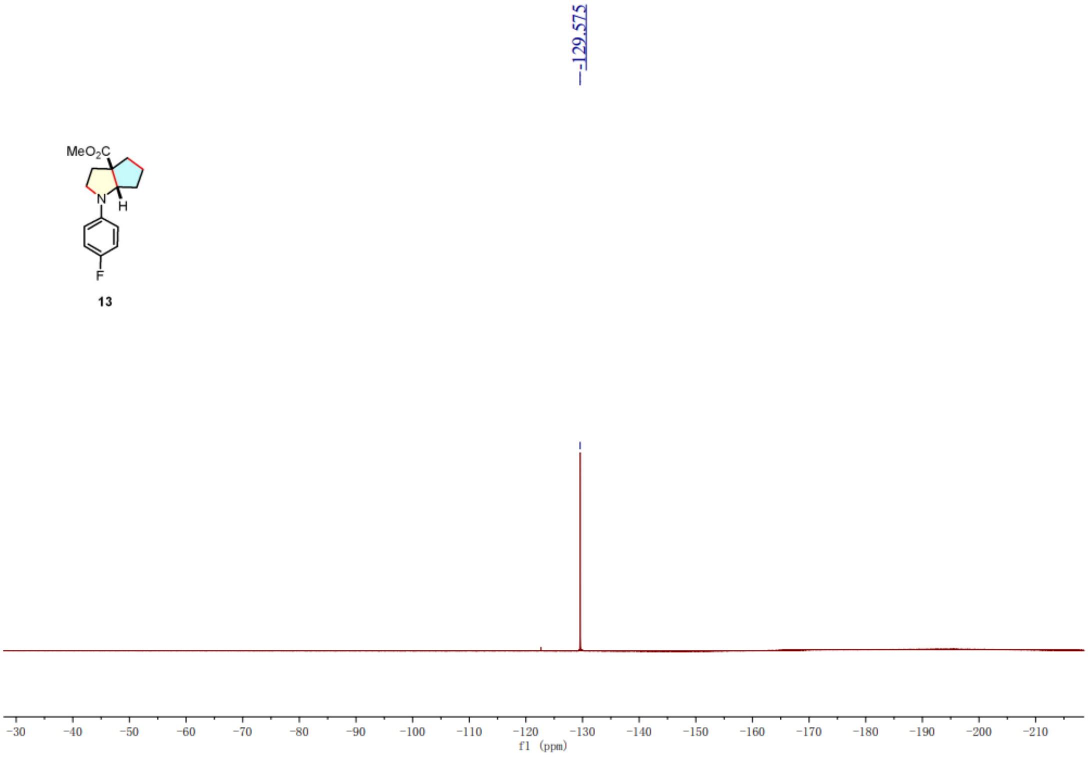

$^{19}F\{^{1}H\}$ NMR Spectrum of Compound **13** (376 MHz, $CDCl_3$)

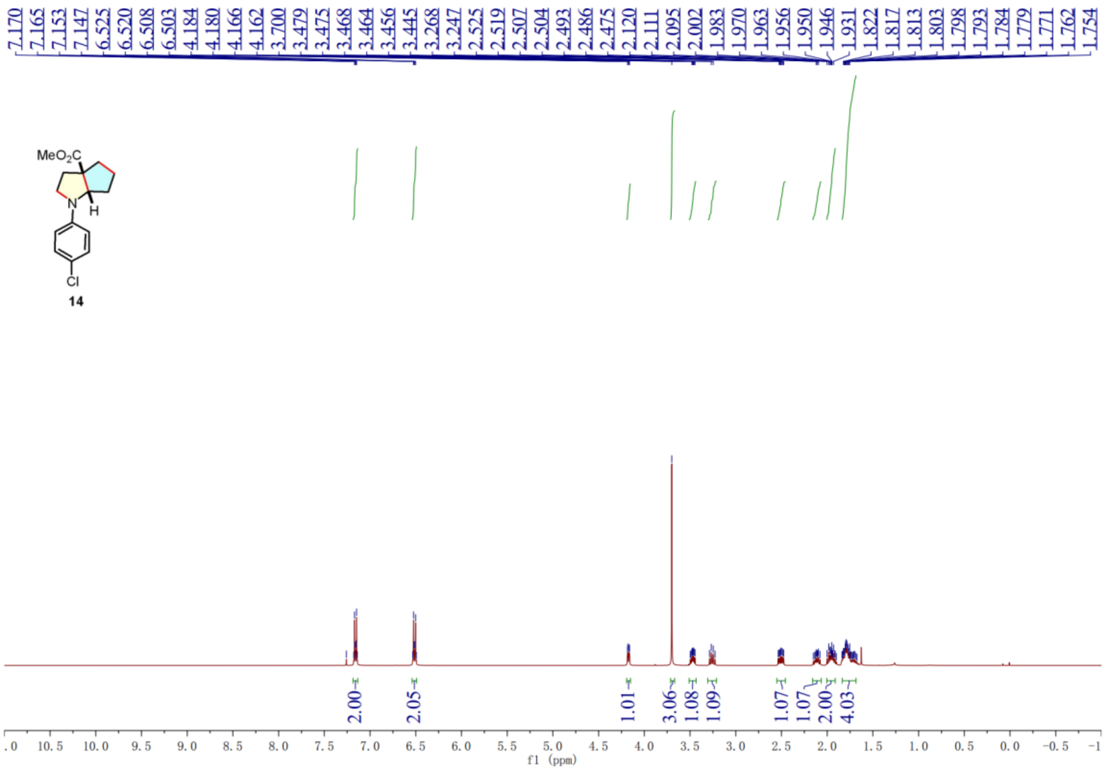

^{1}H NMR Spectrum of Compound **14** (400 MHz, $CDCl_3$)

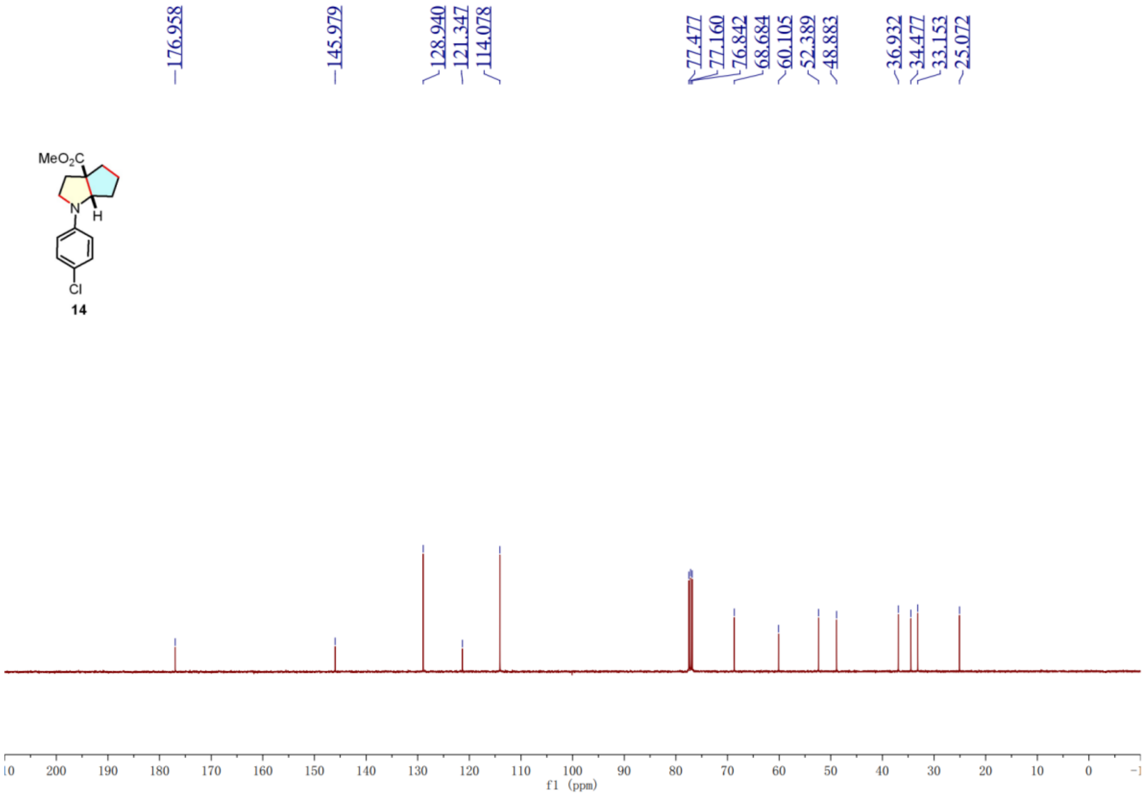

^{13}C{^{1}H} NMR Spectrum of Compound **14** (100 MHz, $CDCl_3$)

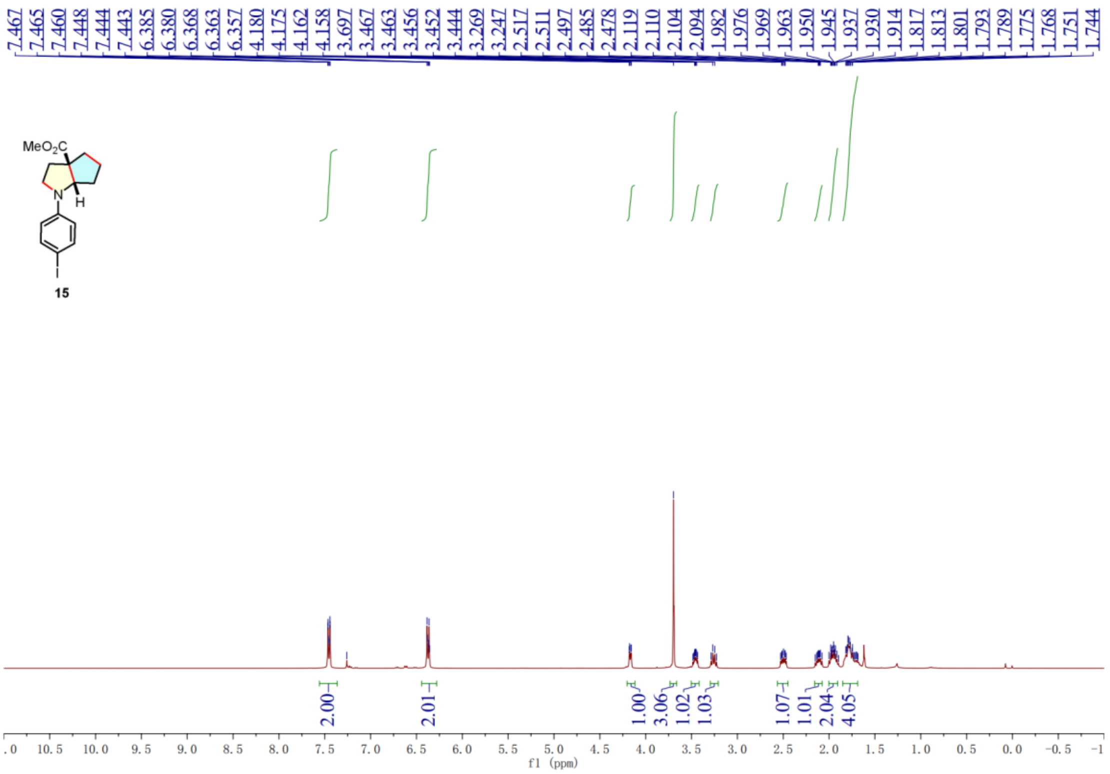

^{1}H NMR Spectrum of Compound **15** (400 MHz, $CDCl_3$)

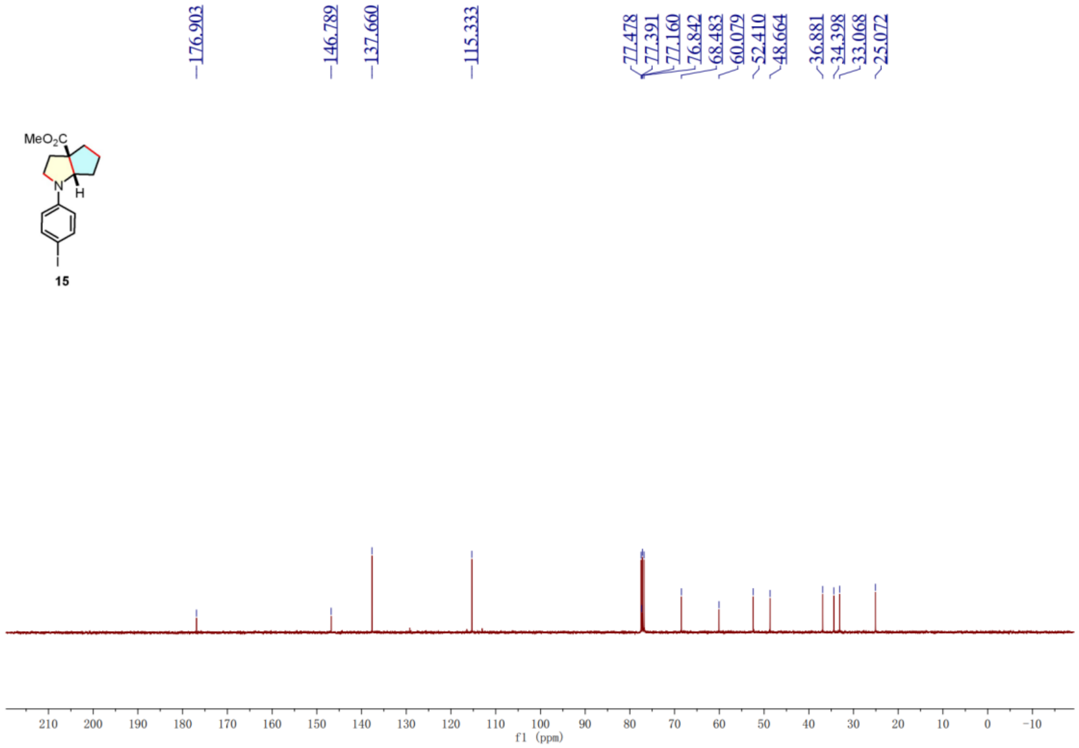

$^{13}C\{^{1}H\}$ NMR Spectrum of Compound **15** (100 MHz, $CDCl_3$)

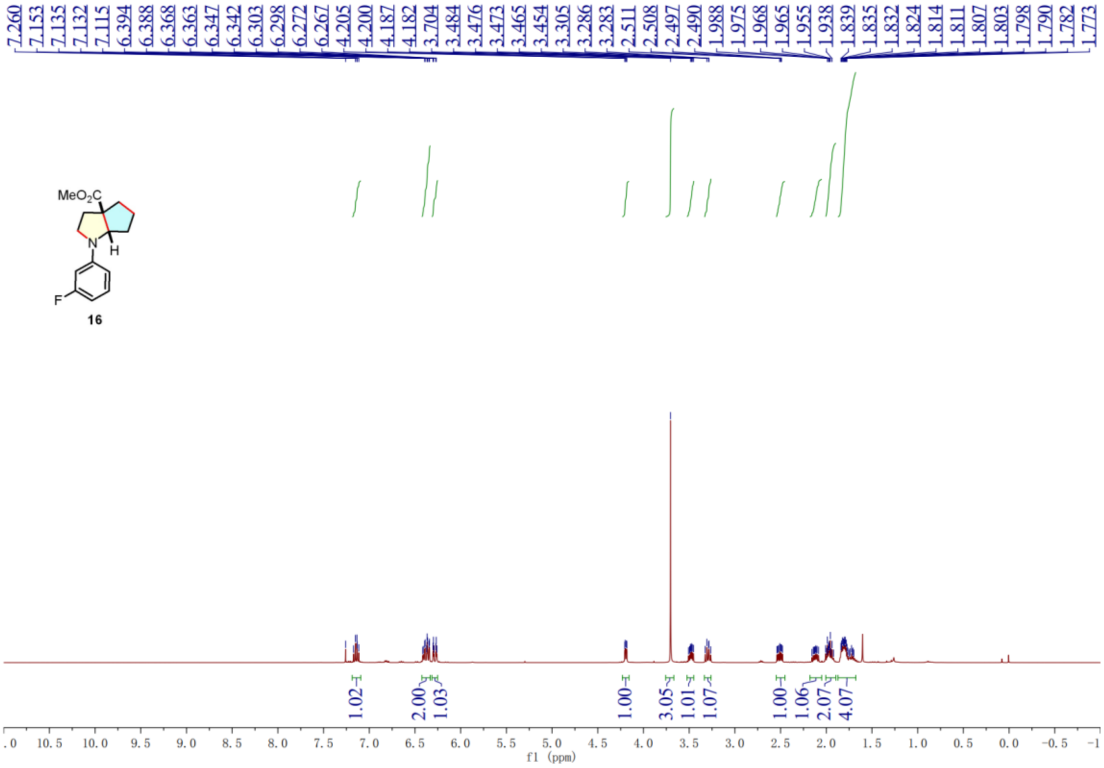

^{1}H NMR Spectrum of Compound **16** (400 MHz, $CDCl_3$)

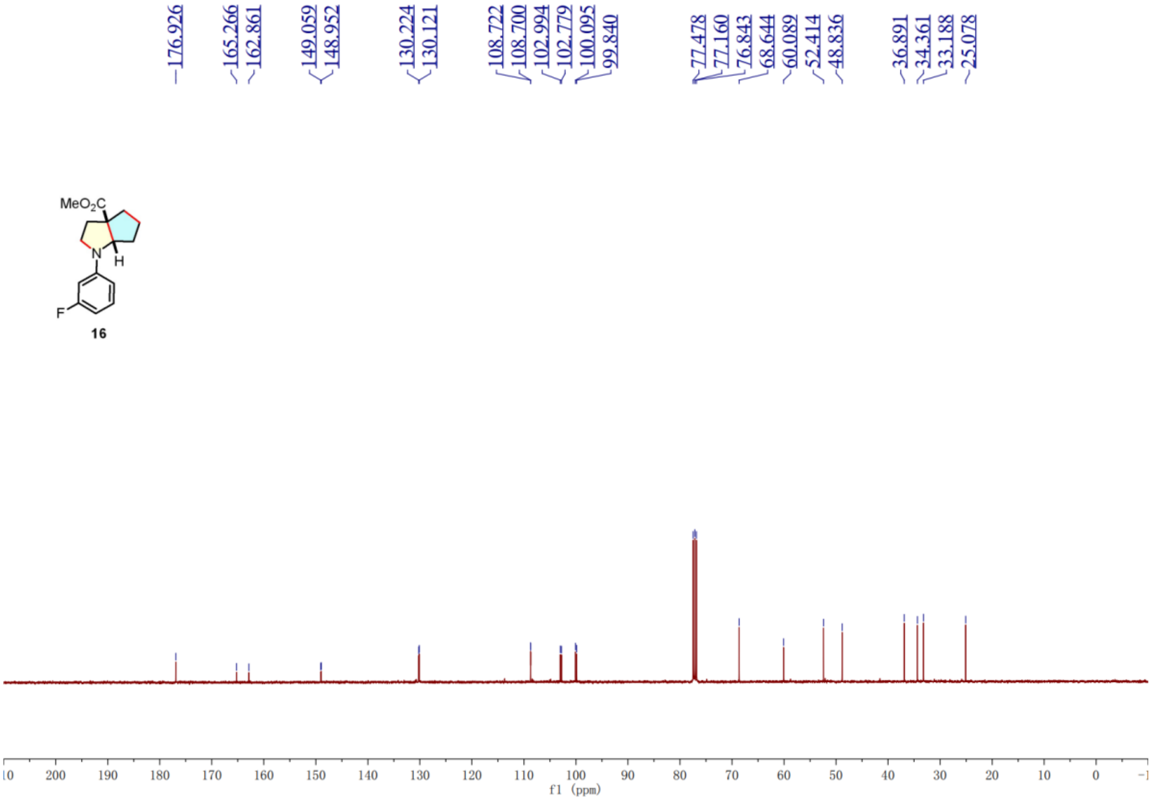

^{13}C{^{1}H} NMR Spectrum of Compound **16** (100 MHz, $CDCl_3$)

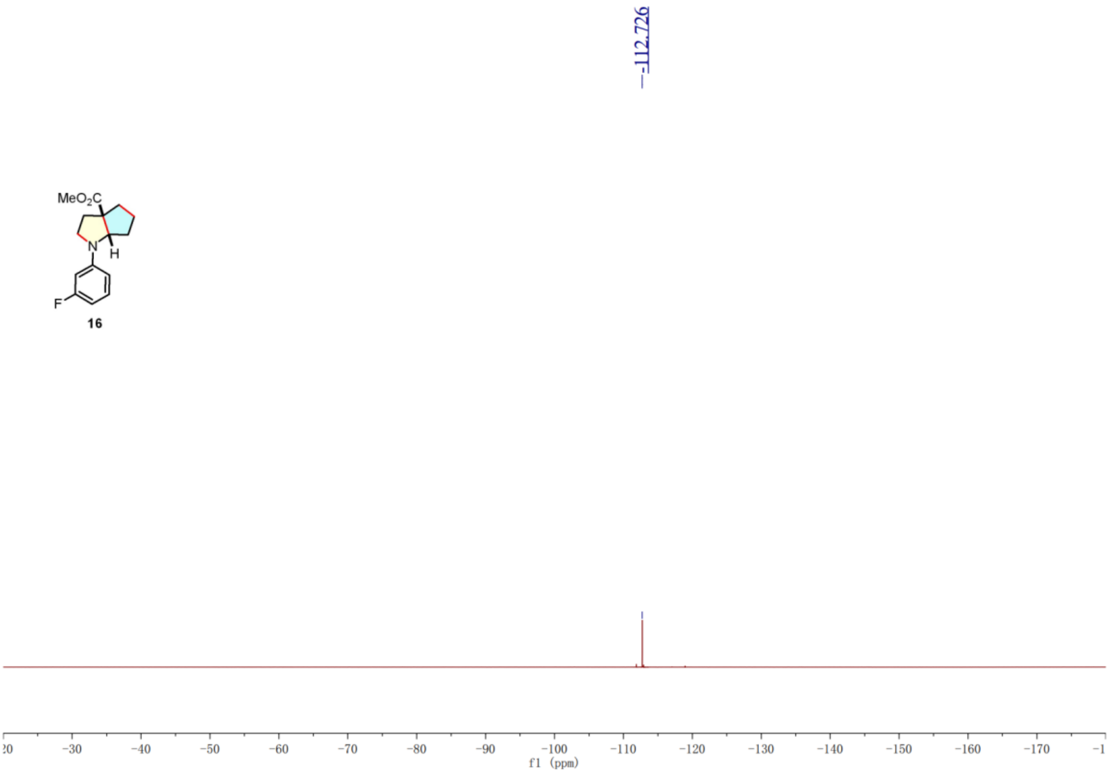

$^{19}F\{^{1}H\}$ NMR Spectrum of Compound **16** (376 MHz, $CDCl_3$)

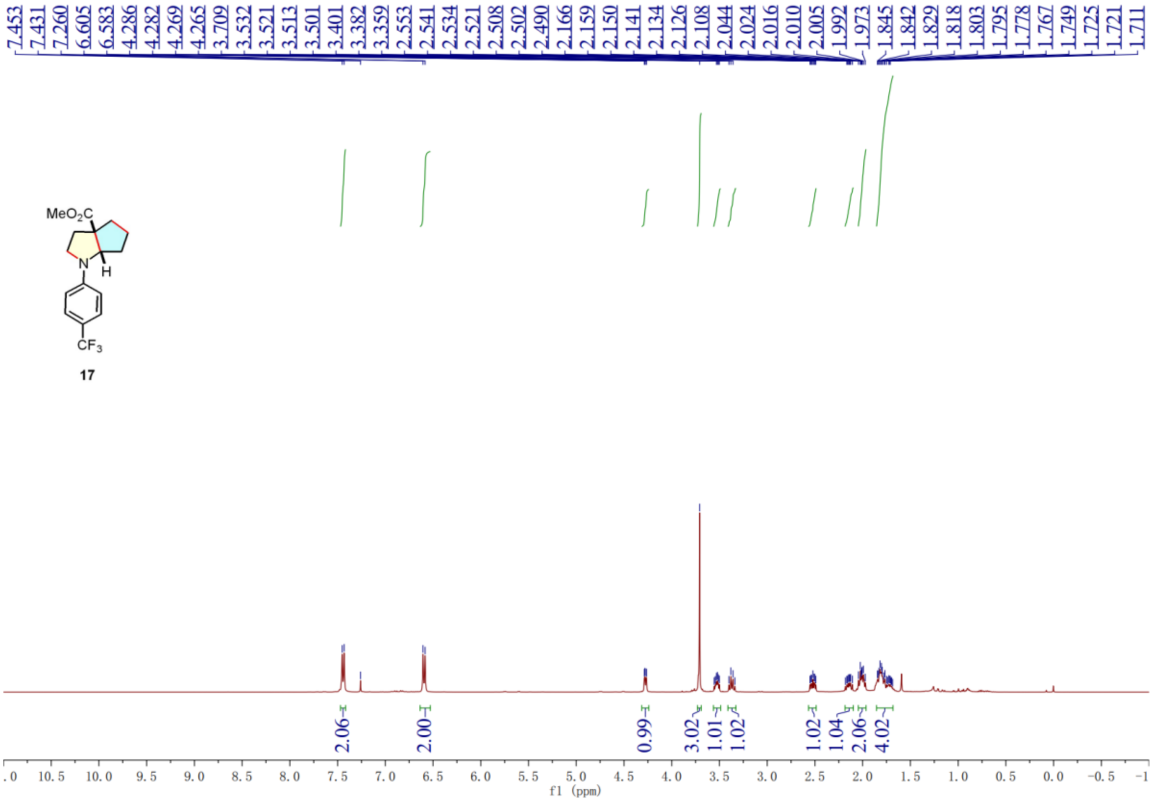

^{1}H NMR Spectrum of Compound **17** (400 MHz, $CDCl_3$)

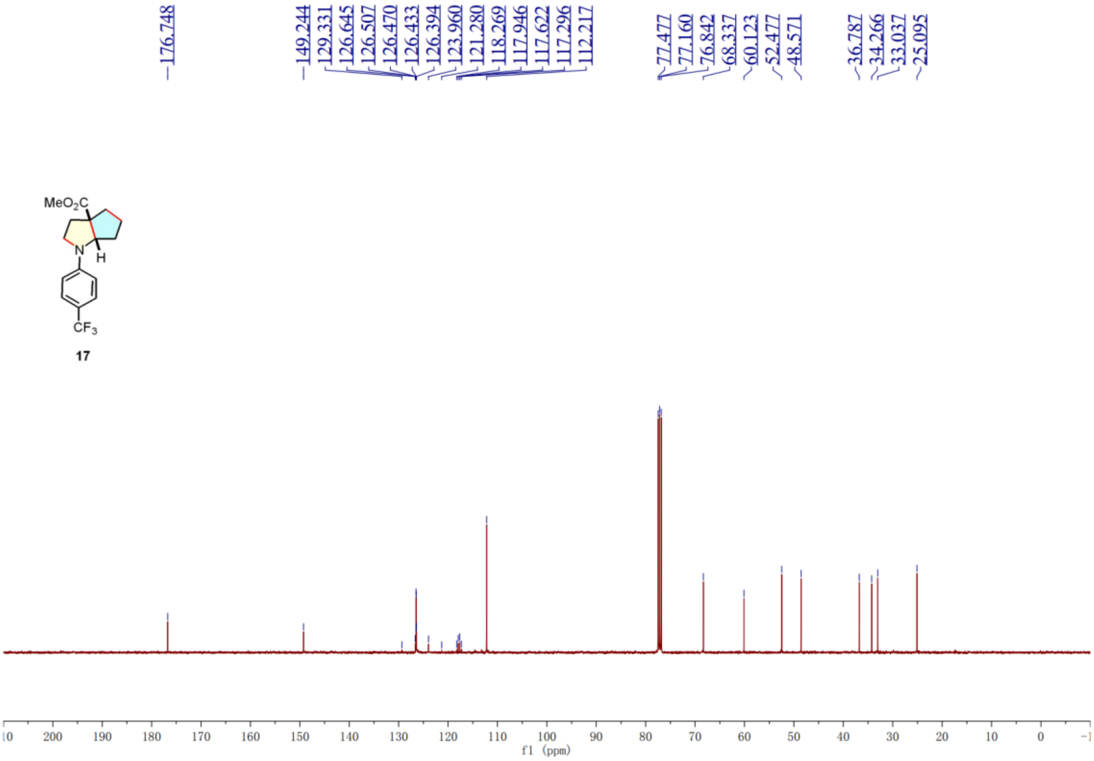

$^{13}C\{^1H\}$ NMR Spectrum of Compound **17** (100 MHz, $CDCl_3$)

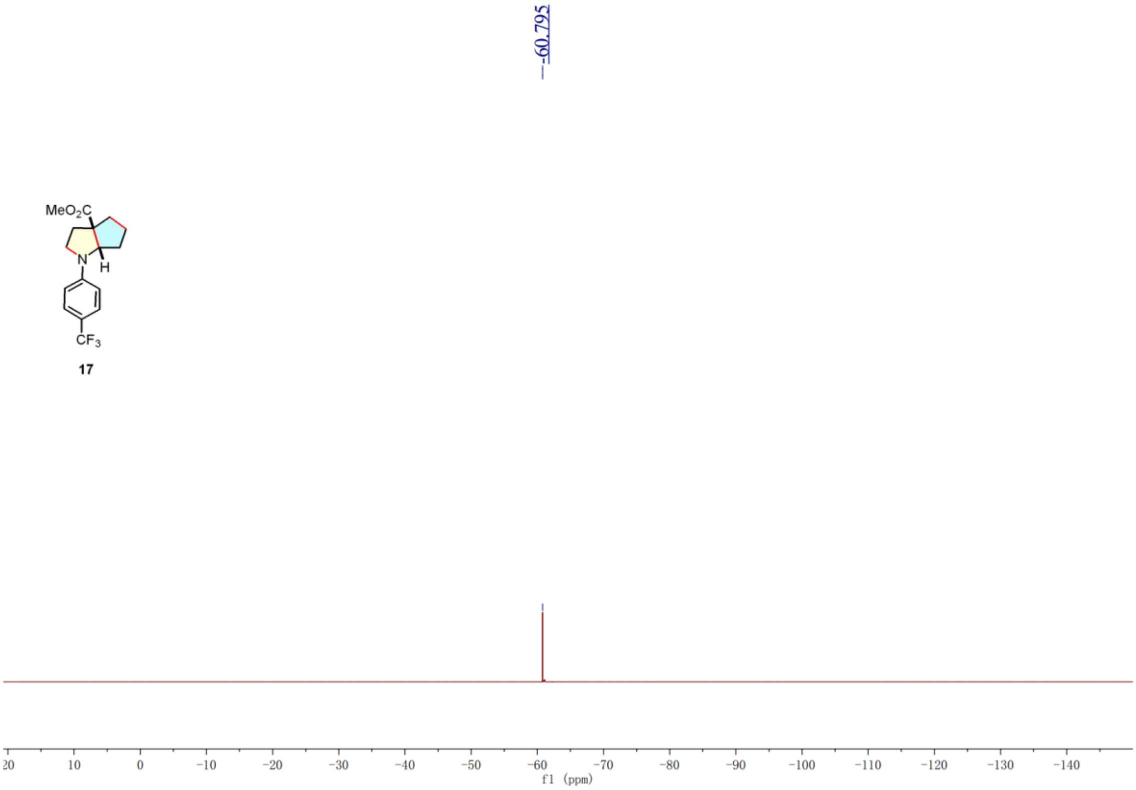

$^{19}F\{^1H\}$ NMR Spectrum of Compound **17** (376 MHz, $CDCl_3$)

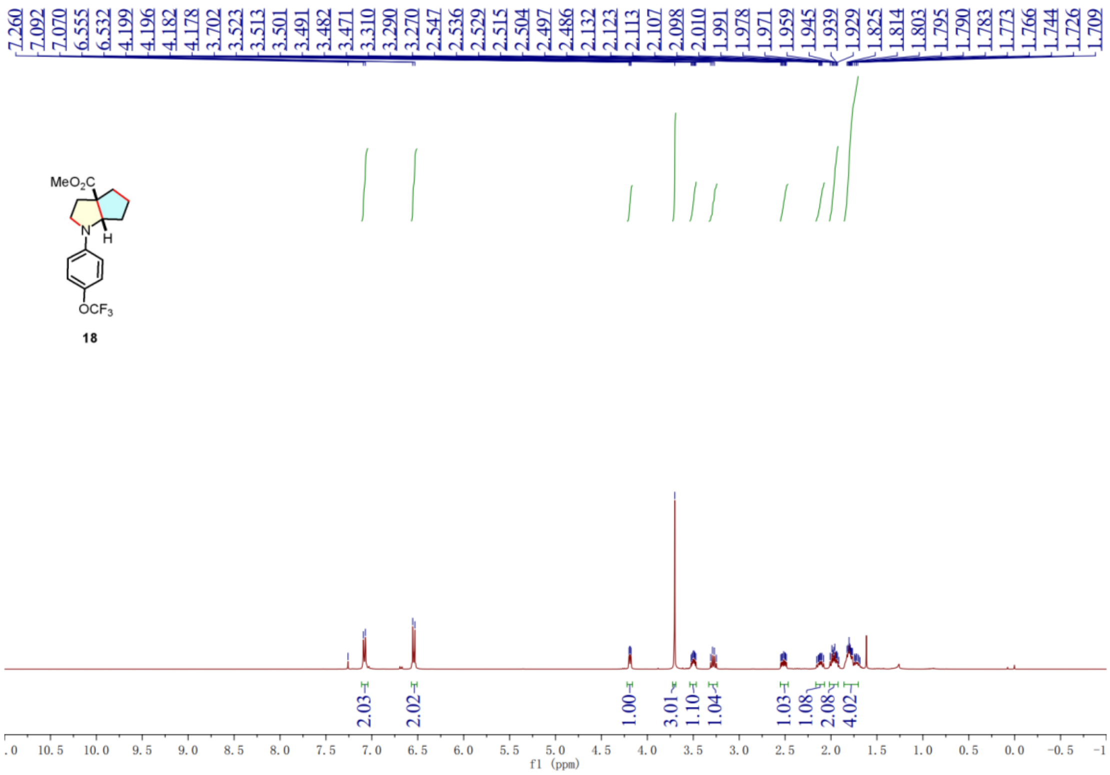

^{1}H NMR Spectrum of Compound **18** (400 MHz, $CDCl_3$)

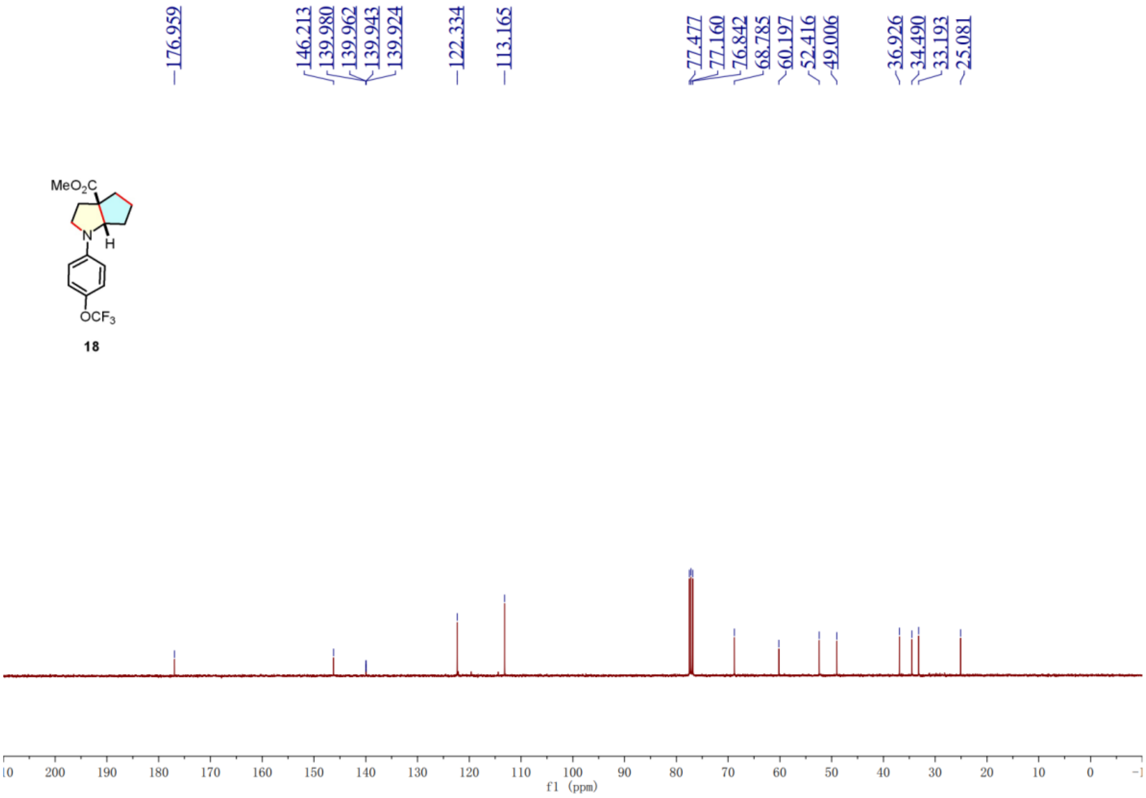

$^{13}C\{^1H\}$ NMR Spectrum of Compound **18** (100 MHz, $CDCl_3$)

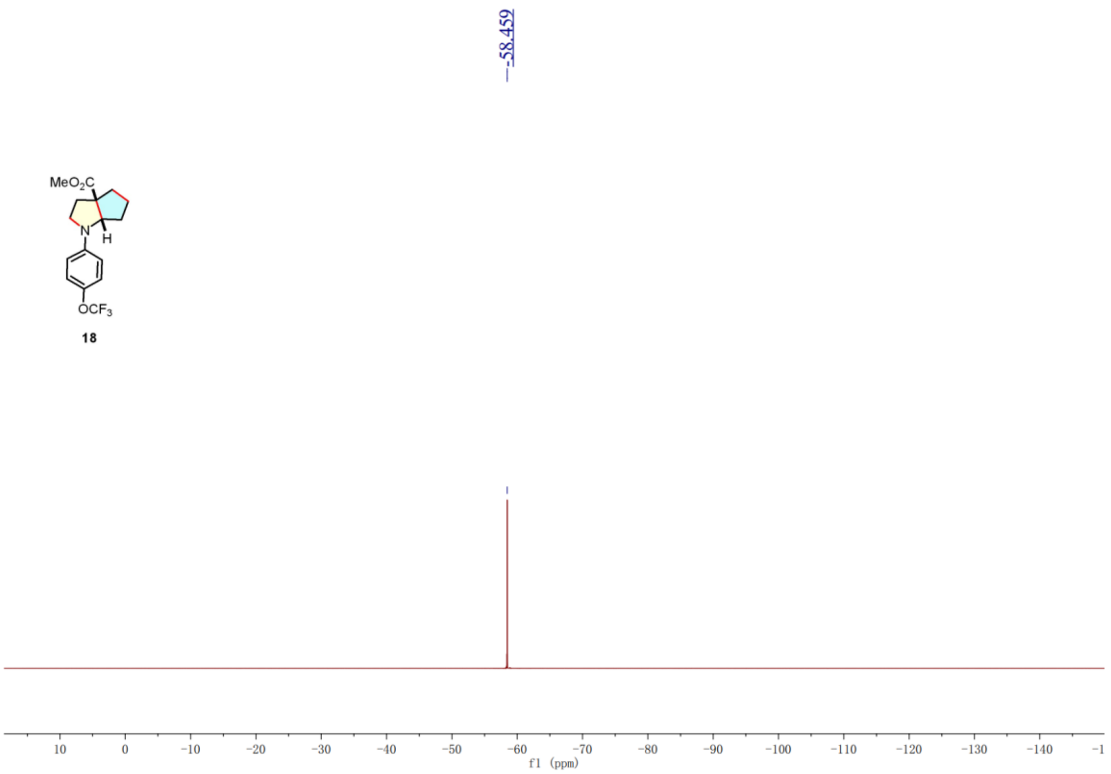

$^{19}F\{^{1}H\}$ NMR Spectrum of Compound **18** (376 MHz, $CDCl_3$)

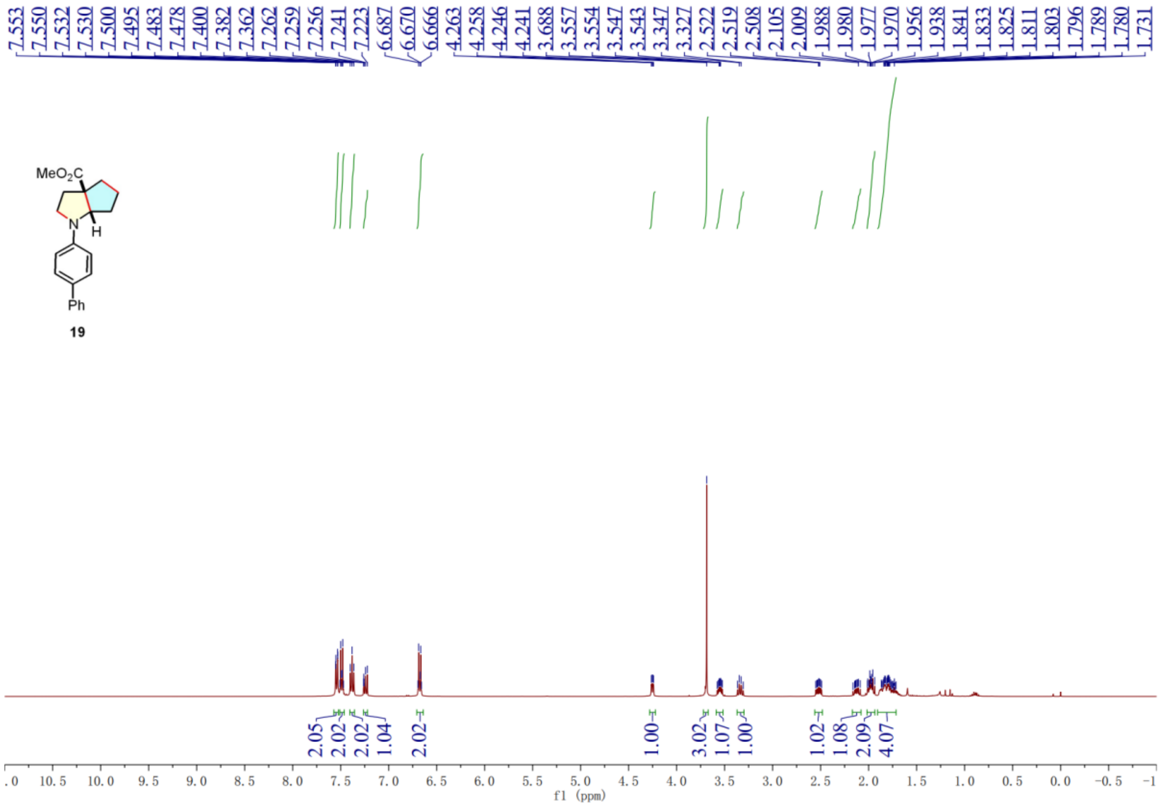

^{1}H NMR Spectrum of Compound **19** (400 MHz, $CDCl_3$)

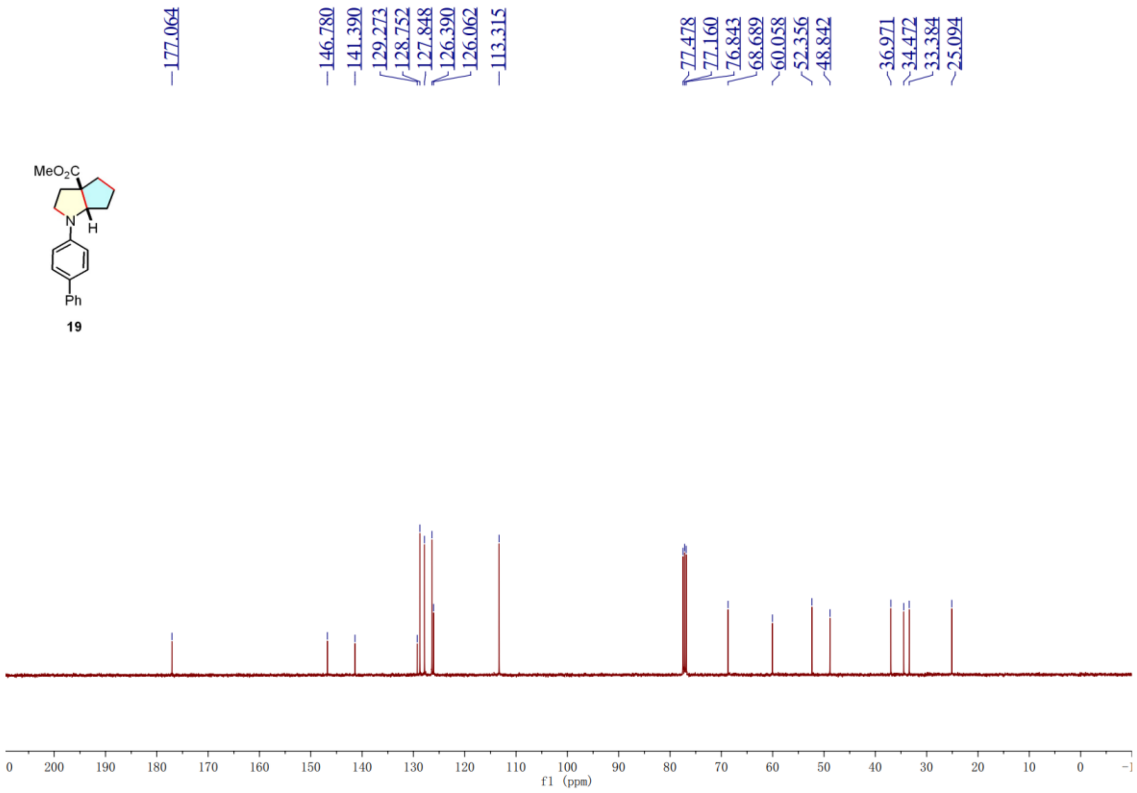

$^{13}C\{^{1}H\}$ NMR Spectrum of Compound **19** (100 MHz, $CDCl_3$)

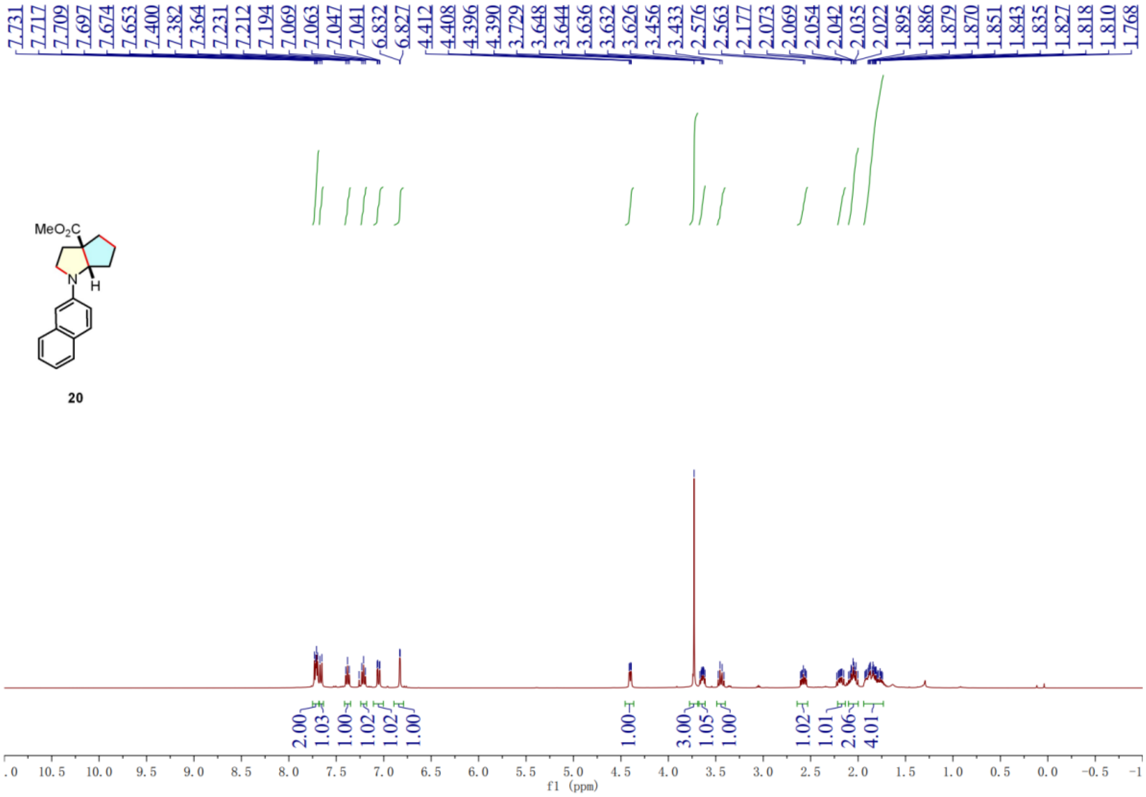

^{1}H NMR Spectrum of Compound **20** (400 MHz, $CDCl_3$)

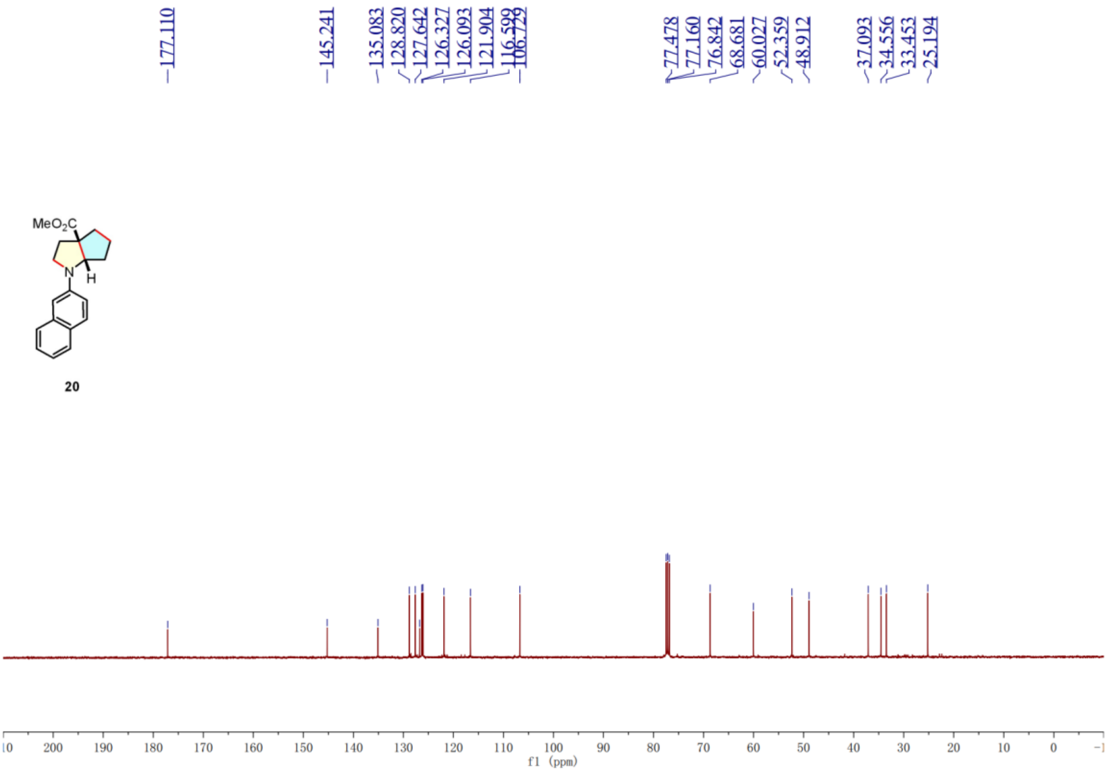

$^{13}C\{^1H\}$ NMR Spectrum of Compound **20** (100 MHz, $CDCl_3$)

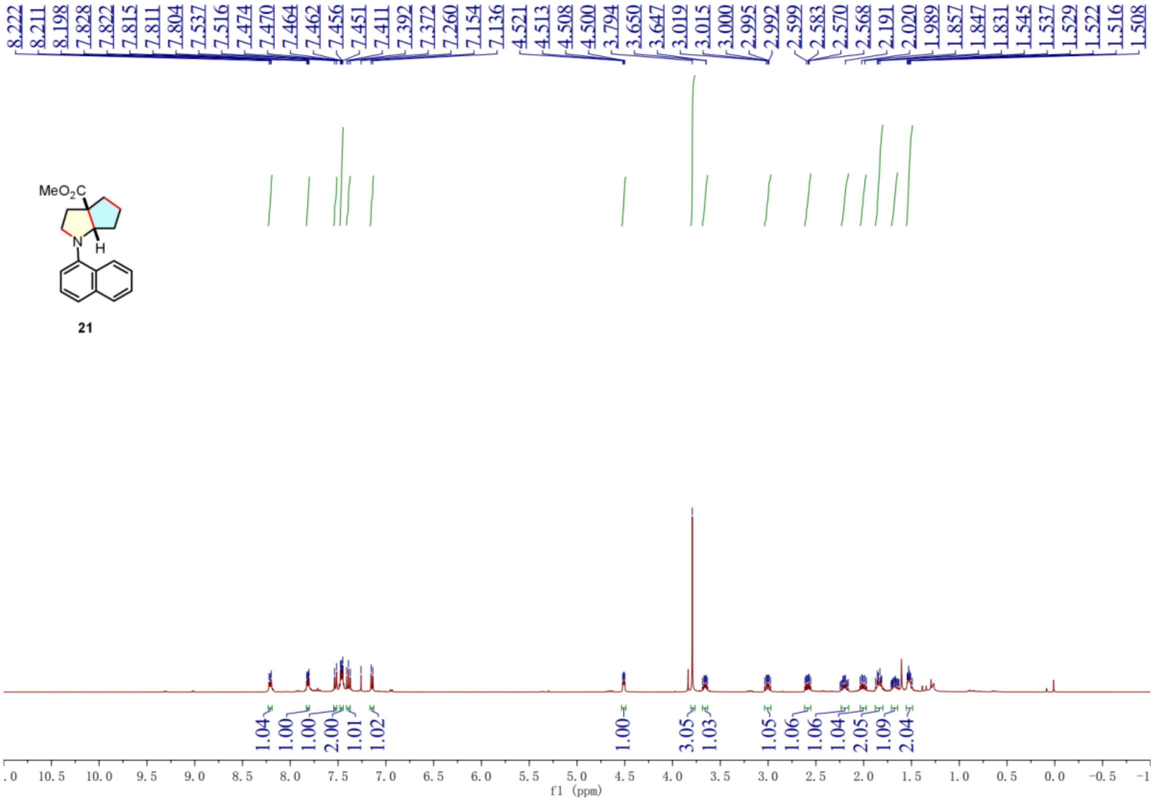

^1H NMR Spectrum of Compound **21** (400 MHz, $CDCl_3$)

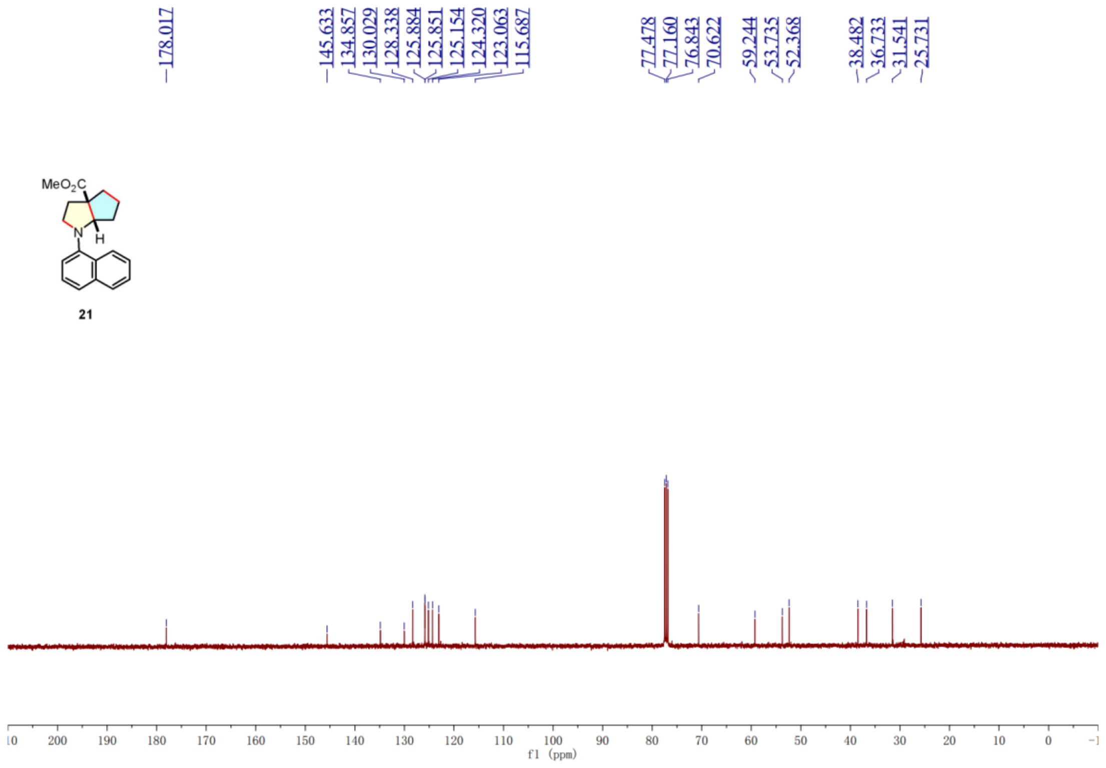

$^{13}C\{^1H\}$ NMR Spectrum of Compound **21** (100 MHz, $CDCl_3$)

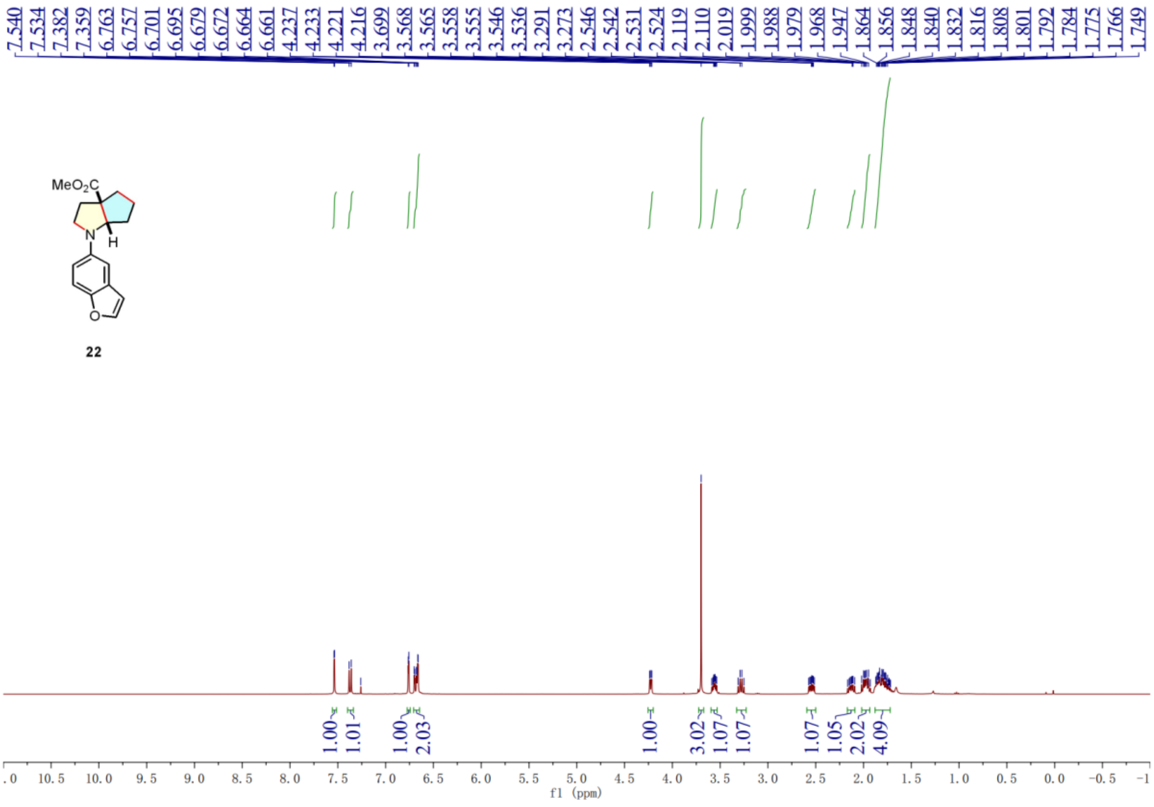

^1H NMR Spectrum of Compound **22** (400 MHz, $CDCl_3$)

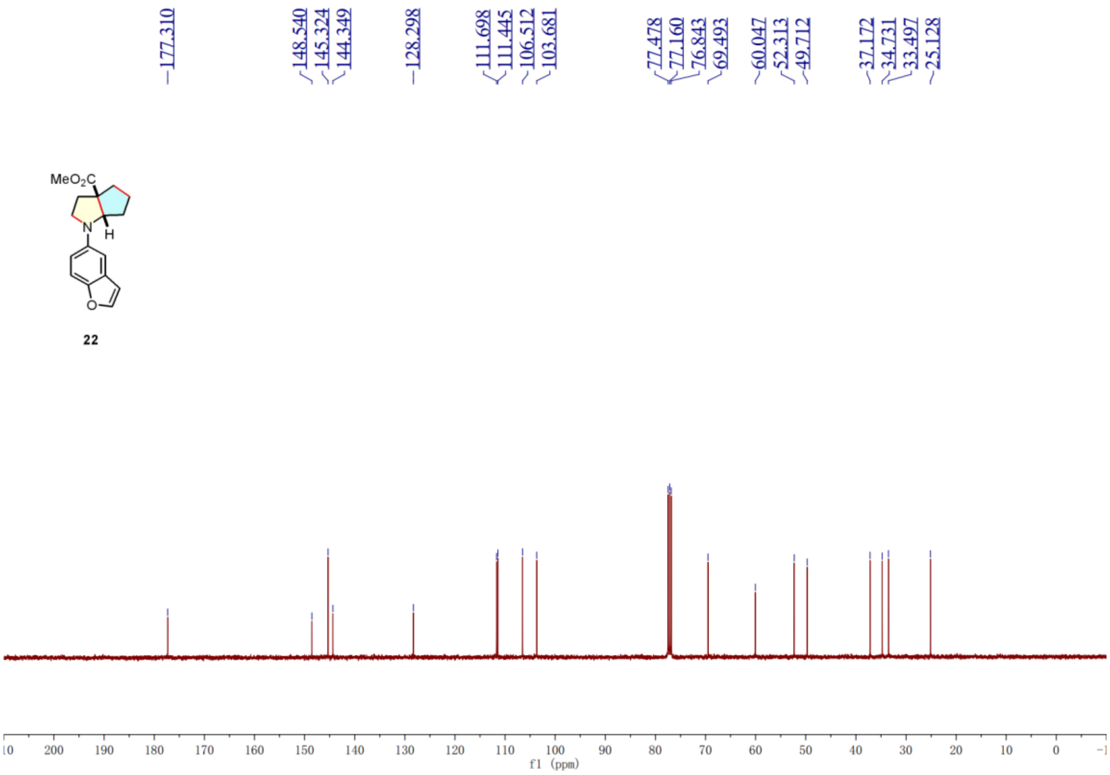

$^{13}C\{^{1}H\}$ NMR Spectrum of Compound **22** (100 MHz, $CDCl_3$)

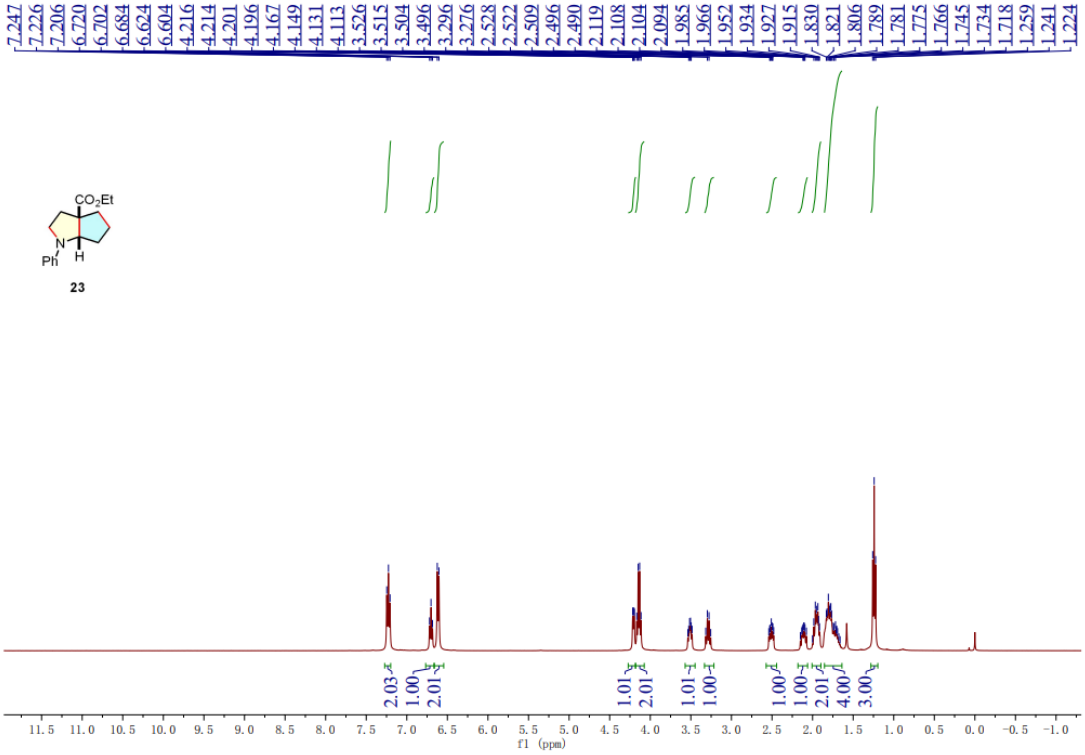

^{1}H NMR Spectrum of Compound **23** (400 MHz, $CDCl_3$)

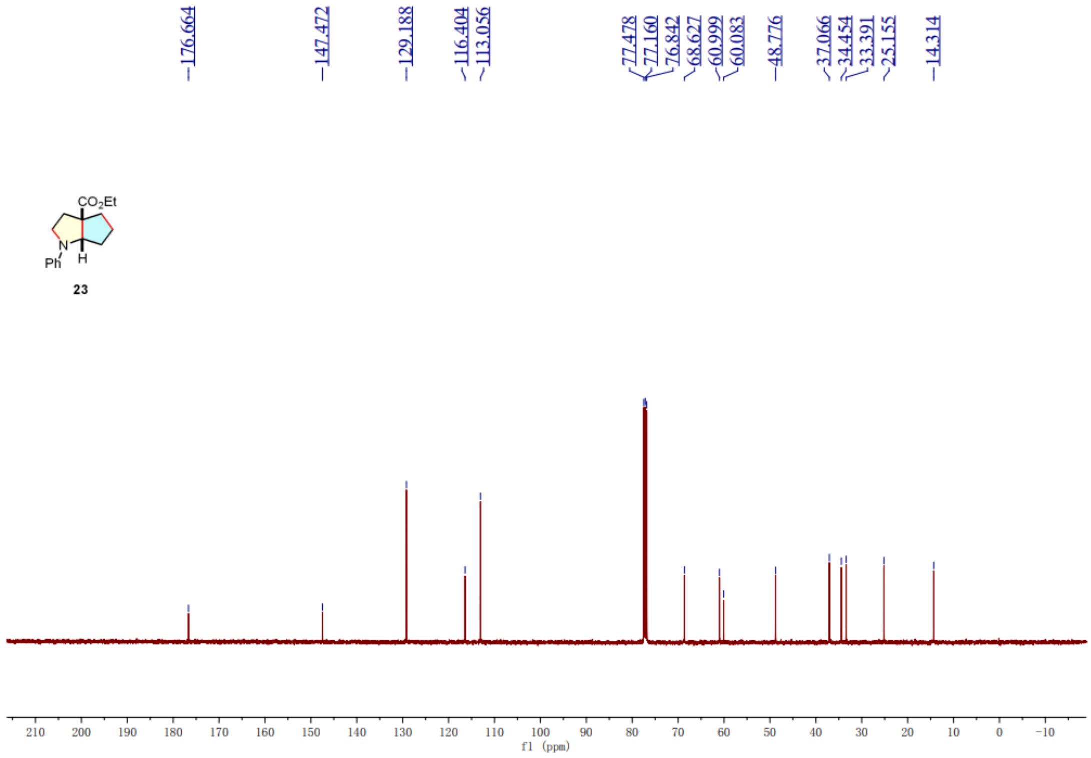

$^{13}C\{^{1}H\}$ NMR Spectrum of Compound **23** (100 MHz, $CDCl_3$)

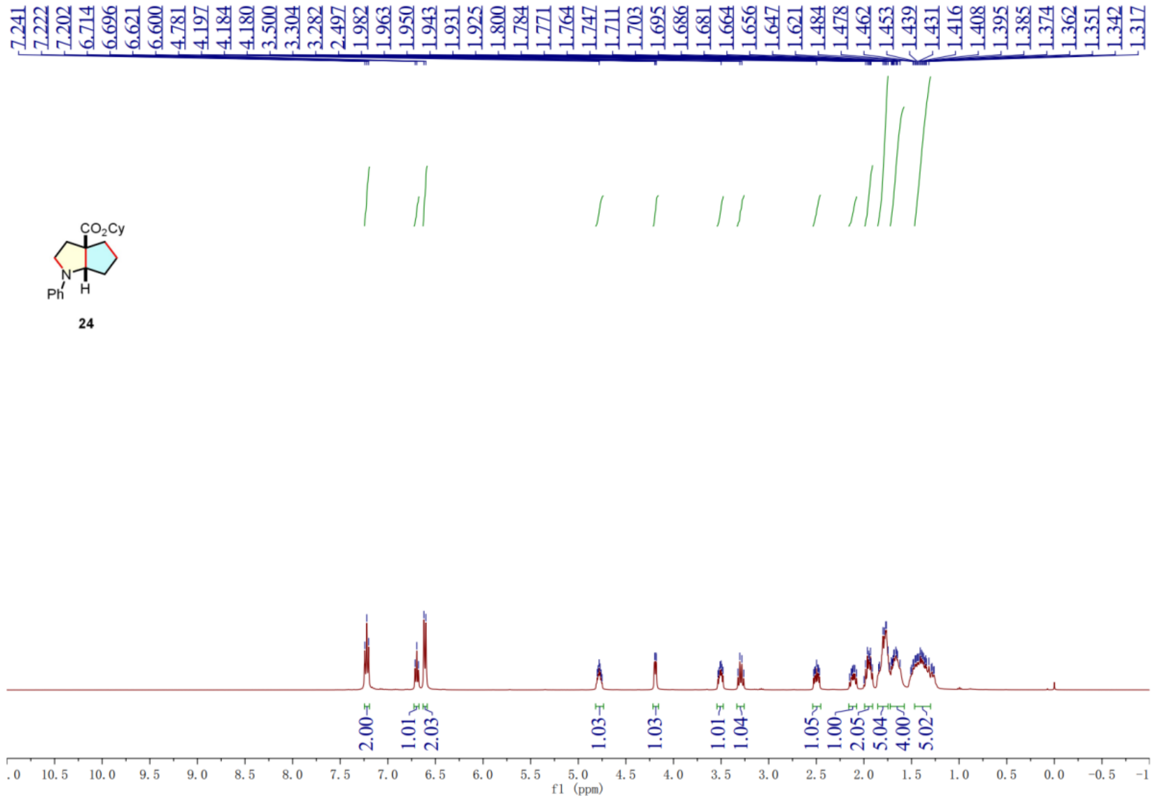

^{1}H NMR Spectrum of Compound **24** (400 MHz, $CDCl_3$)

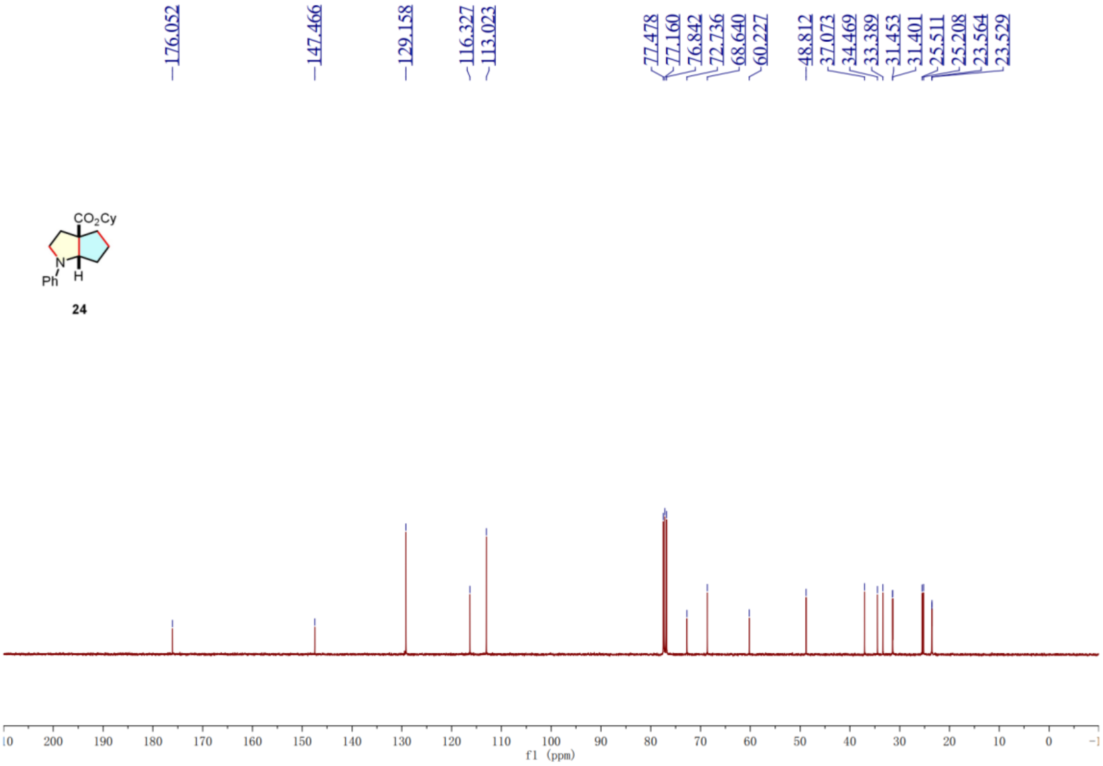

$^{13}C\{^{1}H\}$ NMR Spectrum of Compound **24** (100 MHz, $CDCl_3$)

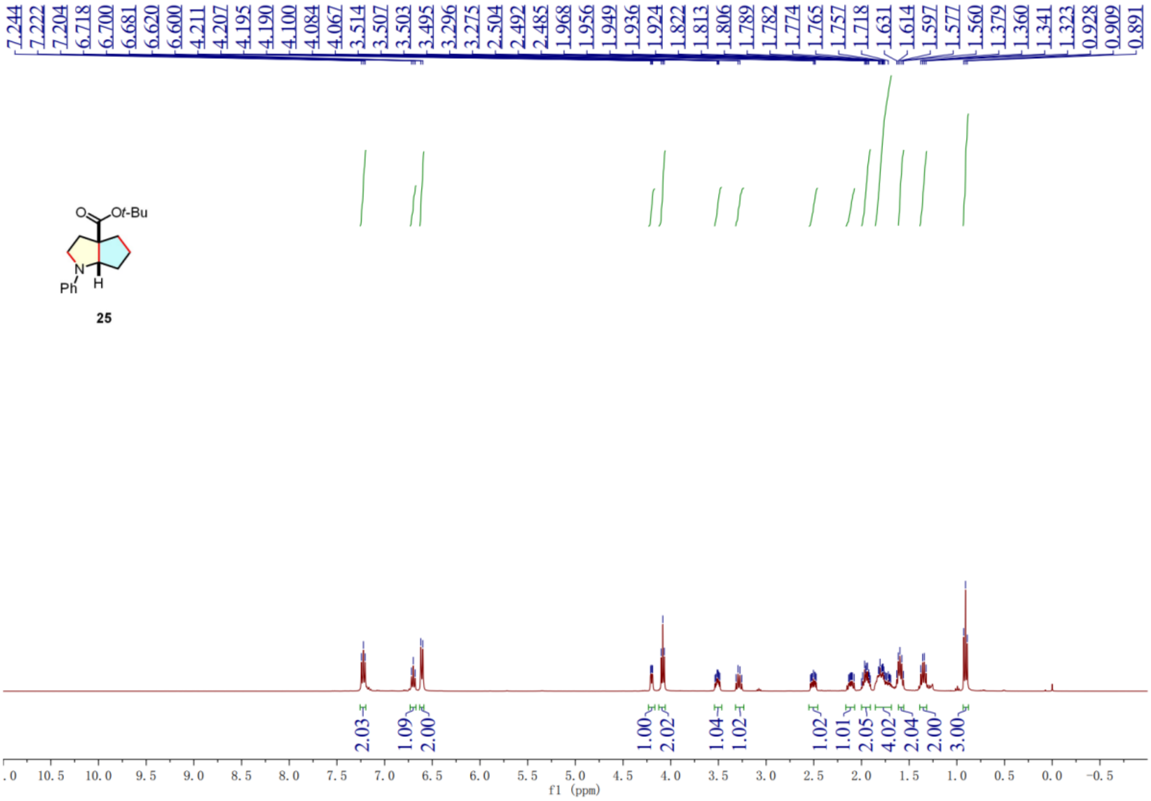

^{1}H NMR Spectrum of Compound **25** (400 MHz, $CDCl_3$)

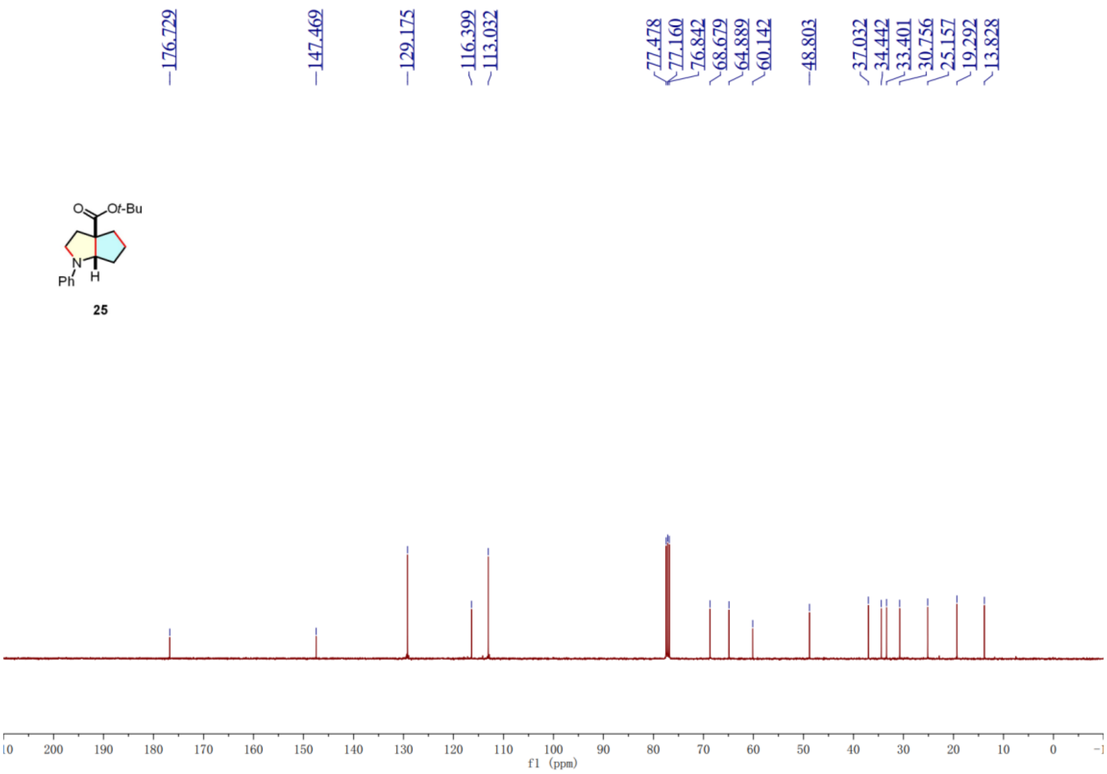

^{13}C{^{1}H} NMR Spectrum of Compound **25** (100 MHz, $CDCl_3$)

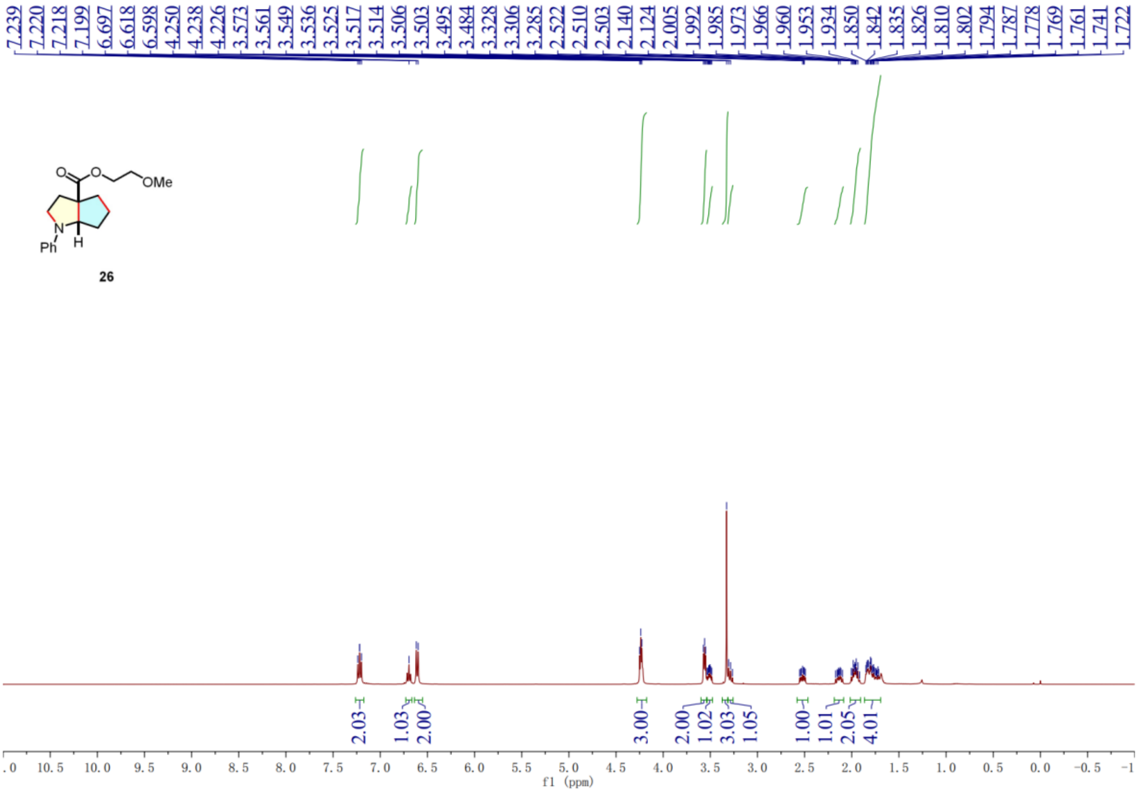

^{1}H NMR Spectrum of Compound **26** (400 MHz, $CDCl_3$)

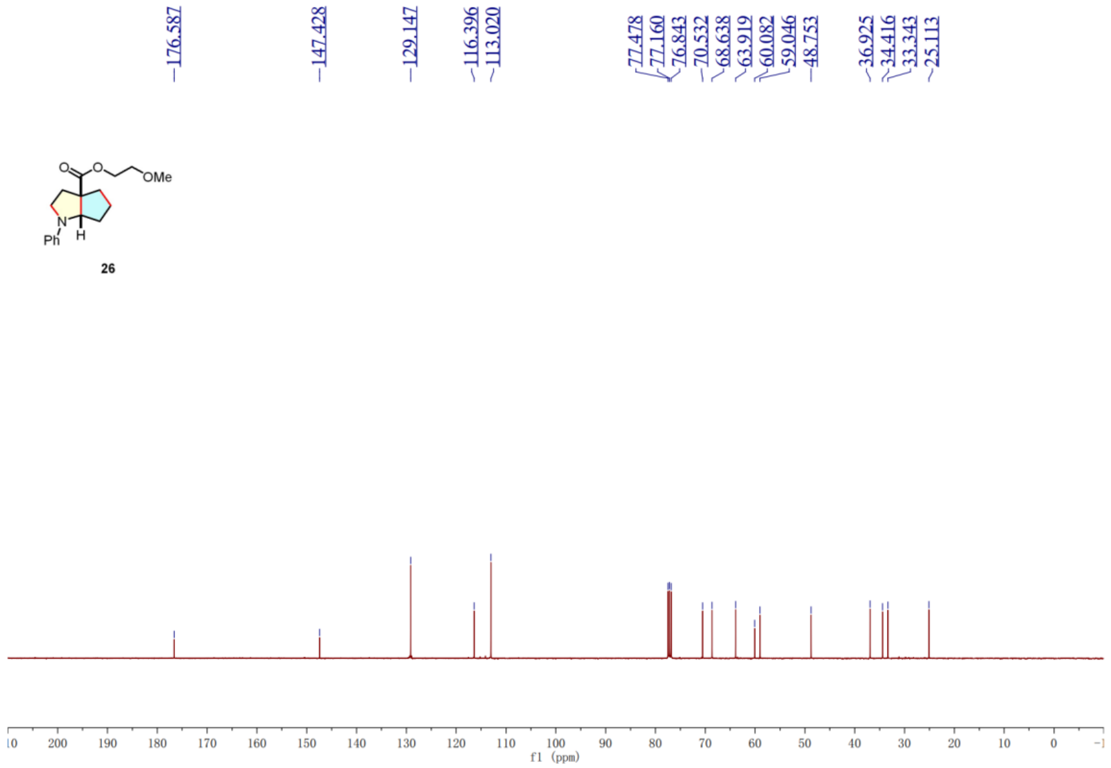

$^{13}C\{^{1}H\}$ NMR Spectrum of Compound **26**(100 MHz, $CDCl_3$)

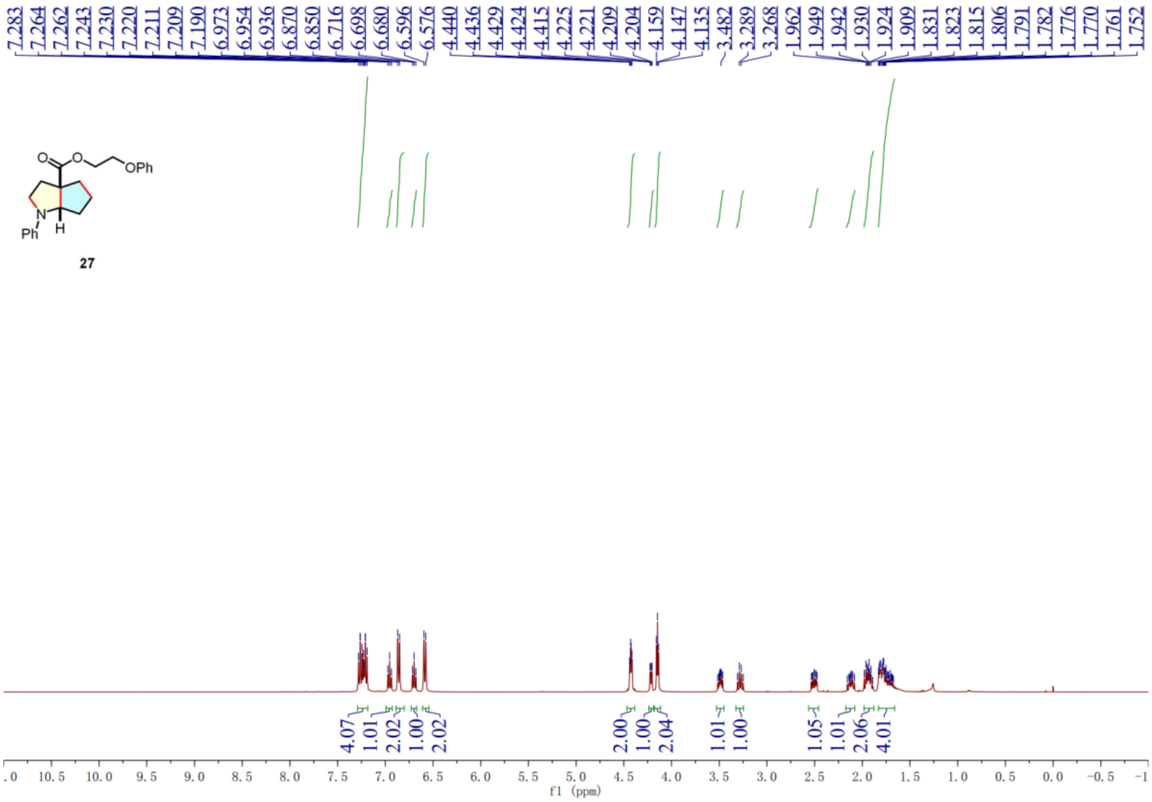

^{1}H NMR Spectrum of Compound **27** (400 MHz, $CDCl_3$)

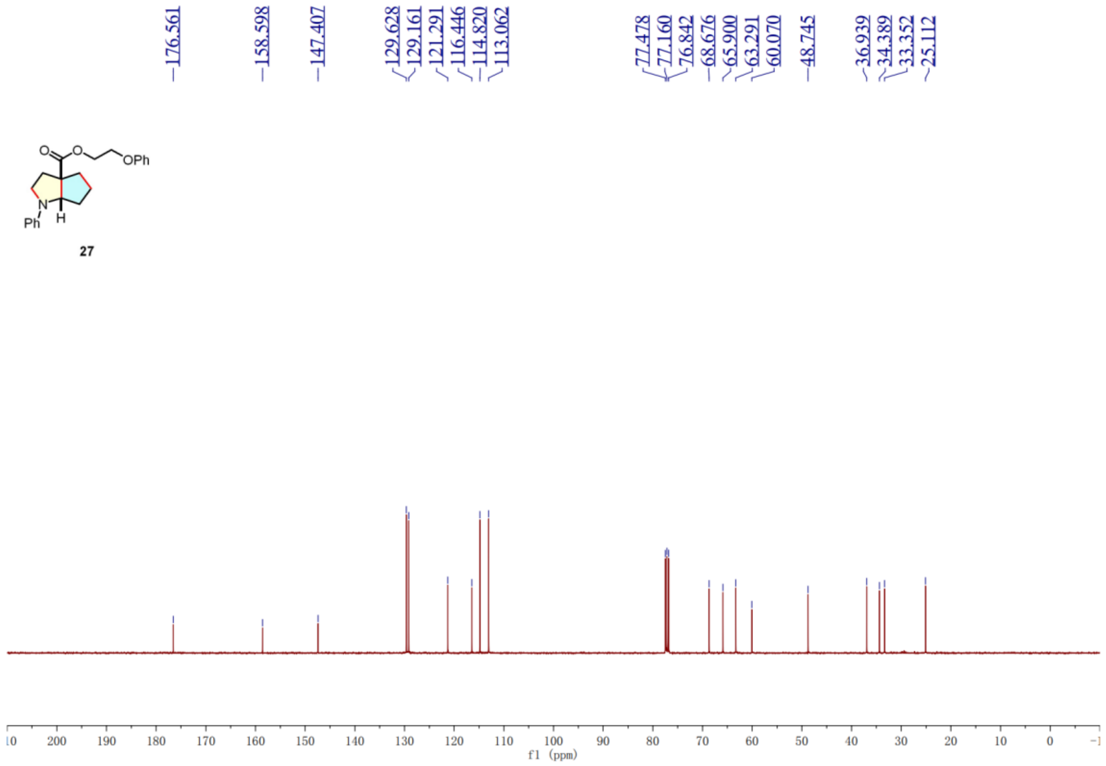

$^{13}C\{^1H\}$ NMR Spectrum of Compound **27**(100 MHz, $CDCl_3$)

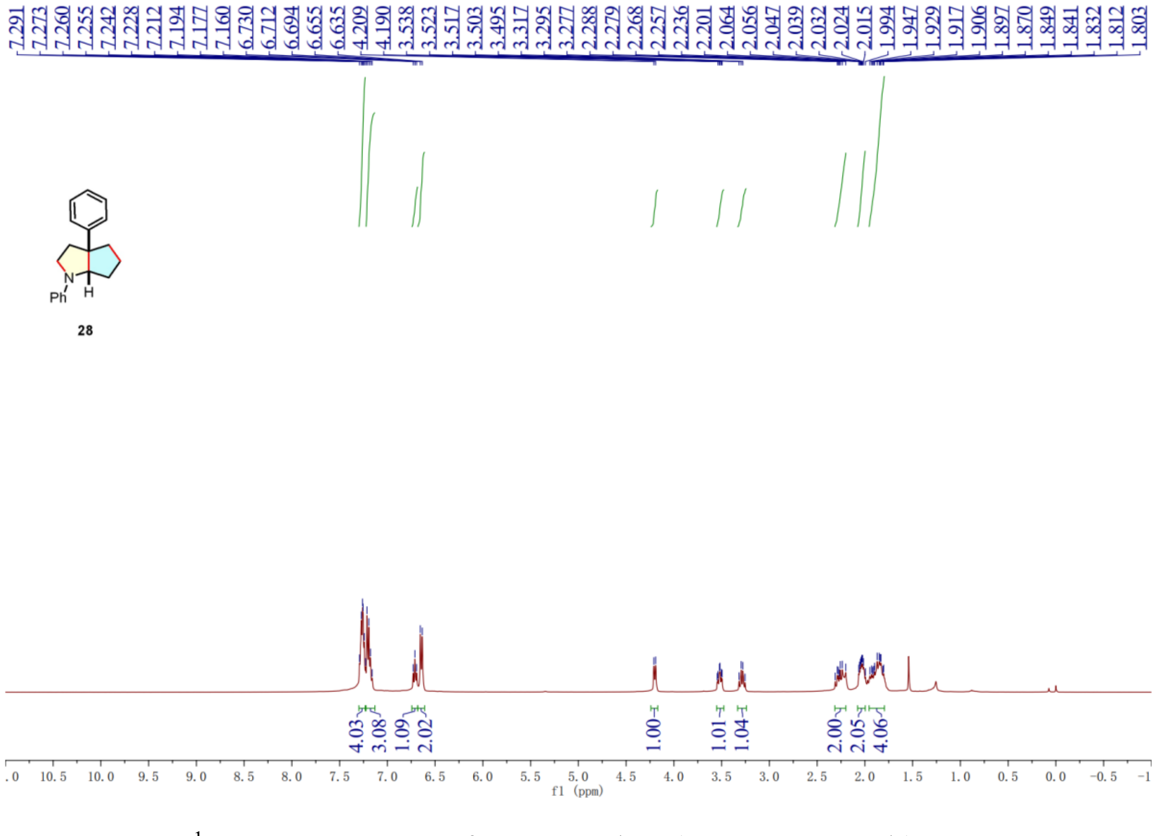

^1H NMR Spectrum of Compound **28** (400 MHz, $CDCl_3$)

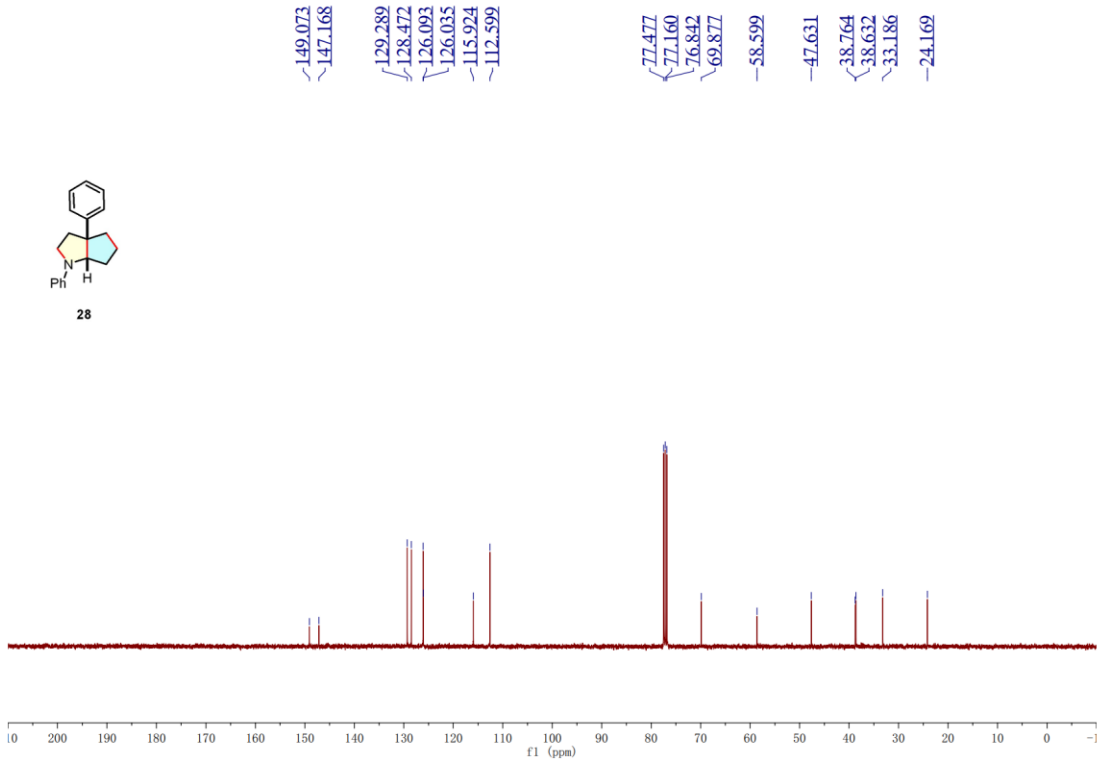

$^{13}C\{^{1}H\}$ NMR Spectrum of Compound **28**(100 MHz, $CDCl_3$)

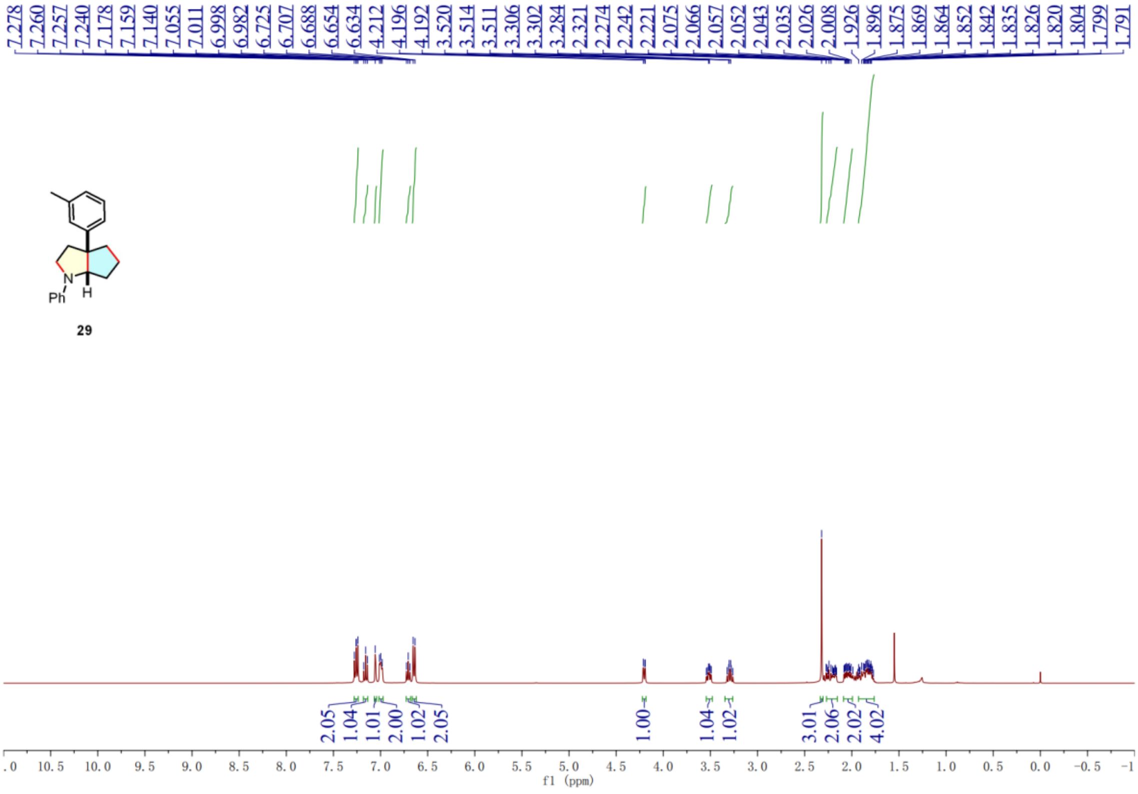

^{1}H NMR Spectrum of Compound **29** (400 MHz, $CDCl_3$)

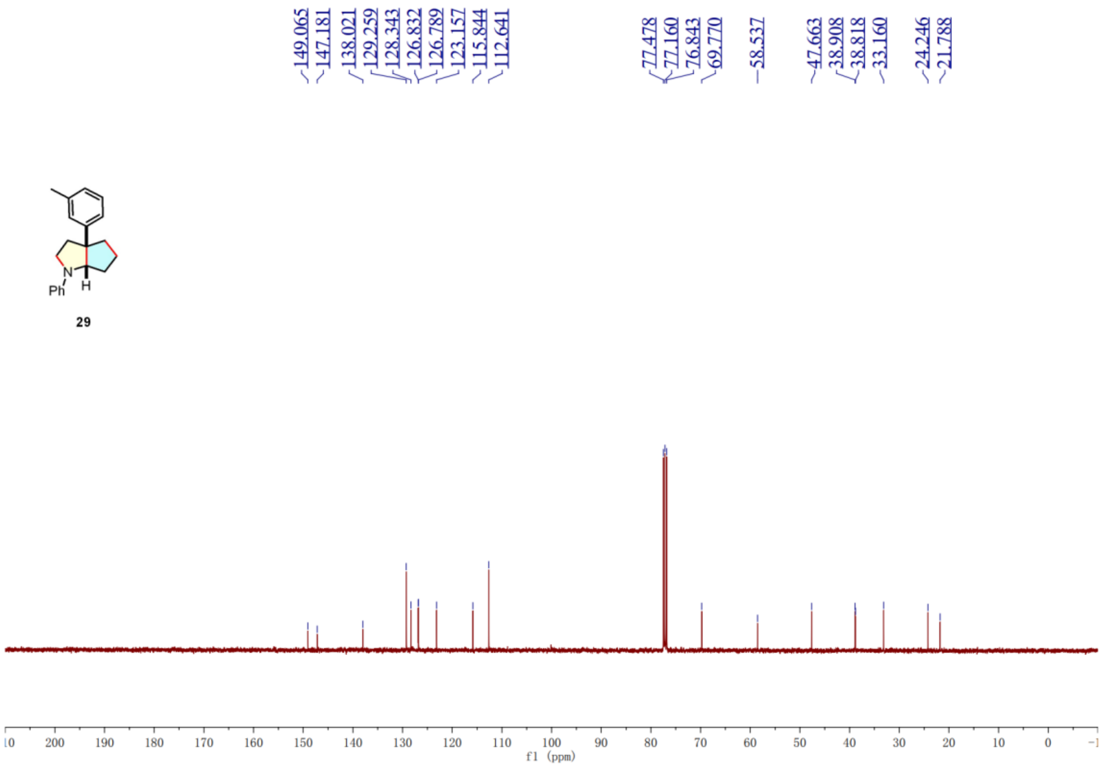

$^{13}C\{^{1}H\}$ NMR Spectrum of Compound **29**(100 MHz, $CDCl_3$)

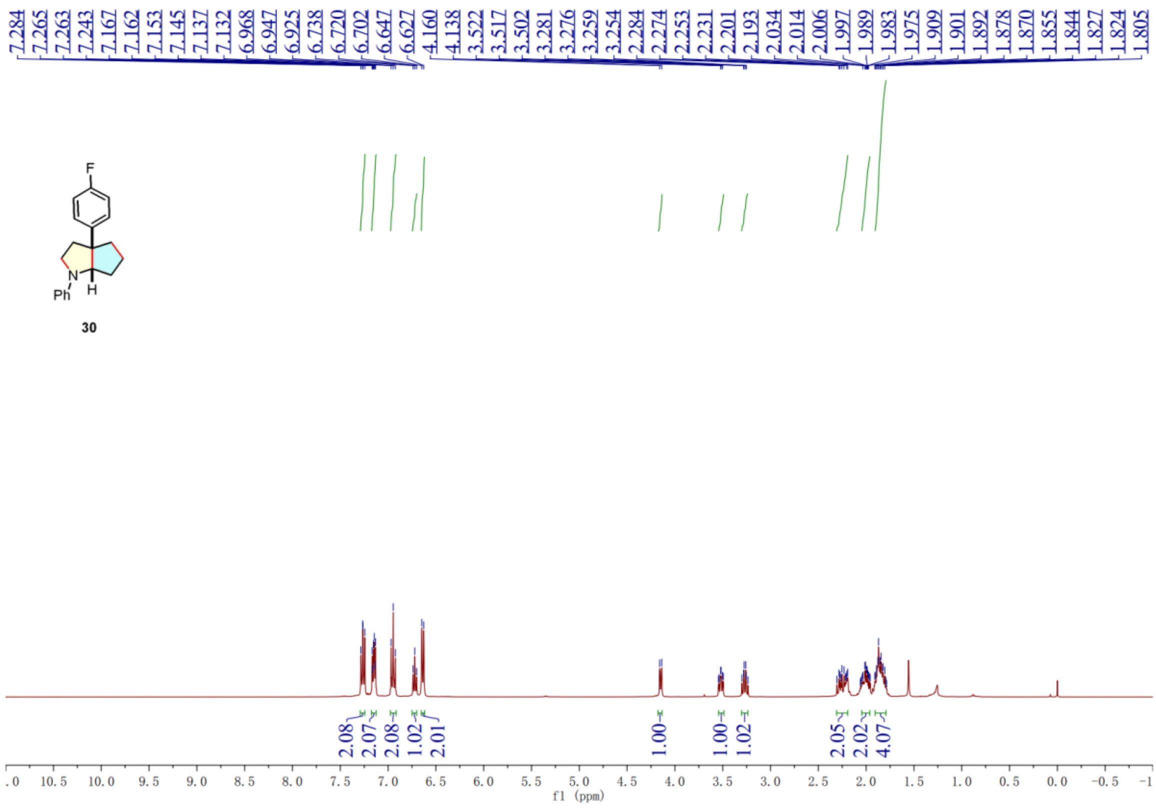

^{1}H NMR Spectrum of Compound **30** (400 MHz, $CDCl_3$)

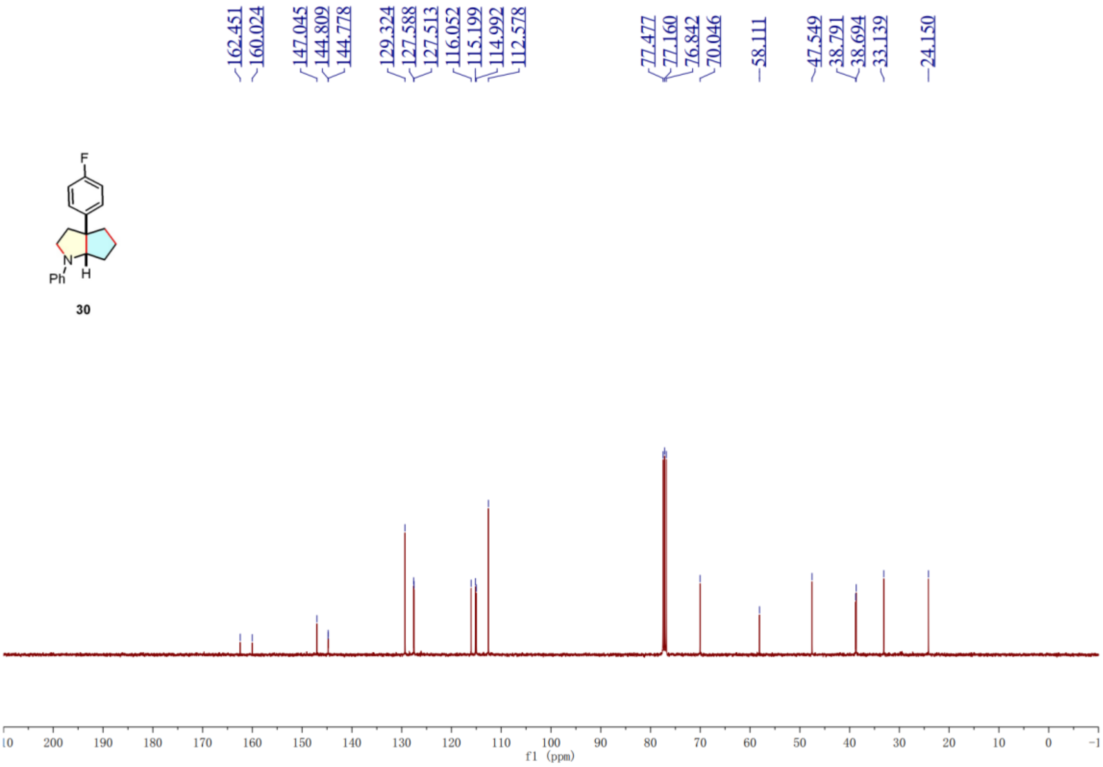

30

$^{13}C\{^{1}H\}$ NMR Spectrum of Compound **30** (100 MHz, $CDCl_3$)

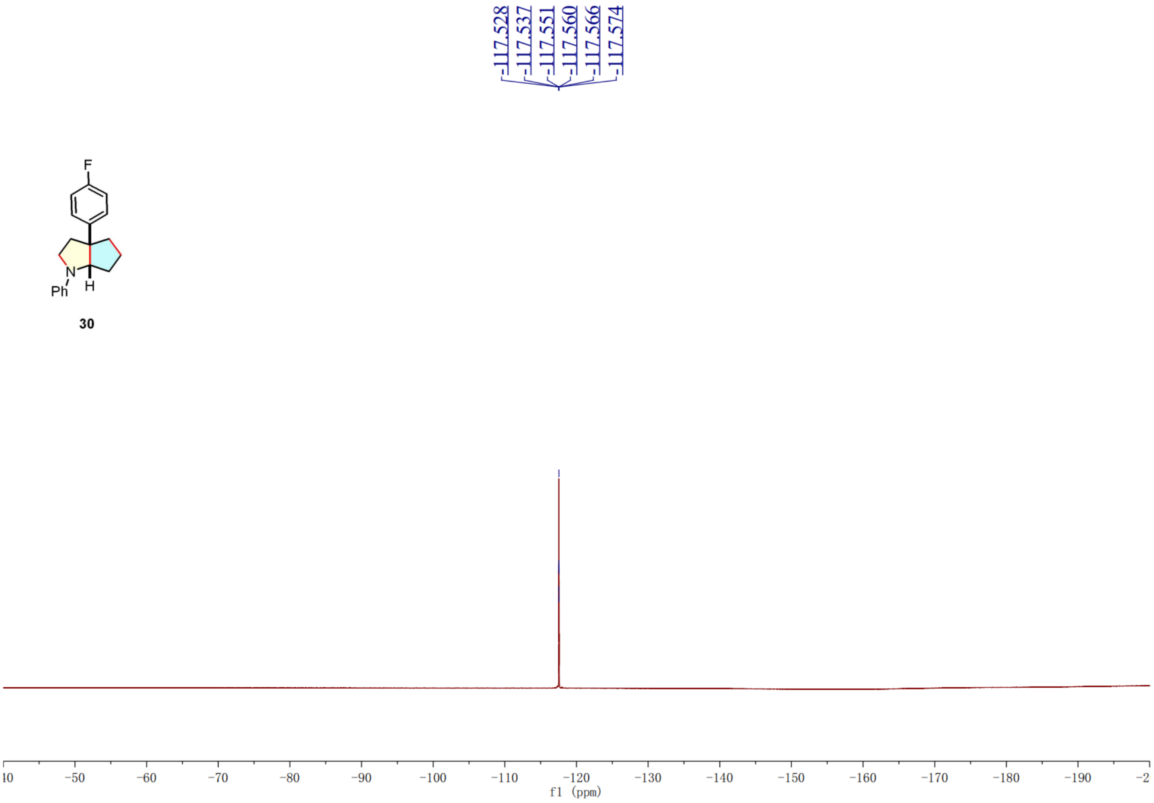

30

$^{19}F\{^{1}H\}$ NMR Spectrum of Compound **30** (376 MHz, $CDCl_3$)

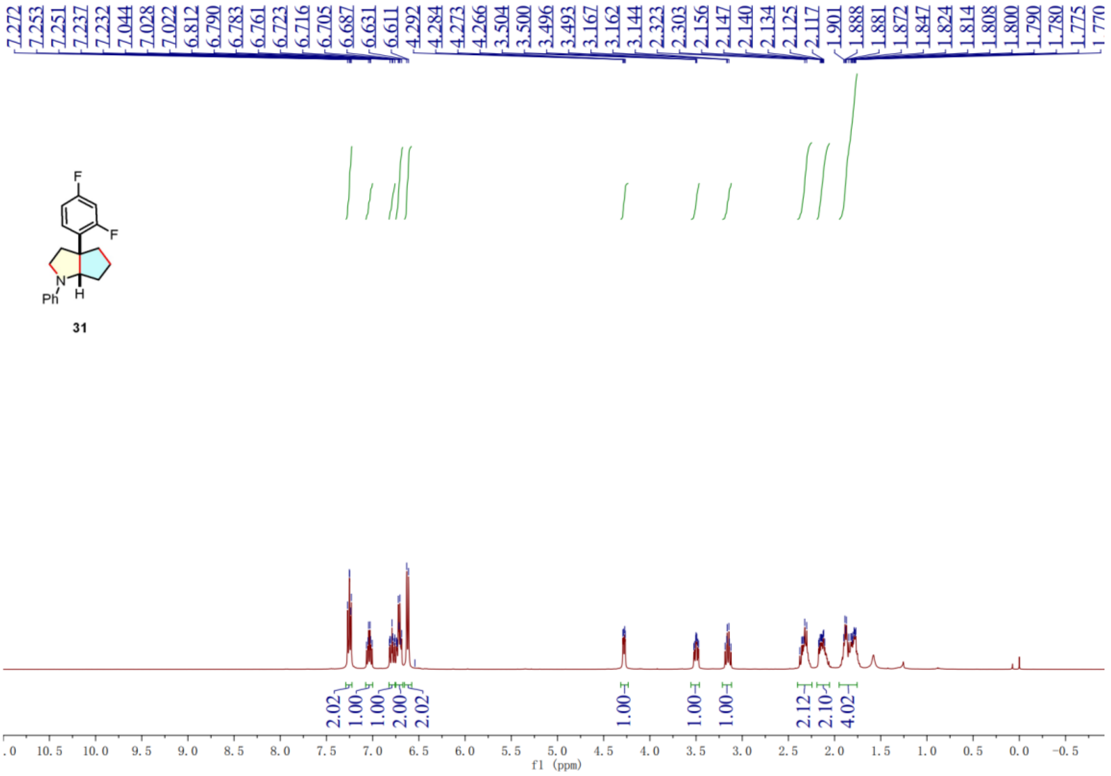

^{1}H NMR Spectrum of Compound **31** (400 MHz, $CDCl_3$)

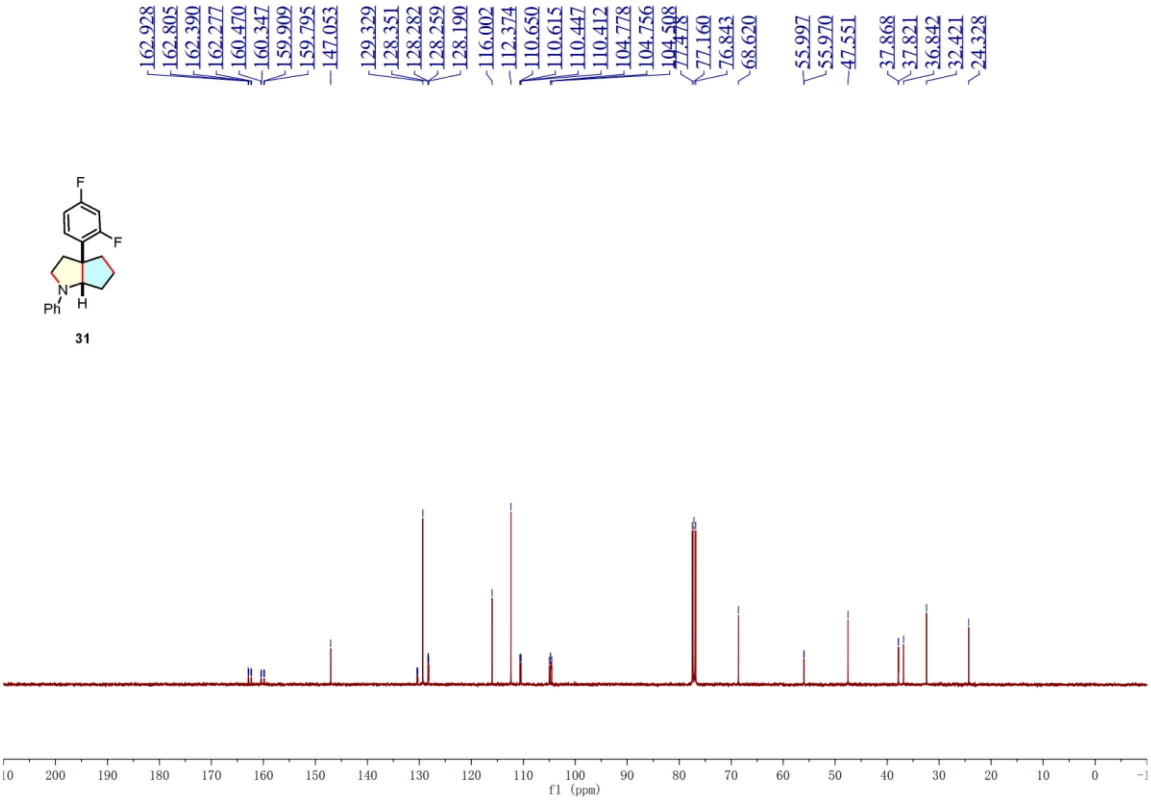

$^{13}C\{^{1}H\}$ NMR Spectrum of Compound **31** (100 MHz, $CDCl_3$)

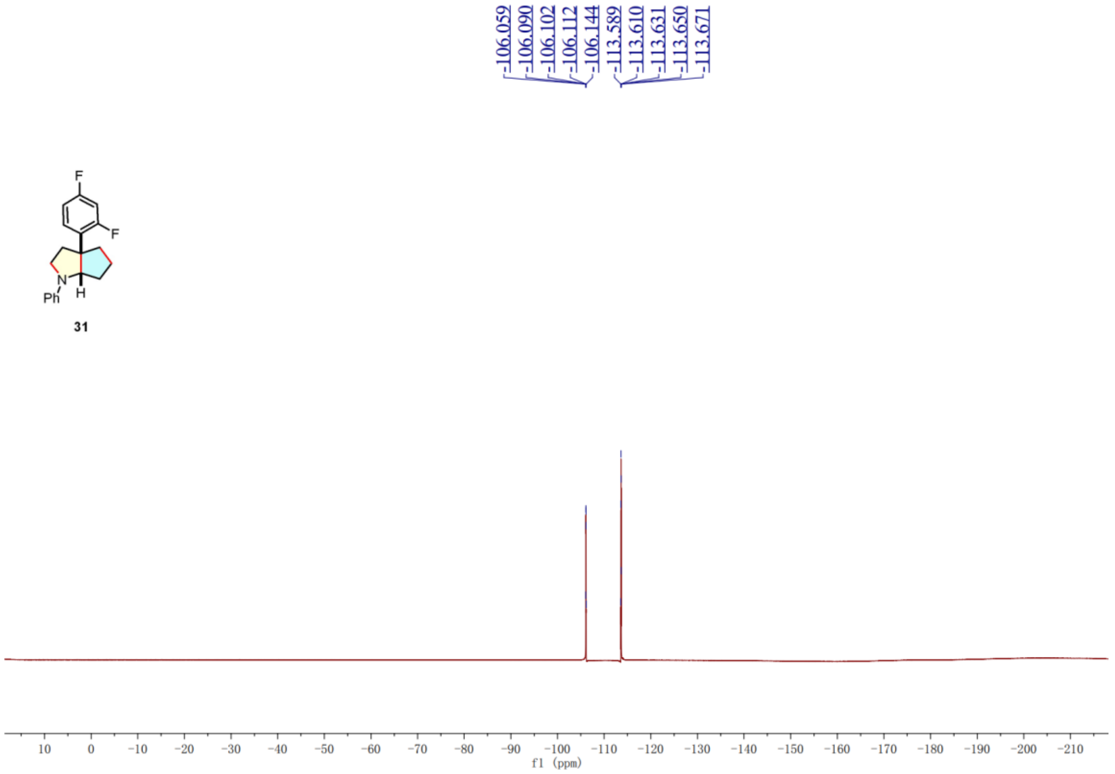

$^{19}F\{^{1}H\}$ NMR Spectrum of Compound **31** (376 MHz, $CDCl_3$)

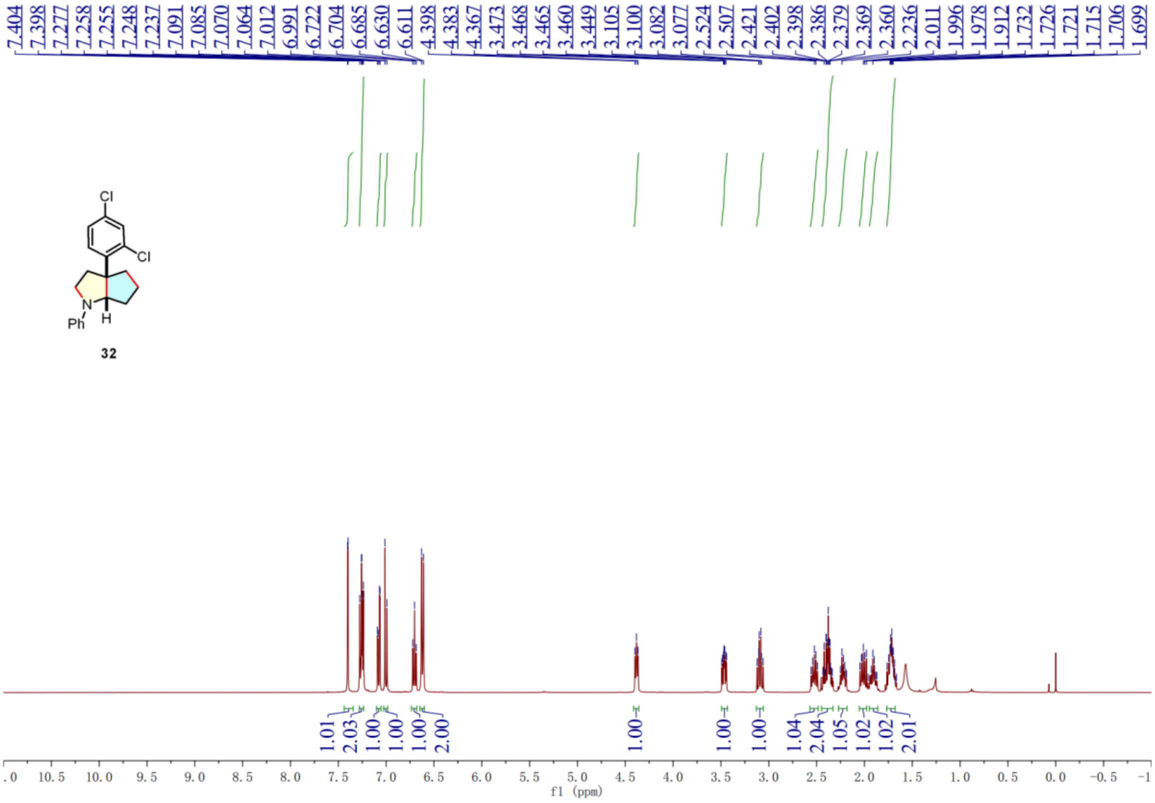

^{1}H NMR Spectrum of Compound **32** (400 MHz, $CDCl_3$)

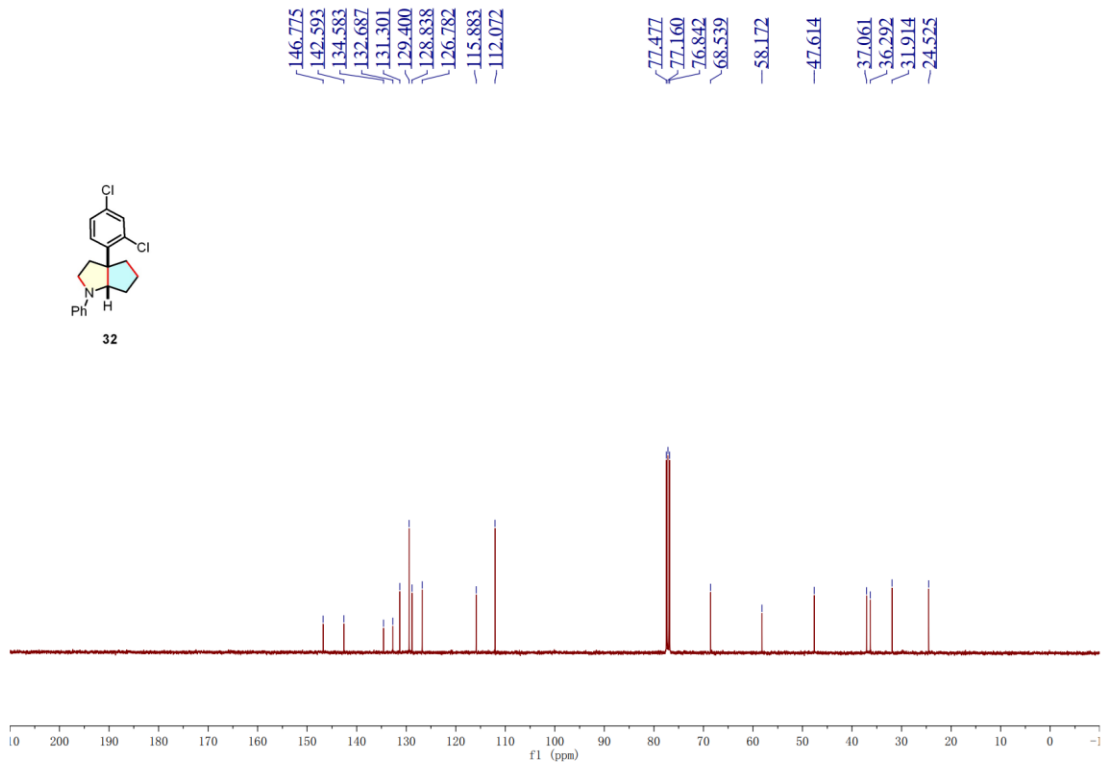

$^{13}C\{^1H\}$ NMR Spectrum of Compound **32** (100 MHz, $CDCl_3$)

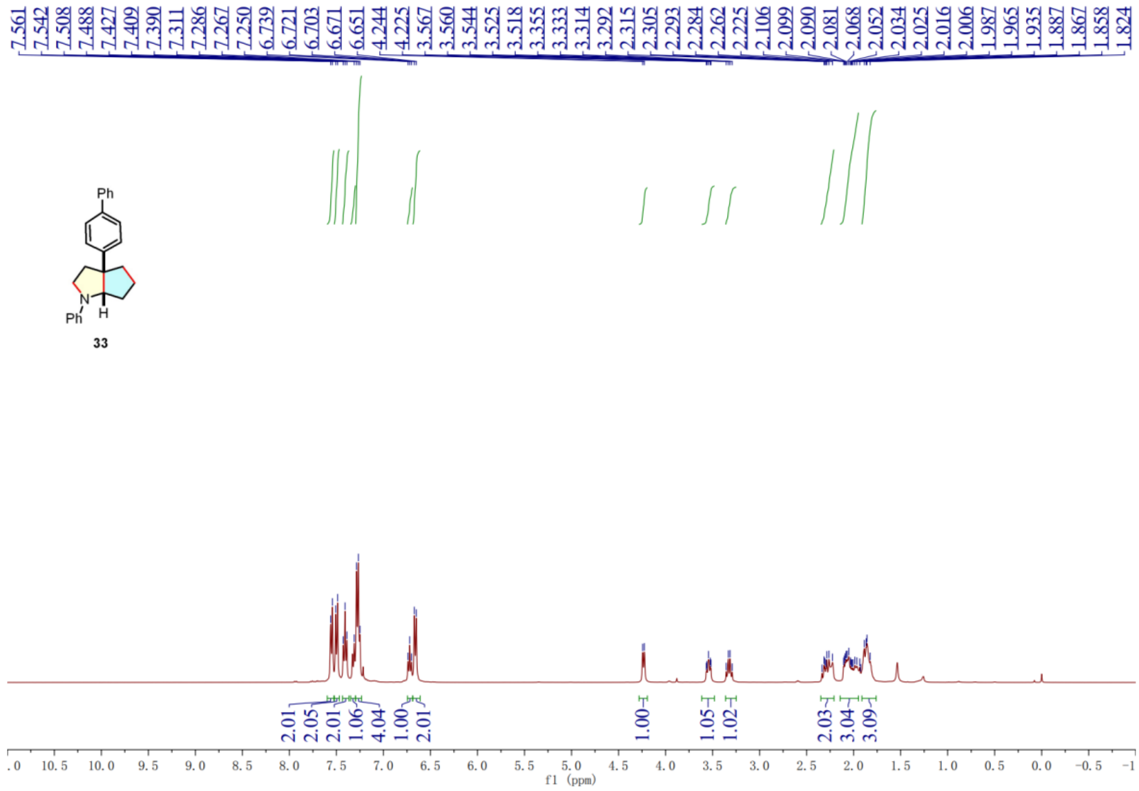

^1H NMR Spectrum of Compound **33** (400 MHz, $CDCl_3$)

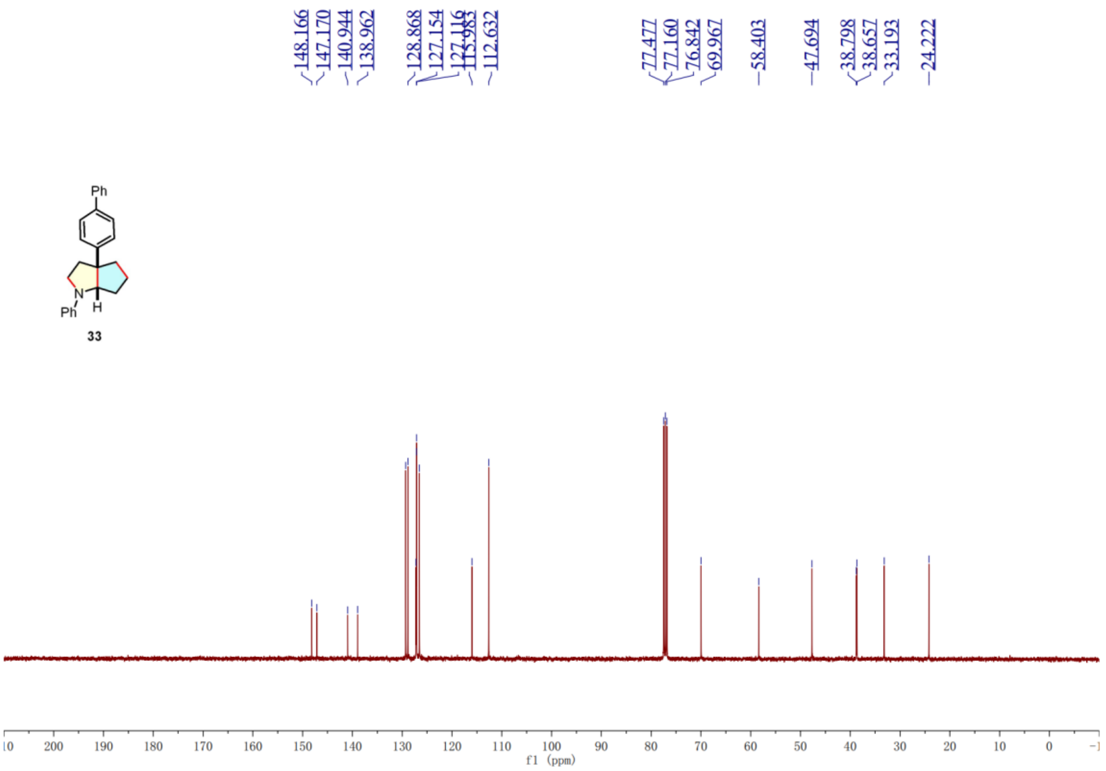

$^{13}C\{^{1}H\}$ NMR Spectrum of Compound **33** (100 MHz, $CDCl_3$)

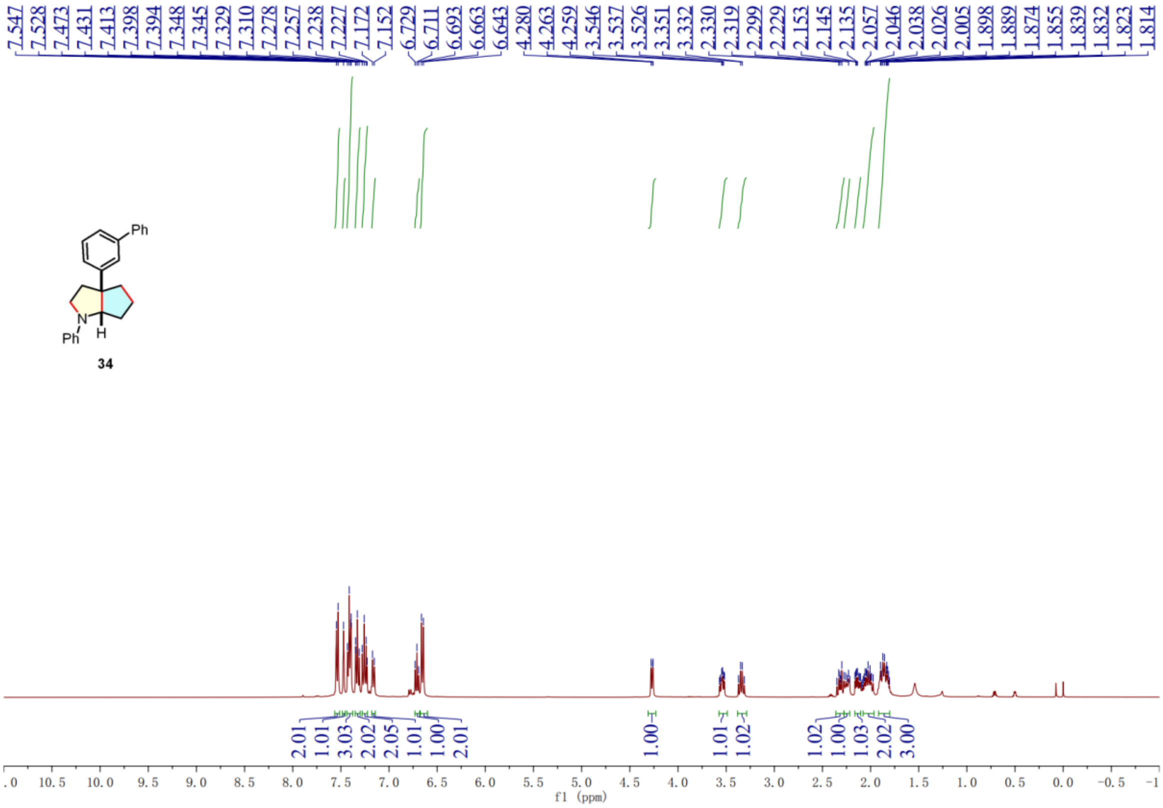

^{1}H NMR Spectrum of Compound **34** (400 MHz, $CDCl_3$)

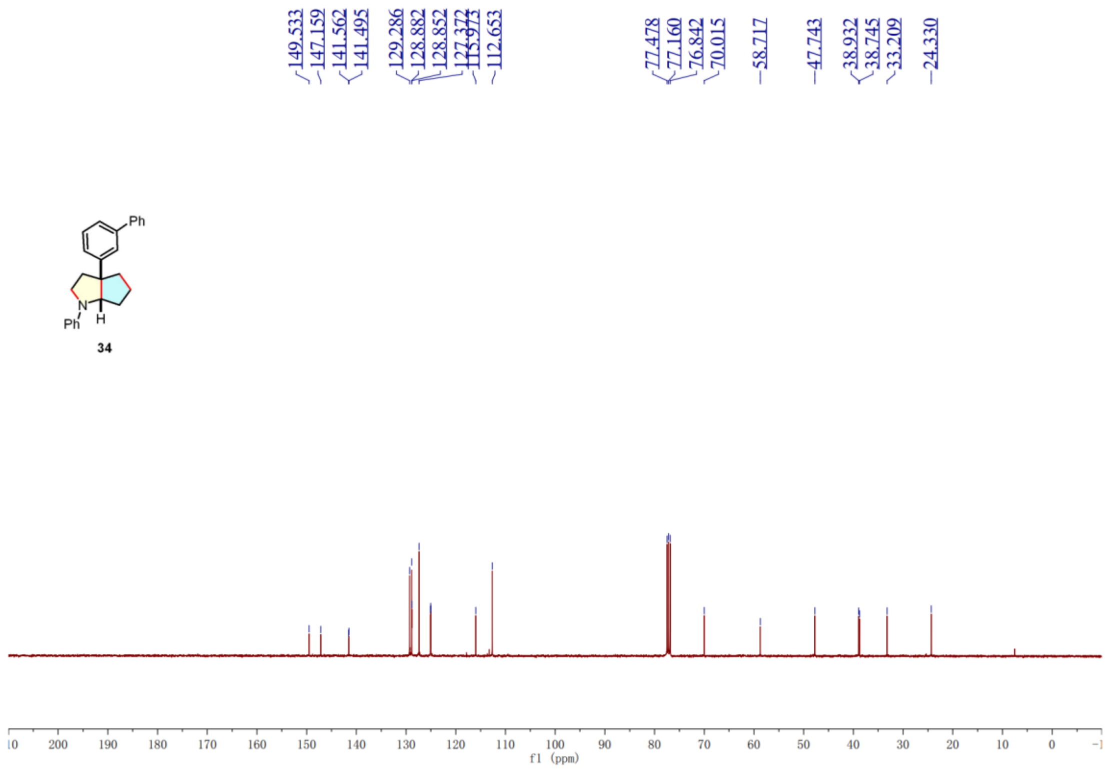

$^{13}C\{^{1}H\}$ NMR Spectrum of Compound **34** (100 MHz, $CDCl_3$)

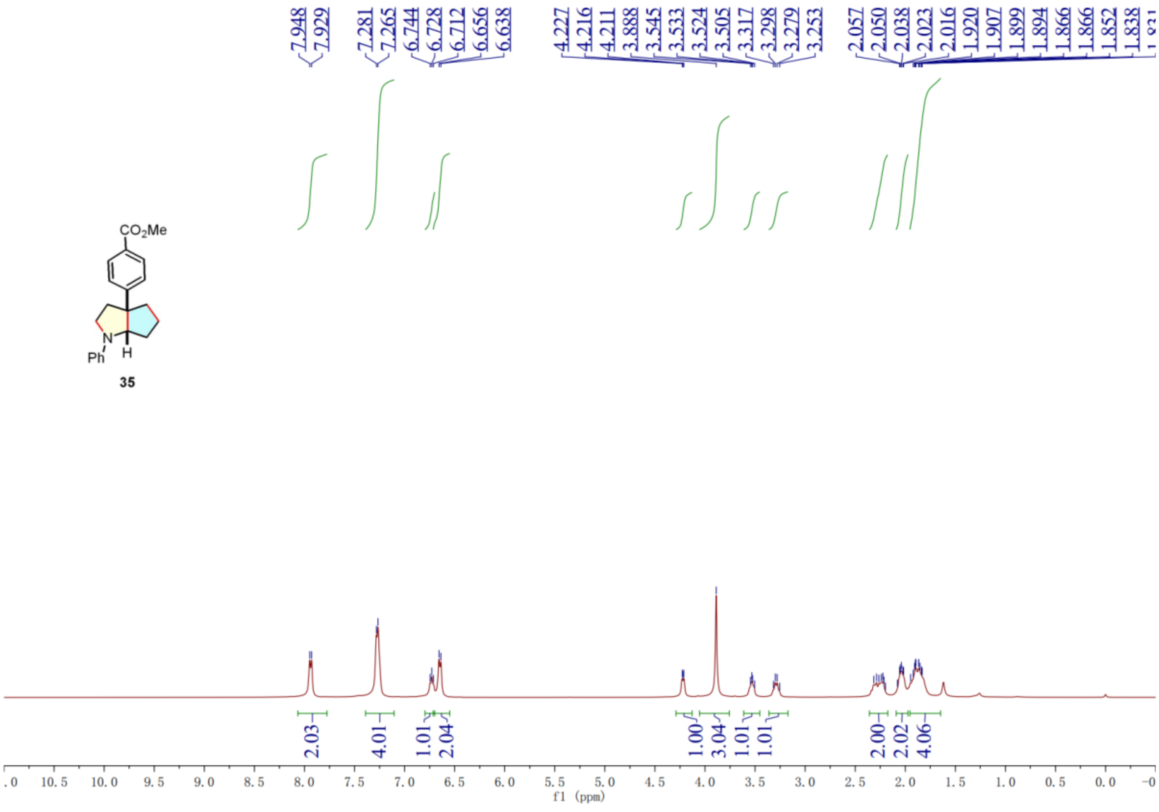

^{1}H NMR Spectrum of Compound **35** (400 MHz, $CDCl_3$)

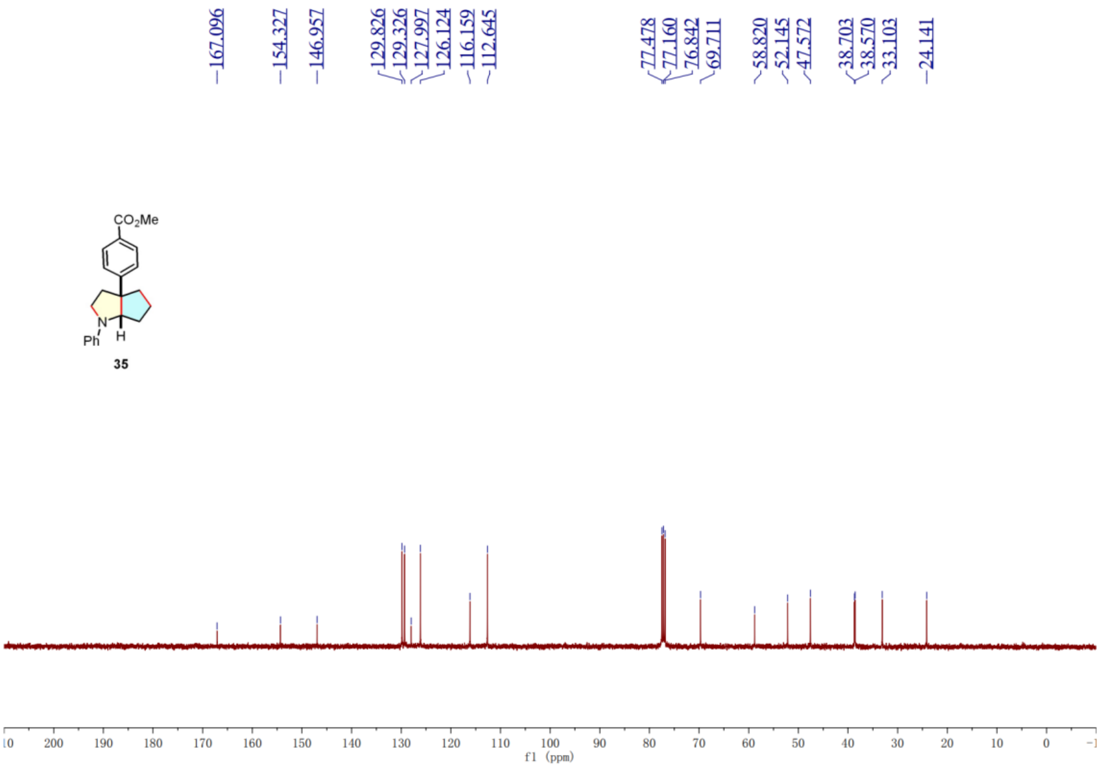

$^{13}C\{^1H\}$ NMR Spectrum of Compound **35** (100 MHz, $CDCl_3$)

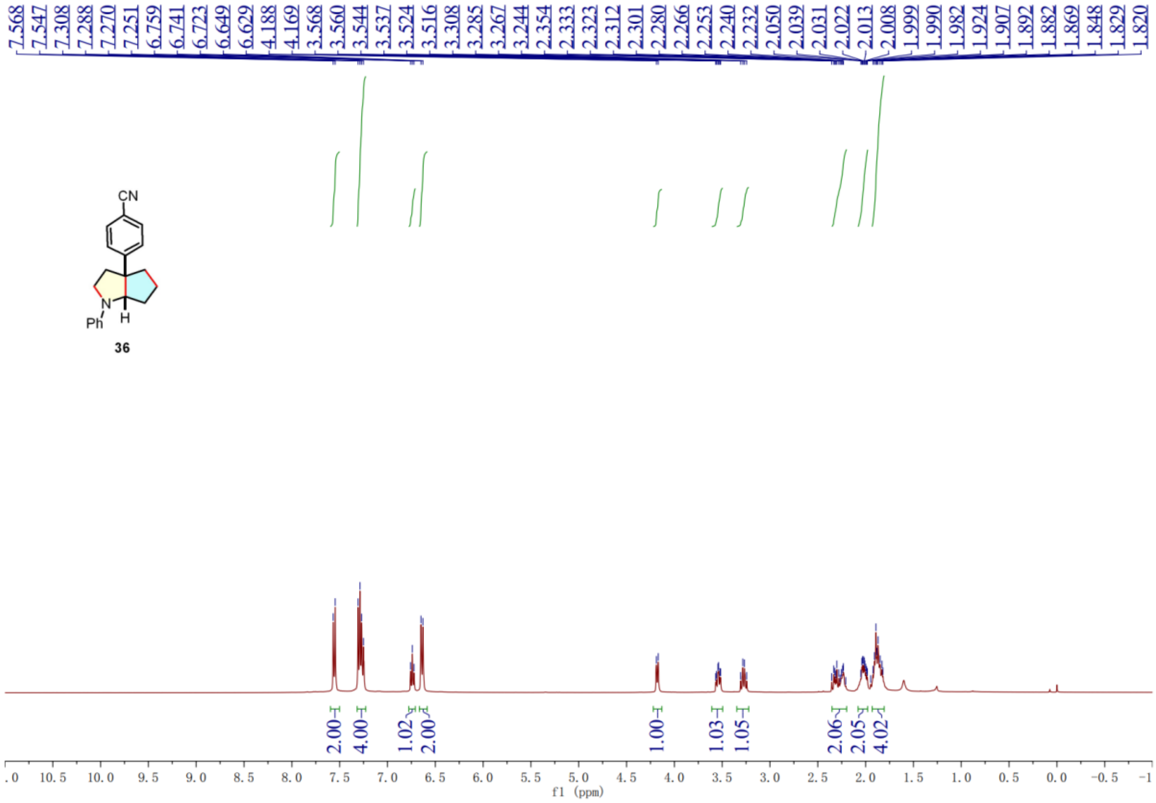

^1H NMR Spectrum of Compound **36** (400 MHz, $CDCl_3$)

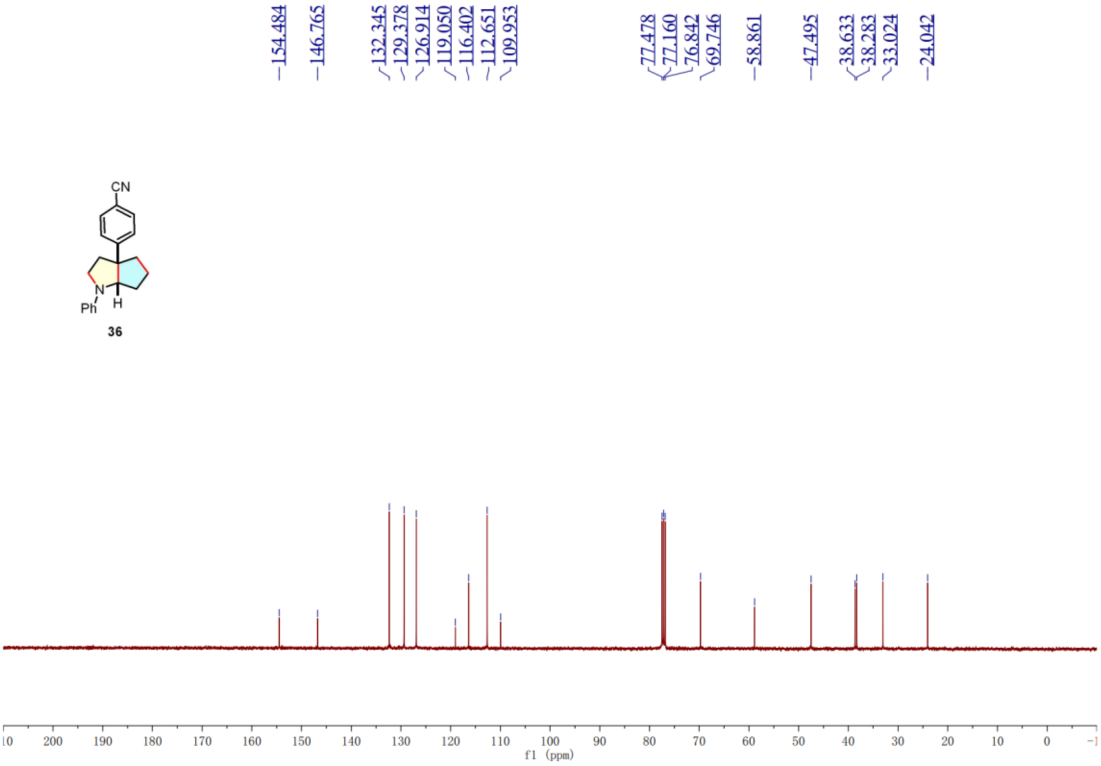

$^{13}C\{^{1}H\}$ NMR Spectrum of Compound **36** (100 MHz, $CDCl_3$)

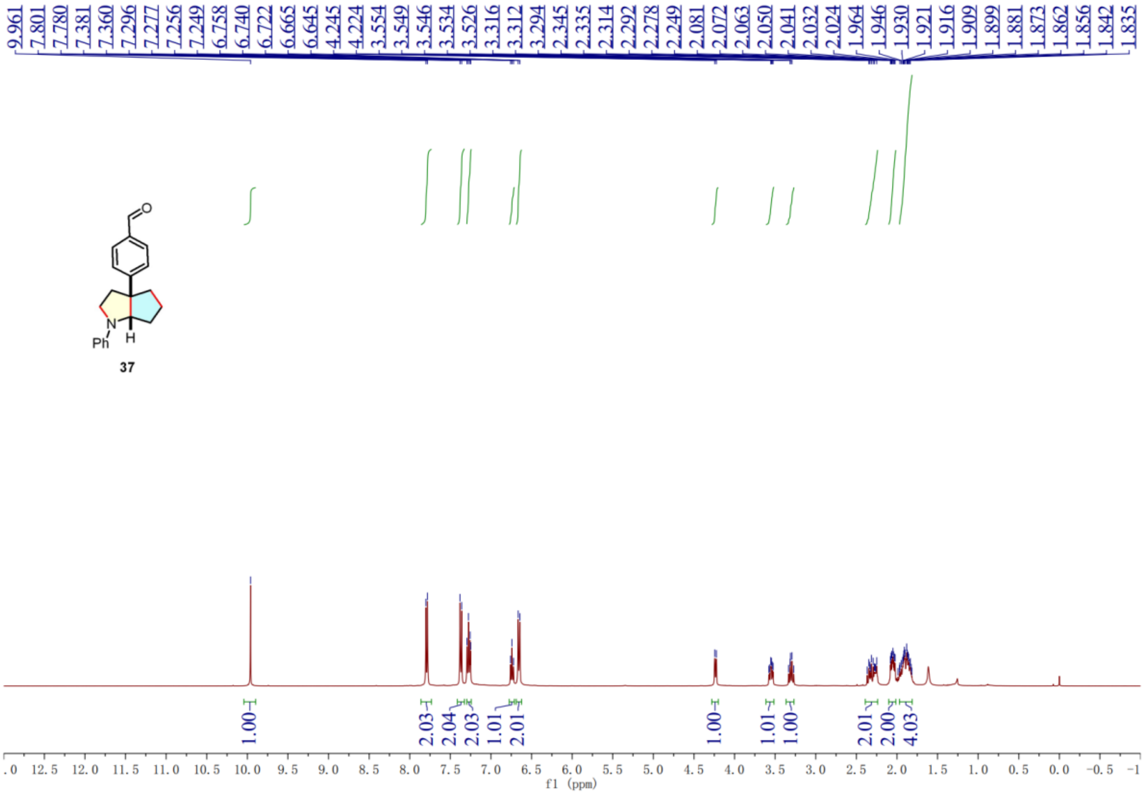

^{1}H NMR Spectrum of Compound **37** (400 MHz, $CDCl_3$)

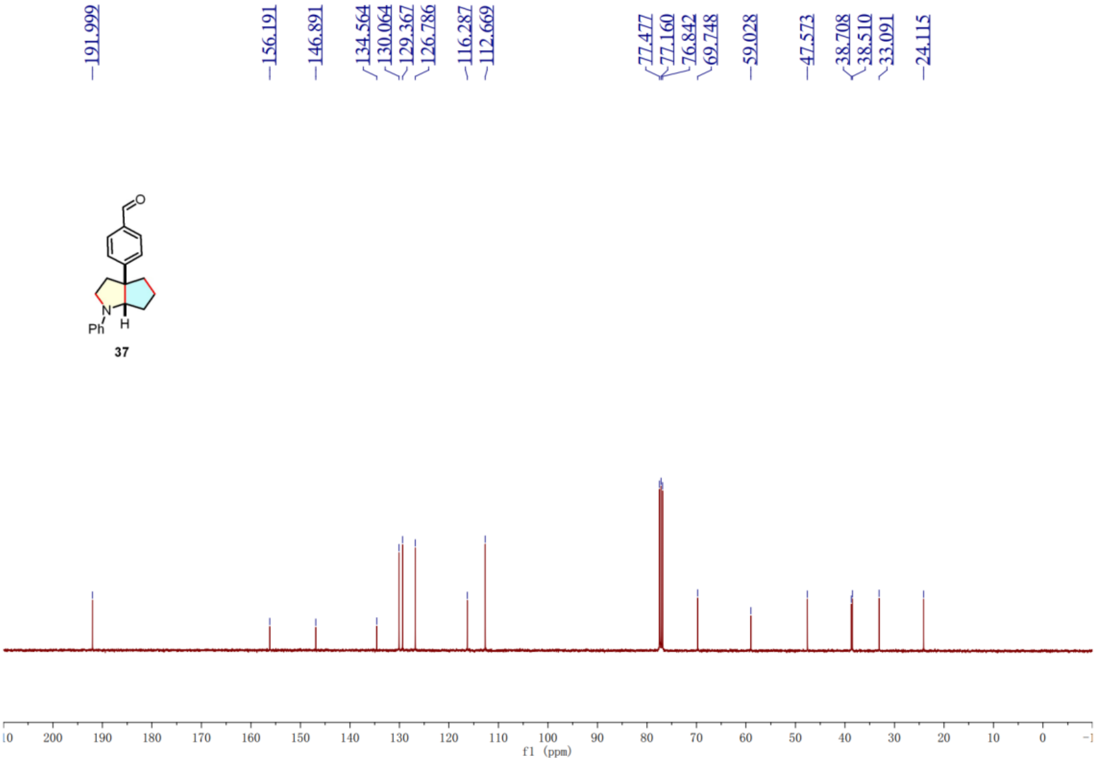

$^{13}C\{^{1}H\}$ NMR Spectrum of Compound **37** (100 MHz, $CDCl_3$)

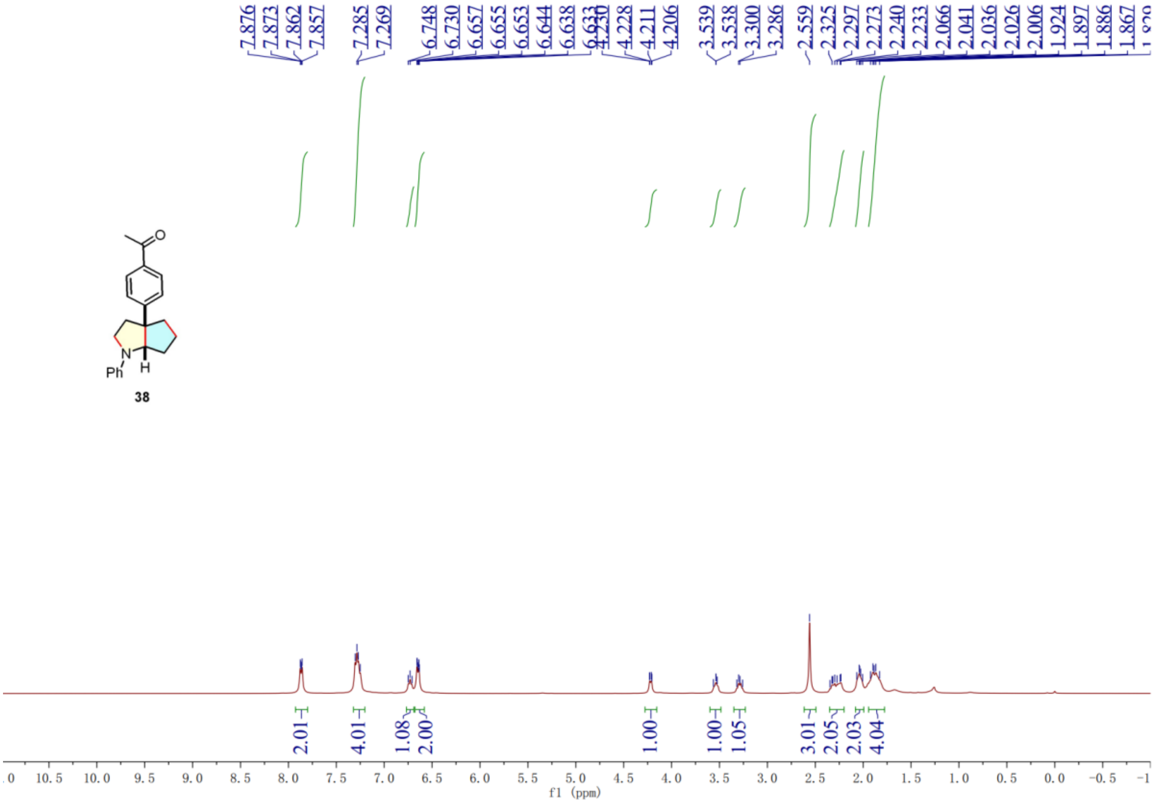

^{1}H NMR Spectrum of Compound **38** (400 MHz, $CDCl_3$)

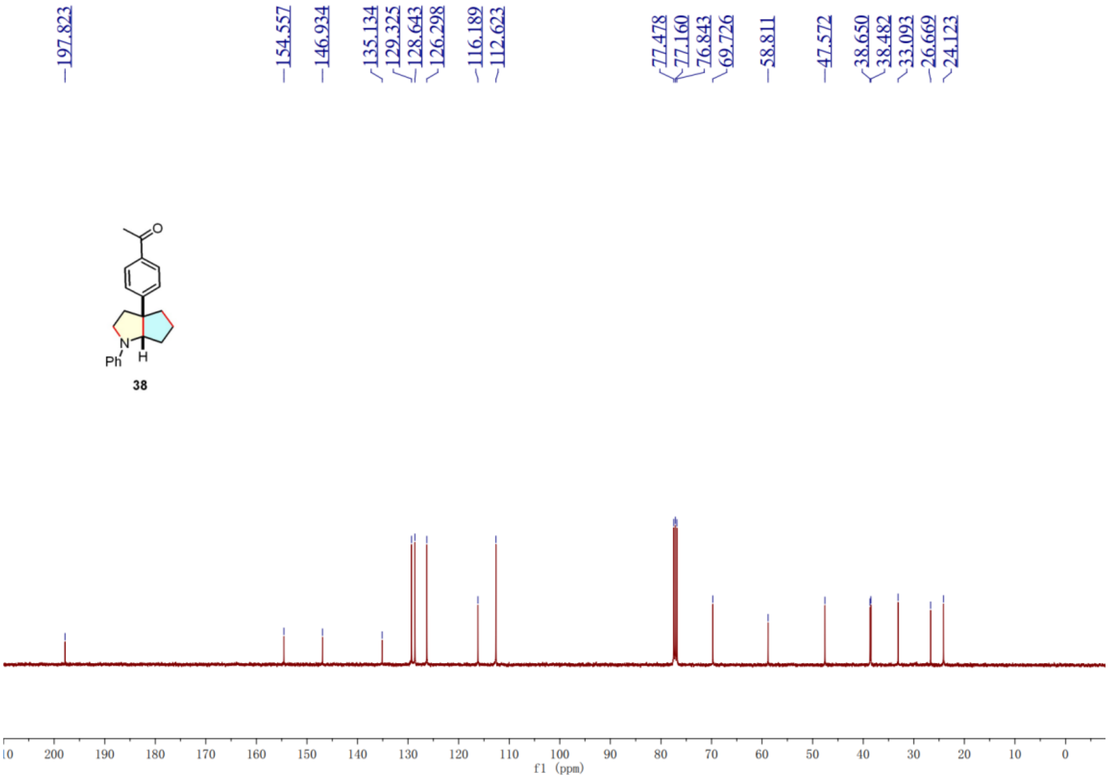

$^{13}C\{^{1}H\}$ NMR Spectrum of Compound **38** (100 MHz, $CDCl_3$)

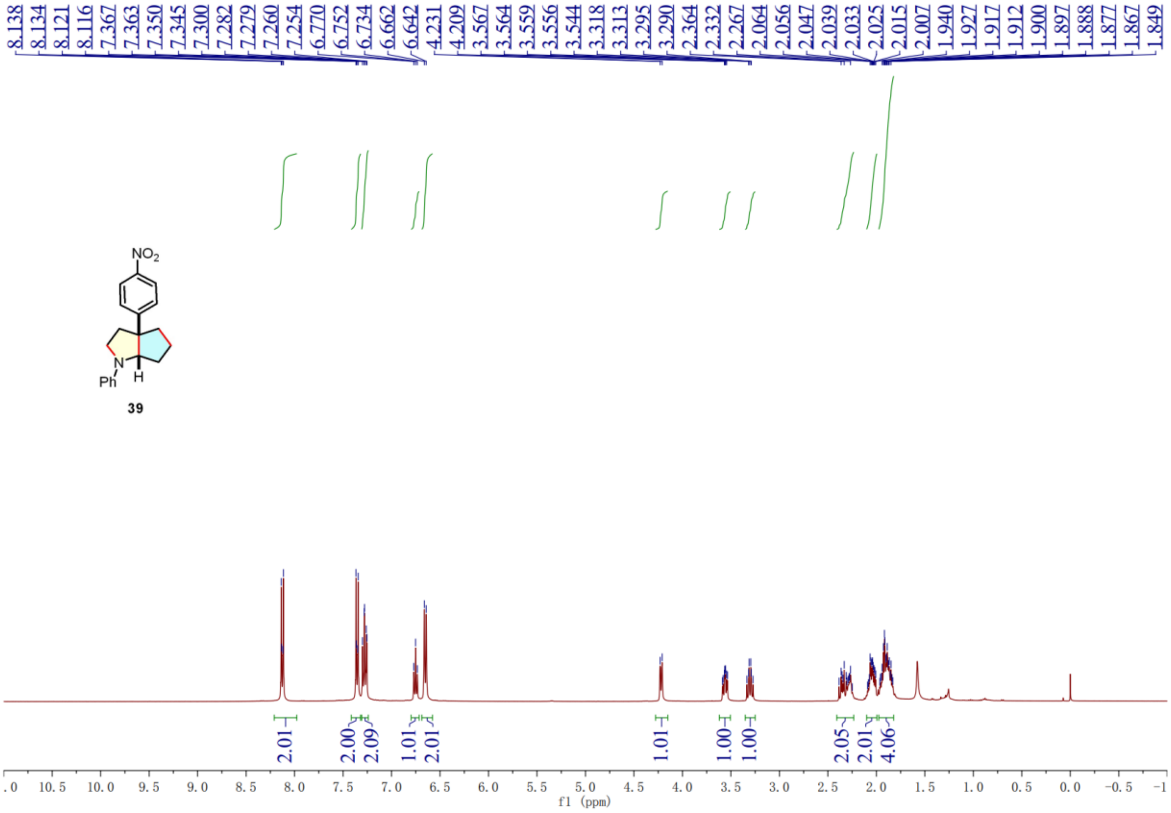

^{1}H NMR Spectrum of Compound **39** (400 MHz, $CDCl_3$)

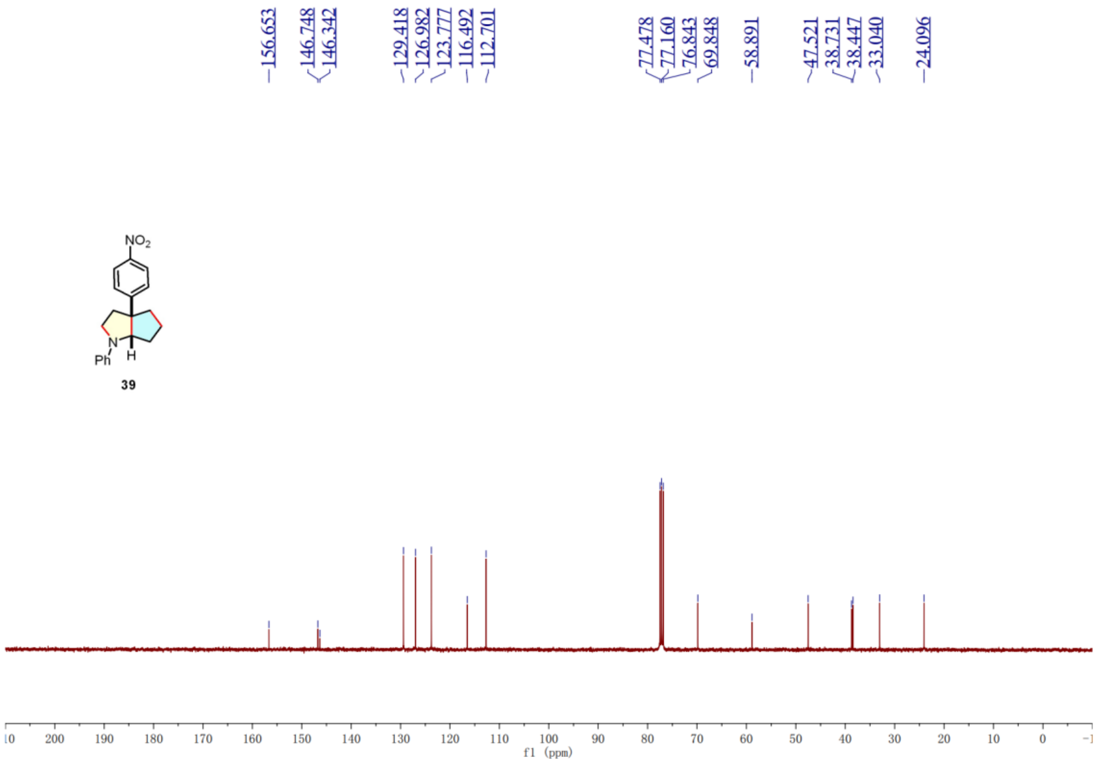

^{13}C{^{1}H} NMR Spectrum of Compound **39** (100 MHz, $CDCl_3$)

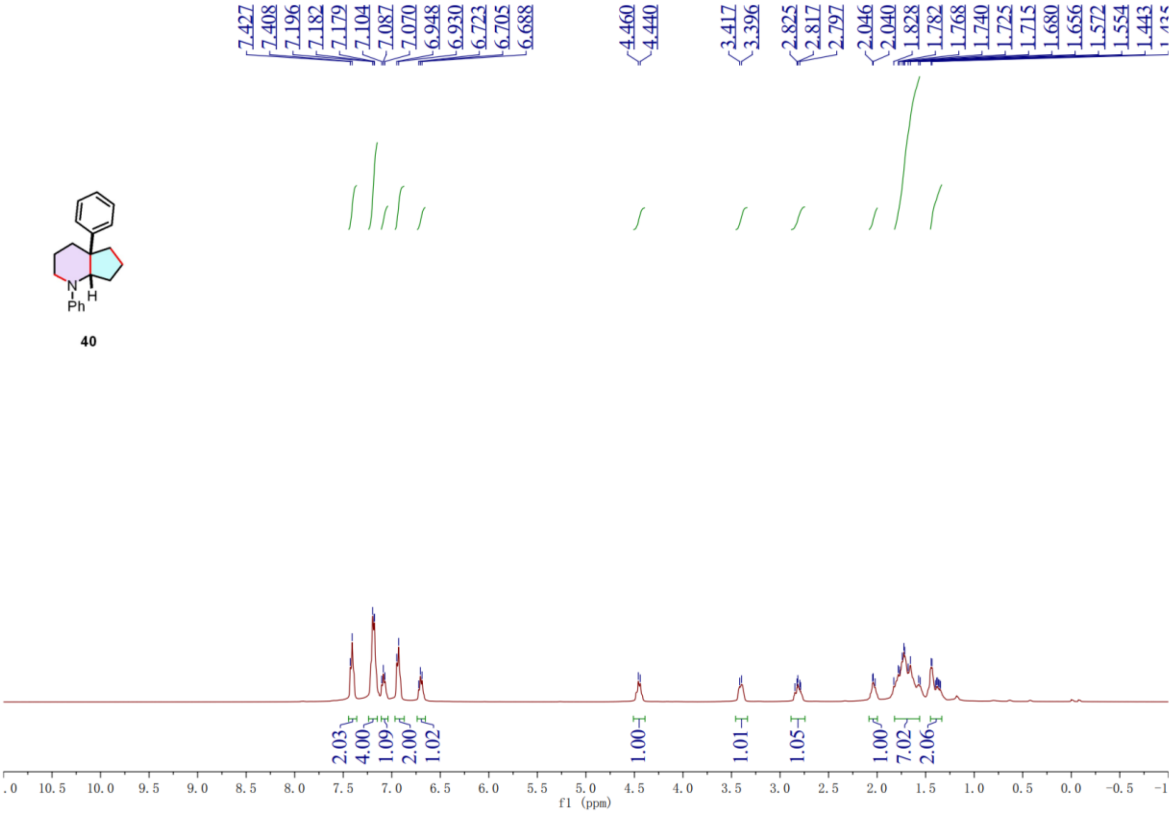

^{1}H NMR Spectrum of Compound **40** (400 MHz, $CDCl_3$)

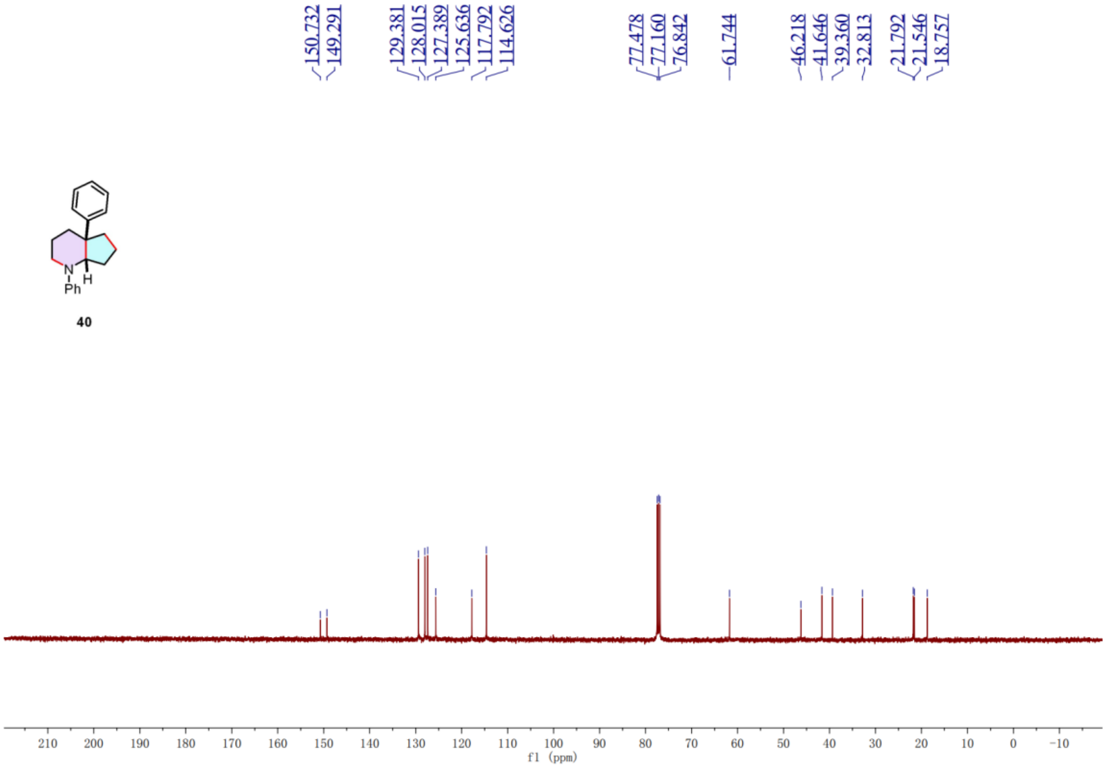

$^{13}C\{^{1}H\}$ NMR Spectrum of Compound **40** (100 MHz, $CDCl_3$)

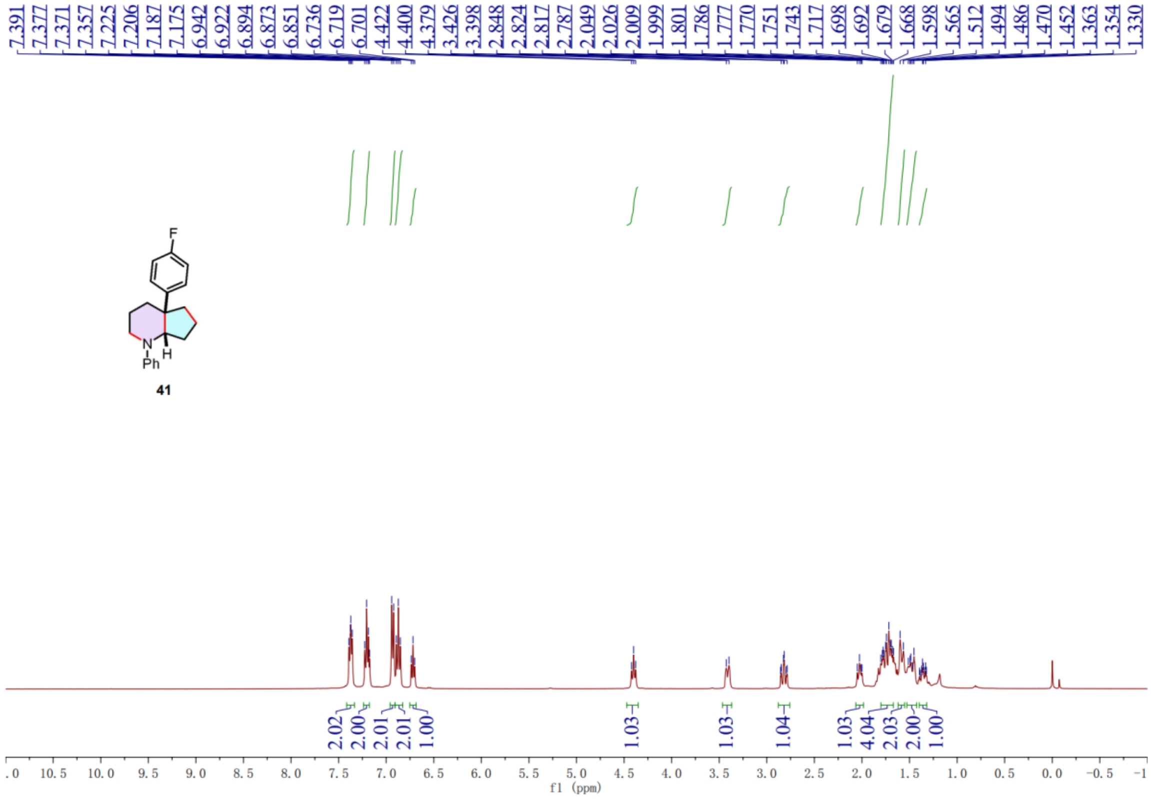

^{1}H NMR Spectrum of Compound **41** (400 MHz, $CDCl_3$)

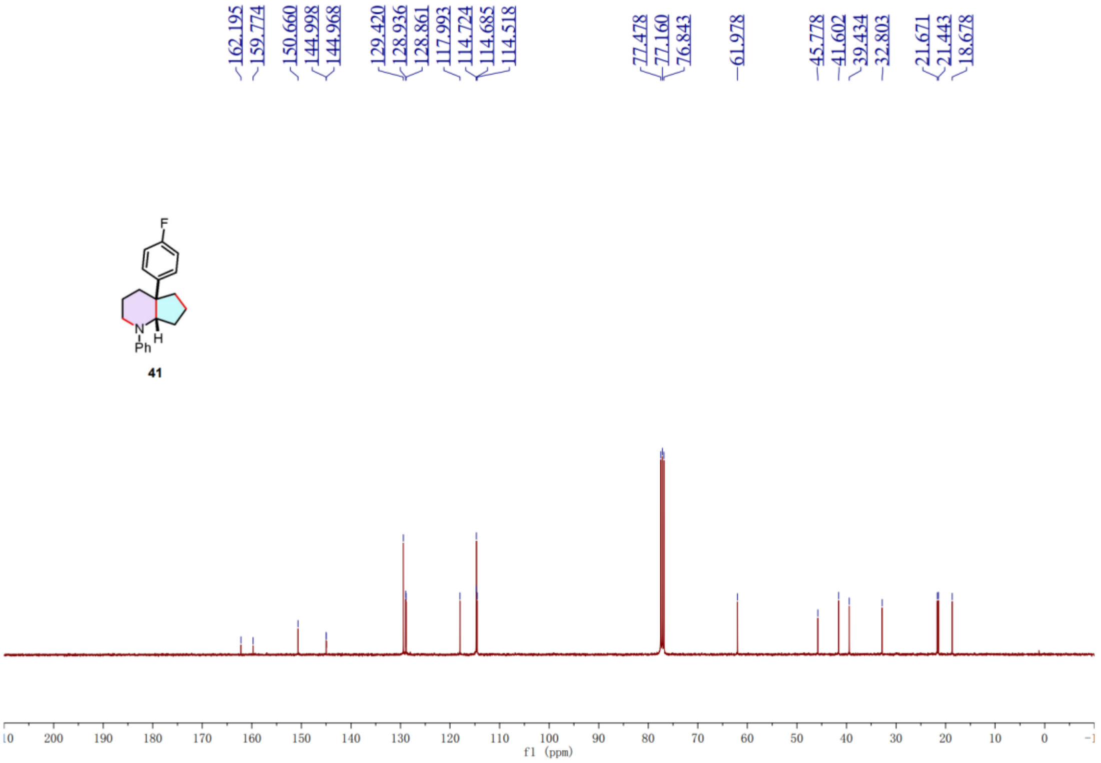

$^{13}C\{^1H\}$ NMR Spectrum of Compound **41** (100 MHz, $CDCl_3$)

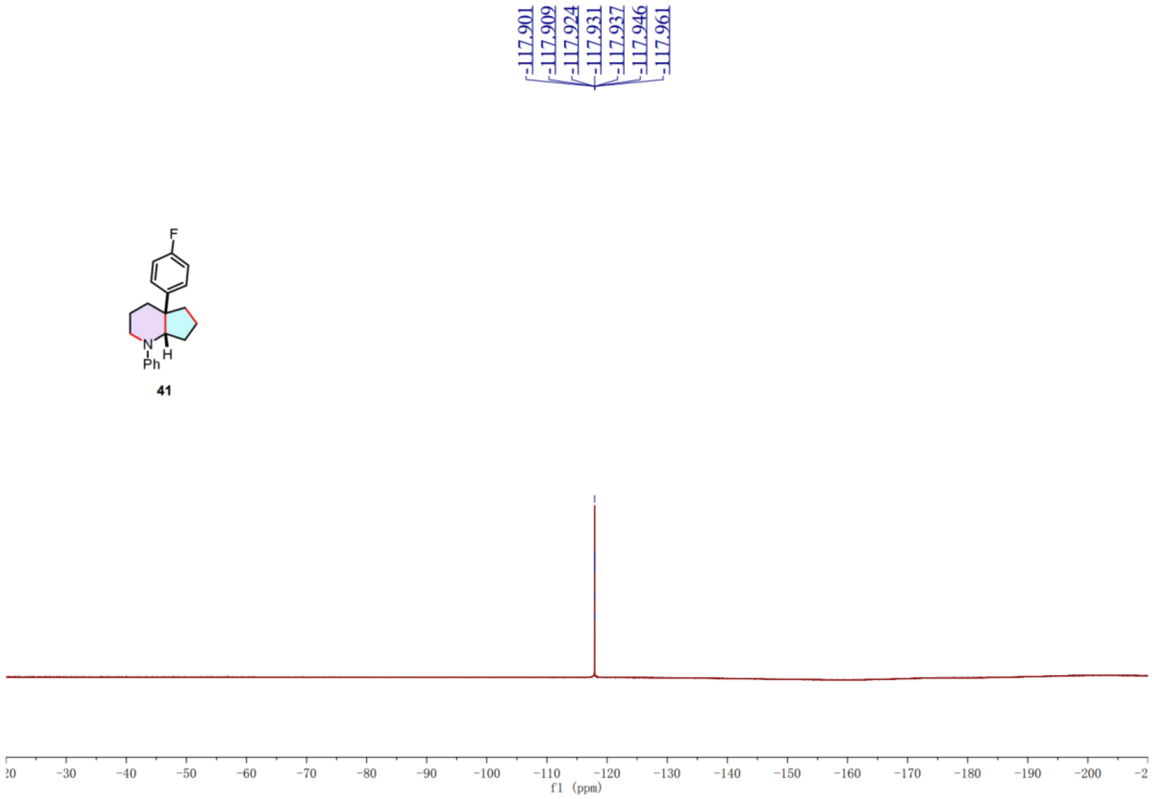

$^{19}F\{^1H\}$ NMR Spectrum of Compound **41** (376 MHz, $CDCl_3$)

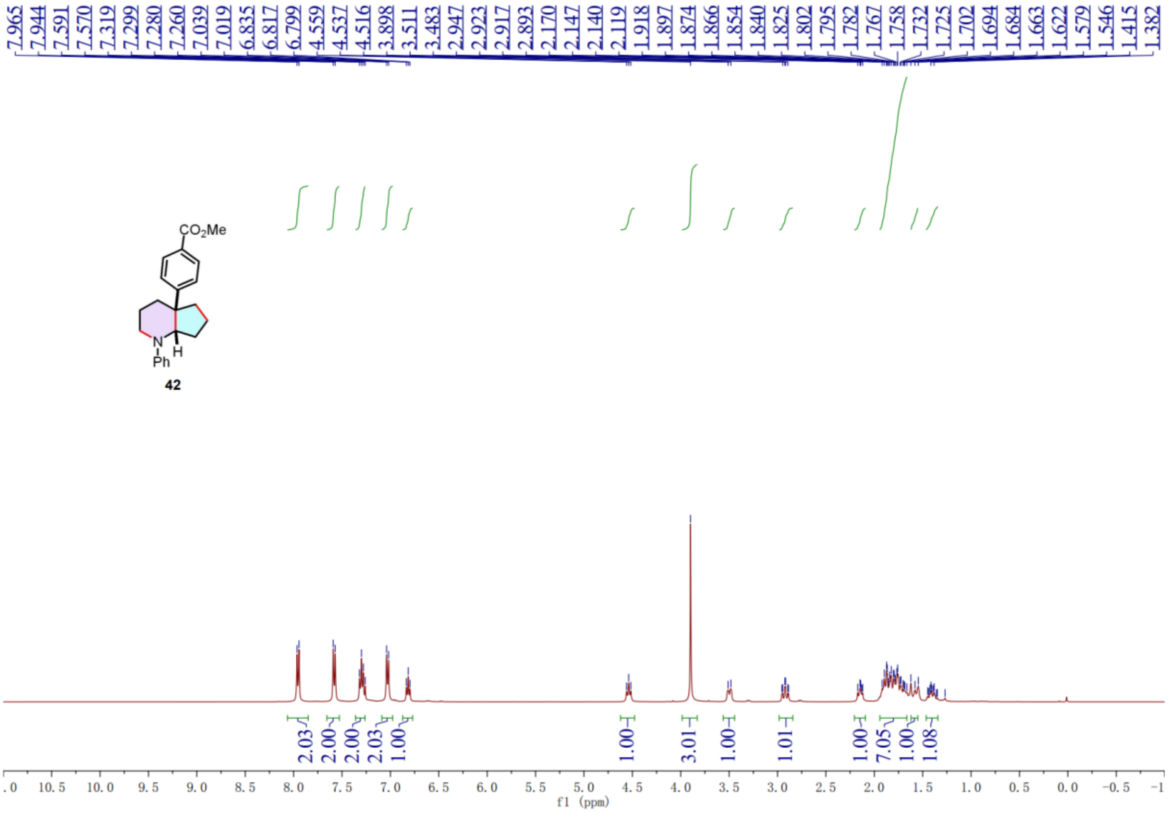

^{1}H NMR Spectrum of Compound **42** (400 MHz, $CDCl_3$)

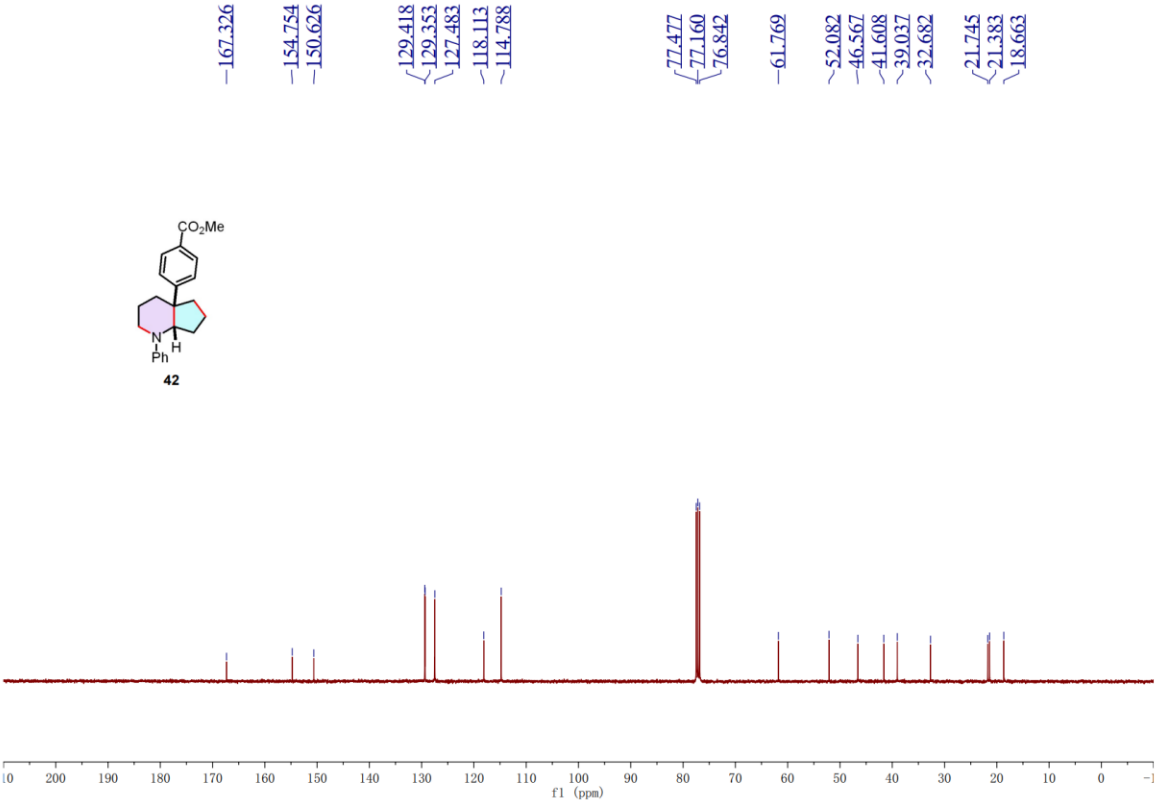

^{13}C{^{1}H} NMR Spectrum of Compound **42** (100 MHz, $CDCl_3$)

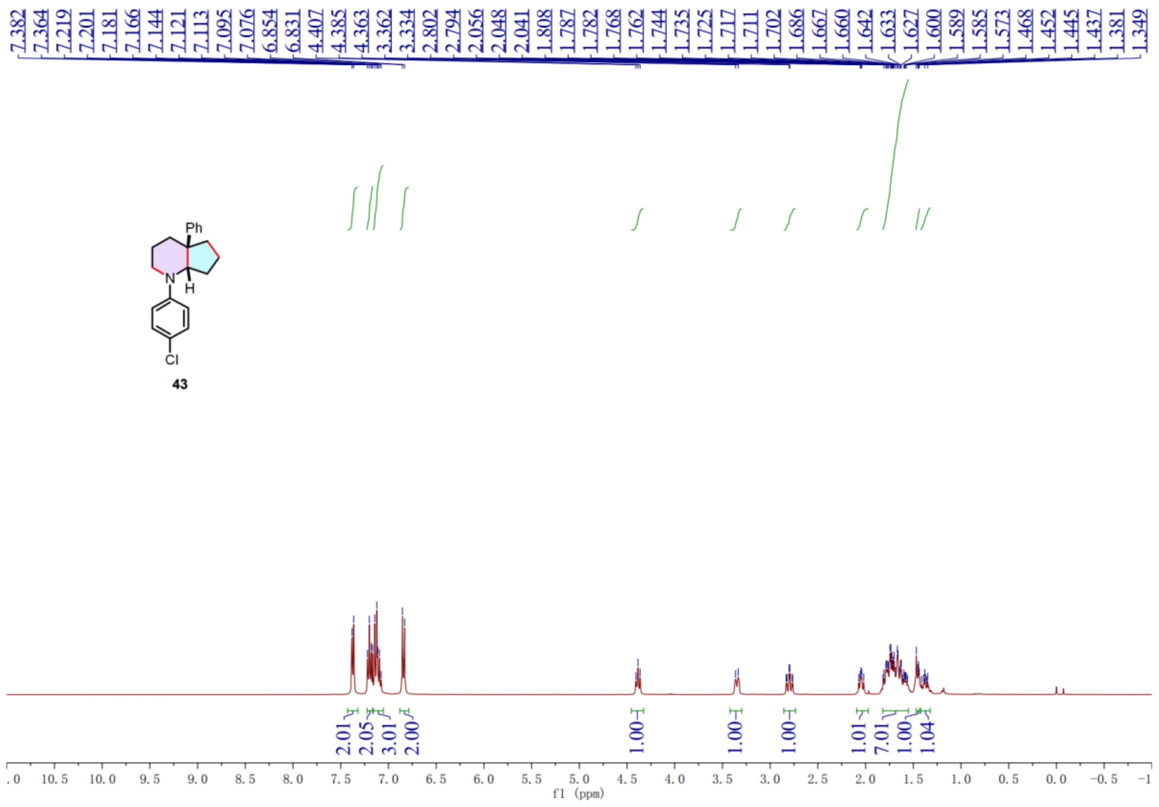

^{1}H NMR Spectrum of Compound **43** (400 MHz, $CDCl_3$)

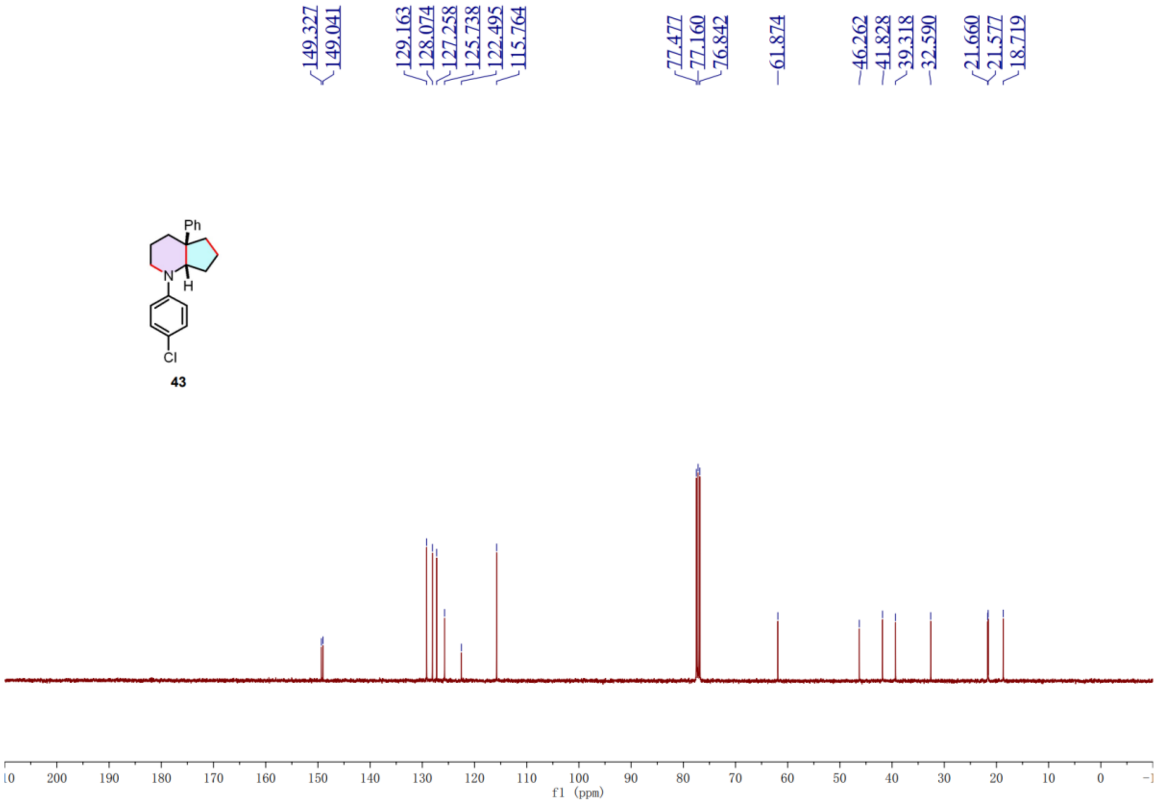

$^{13}C\{^{1}H\}$ NMR Spectrum of Compound **43** (100 MHz, $CDCl_3$)

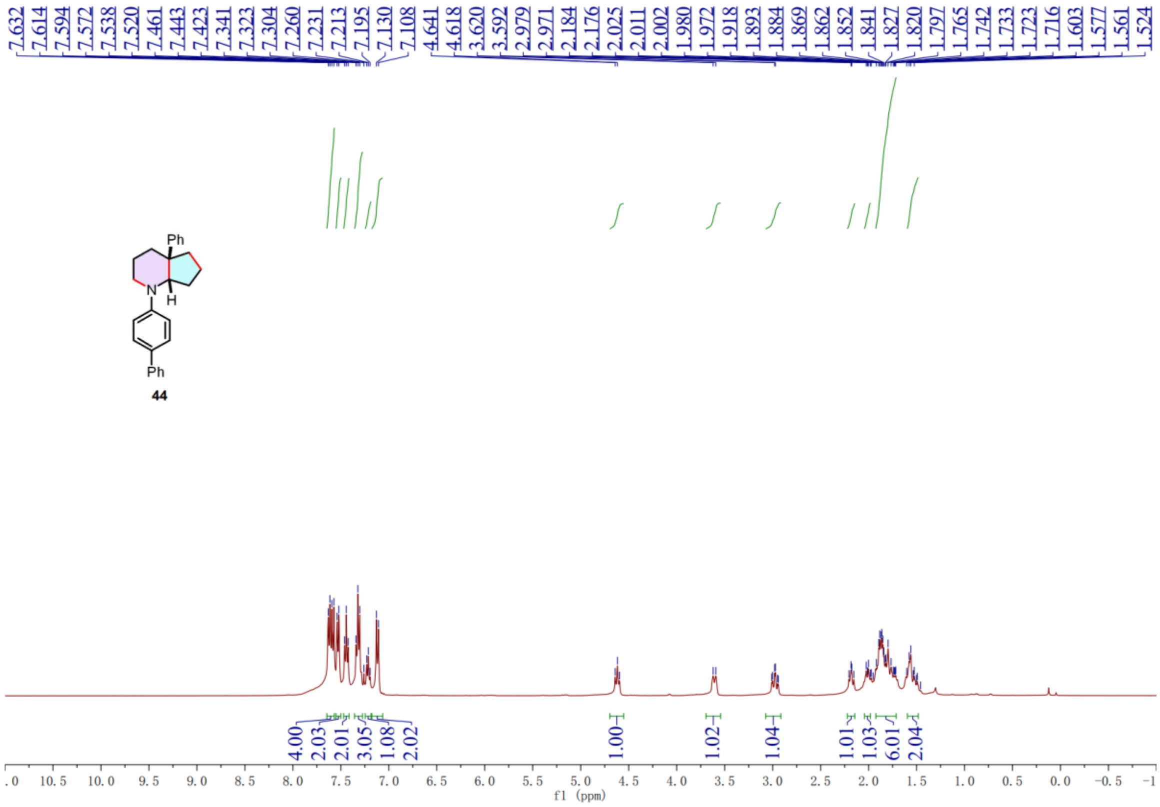

^{1}H NMR Spectrum of Compound **44** (400 MHz, $CDCl_3$)

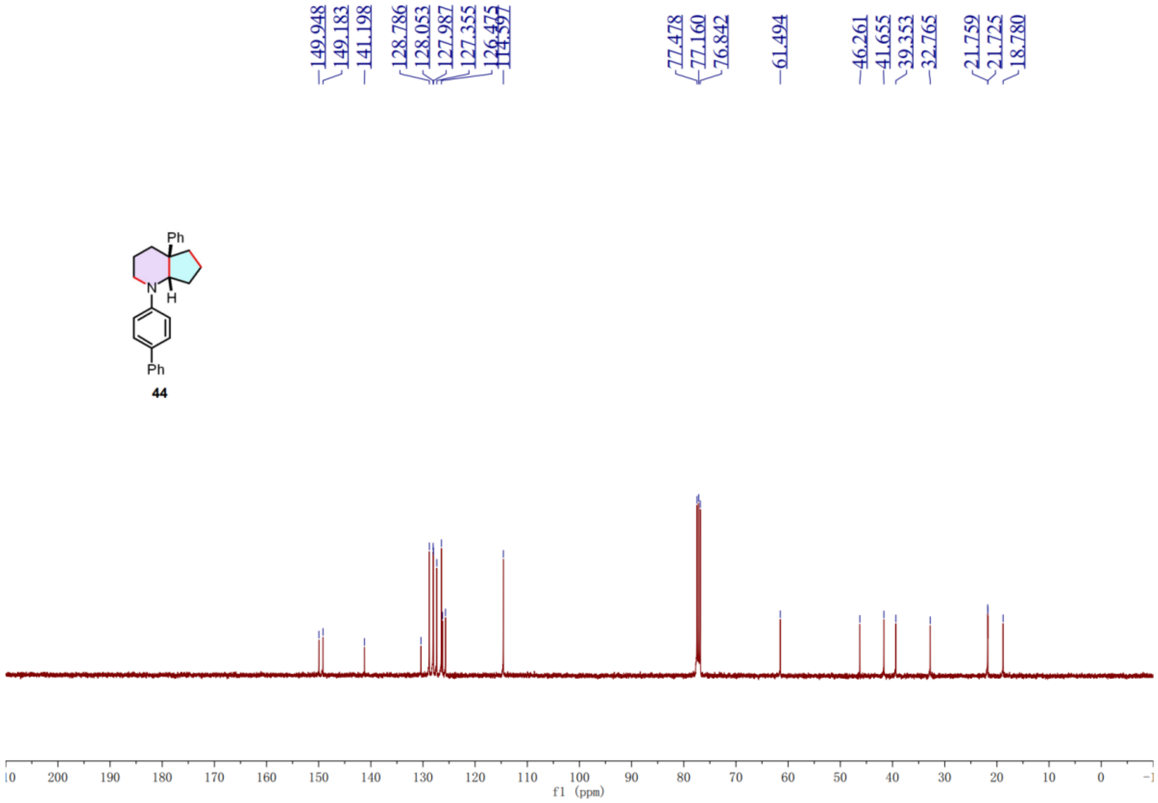

$^{13}C\{^{1}H\}$ NMR Spectrum of Compound **44** (100 MHz, $CDCl_3$)

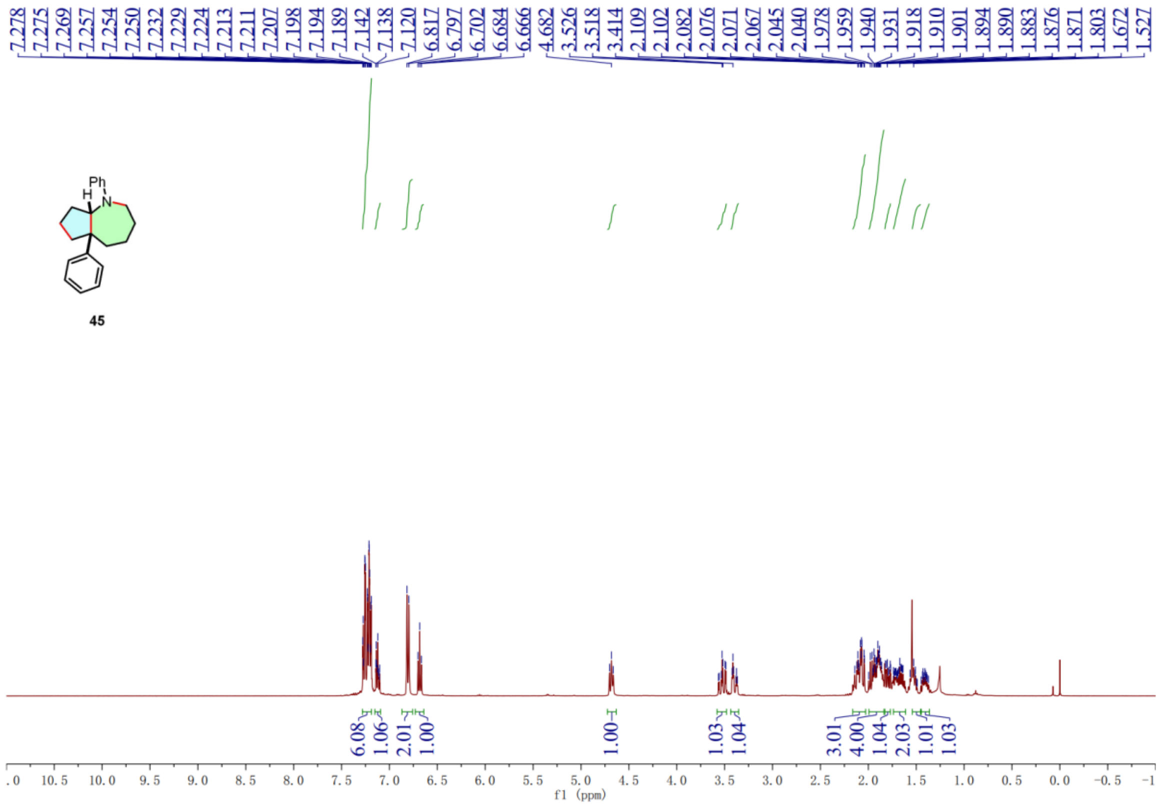

^{1}H NMR Spectrum of Compound **45** (400 MHz, $CDCl_3$)

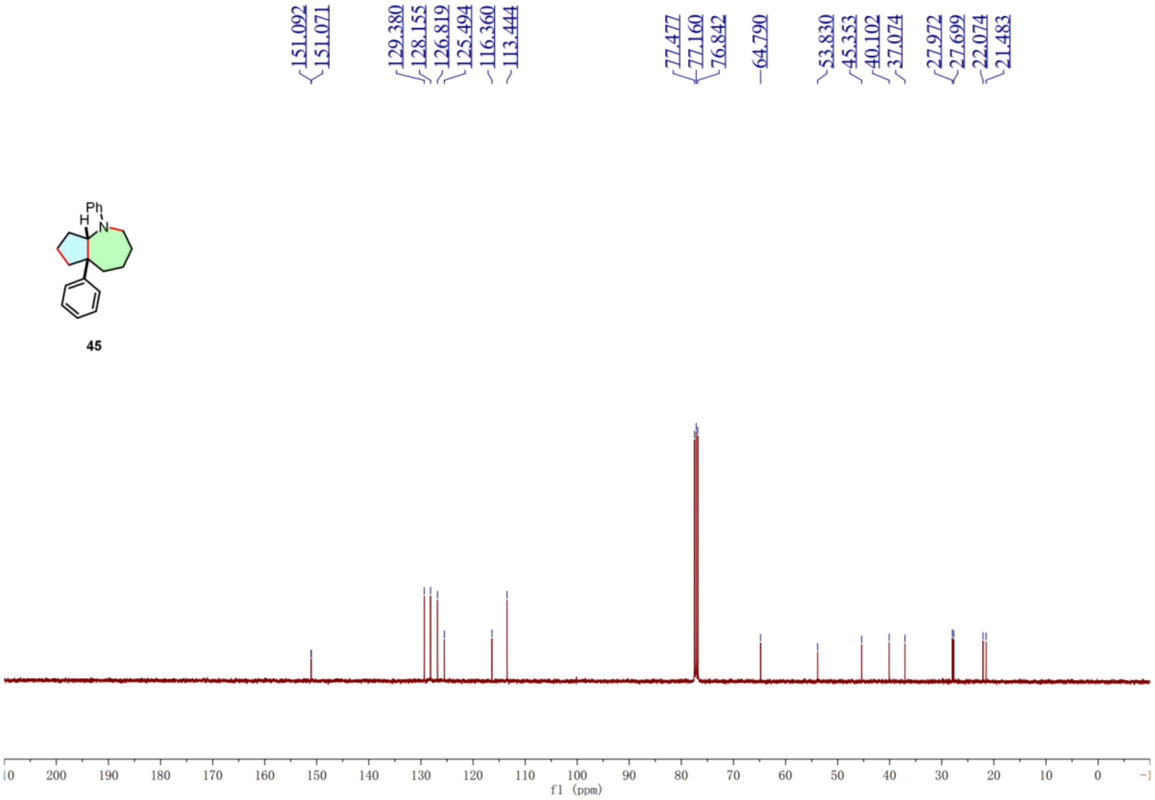

^{13}C{^{1}H} NMR Spectrum of Compound **45** (100 MHz, $CDCl_3$)

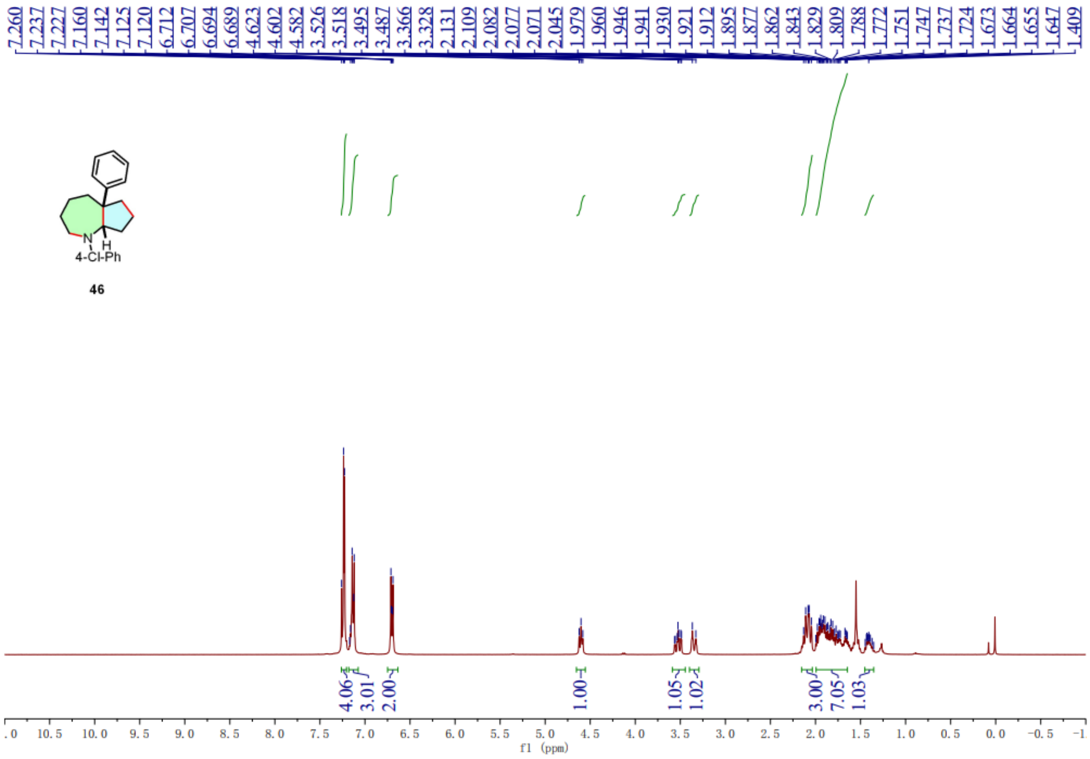

^{1}H NMR Spectrum of Compound **46** (400 MHz, $CDCl_3$)

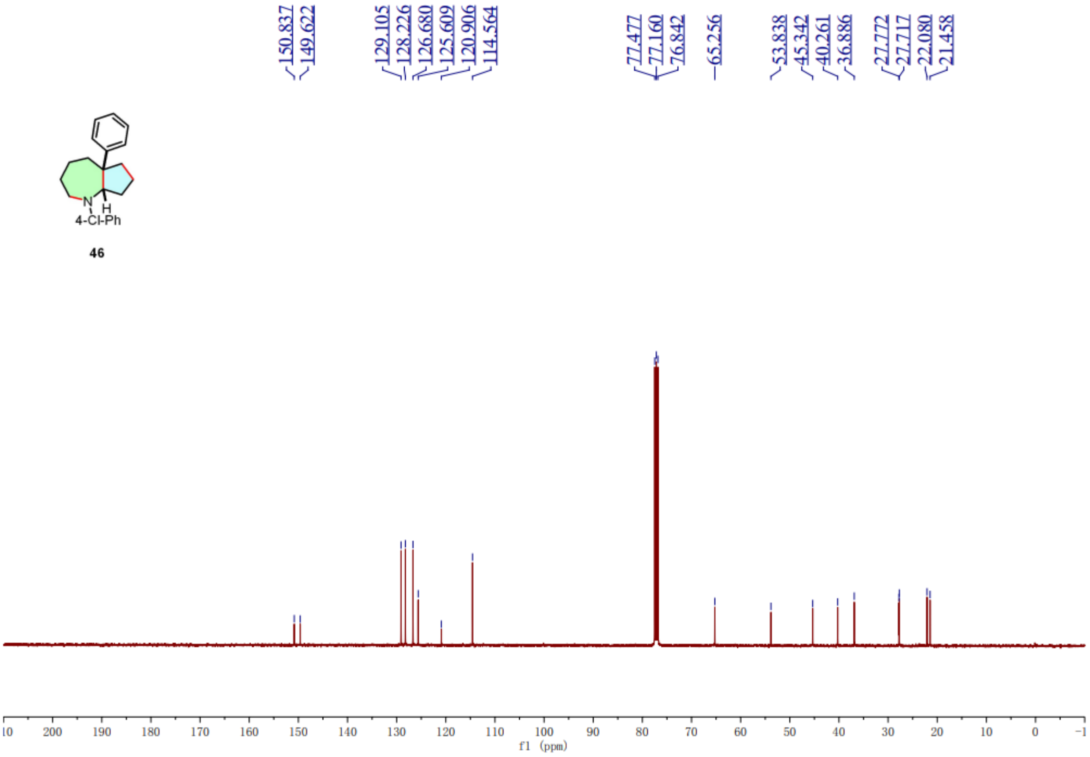

$^{13}C\{^{1}H\}$ NMR Spectrum of Compound **46** (100 MHz, $CDCl_3$)

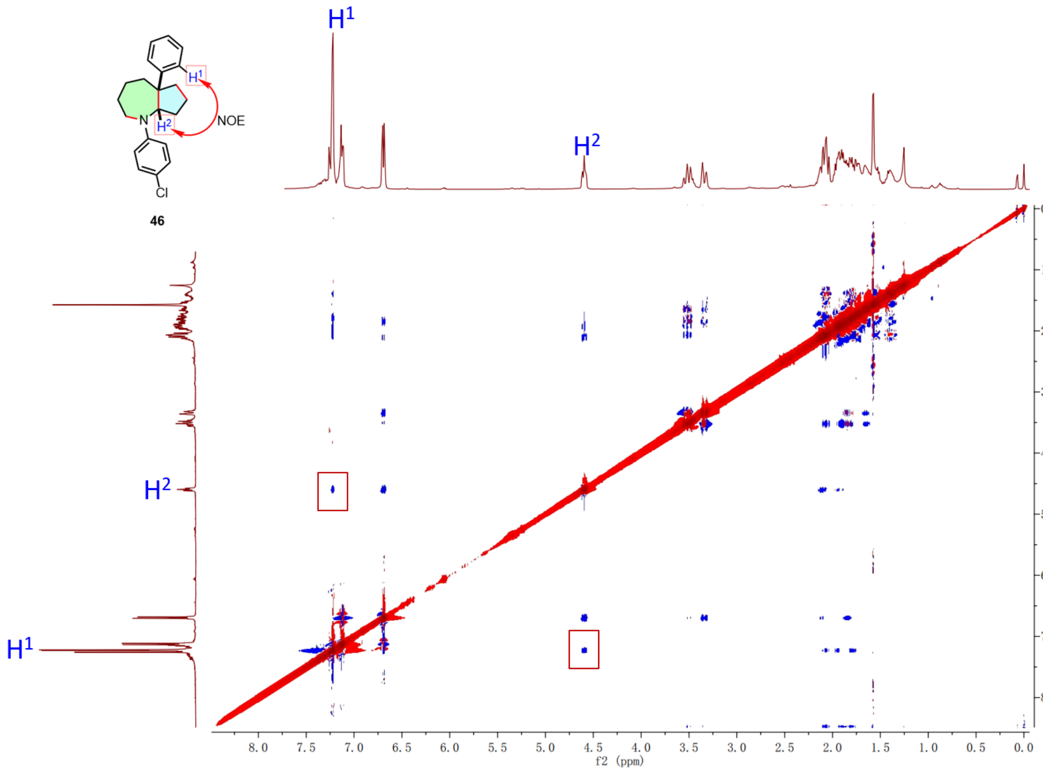

NOE NMR Spectrum of Compound **46** (100 MHz, $CDCl_3$)

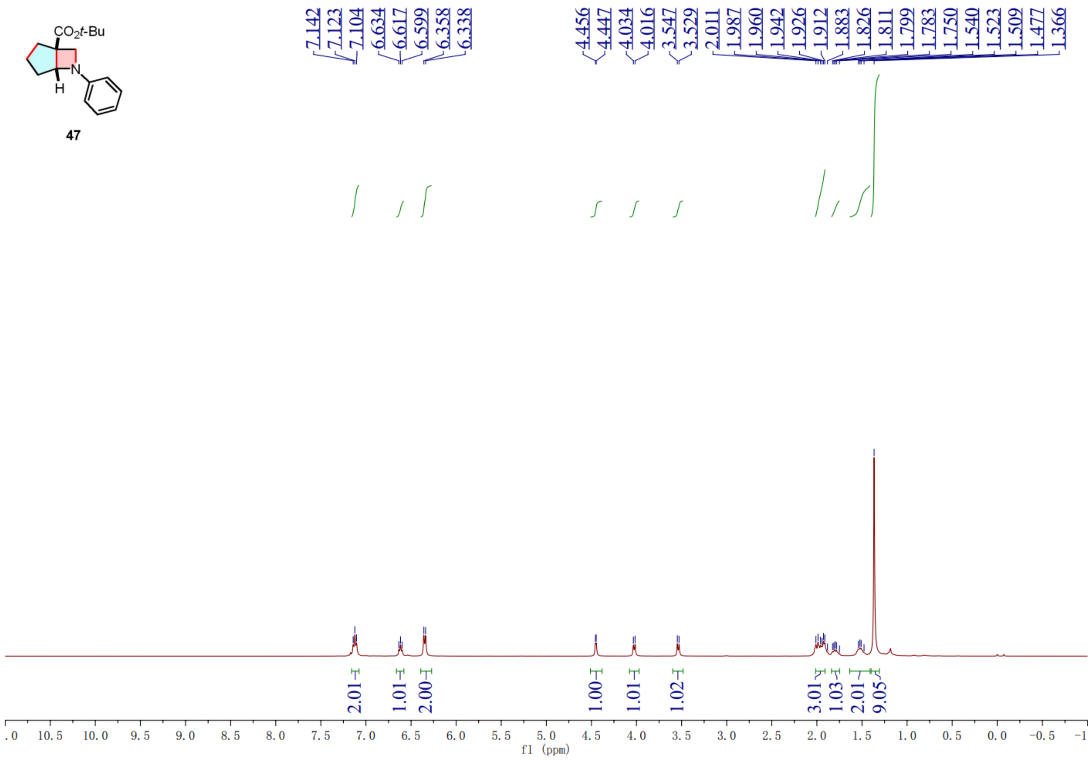

^{1}H NMR Spectrum of Compound **47** (400 MHz, $CDCl_3$)

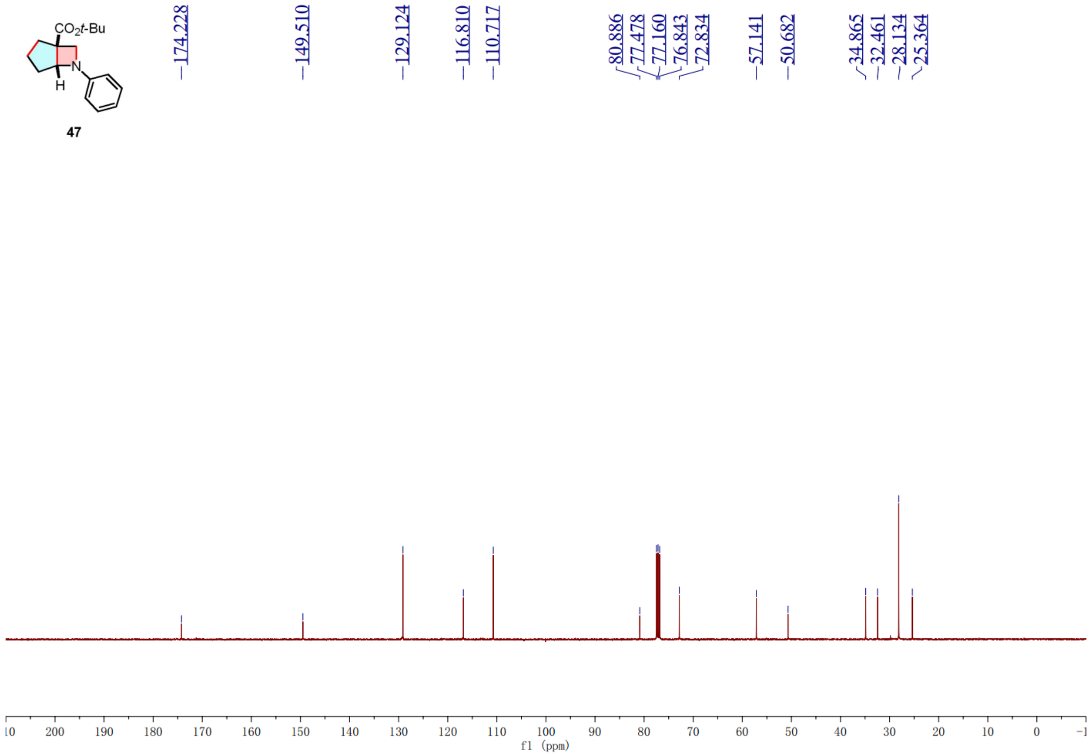

$^{13}C\{^{1}H\}$ NMR Spectrum of Compound **47** (100 MHz, $CDCl_3$)

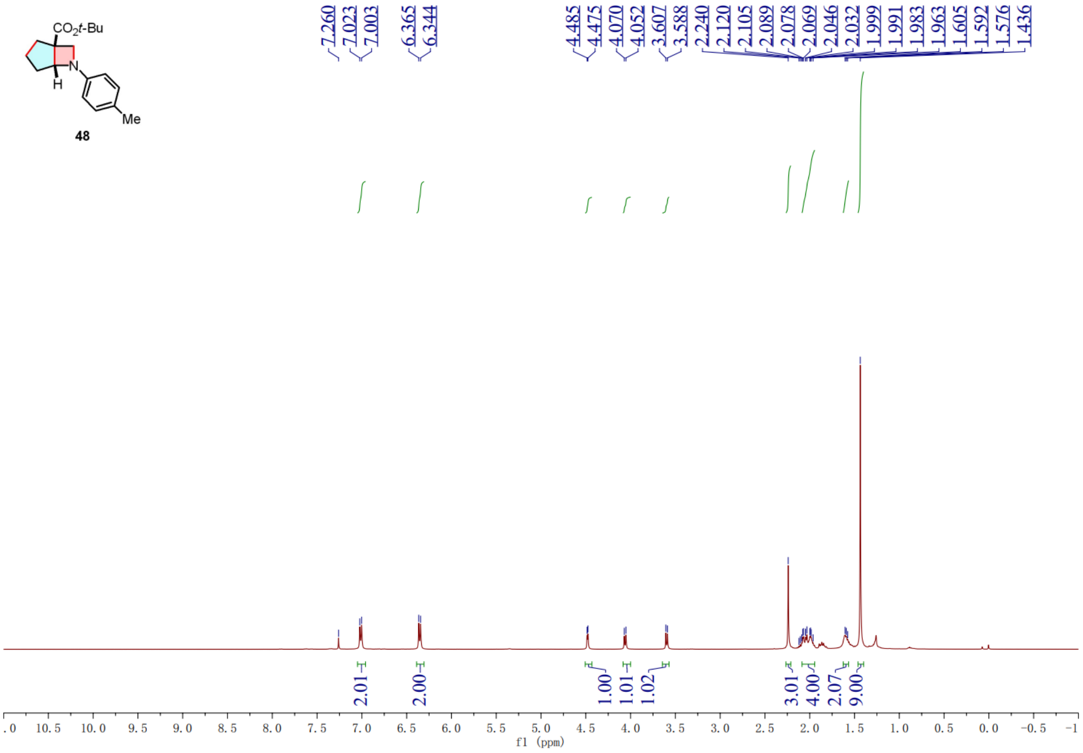

^{1}H NMR Spectrum of Compound **48** (400 MHz, $CDCl_3$)

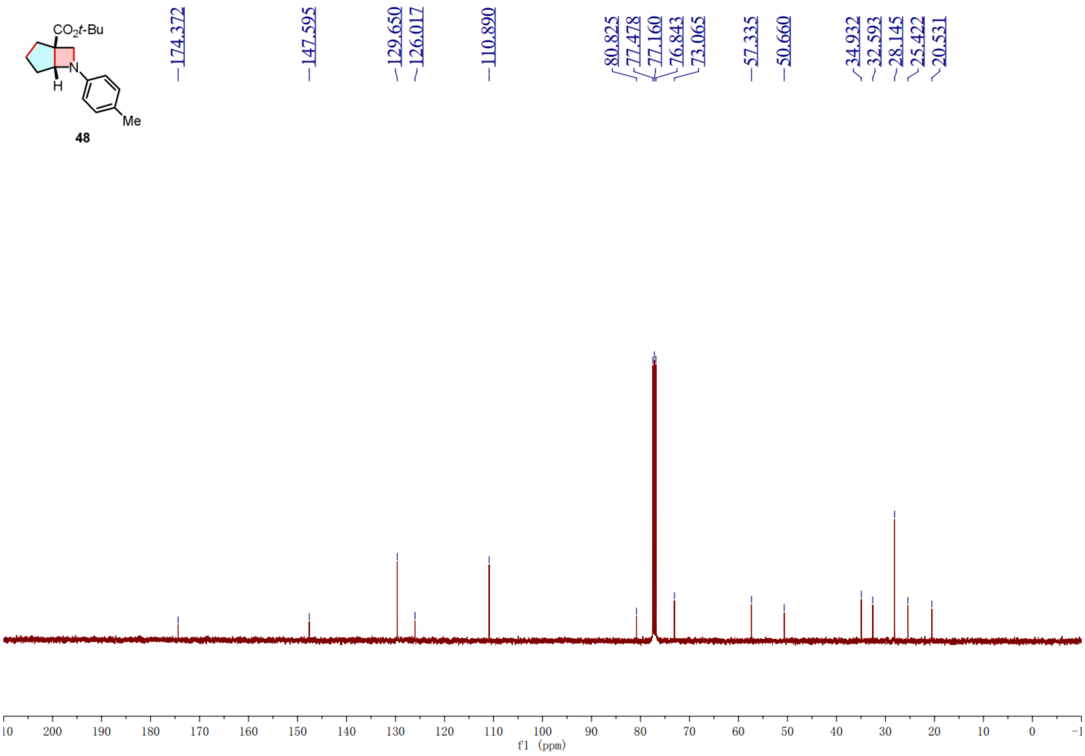

^{13}C{^{1}H} NMR Spectrum of Compound **48** (100 MHz, $CDCl_3$)

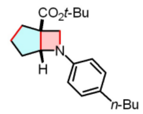

49

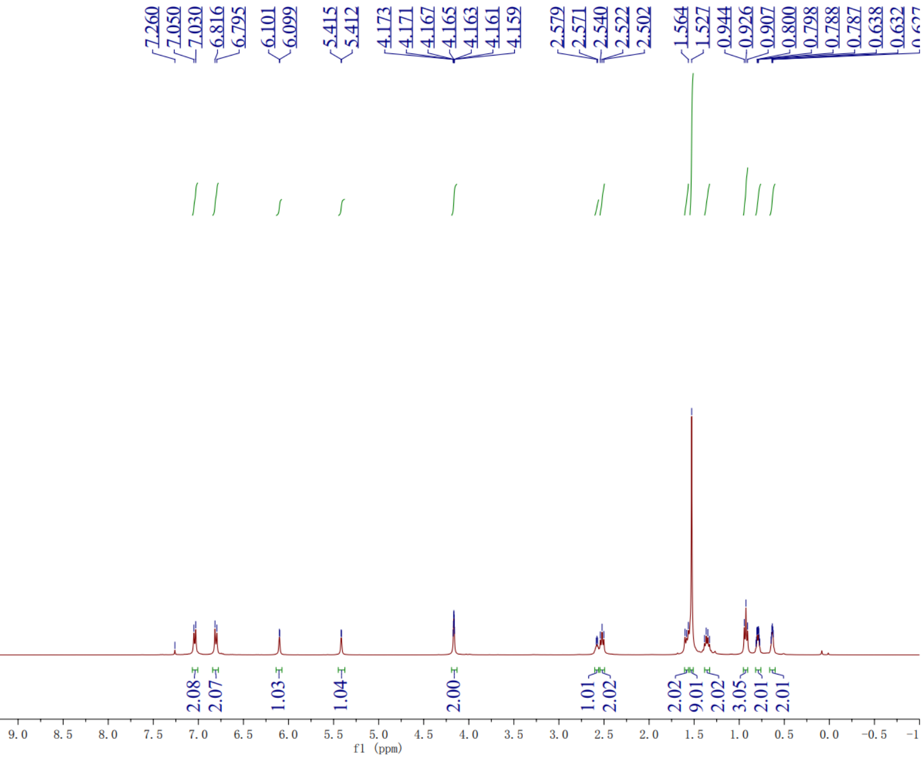

^{1}H NMR Spectrum of Compound **49** (400 MHz, $CDCl_3$)

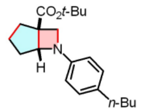

49

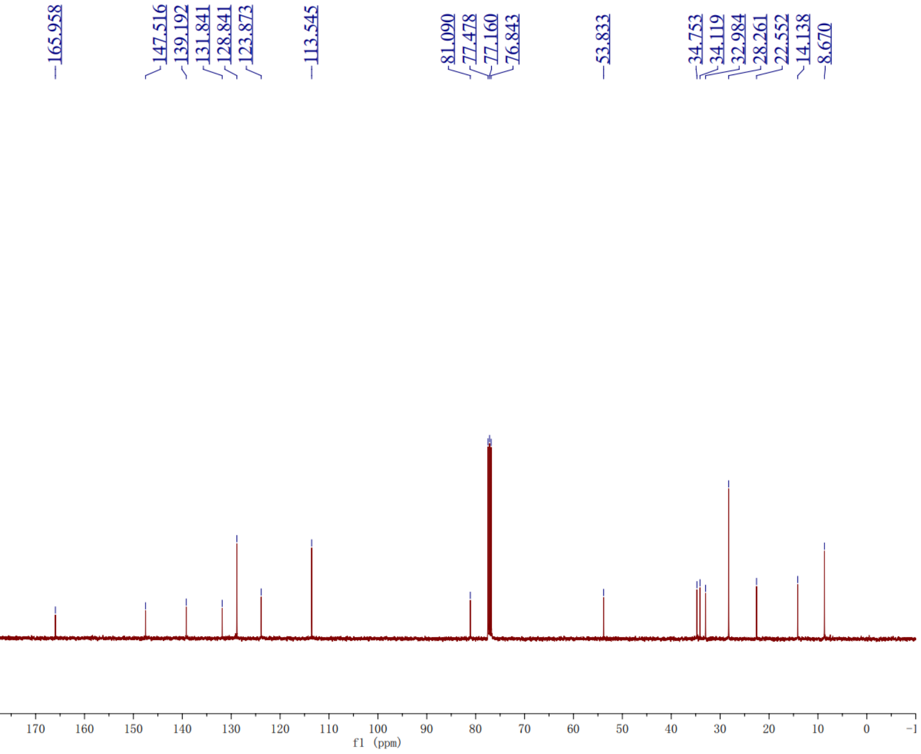

$^{13}C\{^1H\}$ NMR Spectrum of Compound **49** (100 MHz, $CDCl_3$)

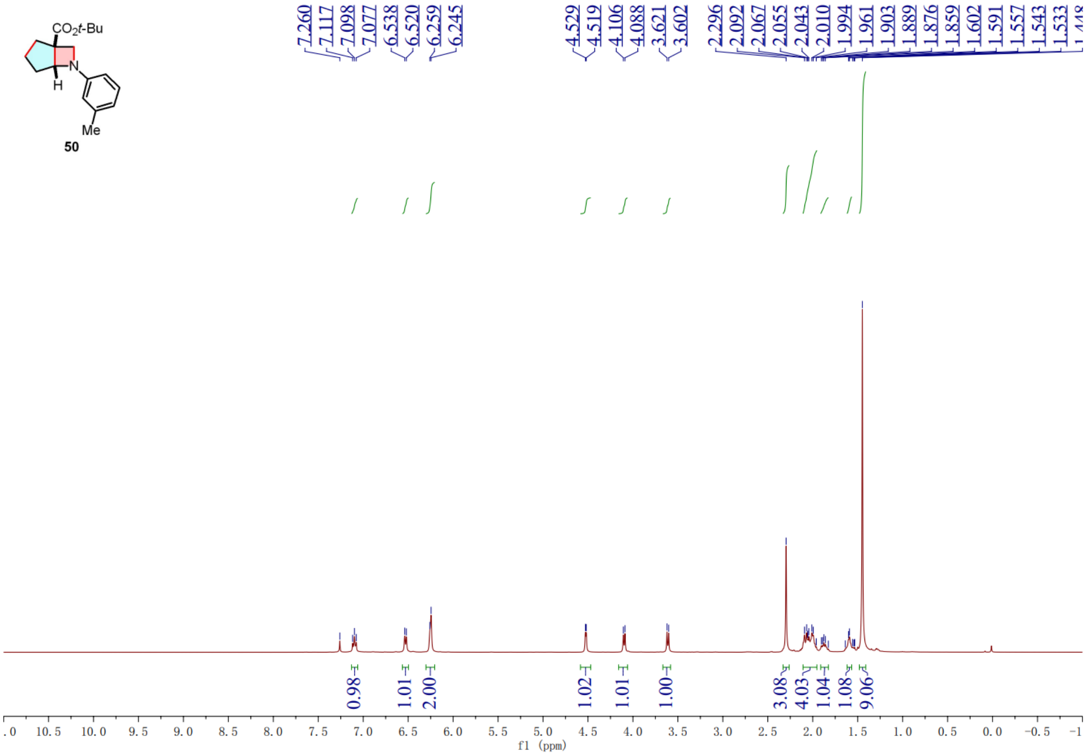

^{1}H NMR Spectrum of Compound **50** (400 MHz, $CDCl_3$)

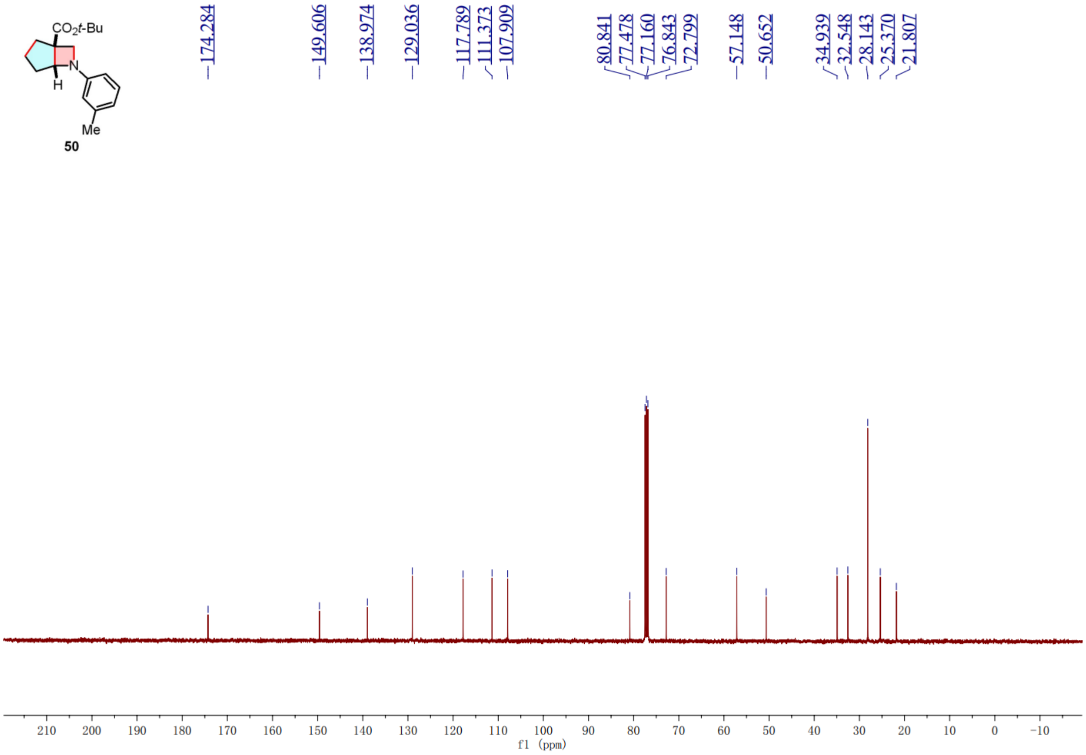

$^{13}C\{^{1}H\}$ NMR Spectrum of Compound **50** (100 MHz, $CDCl_3$)

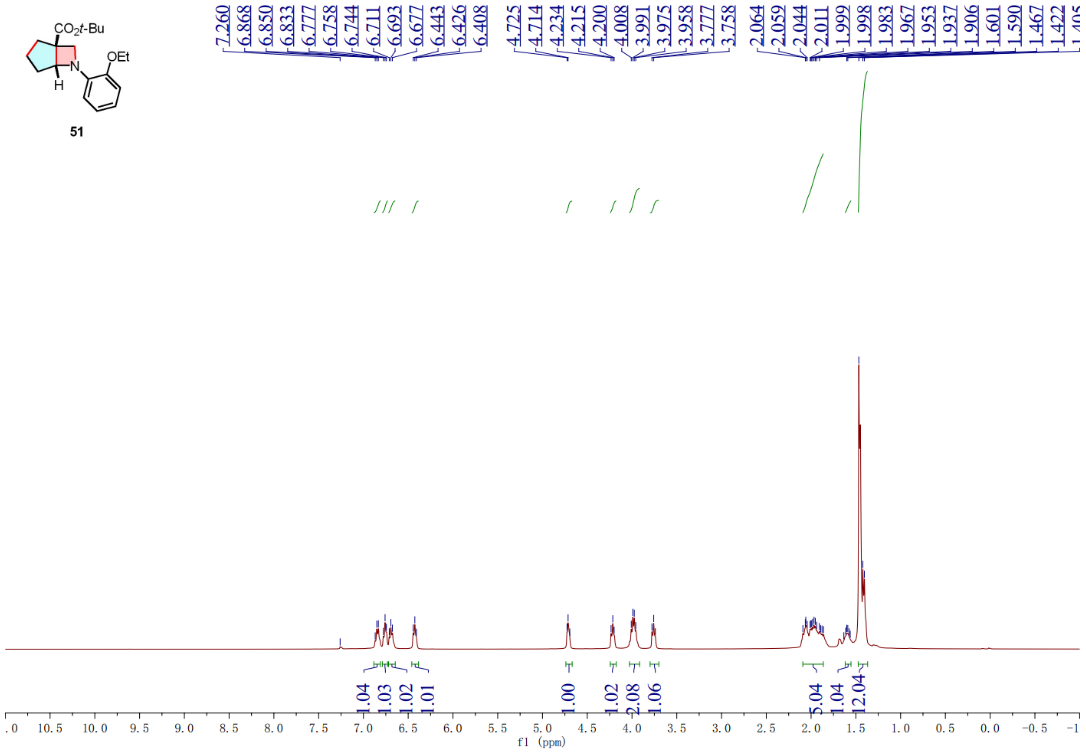

^{1}H NMR Spectrum of Compound **51** (400 MHz, $CDCl_3$)

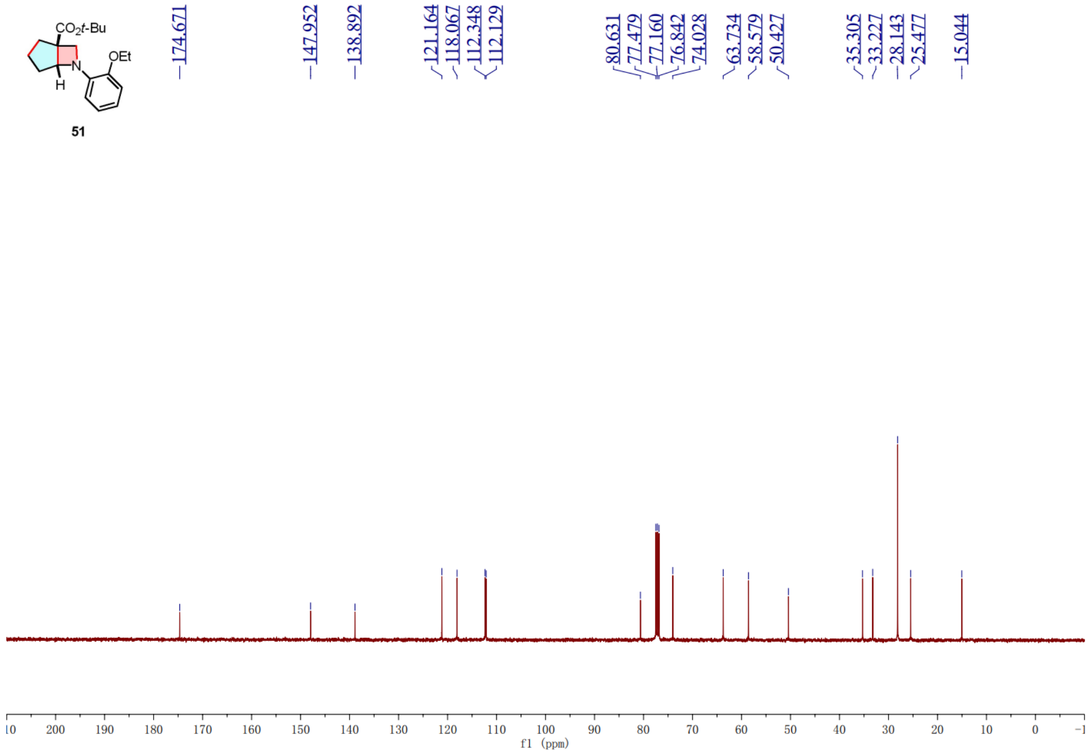

^{13}C{^{1}H} NMR Spectrum of Compound **51** (100 MHz, $CDCl_3$)

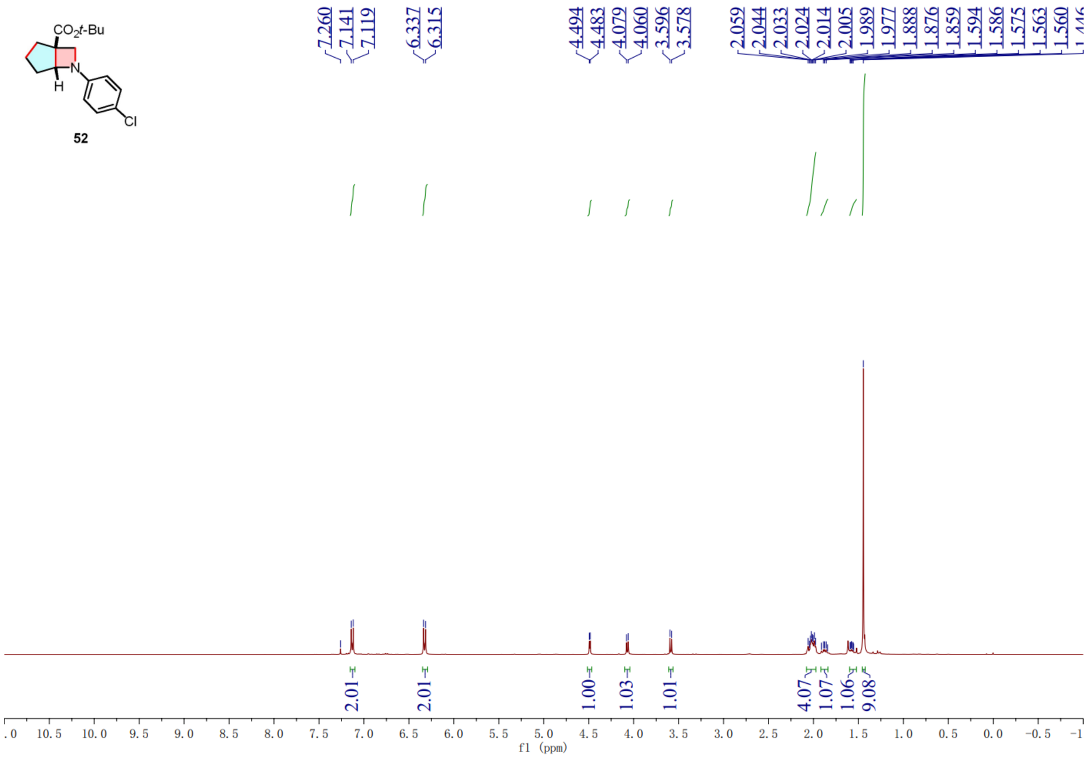

^{1}H NMR Spectrum of Compound **52** (400 MHz, $CDCl_3$)

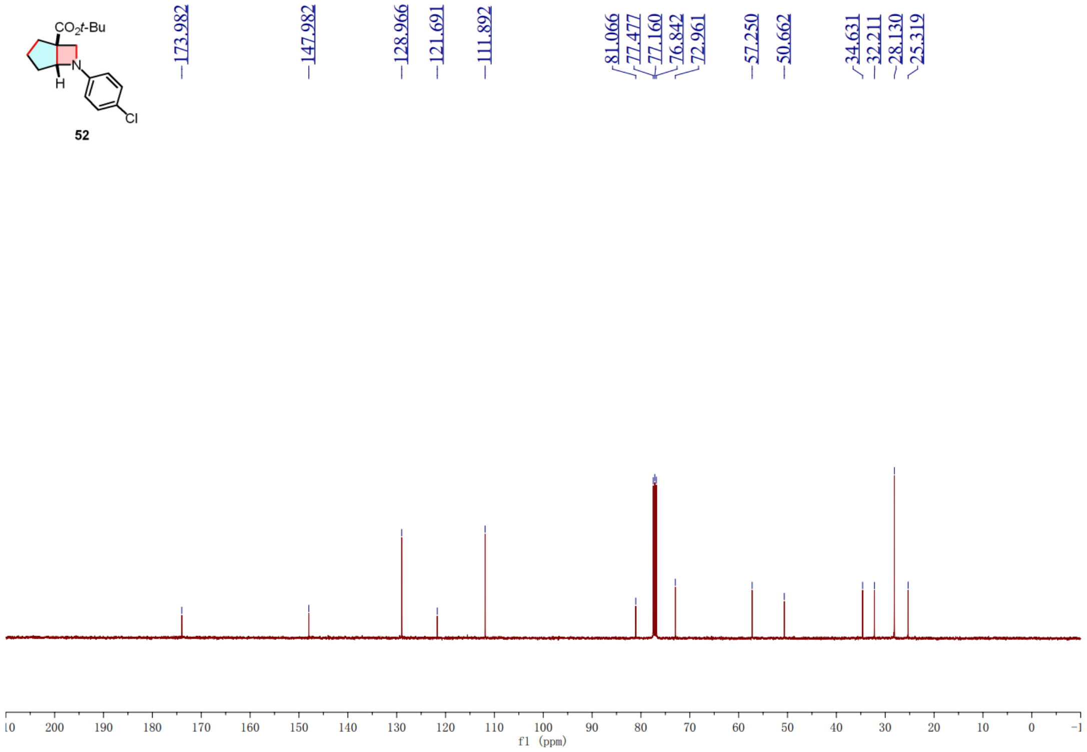

$^{13}C\{^{1}H\}$ NMR Spectrum of Compound **52** (100 MHz, $CDCl_3$)

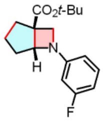

53

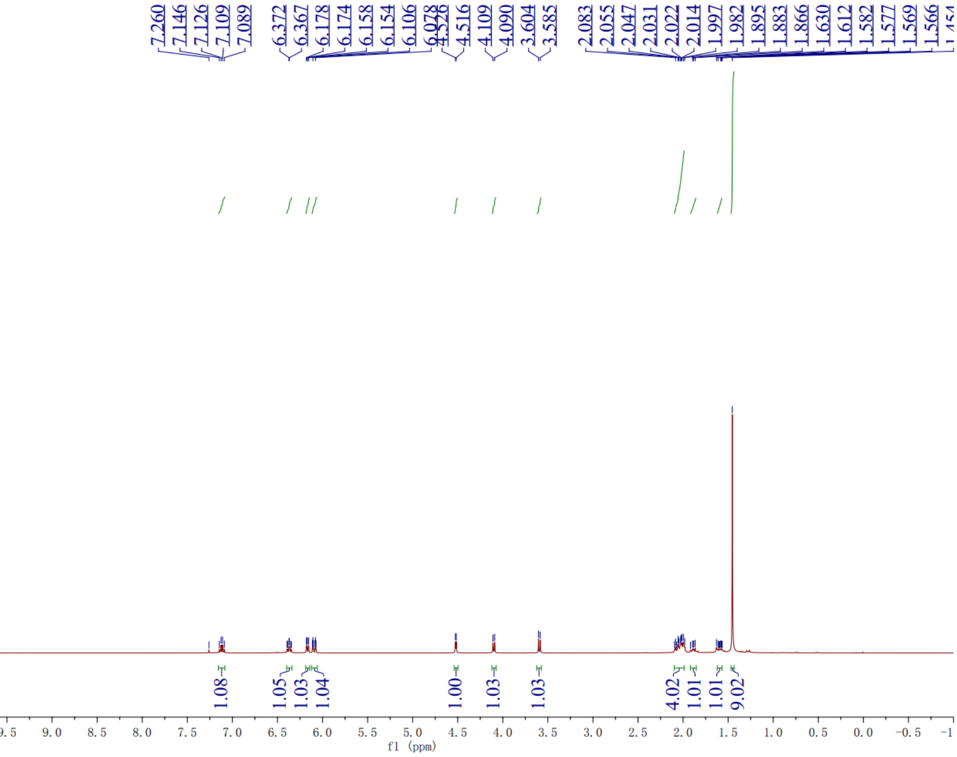

^{1}H NMR Spectrum of Compound **53** (400 MHz, $CDCl_3$)

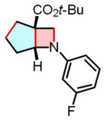

53

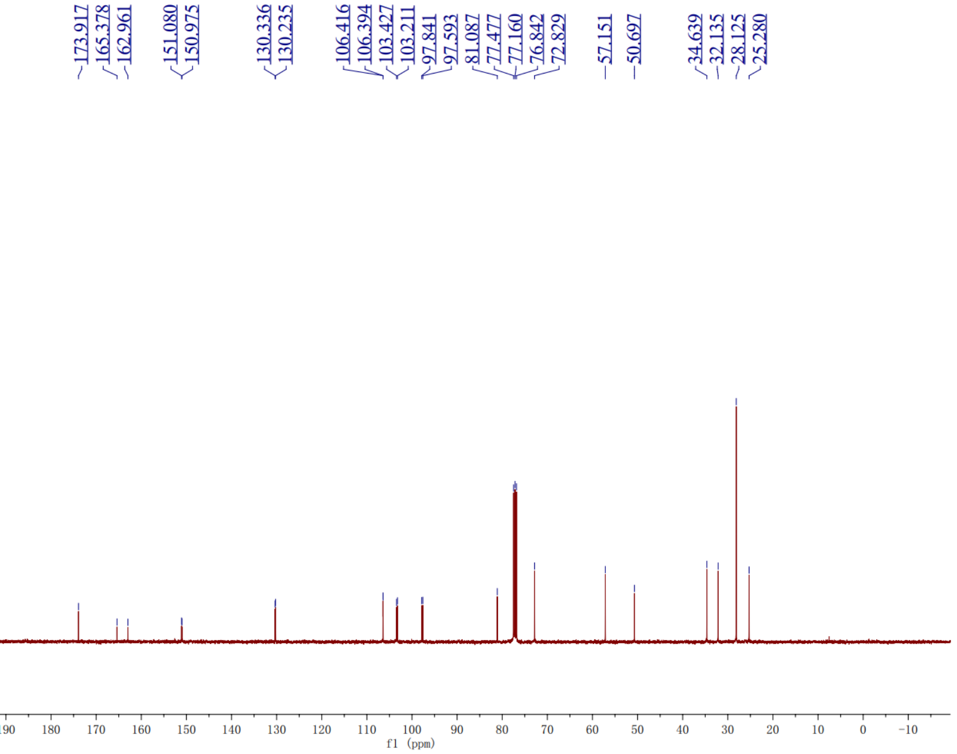

^{13}C{^{1}H} NMR Spectrum of Compound **53** (100 MHz, $CDCl_3$)

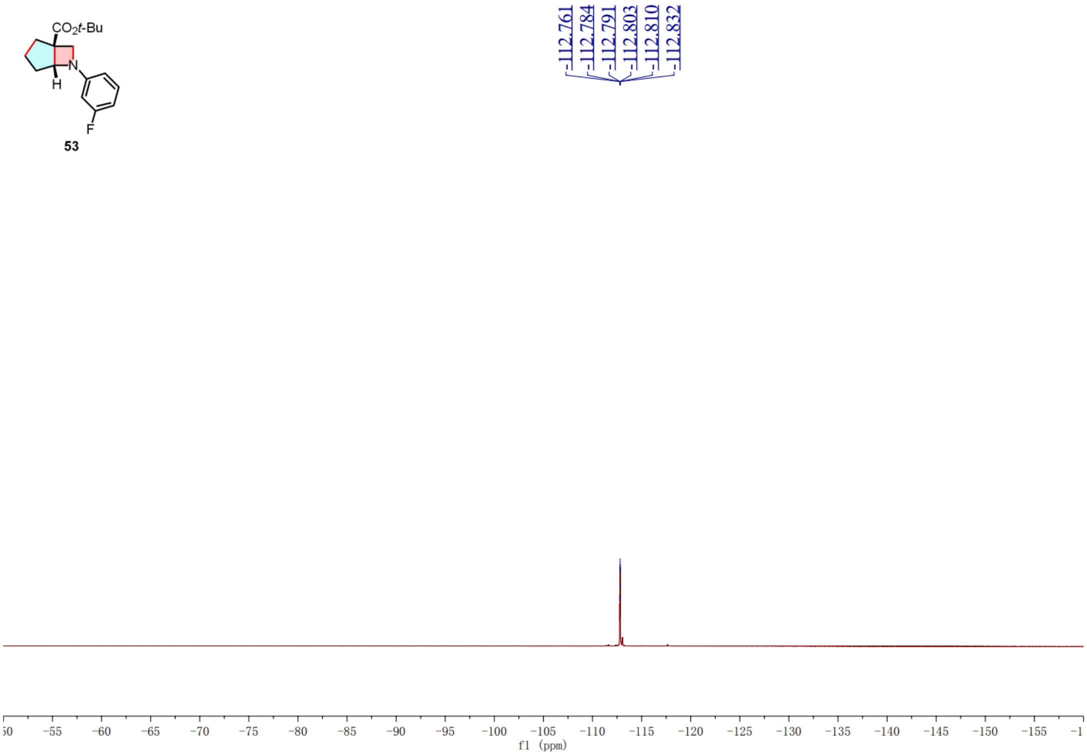

53

$^{19}F\{^{1}H\}$ NMR Spectrum of Compound **53** (376 MHz, $CDCl_3$)

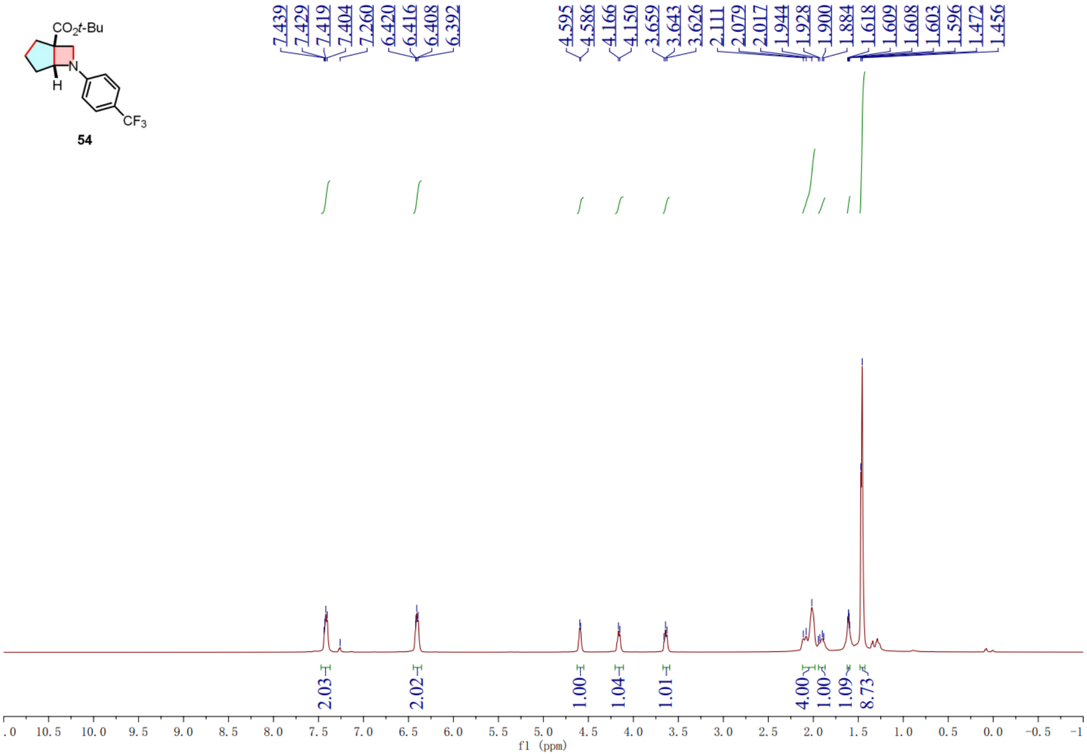

54

^{1}H NMR Spectrum of Compound **54** (400 MHz, $CDCl_3$)

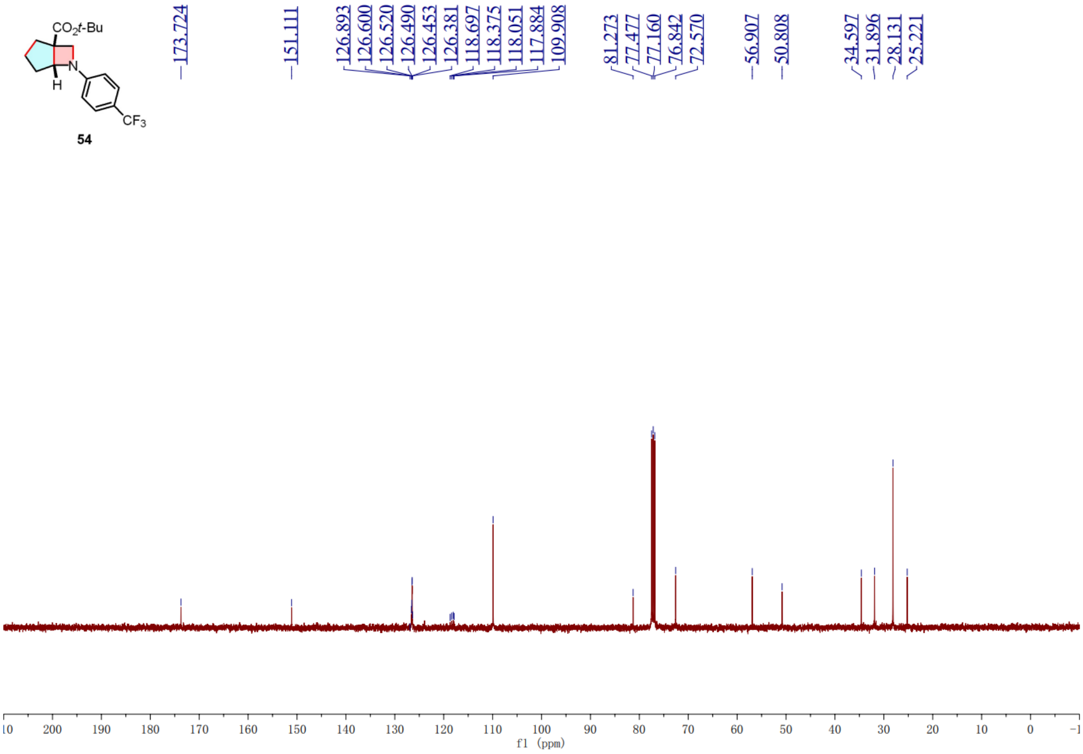

$^{13}C\{^{1}H\}$ NMR Spectrum of Compound **54** (100 MHz, $CDCl_3$)

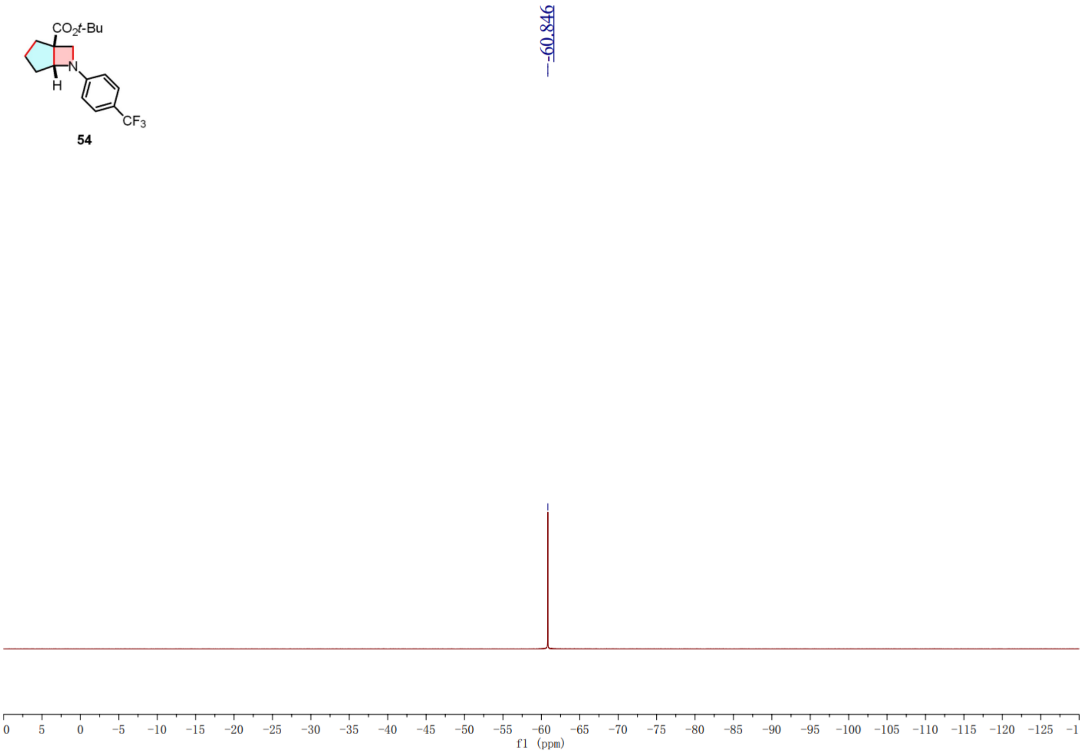

$^{19}F\{^{1}H\}$ NMR Spectrum of Compound **54** (376 MHz, $CDCl_3$)

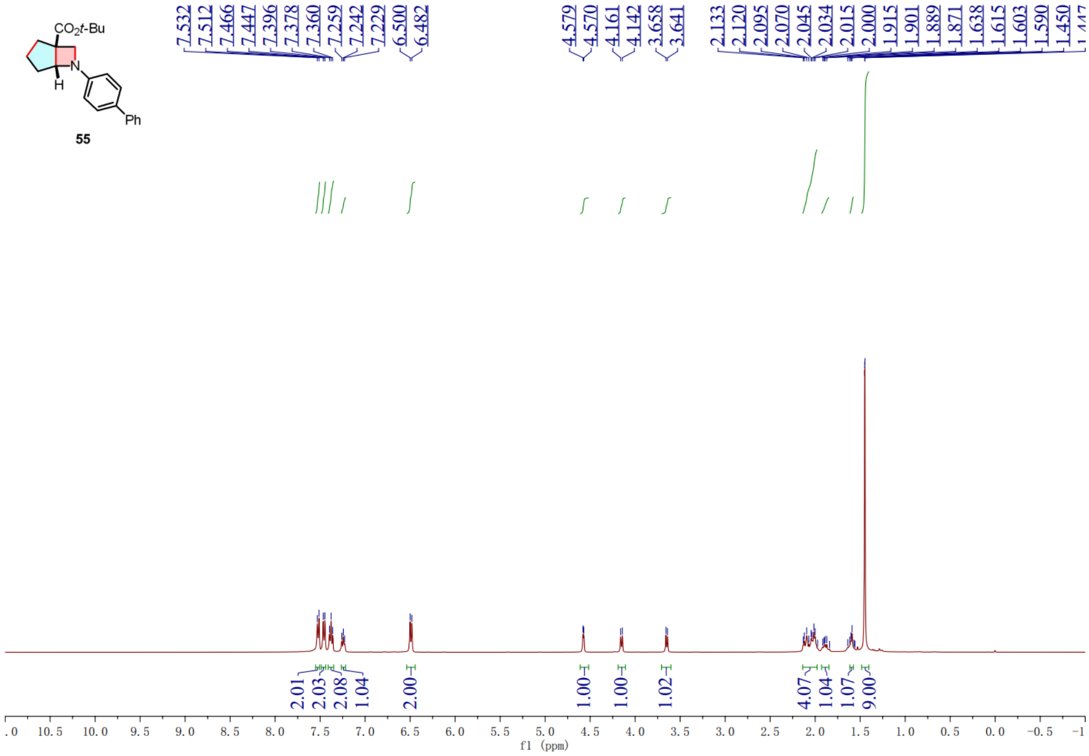

^{1}H NMR Spectrum of Compound **55** (400 MHz, $CDCl_3$)

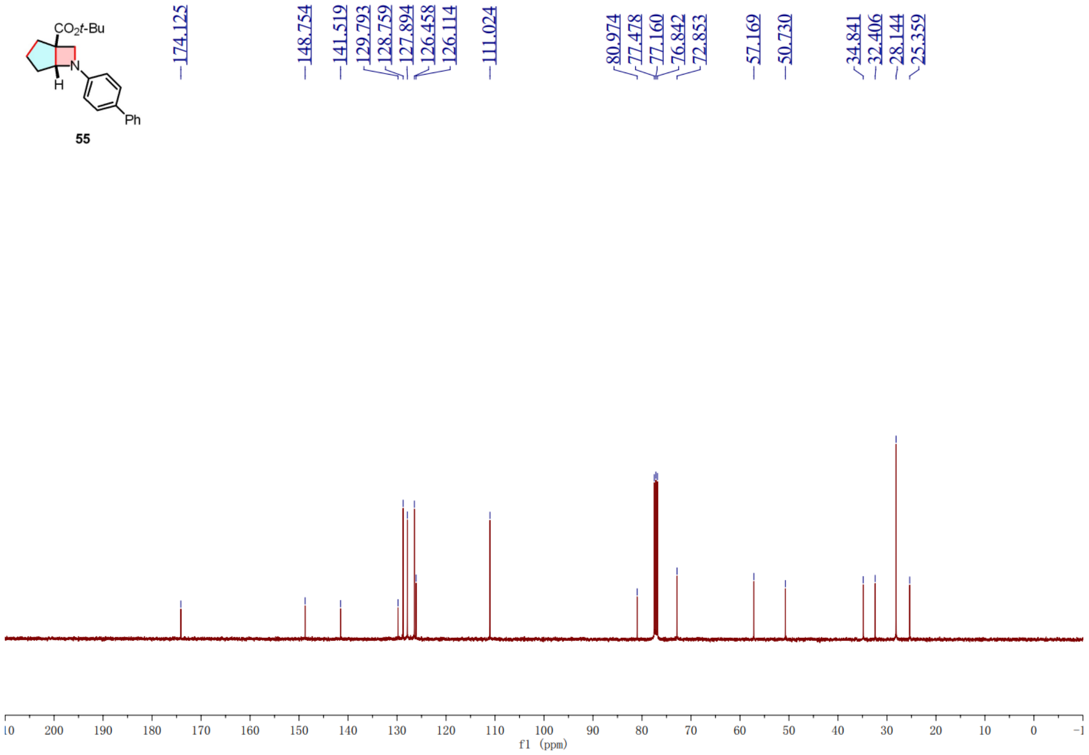

^{13}C{^{1}H} NMR Spectrum of Compound **55** (100 MHz, $CDCl_3$)

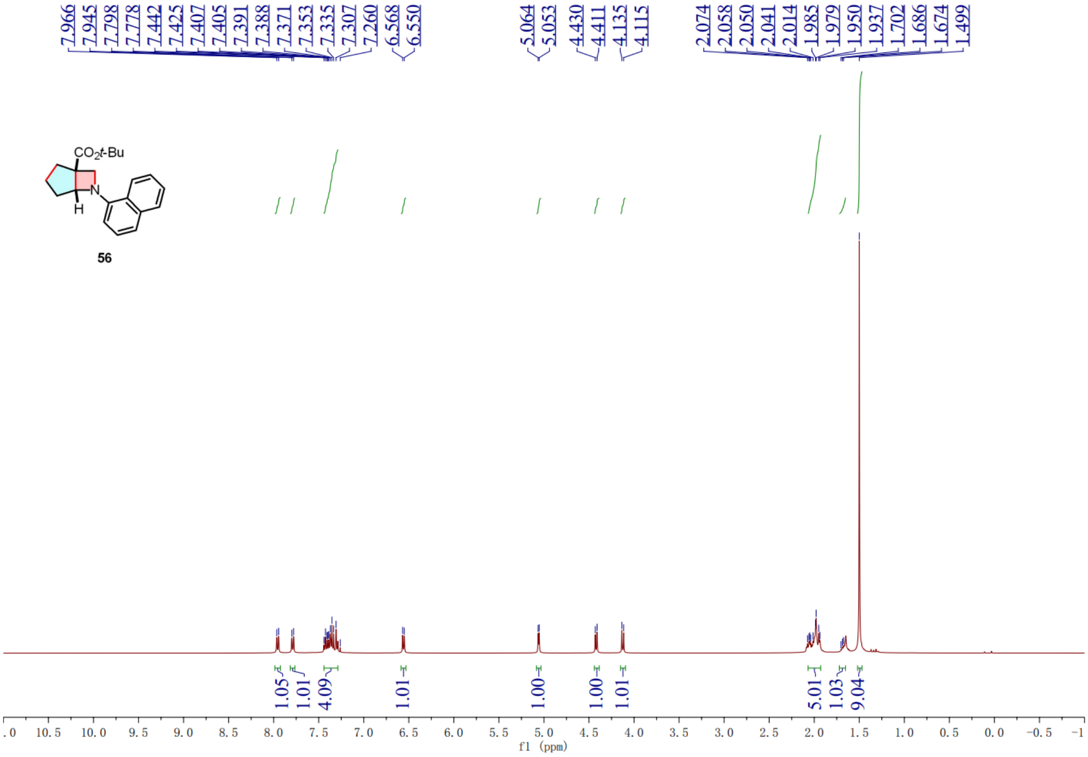

^{1}H NMR Spectrum of Compound **56** (400 MHz, $CDCl_3$)

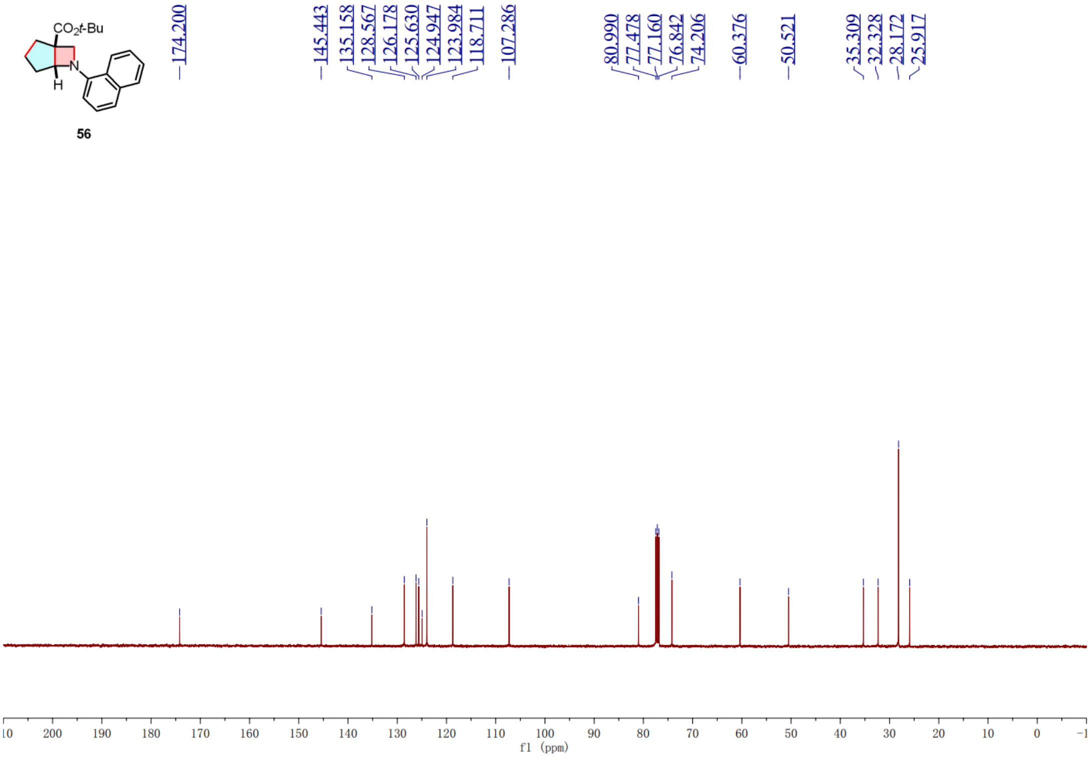

^{13}C{^{1}H} NMR Spectrum of Compound **56** (100 MHz, $CDCl_3$)

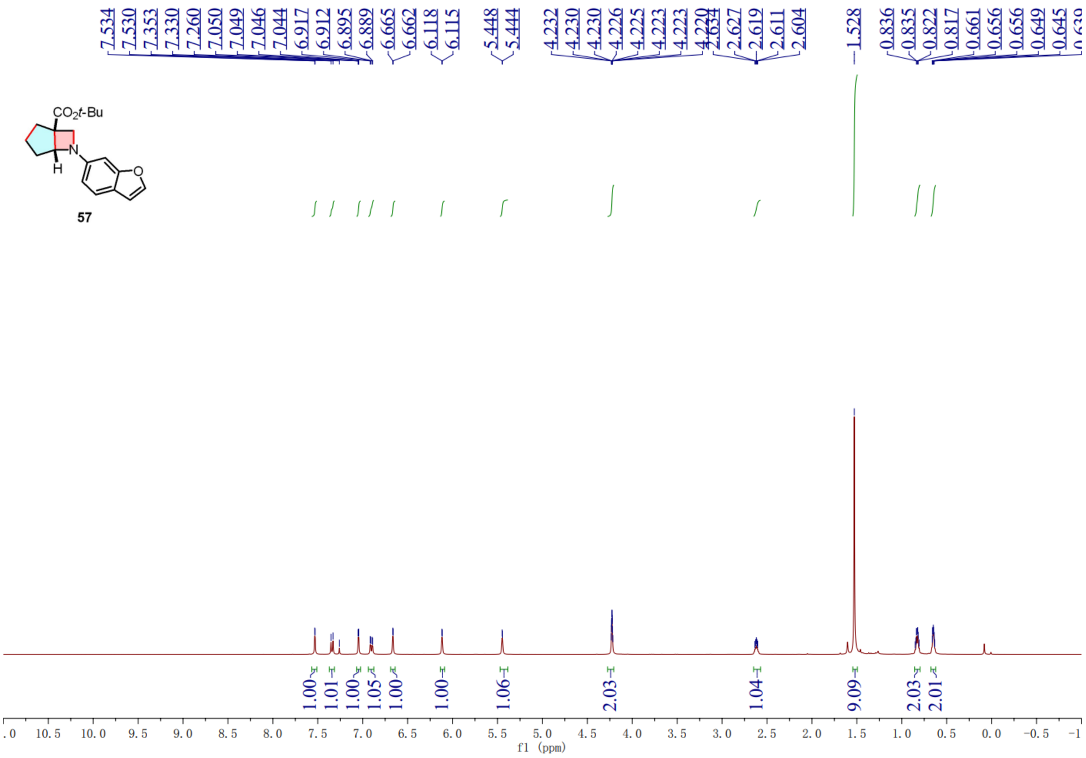

^{1}H NMR Spectrum of Compound **57** (400 MHz, $CDCl_3$)

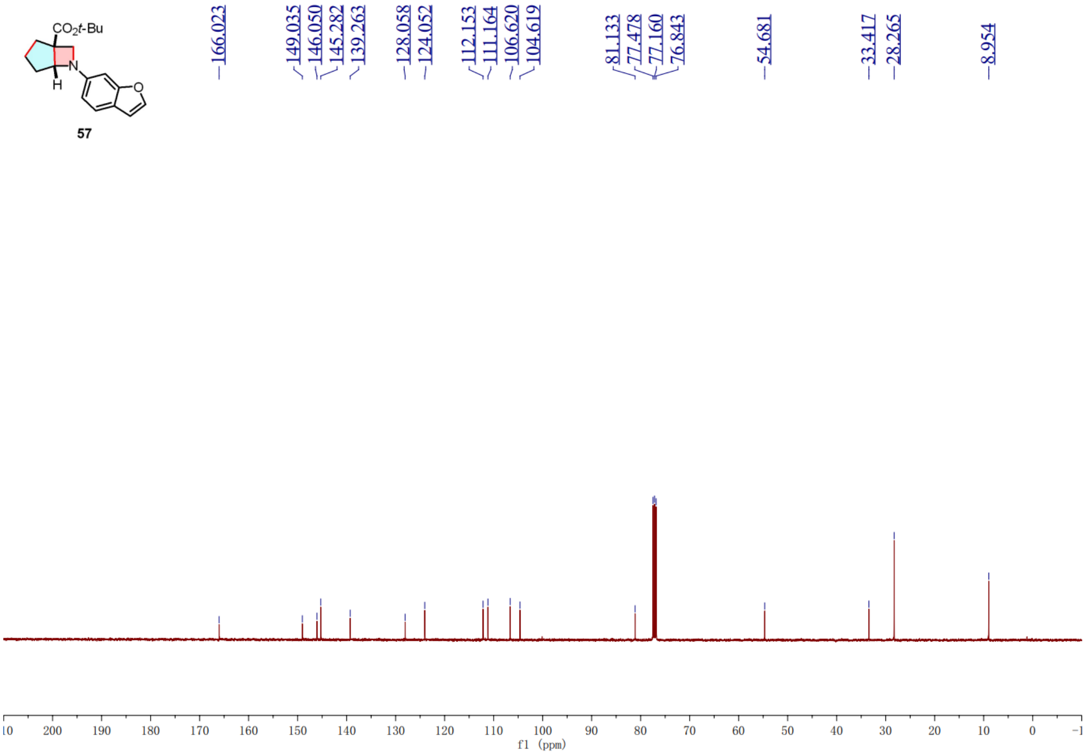

^{13}C{^{1}H} NMR Spectrum of Compound **57**(100 MHz, $CDCl_3$)

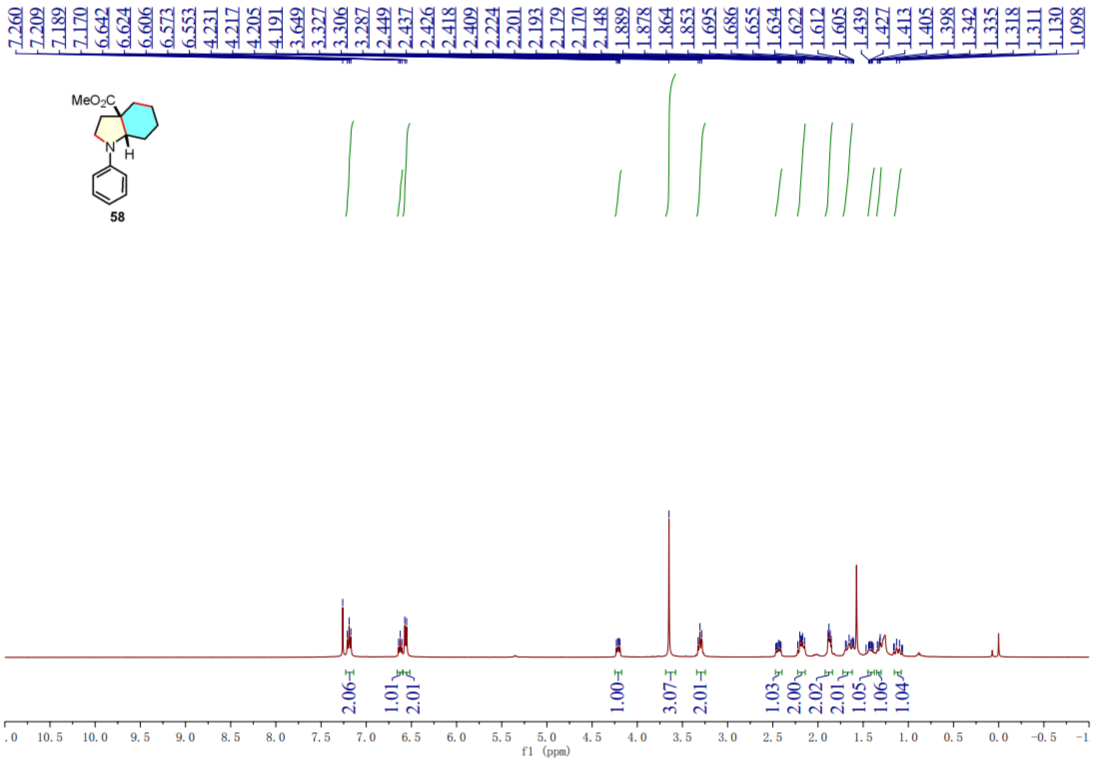

^{1}H NMR Spectrum of Compound **58** (400 MHz, $CDCl_3$)

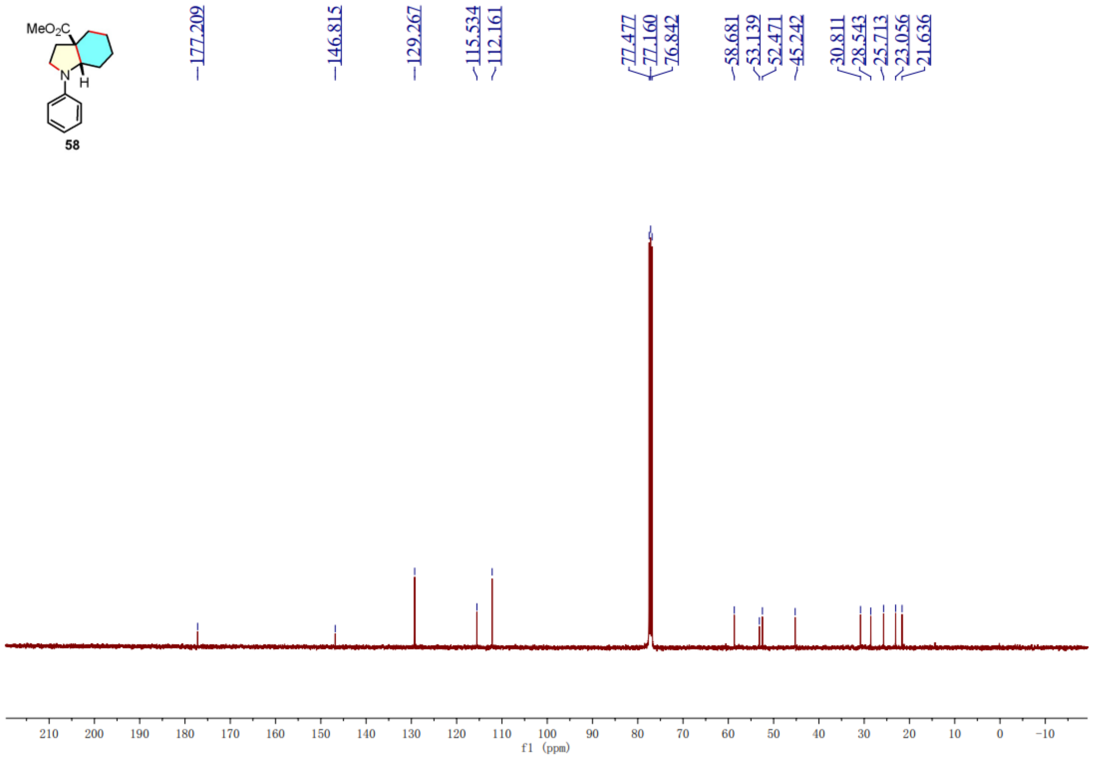

^{13}C{^{1}H} NMR Spectrum of Compound **58**(100 MHz, $CDCl_3$)

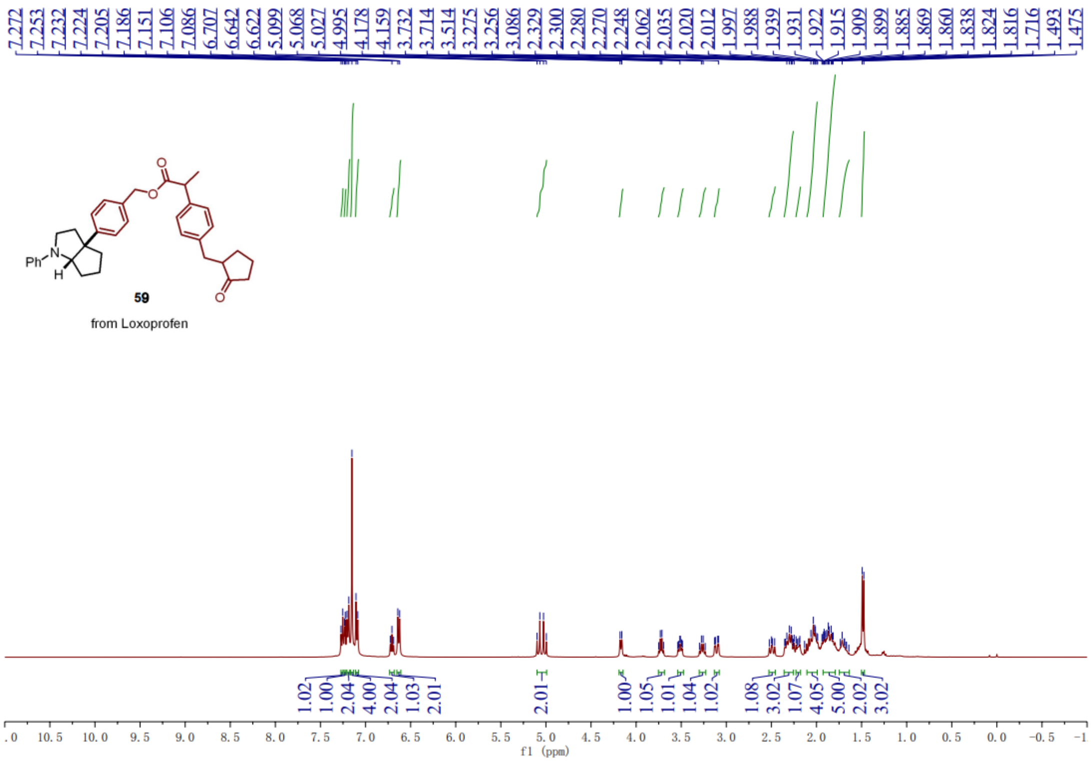

^{1}H NMR Spectrum of Compound **59** (400 MHz, $CDCl_3$)

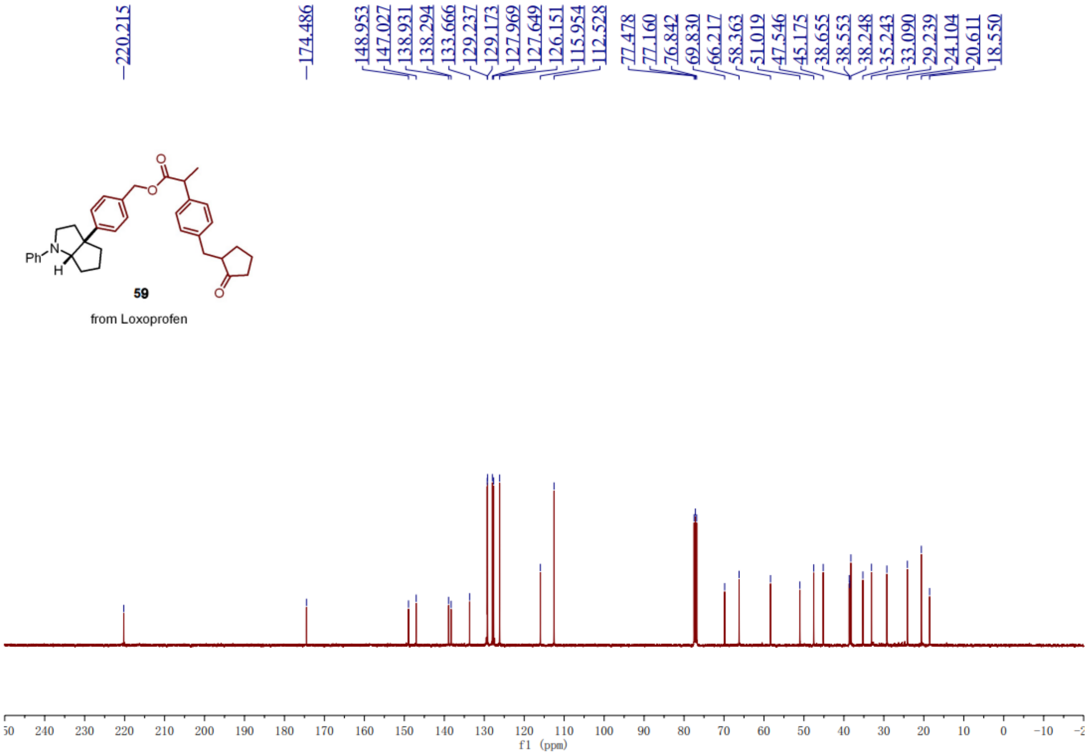

^{13}C{^{1}H} NMR Spectrum of Compound **59** (100 MHz, $CDCl_3$)

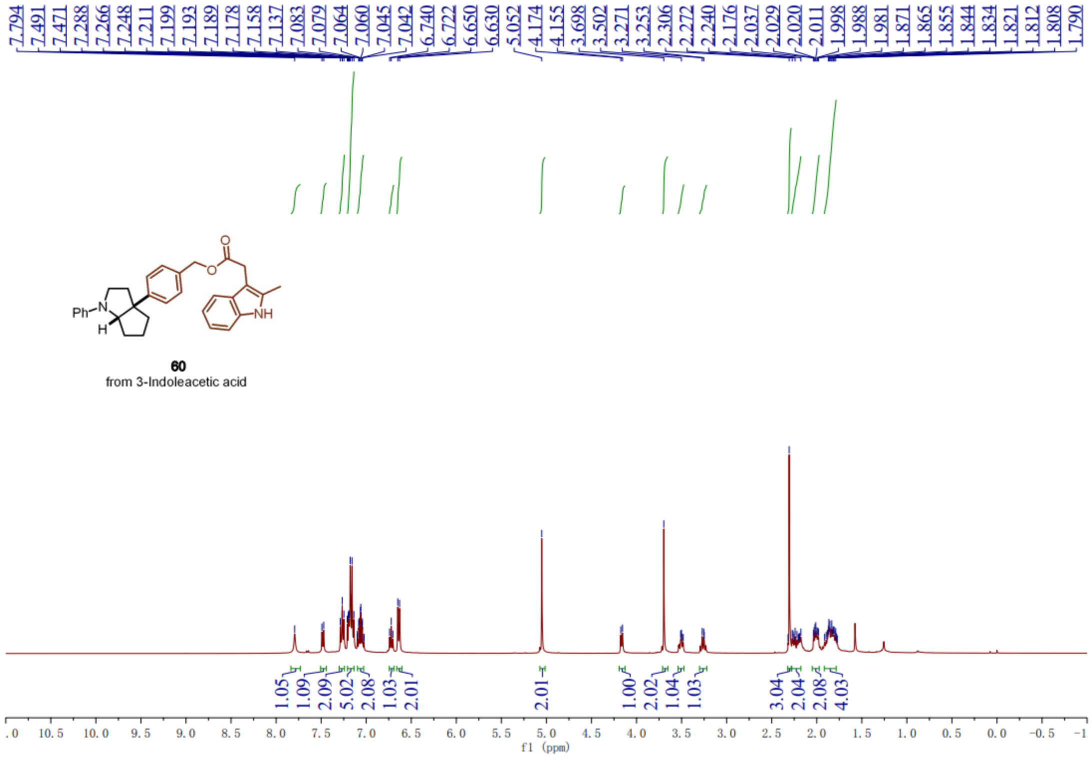

^{1}H NMR Spectrum of Compound **60** (400 MHz, $CDCl_3$)

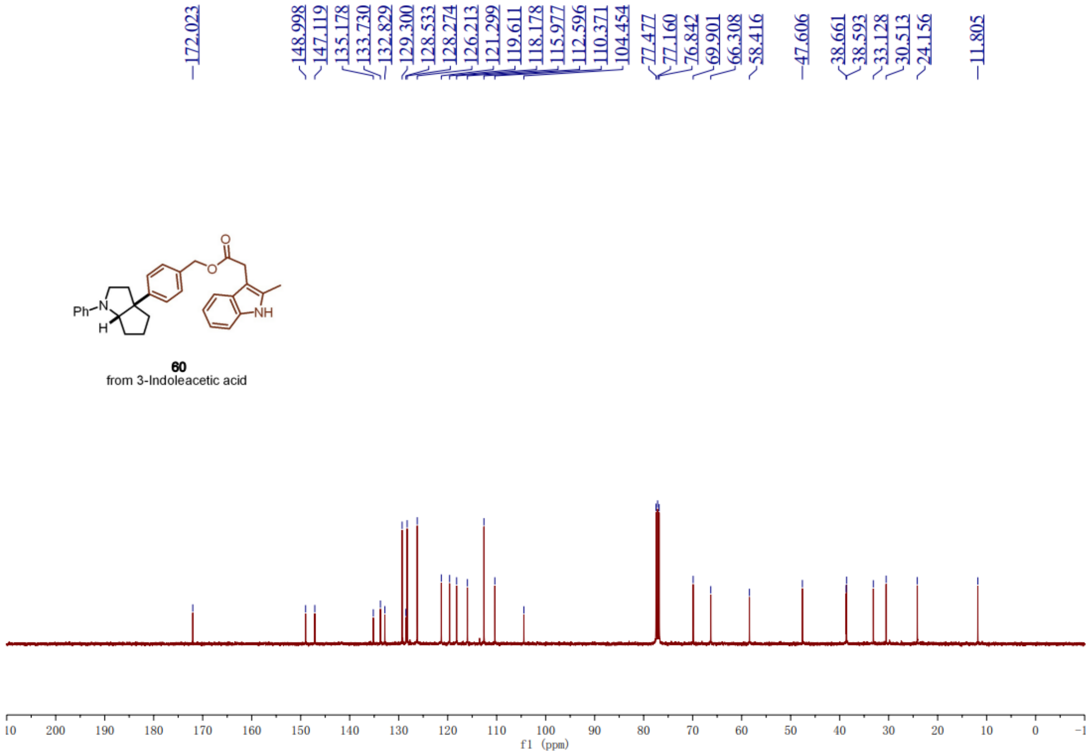

^{13}C{^{1}H} NMR Spectrum of Compound **60** (100 MHz, $CDCl_3$)

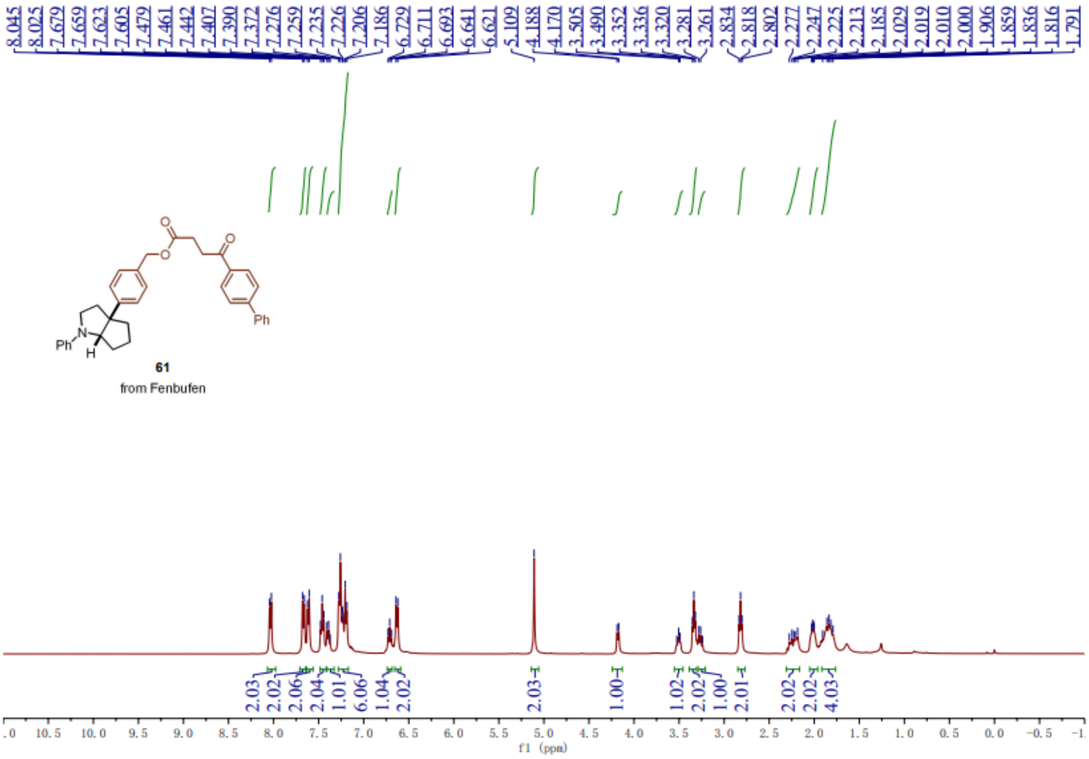

^{1}H NMR Spectrum of Compound **61** (400 MHz, $CDCl_3$)

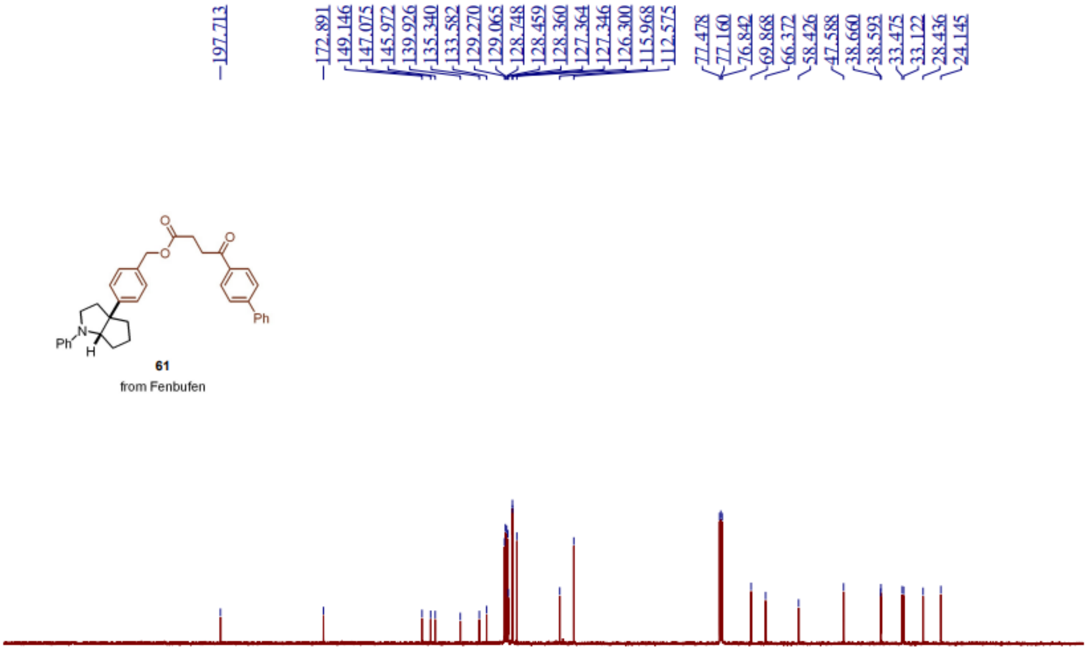

^{13}C{^{1}H} NMR Spectrum of Compound **61** (100 MHz, $CDCl_3$)

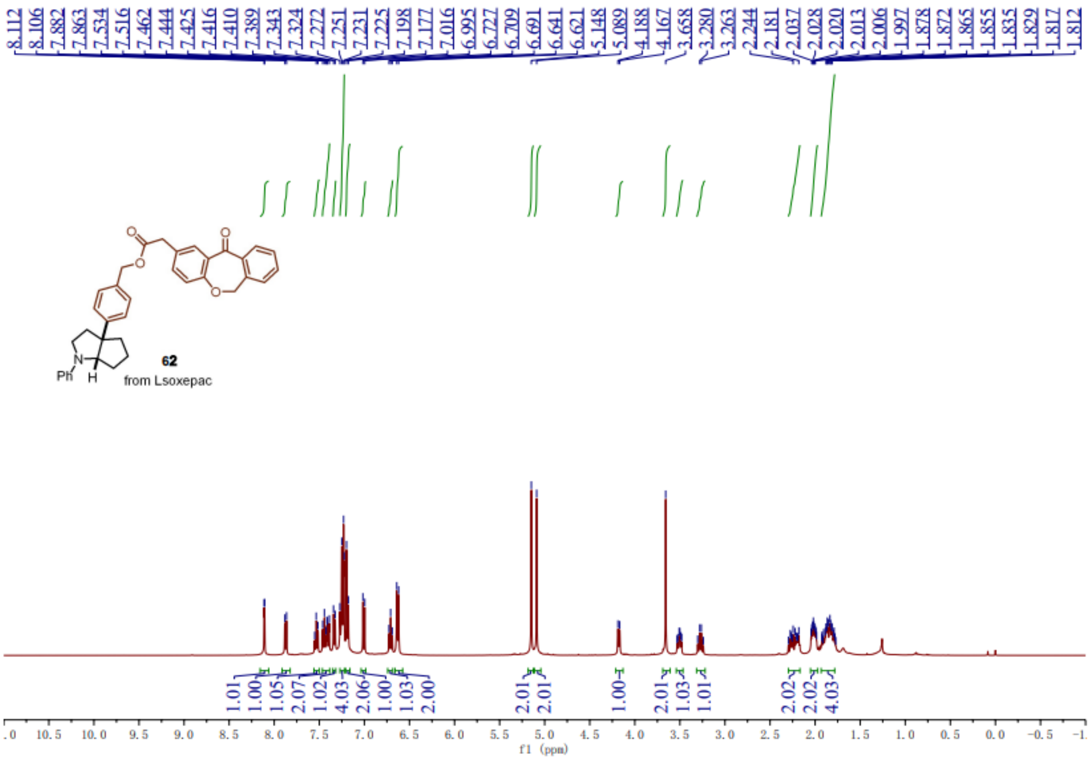

^{1}H NMR Spectrum of Compound **62** (400 MHz, $CDCl_3$)

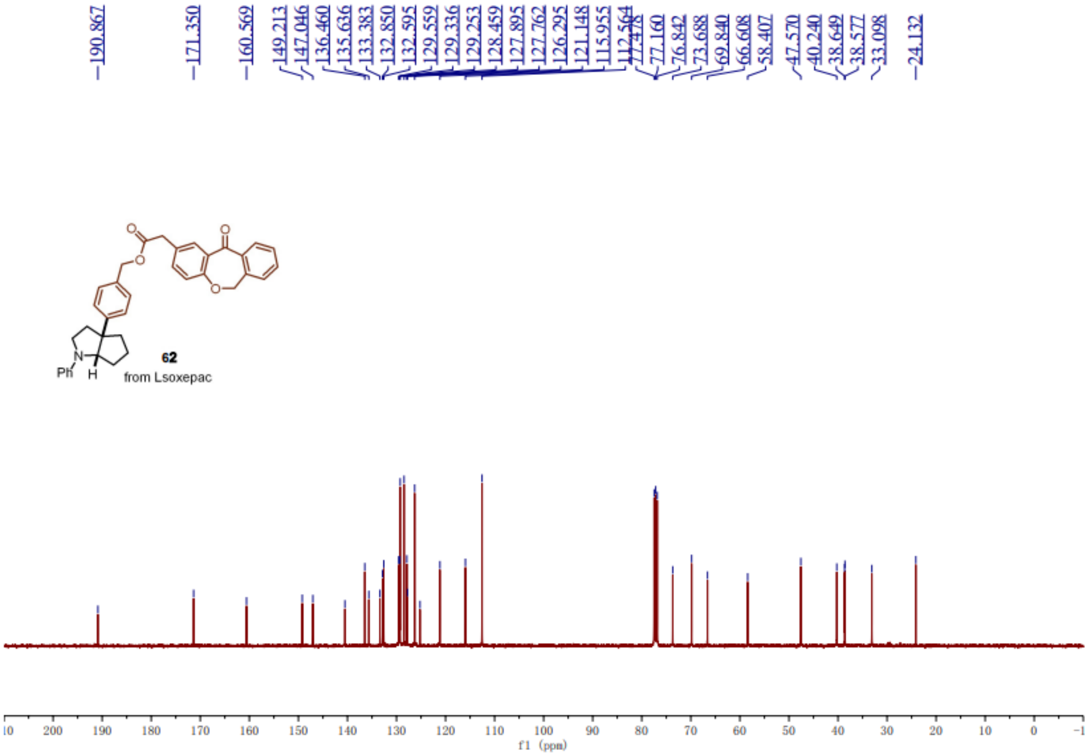

$^{13}C\{^1H\}$ NMR Spectrum of Compound **62** (100 MHz, $CDCl_3$)

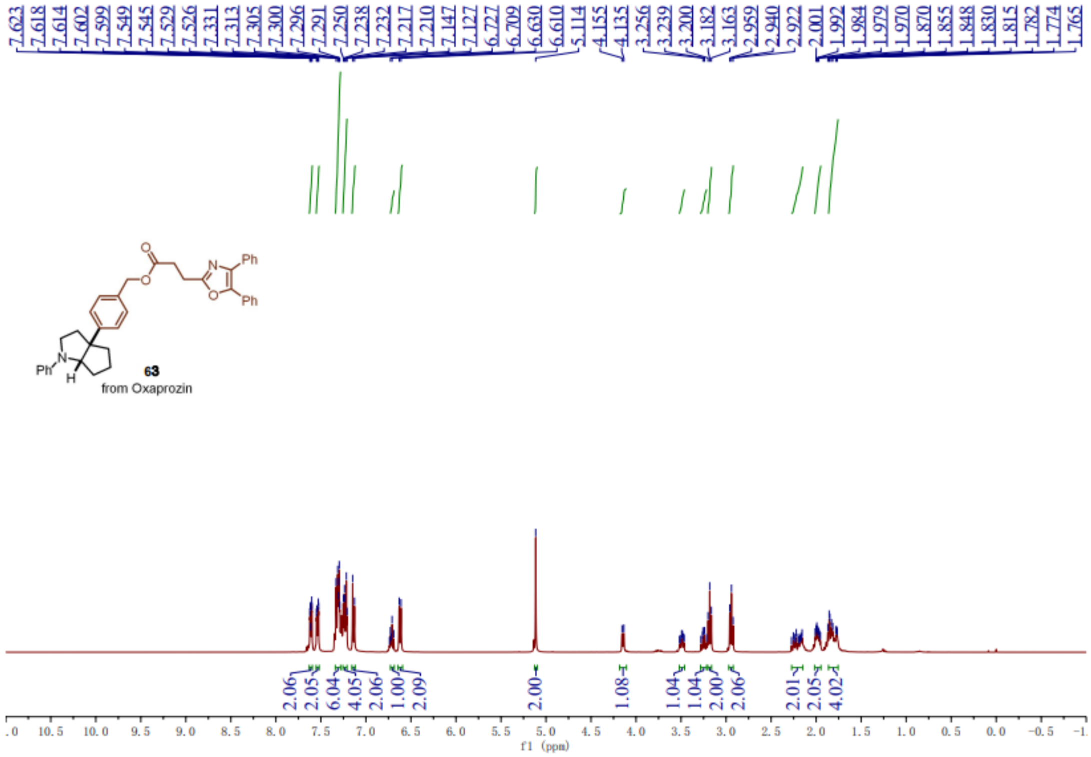

^{1}H NMR Spectrum of Compound **63** (400 MHz, $CDCl_3$)

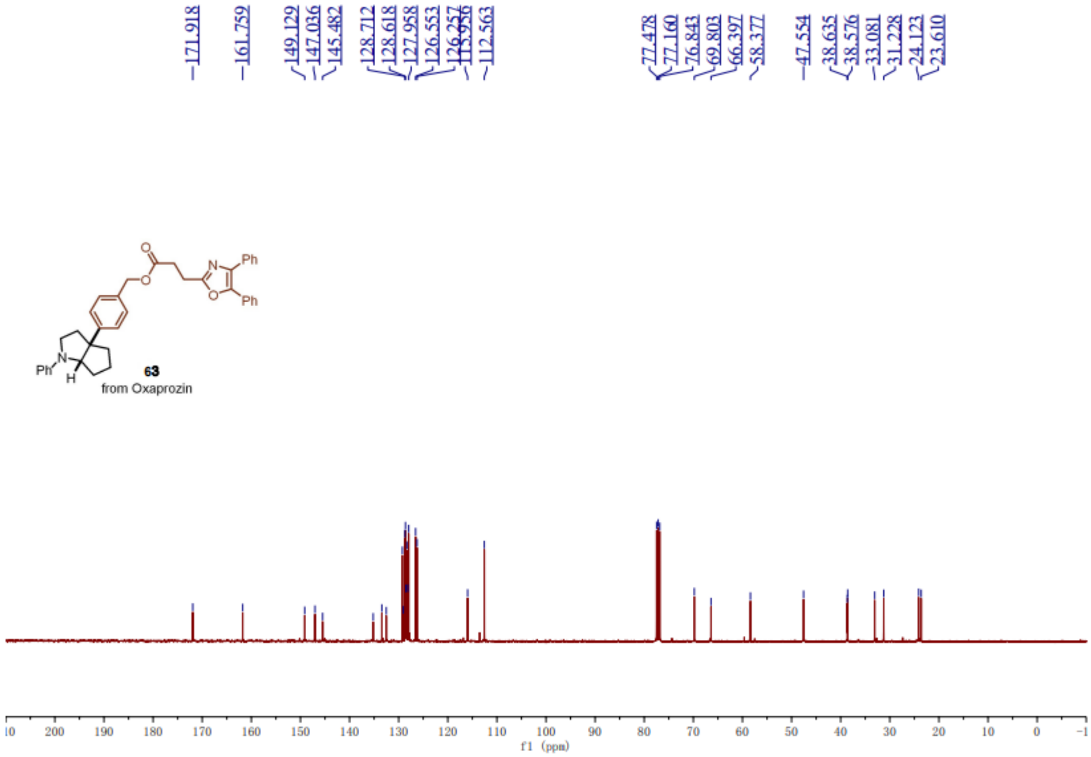

^{13}C{^{1}H} NMR Spectrum of Compound **63** (100 MHz, $CDCl_3$)

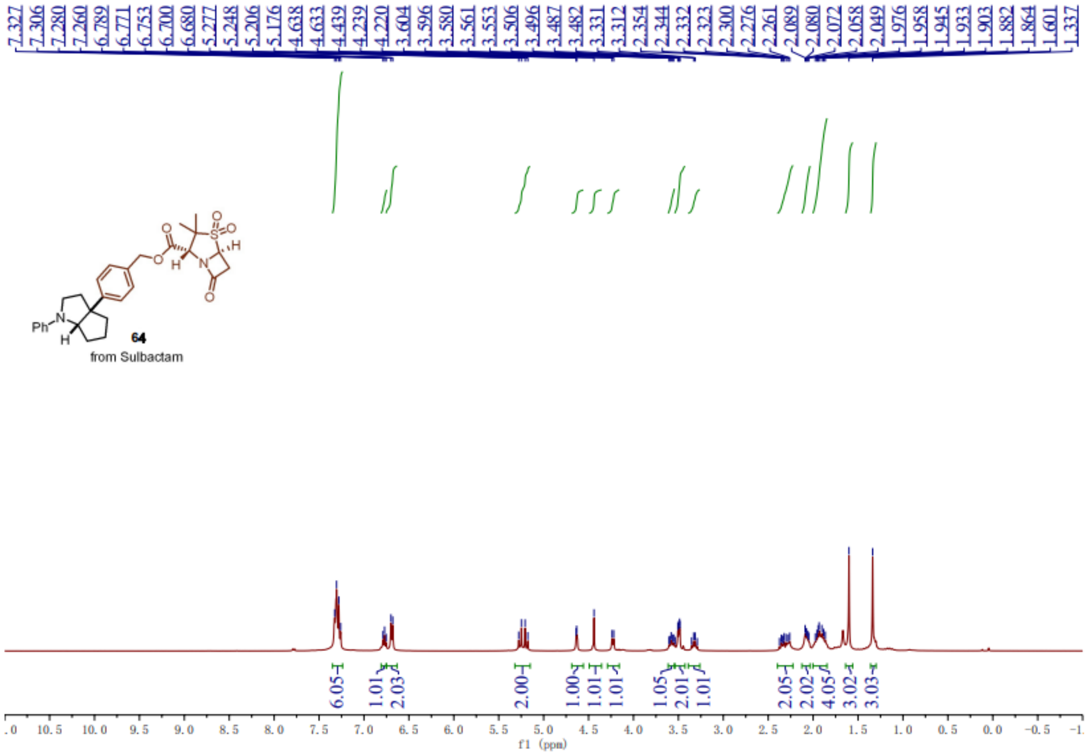

^{1}H NMR Spectrum of Compound **64** (400 MHz, $CDCl_3$)

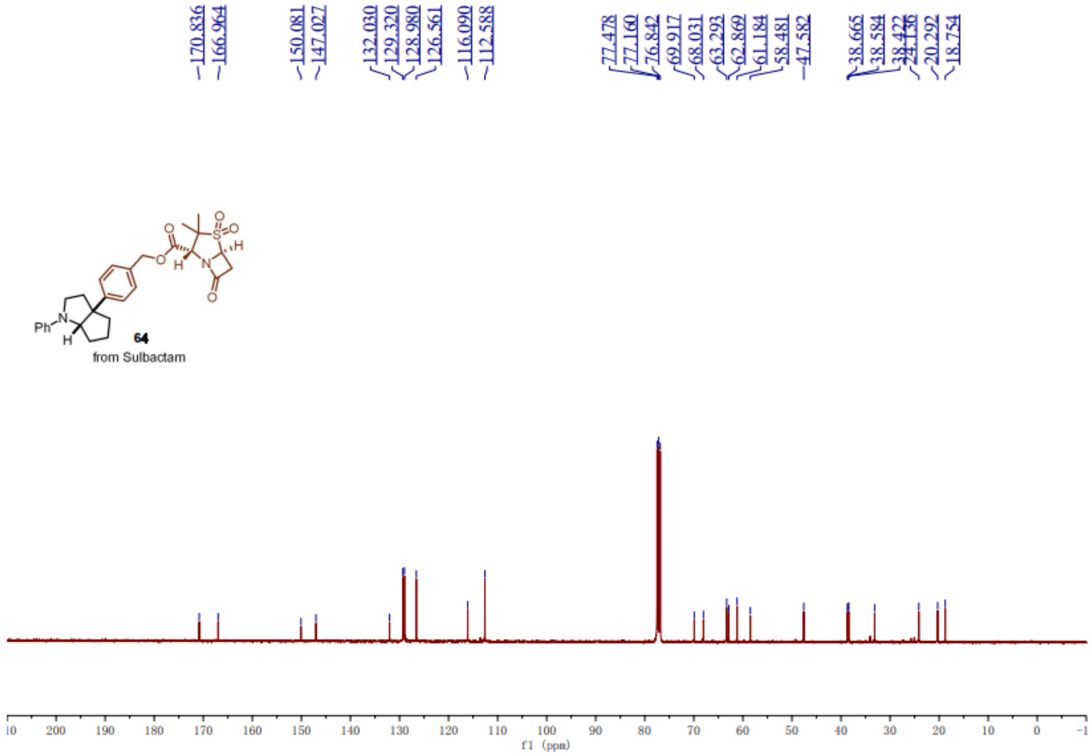

^{13}C{^{1}H} NMR Spectrum of Compound **64** (100 MHz, $CDCl_3$)

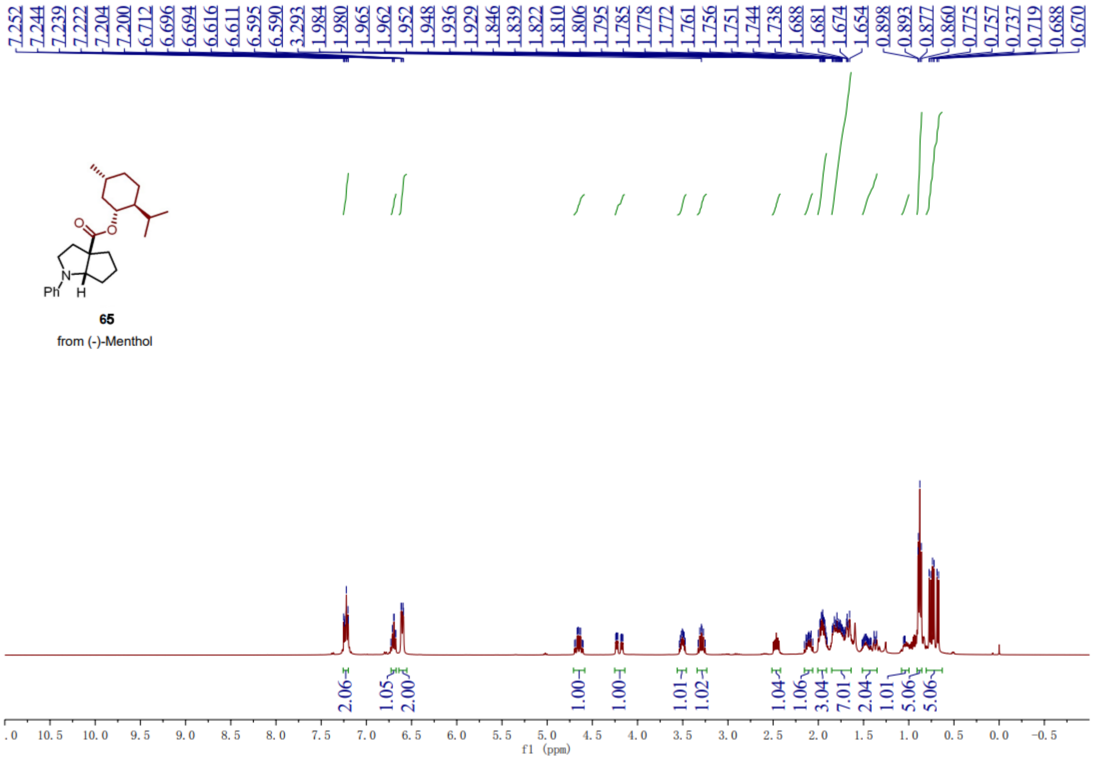

^{1}H NMR Spectrum of Compound **65** (400 MHz, $CDCl_3$)

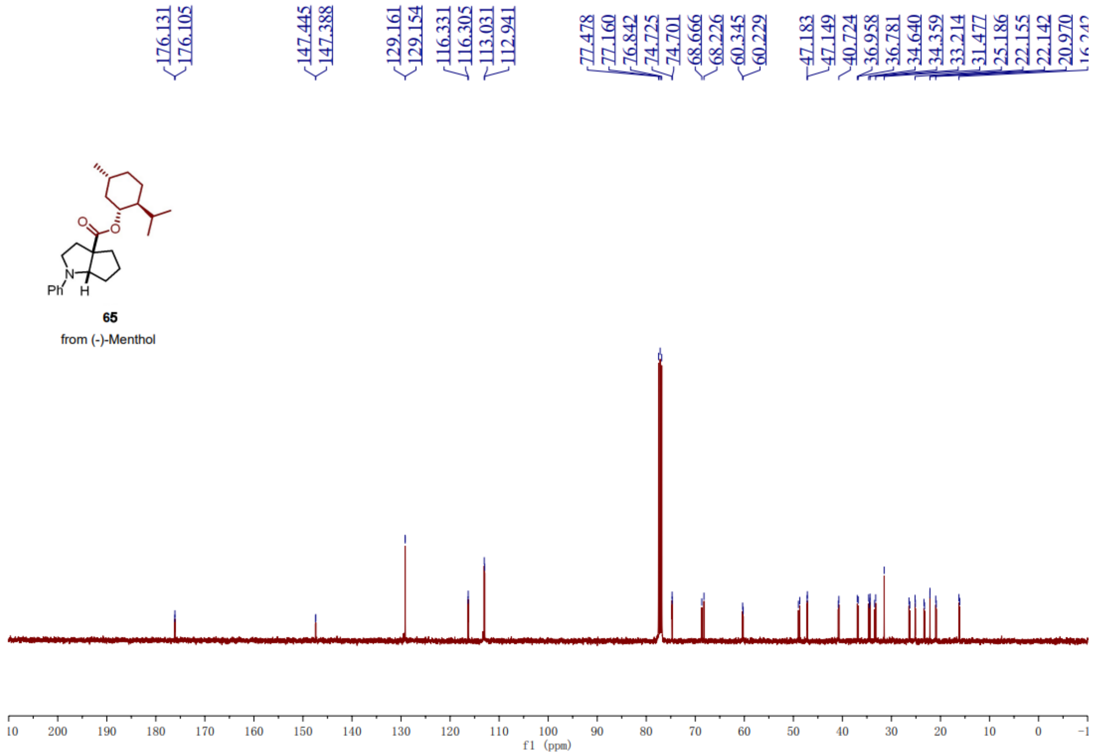

^{13}C{^{1}H} NMR Spectrum of Compound **65** (100 MHz, $CDCl_3$)

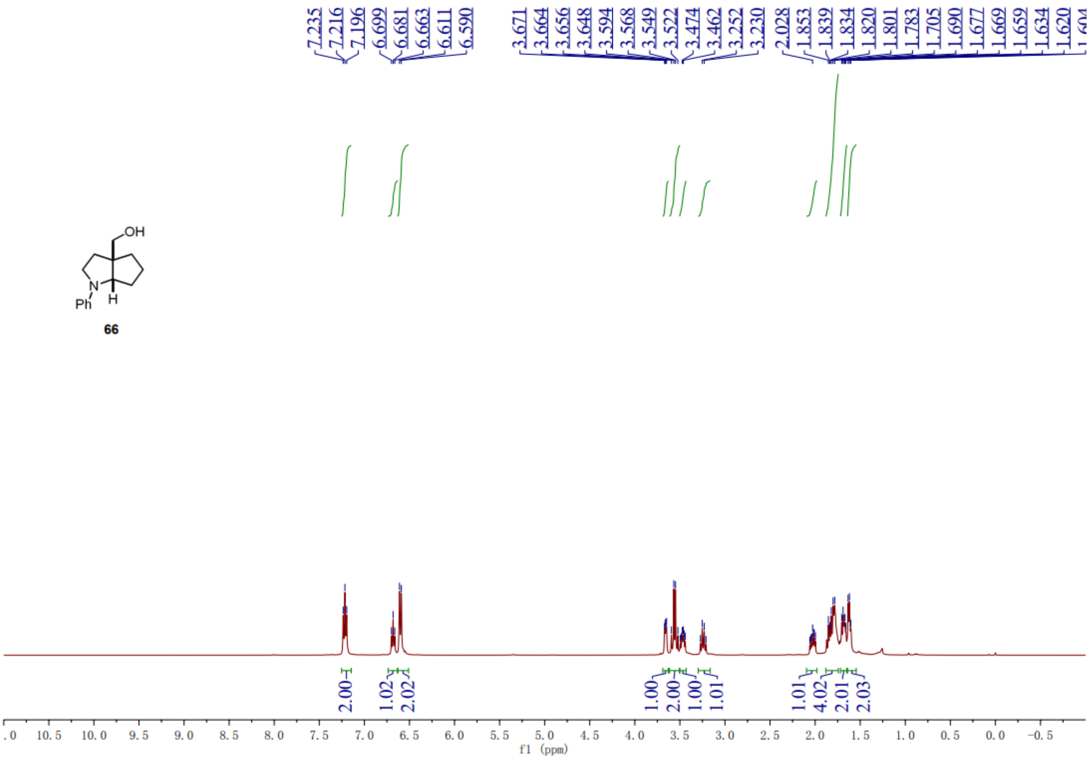

^{1}H NMR Spectrum of Compound **66** (400 MHz, $CDCl_3$)

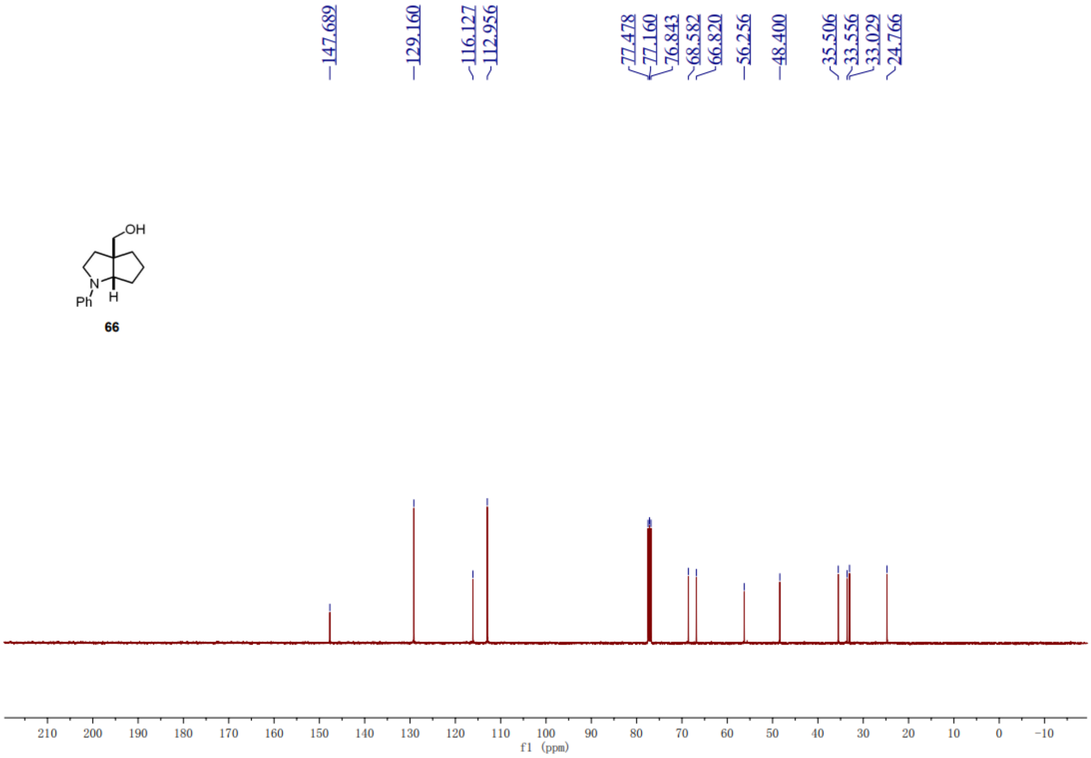

^{13}C{^{1}H} NMR Spectrum of Compound **66** (100 MHz, $CDCl_3$)

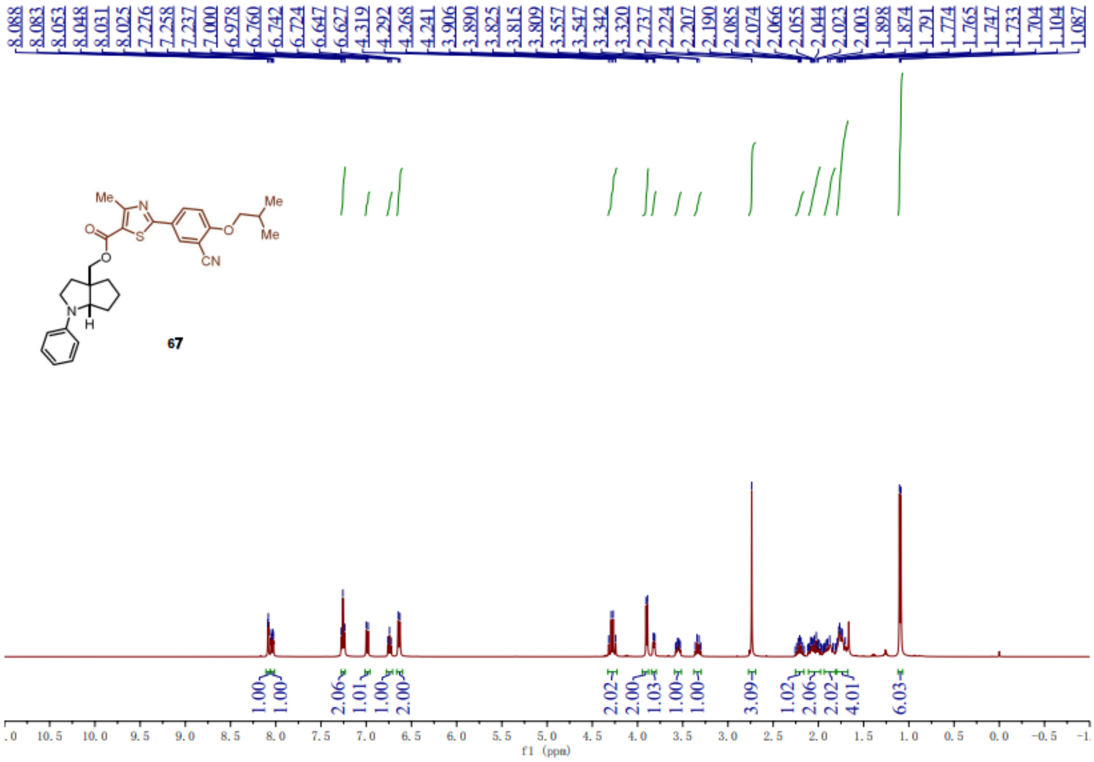

^{1}H NMR Spectrum of Compound **67** (400 MHz, $CDCl_3$)

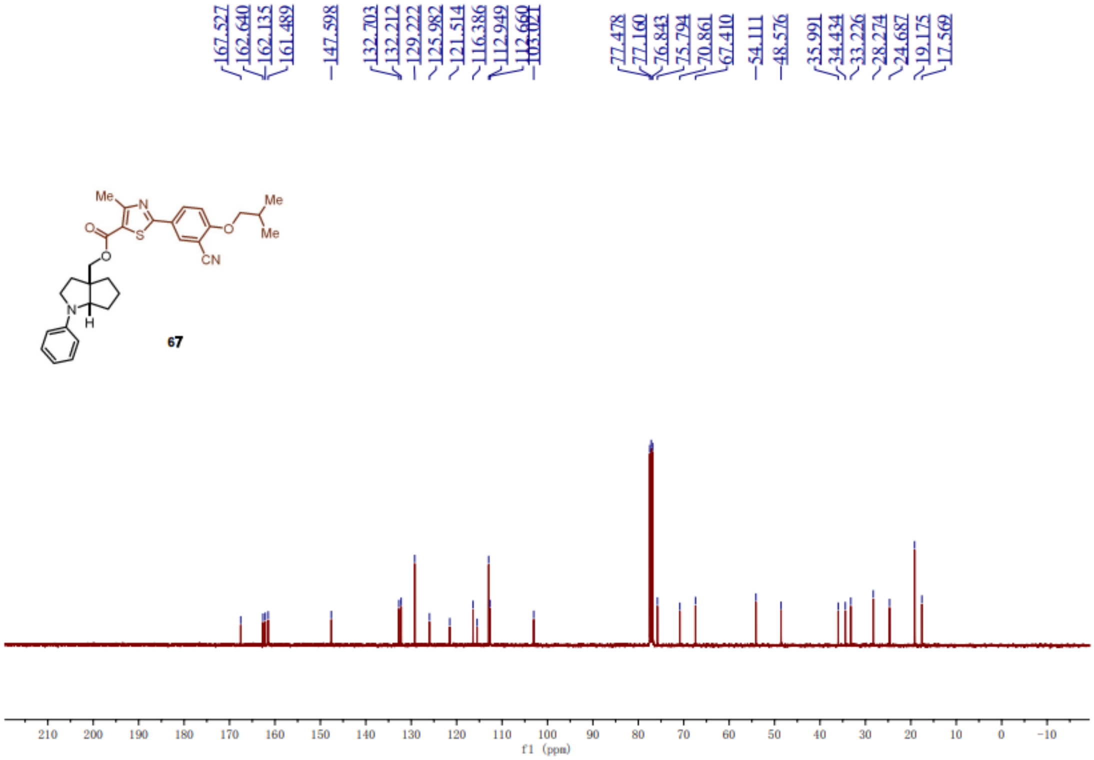

^{13}C{^{1}H} NMR Spectrum of Compound **67** (100 MHz, $CDCl_3$)

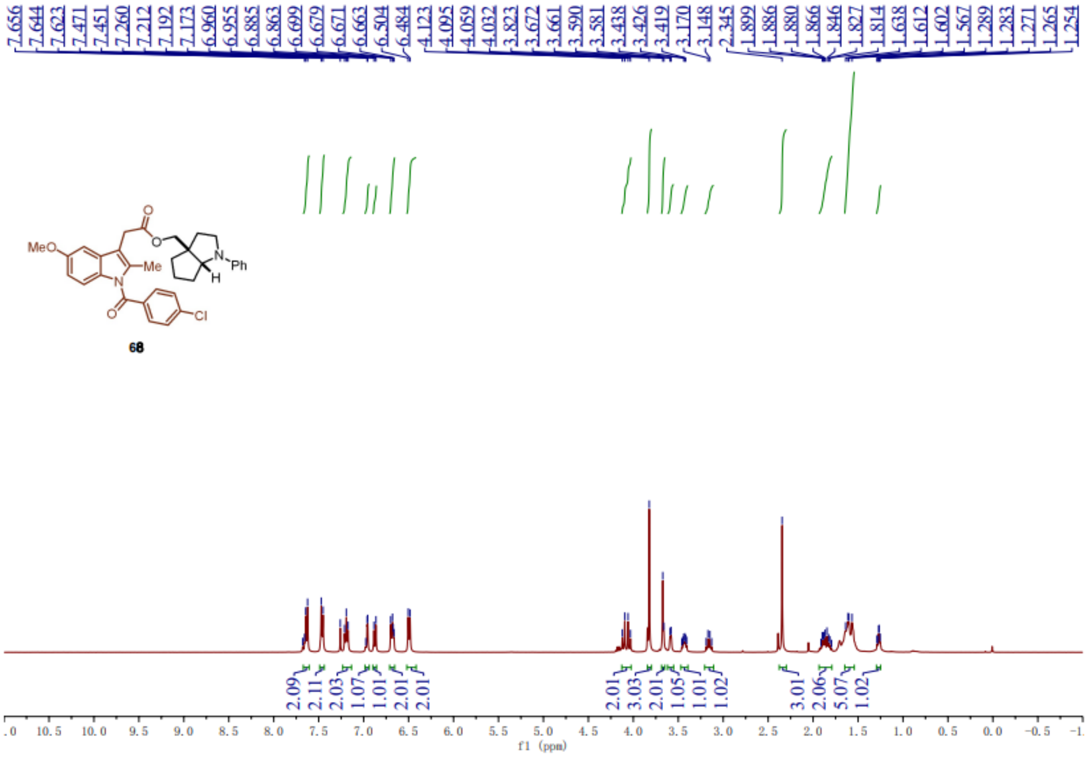

^{1}H NMR Spectrum of Compound **68** (400 MHz, $CDCl_3$)

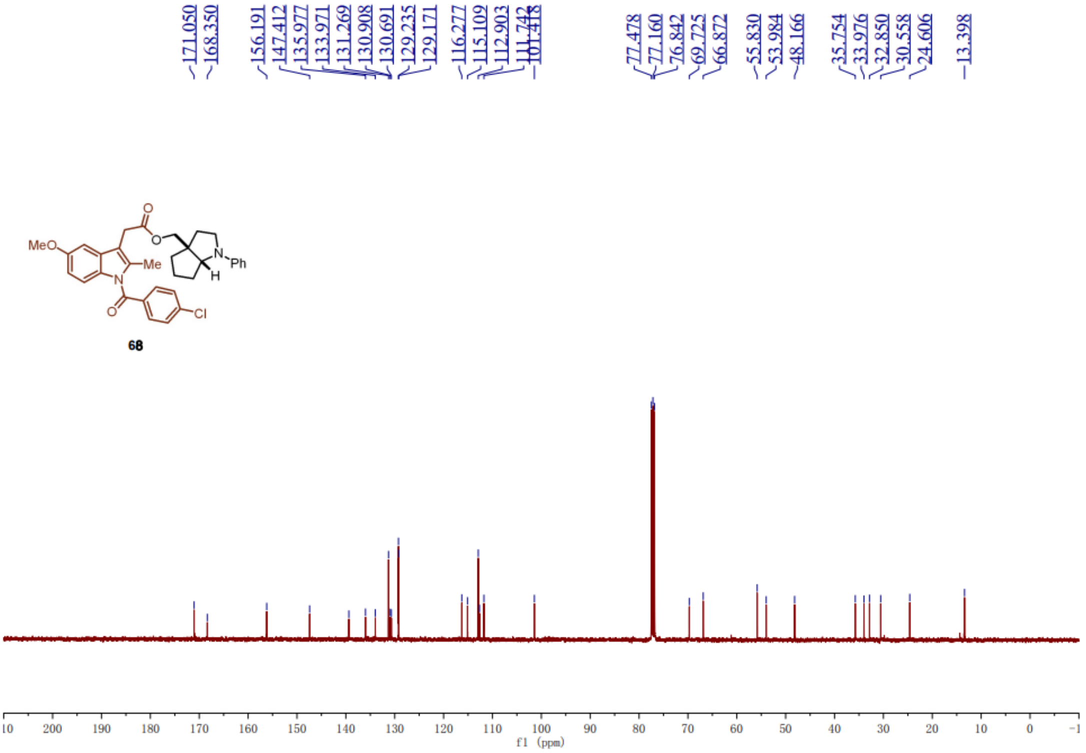

^{13}C{^{1}H} NMR Spectrum of Compound **68** (100 MHz, $CDCl_3$)

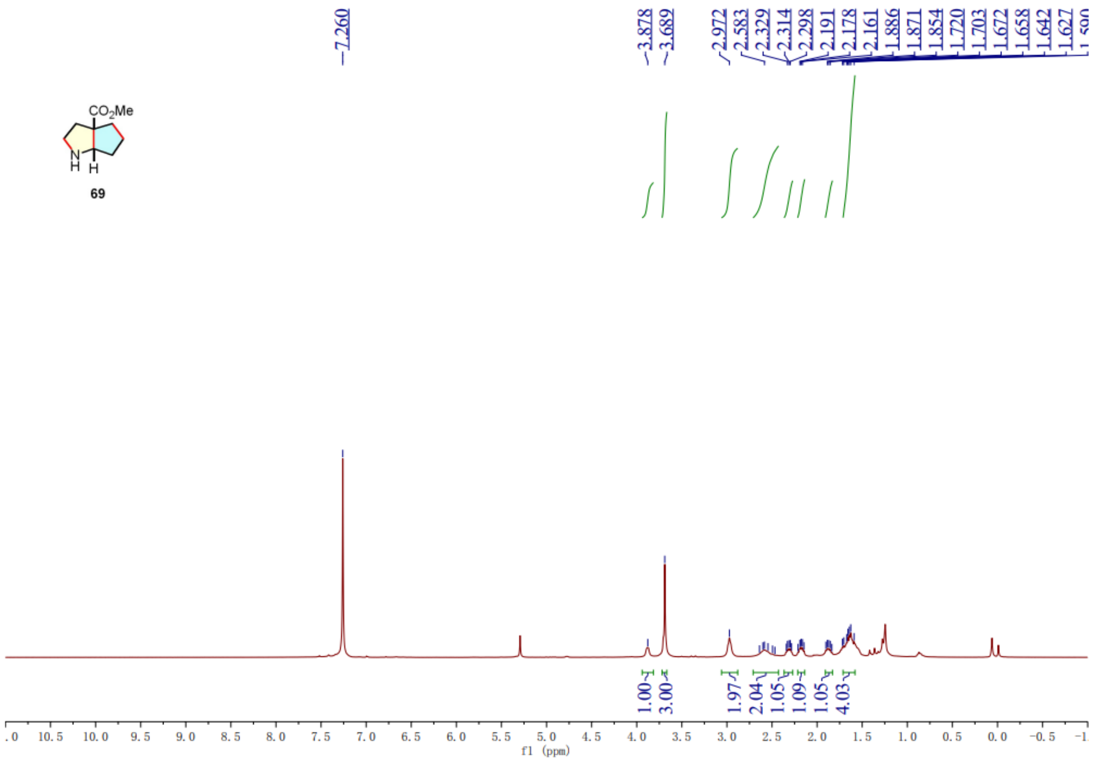

^{1}H NMR Spectrum of Compound **69** (400 MHz, $CDCl_3$)

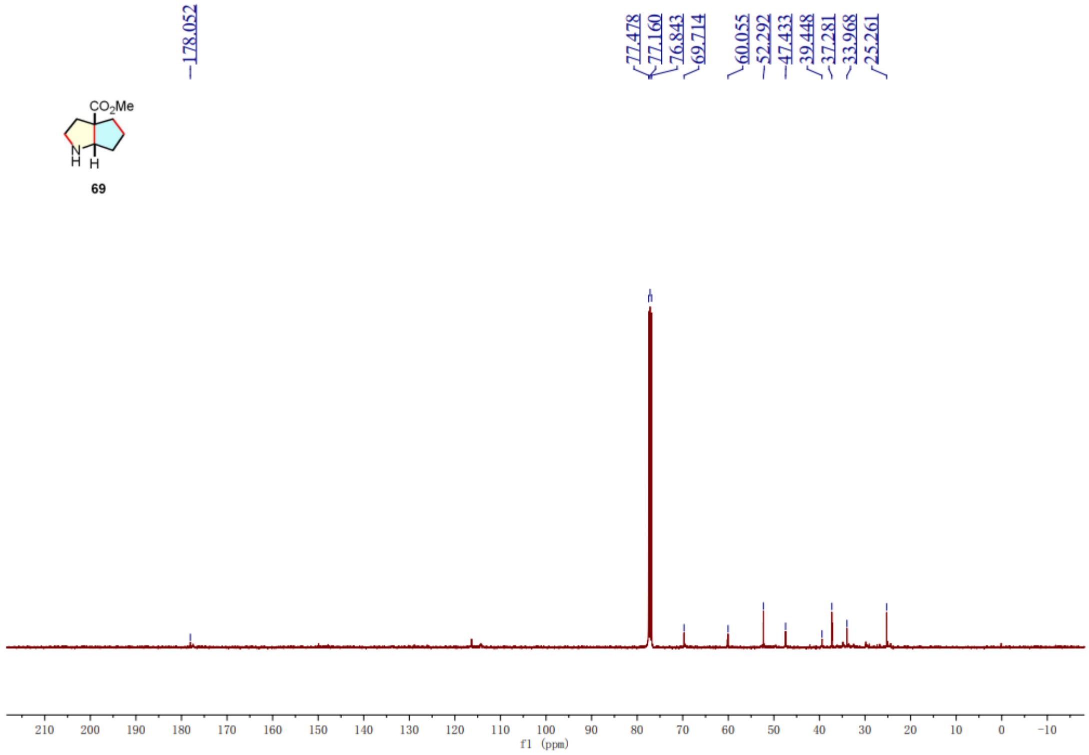

$^{13}C\{^{1}H\}$ NMR Spectrum of Compound **69** (100 MHz, $CDCl_3$)

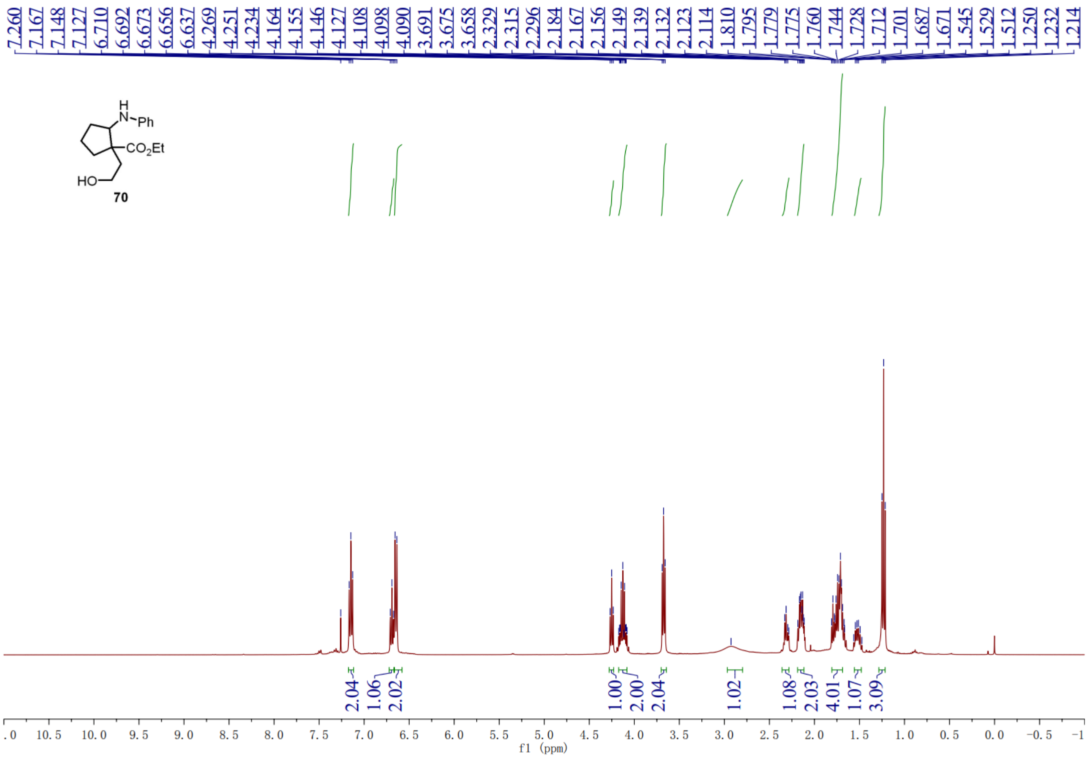

^{1}H NMR Spectrum of Compound **70** (400 MHz, $CDCl_3$)

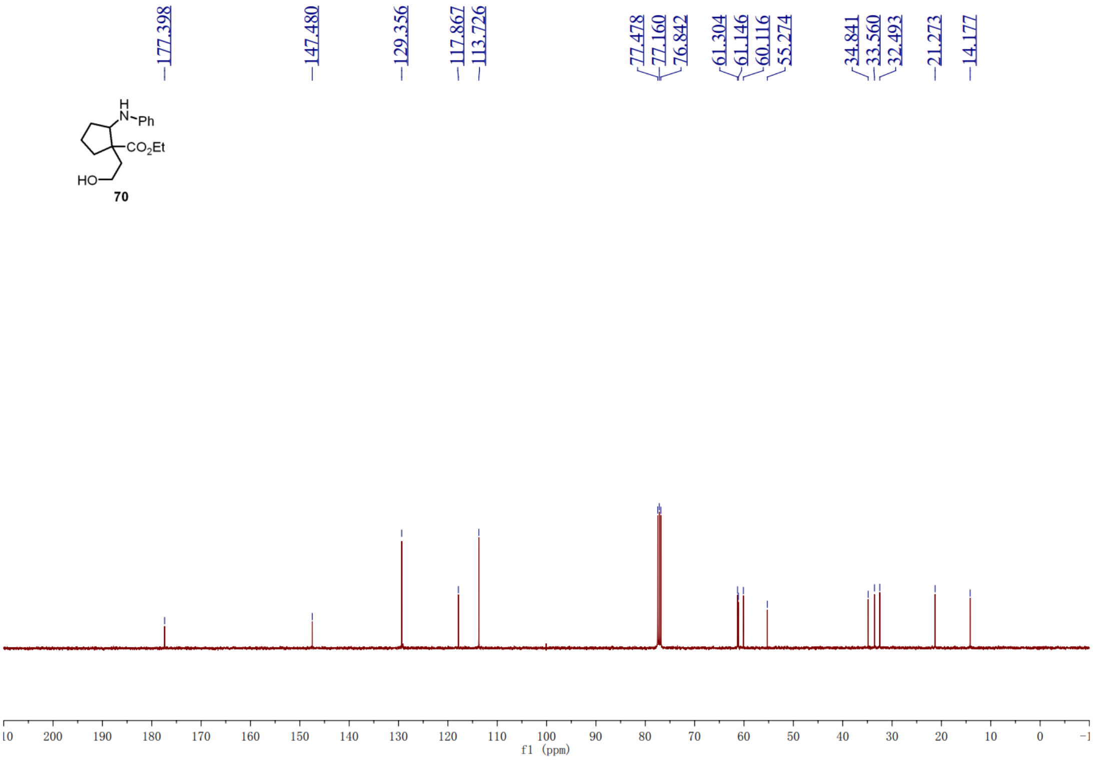

^{13}C{^{1}H} NMR Spectrum of Compound **70** (100 MHz, $CDCl_3$)
